# Synthesis and Biological Activity of Brassinosteroid Analogues with a Nitrogen-Containing Side Chain

**DOI:** 10.3390/ijms22010155

**Published:** 2020-12-25

**Authors:** Mikhail V. Diachkov, Karoll Ferrer, Jana Oklestkova, Lucie Rarova, Vaclav Bazgier, Miroslav Kvasnica

**Affiliations:** 1 Laboratory of Growth Regulators, The Czech Academy of Sciences, Institute of Experimental Botany & Palacký University, Šlechtitelů 27, 78371 Olomouc, Czech Republic; misha_d84@mail.ru (M.V.D.); karoll.ferrer.14@sansano.usm.cl (K.F.); jana.oklestkova@upol.cz (J.O.); lucie.rarova@upol.cz (L.R.); vaclav.bazgier@upol.cz (V.B.); 2 Department of Physical Chemistry, Faculty of Science, Palacký University Olomouc, Šlechtitelů 241/27, 77900 Olomouc, Czech Republic

**Keywords:** brassinosteroid, organic synthesis, nitrogen-containing steroid, plant bioassay, cytotoxicity

## Abstract

Brassinosteroids are a class of plant hormones that regulate a broad range of physiological processes such as plant growth, development and immunity, including the suppression of biotic and abiotic stresses. In this paper, we report the synthesis of new brassinosteroid analogues with a nitrogen-containing side chain and their biological activity on *Arabidopis thaliana*. Based on molecular docking experiments, two groups of brassinosteroid analogues were prepared with short and long side chains in order to study the impact of side chain length on plants. The derivatives with a short side chain were prepared with amide, amine and ammonium functional groups. The derivatives with a long side chain were synthesized using amide and ammonium functional groups. A total of 25 new brassinosteroid analogues were prepared. All 25 compounds were tested in an Arabidopsis root sensitivity bioassay and cytotoxicity screening. The synthesized substances showed no significant inhibitory activity compared to natural 24-epibrassinolide. In contrast, in low concentration, several compounds (8a, 8b, 8e, 16e, 22a and 22e) showed interesting growth-promoting activity. The cytotoxicity assay showed no toxicity of the prepared compounds on cancer and normal cell lines.

## 1. Introduction

**Figure 1:**
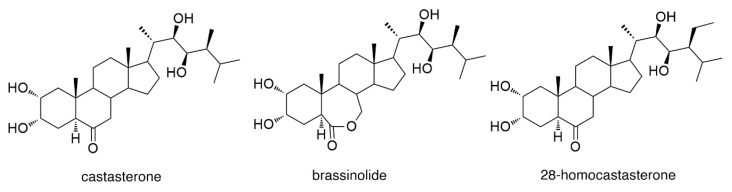
Structure of the most common natural brassinosteroids.

Brassinosteroids (BRs, Figure 1) are a group of plant steroid hormones that regulate many important aspects of plant growth and development, such as cell division, elongation and differentiation, pollen tube growth, seed germination, regulation of gene expression, enzyme activation and photosynthesis [1,2,3]. They also induce tolerance against a wide range of biotic and abiotic stresses, such as water or drought, temperature, oxidative stresses, high salinity and different environmental pollutants [4,5]. At the molecular level, BRs modify the pathway of enzymes and change the gene expression when plants are exposed to stress. Plants perceive brassinosteroids at the cell membrane, using the membrane-integral receptor kinase brassinosteroid insensitive 1 (BRI1) and its co-receptor BAK1 [6,7,8,9]. The encoded protein, BRI1, is a member of a large family of plant LRR (leucine-rich repeat) receptor-like kinases, characterized by an extracellular LRR domain, a single-pass transmembrane segment and a cytoplasmic kinase domain. BRI1 has been established as an authentic brassinosteroid receptor through genetic and biochemical investigations [10]. Moreover, recent studies have shown that natural BRs and their synthetic analogues have potential application not only in agriculture, but also in medicine due to their antiviral [11,12], immunomodulatory and neuroprotective activities [13,14,15], and anti-proliferative effects on animal cells in vitro [16,17,18,19,20]. 

Since the first BR isolation and structural identification, chemists began to synthesize new brassinosteroid analogues with different modifications of the sterol skeleton and tested them on various biological activities, especially in plant bioassays. Modification of the side chain is one of the main aims of synthesis. Such modifications include shortening or prolonging the side chain, oxidation or reduction of oxygen functional groups, incorporation of heteroatoms other than oxygen (mostly nitrogen and halogens) and modification of newly synthesized functional groups (e.g., acetylation, methylation, etc.) [21,22,23,24,25,26,27,28]. Preparation of derivatives with a nitrogen-containing side chain has not been at the forefront of the interest of steroidal chemists. Few examples have been published so far. Back et al. described the synthesis and biological activities of 25-azabrassinolide. Unfortunately, the rice lamina inclination biotest showed no biological activity [29]. Several BR analogues with nitrogen-containing heterocycles (e.g., isoxazoline, oxazole, thiazole and pyridine) were synthesized by Wendeborn’s group [25]. Other analogues with nitrogen groups in the side chain were prepared mostly for other chemical modifications, e.g., azide derivatives for photoaffinity labeling [30] or activated esters for conjugation reactions [31,32]. 

The aim of this study was to synthesize new BRs analogues with nitrogen functional groups (amides, amines and ammonium salts) in the side chain and study their biological properties. The synthesis was also based on molecular docking. It uses computational methods for prediction of interaction between receptors and ligands. It is a tool for predicting the first results during the preparation of new ligands. In our study, we were interested mostly in tertiary amides and amines to avoid the presence of hydrogen directly attached to nitrogen (with the exception of hydrochloride salts of tertiary amines whose existence is pH-dependent) and because they have an additional hydrogen bond acceptor than standard BRs hydroxy groups. The plant growth-promoting activity of synthetic analogues was assayed using the Arabidopsis root sensitivity bioassay. Further, the cytotoxic activity of all compounds was tested on one normal and three cancer cell lines. 

## 2. Results and Discussion

### 2.1. Chemistry

**Scheme 1:**
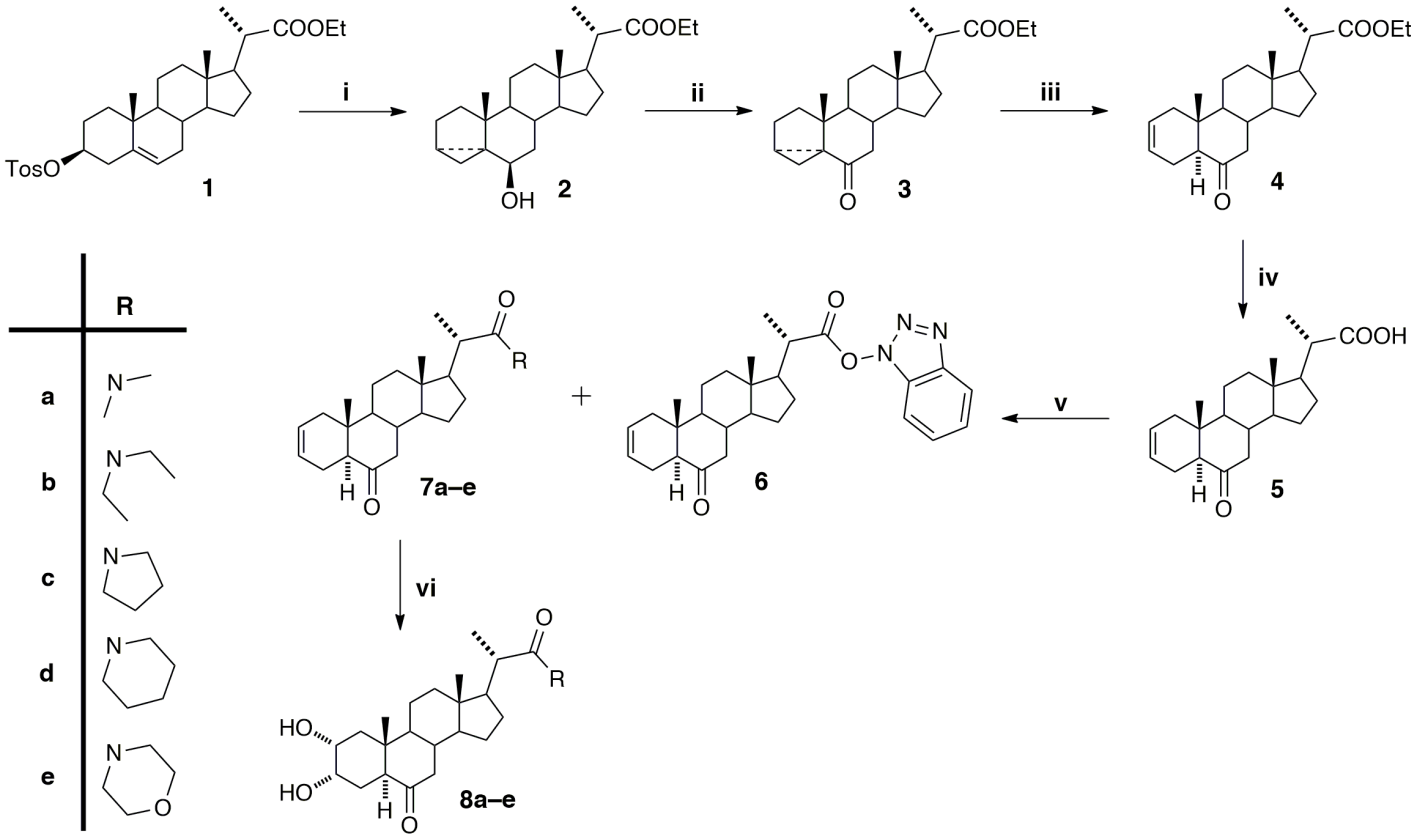
Synthesis of short side chain amides: (i) AcOK, acetone, H_2_O, reflux.; (ii) Jones reagent, acetone, 0 °C; (iii) LiBr, PPTS, dimethylacetamide, reflux; (iv) KOH, EtOH, H_2_O, reflux; (v) amine, HOBt (hydroxybenzotriazole), EDC, DMF, 40 °C; (vi) OsO_4_, *N*-Me-Mf-*N*-oxide, THF, acetone, H_2_O, r.t.

**Scheme 2:**
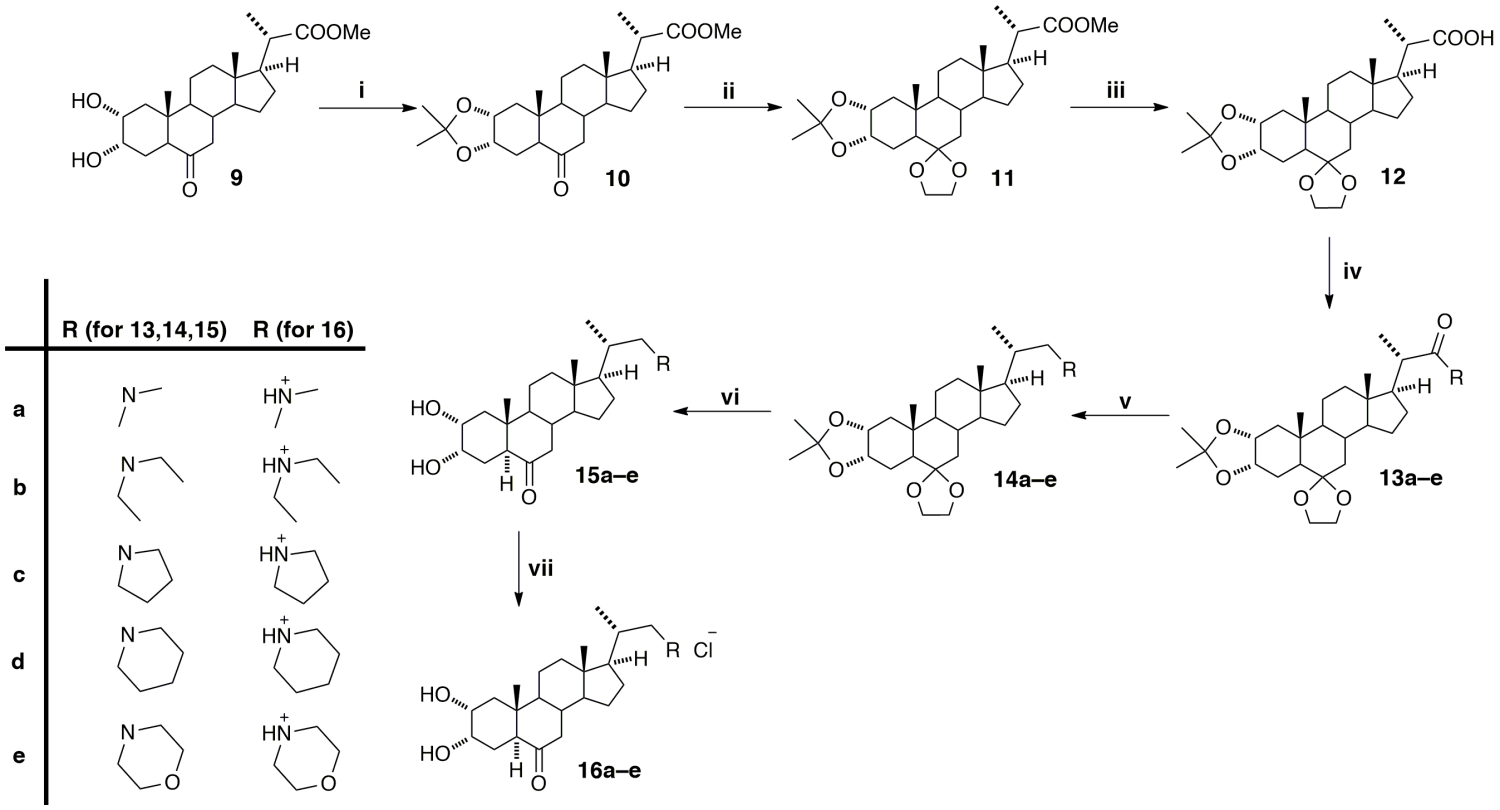
Synthesis of short side chain amines and ammonium salts: (i) TsOH, acetone, r.t.; (ii) 2,2-diMe-1,3-dioxolane, TsOH, 90 °C; (iii) KOH, *i*-PrOH, H_2_O, reflux, then HCl; (iv) amine, HOBt, EDC, DMF, 40 °C; (v) LiAlH_4_, dioxane, reflux; (vi) HCl, THF, H_2_O, 45 °C, then NaHCO_3_; (vii) HCl, MeOH, H_2_O, r.t.

**Scheme 3:**
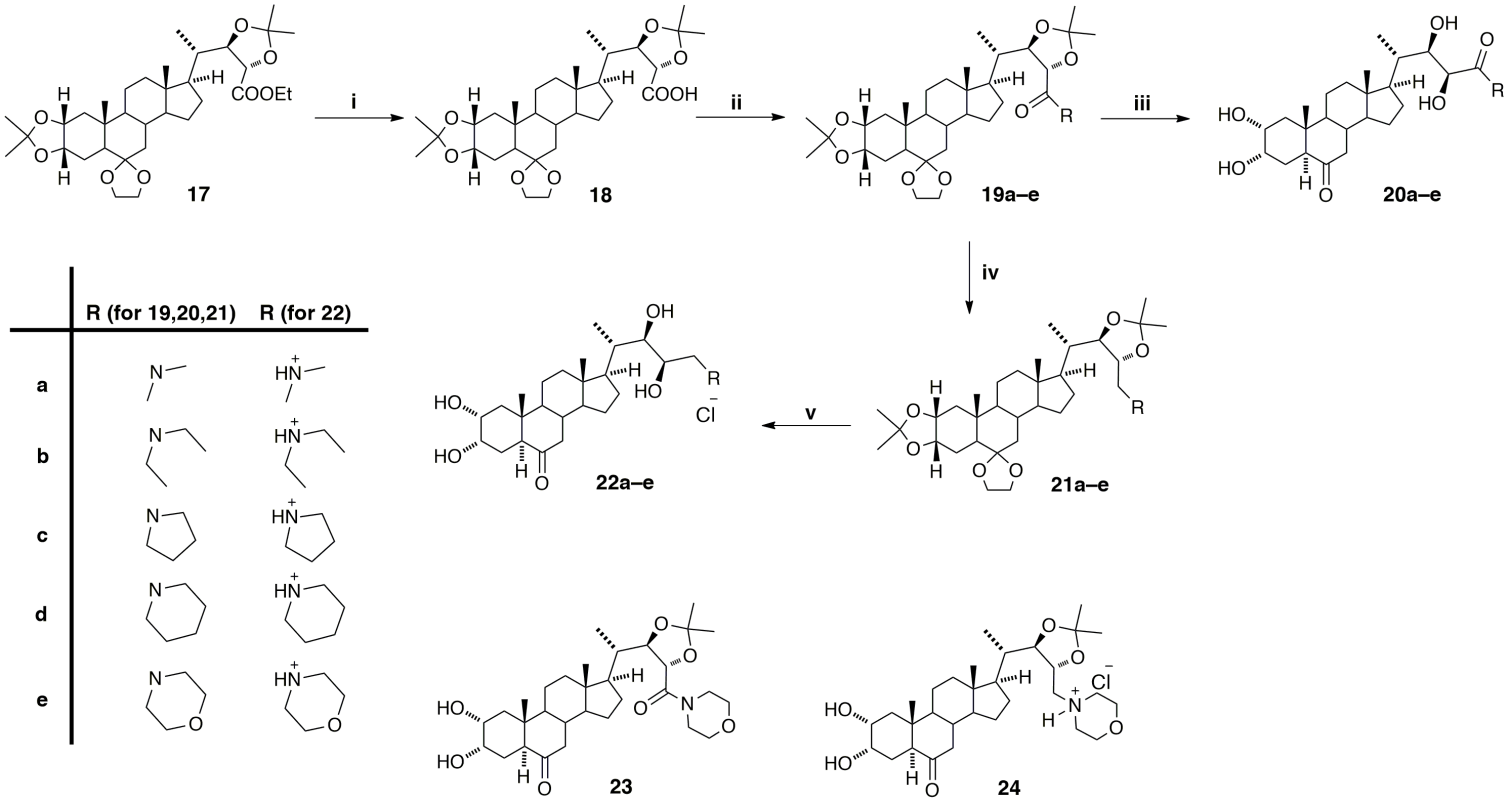
Synthesis of long side chain derivatives: (i) KOH, MeOH, H_2_O, reflux, then HCl; (ii) amine, HOBt, EDC, DMF, 40 °C; (iii) HCl, THF, H_2_O, 45 °C, then NaHCO_3_; (iv) LiAlH_4_, dioxane, reflux; (v) HCl, THF, H_2_O, 45 °C.

The synthesis of all nitrogen-containing compounds was divided into three groups—short chain amides (Scheme 1), short chain amines and ammonium chlorides (Scheme 2) and long chain derivatives (Scheme 3). The synthesis of amides with a short side chain started with the known tosylate **1** [33]. Other reaction steps correspond to standard brassinosteroid synthesis [3]. Firstly, hydroxy ester **2** is formed from tosylate, followed by oxidation with Jones reagent to oxo-ester **3**. This ketone undergoes rearrangement to olefin **4**. Hydrolysis of the ester group afforded carboxylic acid **5** that is suitable for amide formation. Five different secondary amines were used for the amidation reaction—dimethylamine, diethylamine, pyrrolidine, piperidine and morpholine. In all five cases, olefinic amides **7a**–**e** were obtained in very good yield. However, activated ester **5** can also be isolated after 4–5 h of reaction. As the last reaction, we used Upjohn dihydroxylation to prepare five dihydroxy amides, **8a**–**e**.

Due to the reduction step in the synthesis of amine analogues, the previous reaction strategy cannot be used here. It is necessary to protect both the hydroxy groups and ketone. Thus, we started with the known dihydroxy ester **9** [34]. It was firstly protected on hydroxy groups with acetone (compound **10**), followed by protection of the ketone with ethylene glycol (compound **11**). Fully protected ester was then hydrolyzed to acid **12** which is used for amide formation. Amides **13a**–**e** were then subjected to reduction with lithium aluminum hydride to obtain amines **14a**–**e**. Subsequent deprotection with basic work-up afforded dihydroxy amines **15a**–**e**, whose treatment with hydrochloric acid formed ammonium hydrochloride **16a**–**e**.

For the preparation of long side chain analogues, we used the known fully protected ester **17** [35]. Hydrolysis of the ester and condensation of acid **18** with amines afforded five fully protected amides, **19a**–**e**. Firstly, all amides were deprotected to form tetrahydroxy amides **20a**–**e**. Another portion of amides was used in lithium aluminum hydride reduction to prepare amines **21a**–**e**. After their acidic deprotection, we obtained ammonium chlorides **22a**–**e**. The deprotection on the side chain must be carried out at 45 °C. Lower temperature (below 30 °C) led only to partial deprotection on the A-ring and isolation of amide **23** and ammonium chloride **24** with the still protected side chain. 

### 2.2. Molecular Docking

Molecular docking is a useful tool to understand the pose and energetics of a protein–ligand complex. The binding site of BRI1 is located on the surface of the receptor ectodomain as a nonpolar cavity lined by nonpolar amino acids. Brassinolide fits into the cavity via its nonpolar side and displays its hydroxyl groups towards the solvent and protein partners [26]. Due to this reason, we used only tertiary amides and amines. Especially in the case of long side chain derivatives, the presence of a new hydrogen bond donor can affect their pose in the protein cavity. Molecular docking predicted similar or better binding energies than for brassinolide for compounds **8c**, **8d**, **8e**, **15d**, **20c**, **22c** and **22d** (see Table S1). This implies that derivatives with nitrogen groups in the side chain should bind within the BRI1 cavity at least as easily as brassinolide itself. Based on molecular docking, these were also candidates for showing similar binding experimentally. For poses of all nitrogen-containing derivatives used for the plant biotest, see Figures S1–S20 in the Supplementary Material.

### 2.3. Biology

**Figure 2:**
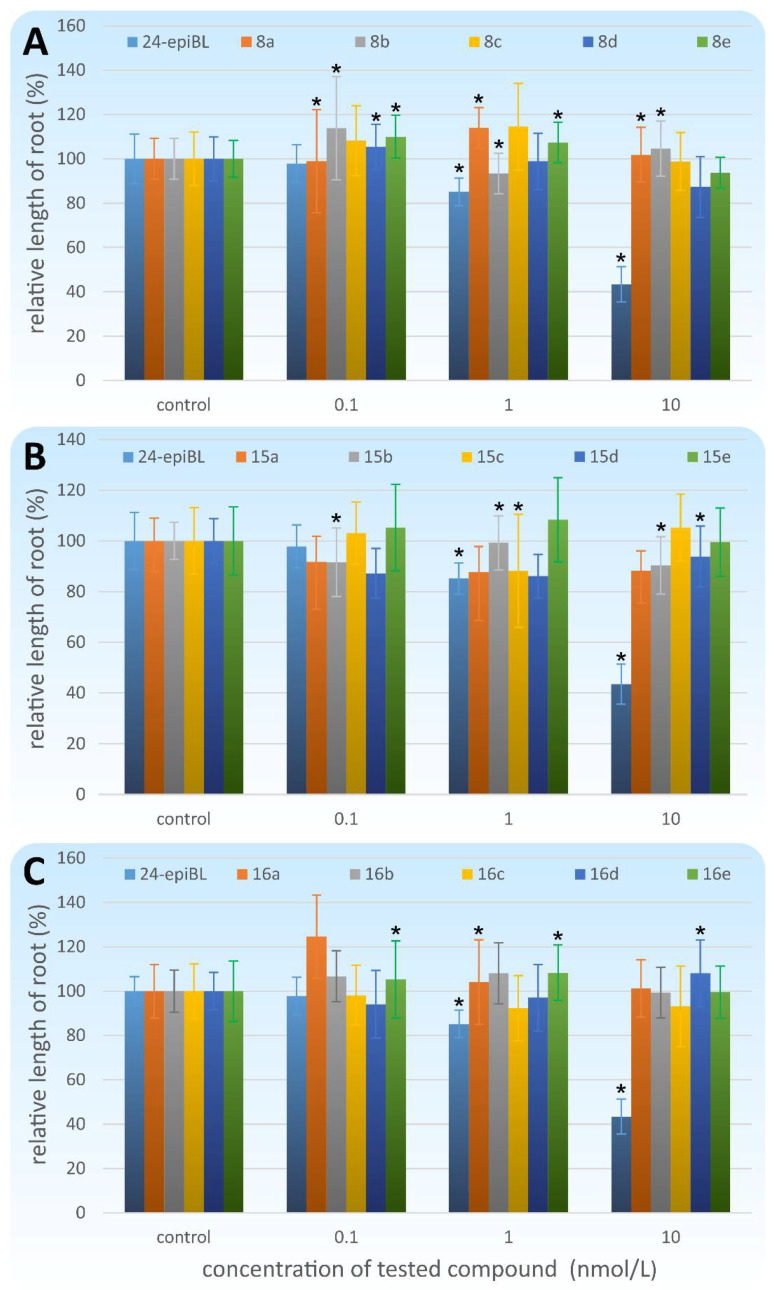
Effect of selected brassinosteroid derivatives on the inhibition of Arabidopsis root length. Seven-day-old Arabidopsis thaliana seedlings (Columbia ecotype, Col-0) were treated with DMSO as control/24epiBL/BR short side chain derivatives; (**A**)—amides, (**B**)—amines, (**C**)—ammonium hydrochlorides. For each treatment, more than 15 seedlings were analyzed in two biological repeats. Error bars represent SD. Asterisks represent significant changes (*t*-test); * represents *p*-value < 0.05, in comparison to control.

**Figure 3:**
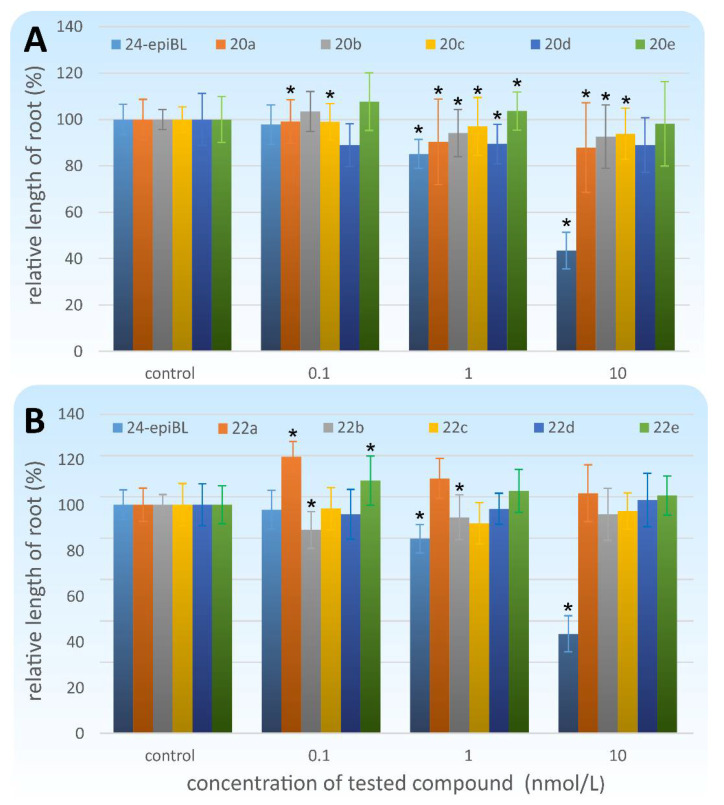
Effect of selected brassinosteroid derivatives on the inhibition of Arabidopsis root length. Seven-day-old Arabidopsis thaliana seedlings (Columbia ecotype, Col-0) were treated with DMSO as control/24epiBL/BR long side chain derivatives; (**A**)—amides, (**B**)—ammonium hydrochlorides. For each treatment, more than 15 seedlings were analyzed in two biological repeats. Error bars represent SD. Asterisks represent significant changes (*t*-test); * represents *p*-value < 0.05, in comparison to control.

Several bioassays such as the first bean internode, root growth and rice lamina inclination have been developed to evaluate the growth-promoting activity of BR derivatives [36]. In this work, the activity of new BR analogues was evaluated using the Arabidopsis root sensitivity test because of its simplicity and high sensitivity for BRs. [27,28]. The characteristic effect of exogenously applied BRs on light-grown Arabidopsis is dose-dependent—BRs in higher concentrations cause inhibition of root growth; vice versa, low concentrations are stimulating [37,38,39]. The results obtained for 24-epiBL, which was used as a positive control, and the BR derivatives are shown in Figure 2, Figure 3 and Figure S21. The most active BR (24-epiBL) inhibited Arabidopsis root growth in 1 nM and higher concentrations. Most of the newly synthesized BR derivatives were not active, and only the long side chain derivatives 20a and 20c show a weak inhibitory effect in the concentration range, which was not confirmed using the higher tested concentration of 100 nM (Figure 3 and Figure S21). Compound 22b shows the same effect only at low concentrations; on the contrary, compound 8a shows weak inhibition up to the highest tested concentration of 100 nM (see Figure S21). On the other hand, in low concentrations (0.1 and 1 nM), a slight elongation of roots was observed for derivatives with a short side chain (8a, 8b, 8e and 16e) compared to the control. A similar trend was shown for dimethylammonium hydrochloride **22a** and morpholinium hydrochloride **22e** with a long side chain. For numerical values of the average, SD and *p*-value, see Table S2.

The structure–activity relationship of brassinosteroids has been studied in detail in recent years and it has been postulated that the (2α,3α)- and (22R,23R)-vicinal diol moieties are required for optimum bioactivity and 7-oxalactone BRs have stronger biological activity than 6-oxo types, whereas B-ring non-oxidized BRs reveal no activity in biological tests [25,40,41]. Recently, several research groups prepared BR synthetic analogues with various nitrogen modifications in the side chain. Wendeborn and co-workers [25] synthesized BR derivatives where the isoxazoline ring was introduced to replace the (22,23)-vicinal diols and showed no statistically significant activity in the bean second internode elongation bioassay. As well as 25-azabrassinolide, it proved to be completely inactive at all doses studied in the rice leaf lamina inclination assay [29]. The low biological activity of BR analogues with the nitrogen-containing side chain is probably due to the lower BRI-1 receptor affinity.

The effect of the prepared BRs derivatives on the viability (in Calcein AM assays) of BJ human fibroblasts (as an example of a “normal” cell line) and human cancer cell lines of various histopathological origins, including: T-lymphoblastic leukemia CEM, breast carcinoma MCF7 and cervical carcinoma cell line HeLa, was also studied. Cells of all these lines were exposed to six 3-fold dilutions of each drug for 72 h prior to determination of cell survival. The IC_50_ (concentration leading to 50% inhibition of viability) values obtained from the Calcein AM cytotoxicity assay were calculated. 28-Homocastasterone was used as a positive control, which is the most potent natural brassinosteroid towards CEM cells (IC_50_ 13 µM [42]). All tested BR nitrogen-containing derivatives had no detectable activity, even when tested in concentrations of up to 50 µM. No BRs derivative-mediated loss of viability was observed in the BJ fibroblasts (see Table S3).

## 3. Materials and Methods 

### 3.1. Materials and Instruments

The melting points (Mp) were determined on a Stuart SMP30 instrument (Bibby Scientific Ltd., Staffordshire, UK). Elemental analyses were performed using an EA 1108 elemental analyzer (Fison Instruments, Glasgow, UK); the values (C, H, N) agreed with the calculated values within acceptable limits. The NMR spectra were taken on a JEOL JNM-ECA 500 (JEOL, Tokyo, Japan; ^1^H, 500 MHz; ^13^C, 125 MHz) spectrometer equipped with a 5 mm JEOL Royal probe. ^1^H NMR and ^13^C NMR chemical shifts (δ) were calibrated using tetramethylsilane (TMS, ^1^H δ = 0 ppm) or solvents: CDCl_3_ (^1^H δ = 7.26 ppm, ^13^C δ = 77.00 ppm), CD_3_OD (^1^H δ = 3.31 ppm, ^13^C δ = 49.00 ppm), DMSO-*d*_6_ (^1^H δ = 2.46 ppm, ^13^C δ = 40.00 ppm) or D_2_O (^1^H δ = 4.79 ppm). Chemical shifts are given in ppm (δ-scale) and coupling constants (J) are given in Hz. All values were obtained by first-order analysis. The most common abbreviations for NMR signals were used: s—singlet, d—doublet, t—triplet, q—quartet, m—multiplet, b—broad (and combinations thereof, e.g., dd—doublet of doublet, bs—broad singlet, etc.). For API HRMS analysis, the samples were dissolved in chloroform (or chloroform/methanol; 1:1; *v*/*v*, in the case of hydroxylated compounds) to a concentration 10 μg·mL^−1^. The ASAP (Atmospheric Solids Analysis Probe) was dipped into the sample solution, placed into the ion source and analyzed in full scan mode. The source of the Synapt G2-Si Mass Spectrometer (Waters, Manchester, UK) was operated in positive ionization mode (ASAP+) and, if not stated otherwise, at a source temperature of 120 °C. The Corona needle current was kept at 5 μA and the collision energy was kept at a value of 4. The probe temperature was ramped up from 50 to 600 °C in 3 min. Data were acquired from 50 to 1000 Da with 1.0 s scan time in high-resolution mode and processed using the Masslynx 4.1 software (Waters, Manchester, UK). Mass accuracy of 1 ppm or less was achieved with the described instrumentation for all compounds. Merck silica gel Kieselgel 60 (230–400 mesh) was used for column chromatography. Reagents and solvents were purchased from Sigma-Aldrich (St. Louis, MO, USA) and were not purified. 

### 3.2. Chemistry

Ethyl (*20S*)-6β-hydroxy-3α,5-cyclo-5α-pregnane-20-carboxylate (**2**)

Potassium acetate (5.4 g; 55 mmol) was added to a solution of tosylate **1** (3.0 g; 5.54 mmol) in acetone (140 mL) and water (40 mL). The reaction was stirred under reflux for 7 h. Then, excess of acetone was removed under reduced pressure and the residual suspension was dissolved in diethyl ether and extracted twice with water. Organic solvents were dried with sodium sulfate and removed under reduced pressure. The crude product was purified on silica gel (mobile phase—30% ethyl acetate in cyclohexane) to afford hydroxyl ester **2** (2.1 g; 97%) as a white solid.

Mp 59–60 °C. ^1^H NMR (CDCl_3_) *δ* 0.29 (dd, 3H, *J* = 8.1 and 4.5 Hz); 0.53 (t, 1H, *J* = 4.5 Hz, H-4β); 0.74 (s, 3H, CH_3_); 1.06 (s, 3H, CH_3_); 1.19 (d, 3H, *J* = 6.7 Hz, CH_3_); 1.25 (t, 3H, *J* = 7.0 Hz, CH_2_C**H_3_**); 1.93 (dt, 1H, *J* = 12.5 and 4.0 Hz); 2.41 (dq, 1H, *J* = 10.6 and 8.0 Hz, H-20); 3.26 (t, 1H, *J* = 2.9 Hz, H-6α); 4.07–4.16 (m, 2H, C**H_2_**CH_3_). ^13^C NMR *δ* 11.57, 12.37, 14.25, 17.10, 20.20, 22.60, 24.22, 24.26, 24.98, 27.19, 29.85, 33.17, 37.03, 38.85, 39.92, 42.61, 42.78, 42.86, 47.57, 52.97, 56.00, 59.96, 73.68, 177.00. HRMS: (API+) calculated for C_24_H_39_O_3_ ([M + H]^+^) 375.2899, found 375.2899.

Ethyl (*20S*)-6-oxo-3α,5-cyclo-5α-pregnane-20-carboxylate (**3**)

Jones reagent (10 mL) was added to a solution of hydroxy ester **2** (2 g; 5.13 mmol) in acetone (150 mL) at 0 °C. The reaction was stirred at 0 °C for an additional 20 min and then quenched with isopropanol (20 mL). Excess of acetone was removed under reduced pressure. The suspension was diluted with diethyl ether and extracted twice with water. Organic solvents were dried with sodium sulfate and removed under reduced pressure. The crude product was purified on silica gel (mobile phase—10% ethyl acetate in cyclohexane) to afford oxoester **3** (1.7 g; 85%) as a white solid.

Mp 86–87 °C. ^1^H NMR (CDCl_3_) *δ* 0.73 (t, 1H, *J* = 4.9 Hz, H-4β); 0.74 (s, 3H, CH_3_); 1.01 (s, 3H, CH_3_); 1.20 (d, 3H, *J* = 6.7 Hz, CH_3_); 1.25 (t, 3H, *J* = 7.0 Hz, CH_2_C**H_3_**); 1.89–1.95 (m, 2H); 1.99 (t, 1H, *J* = 12.6 and 3.5 Hz); 2.38–2.45 (m, 2H, H-7β, H-20); 4.07–4.16 (m, 2H, C**H_2_**CH_3_). ^13^C NMR *δ* 11.65, 12.20, 14.24, 17.08, 19.65, 22.76, 24.10, 25.85, 27.04, 33.44, 34.72, 35.34, 39.45, 42.53, 42.75, 44.67, 45.98, 46.28, 46.70, 52.72, 56.48, 60.04, 176.75, 209.47. HRMS: (API+) calculated for C_24_H_37_O_3_ ([M + H]^+^) 373.2743, found 373.2744.

Ethyl (*20S*)-6-oxo-5α-pregn-2-ene-20-carboxylate (**4**)

Lithium bromide (165 mg; 1.9 mmol) and pyridinium *p*-toluensulfonate (165 mg; 0.66 mmol) were added to a solution of oxoester **3** (1.65 g; 4.2 mmol) in dimethylacetamide (60 mL). The reaction mixture was stirred under reflux for 5 h. Dimethylacetamide was then removed under reduced pressure. The crude mixture was diluted in diethyl ether and extracted twice with water. Organic solvents were dried with sodium sulfate and removed under reduced pressure. The crude product was purified on silica gel (mobile phase—10% ethyl acetate in cyclohexane) to afford unsaturated ester **4** (1.45 g; 88%) as a white solid.

Mp 118–120 °C. ^1^H NMR (CDCl_3_) *δ* 0.70 (s, 3H, CH_3_); 0.72 (s, 3H, CH_3_); 1.20 (d, 3H, *J* = 6.7 Hz, CH_3_); 1.25 (t, 3H, *J* = 7.0 Hz, CH_2_C**H_3_**); 1.71–1.79 (m, 2H); 1.97–2.05 (m, 4H); 2.26 (m, 1H); 2.34–2.36 (m, 2H); 2.41 (dq, 1H, *J* = 10.4 and 7.0 Hz, H-20); 4.07–4.16 (m, 2H, C**H_2_**CH_3_); 5.57, 5.69 (both m, 1H, H-2 and H-3). ^13^C NMR *δ* 12.11, 13.48, 14.24, 17.07, 21.03, 21.68, 23.99, 26.92, 37.61, 39.23, 39.29, 39.99, 42.51, 42.84, 46.89, 52.73, 53.28, 53.78, 56.25, 60.04, 124.45, 124.93, 176.74, 211.81. HRMS: (API+) calculated for C_24_H_37_O_3_ ([M + H]^+^) 373.2743, found 373.2745.

(*20S*)-6-oxo-5α-pregn-2-ene-20-carboxylic acid (**5**)

Potassium hydroxide (1.0 g; 18 mmol) was added to a solution of ester **4** (1.4 g; 3.63 mmol) in ethanol (100 mL) and water (5 mL). The reaction mixture was stirred under reflux for 8 h. The excess ethanol was then removed under reduced pressure. The suspension was diluted with ethyl acetate and extracted with 5% aqueous hydrochloric acid and twice with water. Organic solvents were dried with sodium sulfate and evaporated under reduced pressure. The crude product was purified on silica gel (mobile phase—50–80% ethyl acetate in cyclohexane) to afford acid **5** (1.18 g; 91%) as a white solid. All data correspond to the data published [34].

General procedure for amide formation from acids **5**, **12** and **18**

1-Ethyl-3-(3-dimethylaminopropyl)carbodiimide (1.1 equivalents) was added to a solution of either acid **5** (100 mg; 0.28 mmol), acid **12** (200 mg; 0.43 mmol) or acid **18** (200 mg; 0.36 mmol), secondary amine (1.5 equivalents) and 1-hydroxybenzotriazole hydrate (1.1 equivalents) in dimethylformamide (10 mL). The reaction mixture was stirred at 40 °C for 12 h. Then, it was diluted with ethyl acetate and extracted twice with water. The combined organic fractions were dried with sodium sulfate and evaporated under reduced pressure. The crude product was purified on silica gel (mobile phase at each experiment). 

1H-benzo[d][1,2,3]triazol-1-yl (*20S*)-6-oxo-5α-pregn-2-ene-20-carboxylate (**6**)

Compound **6** can be obtained as a white solid intermediate (quant. yield) in amide formation according to the general procedure after 5 h at room temperature. 

Mp > 160 °C (decomp.). ^1^H NMR (CDCl_3_) *δ* 0.75 (s, 3H, CH_3_); 0.84 (s, 3H, CH_3_); 1.55 (d, 3H, *J* = 6.9 Hz, CH_3_); 1.78–1.91 (m, 2H); 2.00–2.13 (m, 4H); 2.28 (m, 1H); 2.34–2.42 (m, 2H); 2.96 (m, 1H, H-20); 5.59, 5.71 (both m, 1H, H-2 and H-3); 7.38 (dt, 1H, *J* = 8.5 and 0.9 Hz, H_Ar_); 7.44 (ddd, 1H, *J* = 8.5, 7.1 and 0.9 Hz, H_Ar_); 7.56 (ddd, 1H, *J* = 8.5, 7.1 and 0.9 Hz, H_Ar_); 8.08 (dt, 1H, *J* = 8.5 and 0.9 Hz, H_Ar_). ^13^C NMR *δ* 12.23, 13.52, 17.17, 21.05, 21.68, 24.12, 27.37, 37.58, 39.21, 39.29, 39.96, 40.38, 43.31, 46.79, 52.37, 53.20, 53.80, 56.15, 108.10, 120.58, 124.37, 124.78, 124.97, 128.50, 128.71, 143.48, 172.22, 211.48. HRMS: (API+) calculated for C_28_H_36_N_3_O_3_ ([M + H]^+^) 462.2757, found 462.2758.

*N*,*N*-dimethyl-(*20S*)-6-oxo-5α-pregn-2-ene-20-carboxamide (**7a**)

The general procedure for amide formation with acid **5** and dimethylamine and chromatography on silica gel (isopropanol/cyclohexane—1/9) yielded 79 mg (76%) of the title compound **7a** as a colorless oil:

^1^H NMR (CDCl_3_) *δ* 0.73 (s, 3H, CH_3_); 0.74 (s, 3H, CH_3_); 1.15 (d, 3H, *J* = 6.7 Hz, CH_3_); 1.75 (dtd, 1H, *J* = 12.4, 10.8 and 4.3 Hz); 1.81–1.89 (m, 2H); 1.97–2.05 (m, 4H); 2.27 (m, 1H); 2.33–2.39 (m, 2H); 2.96 (m, 1H, H-20); 2.76 (dq, 1H, *J* = 9.8 and 6.7 Hz, H-20); 3.01 (bs, 6H, 2×NCH3); 5.58, 5.69 (both m, 1H, H-2 and H-3). ^13^C NMR *δ* 12.35, 13.48, 17.04, 21.05, 21.67, 23.97, 27.35, 37.66 (2×C), 37.68, 39.25, 39.29 (2×C), 40.05, 42.62, 46.92, 52.88, 53.24, 53.76, 56.12, 124.47, 124.90, 176.56, 211.97. HRMS: (API+) calculated for C_24_H_38_NO_2_ ([M + H]^+^) 372.2903, found 372.2903.

*N*,*N*-diethyl-(*20S*)-6-oxo-5α-pregn-2-ene-20-carboxamide (**7b**)

The general procedure for amide formation with acid **5** and diethylamine and chromatography on silica gel (isopropanol/cyclohexane—1/19) yielded 87 mg (78%) of the title compound **7b** as a colorless oil:

^1^H NMR (CDCl_3_) *δ* 0.72 (s, 3H, CH_3_); 0.73 (s, 3H, CH_3_); 1.10 (t, 3H, *J* = 7.1 Hz, CH_3_); 1.16 (d, 3H, *J* = 6.7 Hz, CH_3_); 1.19 (t, 3H, *J* = 7.1 Hz, CH_3_); 1.74 (m, 1H); 1.80–1.90 (m, 2H); 1.97–2.05 (m, 4H); 2.26 (m, 1H); 2.34 (dd, 1H, *J* = 13.3 and 4.1 Hz); 2.37 (dd, 1H, *J* = 11.1 and 4.9 Hz); 2.64 (dq, 1H, *J* = 9.7 and 6.6 Hz, H-20); 3.22–3.33 (m, 2H, 2×NCH); 3.37–3.51 (m, 2H, 2×NCH); 5.58, 5.69 (both m, 1H, H-2 and H-3). ^13^C NMR *δ* 12.43, 13.01, 13.50, 14.94, 17.69, 21.08, 21.68, 23.96, 27.10, 37.53, 37.72, 39.28, 39.30, 40.07, 40.48, 42.11, 42.61, 46.92, 53.04, 53.26, 53.77, 56.12, 124.49, 124.91, 175.71, 212.02. HRMS: (API+) calculated for C_26_H_42_NO_2_ ([M + H]^+^) 400.3216, found 400.3215.

1-((*20S*)-6-oxo-5α-pregn-2-ene-20-carbonyl)pyrrolidine (**7c**)

The general procedure for amide formation with acid **5** and pyrrolidine and chromatography on silica gel (isopropanol/cyclohexane—1/19) yielded 91 mg (82%) of the title compound **7c** as a colorless oil:

^1^H NMR (CDCl_3_) *δ* 0.71 (s, 6H, 2×CH3); 1.16 (d, 3H, *J* = 6.7 Hz, CH3); 1.74 (m, 1H); 1.81–1.89 (m, 4H); 1.92–2.05 (m, 6H); 2.25 (m, 1H); 2.34 (dd, 1H, *J* = 13.1 and 4.3 Hz); 2.38 (dd, 1H, *J* = 11.0 and 4.9 Hz); 2.55 (dq, 1H, *J* = 9.8 and 6.7 Hz, H-20); 3.43–3.55 (m, 4H, 4×NCH); 5.57, 5.69 (both m, 1H, H-2 and H-3). ^13^C NMR *δ* 12.38, 13.48, 16.92, 21.05, 21.67, 23.97, 24.35, 26.09, 27.25, 37.71, 39.20, 39.28, 40.06, 40.29, 42.58, 45.56, 46.79, 46.92, 52.71, 53.24, 53.75, 56.10, 124.48, 124.89, 175.05, 212.01. HRMS: (API+) calculated for C_26_H_40_NO_2_ ([M + H]^+^) 398.3059, found 398.3062.

1-((*20S*)-6-oxo-5α-pregn-2-ene-20-carbonyl)piperidine (**7d**)

The general procedure for amide formation with acid **5** and piperidine and chromatography on silica gel (isopropanol/cyclohexane—1/19) yielded 99 mg (86%) of the title compound **7d** as a colorless oil:

^1^H NMR (CDCl_3_) *δ* 0.720 (s, 3H, CH_3_); 0.724 (s, 3H, CH_3_); 1.15 (d, 3H, *J* = 6.7 Hz, CH_3_); 1.75 (dtd, 1H, *J* = 12.5, 10.7 and 4.3 Hz); 1.82–1.91 (m, 2H); 1.98–2.05 (m, 4H); 2.26 (m, 1H); 2.35 (dd, 1H, *J* = 13.1 and 4.3 Hz); 2.37 (dd, 1H, *J* = 11.0 and 4.9 Hz); 2.76 (dq, 1H, *J* = 9.7 and 6.7 Hz, H-20); 3.46–3.68 (m, 4H, 4×NCH); 5.58, 5.69 (both m, 1H, H-2 and H-3). 13C NMR δ 12.33, 13.50, 17.43, 21.06, 21.68, 24.01, 24.71, 25.88 (vbs), 26.89 (vbs, 2×C), 27.62, 37.70, 39.27, 39.30, 40.07, 42.67, 42.92 (vbs), 46.81 (vbs), 46.93, 52.74, 53.26, 53.77, 56.17, 124.49, 124.91, 174.79, 212.02. HRMS: (API+) calculated for C_27_H_42_NO_2_ ([M + H]^+^) 412.3216, found 412.3216.

4-((*20S*)-6-oxo-5α-pregn-2-ene-20-carbonyl)morpholine (**7e**)

The general procedure for amide formation with acid **5** and morpholine and chromatography on silica gel (isopropanol/cyclohexane—1/19) yielded 97 mg (84%) of the title compound **7e** as a colorless oil:

^1^H NMR (CDCl_3_) *δ* 0.718 (s, 3H, CH_3_); 0.720 (s, 3H, CH_3_); 1.16 (d, 3H, *J* = 6.7 Hz, CH_3_); 1.75 (dtd, 1H, *J* = 12.4, 10.6 and 4.2 Hz); 1.82–1.91 (m, 2H); 1.98–2.05 (m, 4H); 2.26 (m, 1H); 2.35 (dd, 1H, *J* = 13.1 and 4.3 Hz); 2.38 (dd, 1H, *J* = 11.0 and 4.6 Hz); 2.70 (dq, 1H, *J* = 10.0 and 6.8 Hz, H-20); 3.54–3.69 (m, 8H, 8×Hmorp_holine_); 5.57, 5.69 (both m, 1H, H-2 and H-3). ^13^C NMR *δ* 12.44, 13.61, 17.47, 21.16, 21.79, 24.11, 27.74, 37.41 (bs), 37.77, 39.37, 39.41, 40.16, 42.15 (bs), 42.81, 46.40 (bs), 47.01, 52.80, 53.34, 53.89, 56.24, 66.97 (bs), 67.18 (bs), 124.57, 125.03, 175.26, 212.01. HRMS: (API+) calculated for C_26_H_40_NO_3_ ([M + H]^+^) 414.3008, found 414.3012.

General procedure for Upjohn dihydroxylation

A solution (50 mg/mL) of osmium tetroxide in *t*-butanol (0.1 mL; 0.02 mmol) was added to a solution of unsaturated amide (~0.15 mmol) and *N*-methylmorpholine-*N*-oxide (88 mg; 0.75 mmol) in acetone (4 mL), tetrahydrofuran (4 mL) and water (0.5 mL). The reaction mixture was stirred for 12 h at room temperature. A saturated aqueous solution of sodium sulfite (2 mL) was then added, and the mixture was stirred for an additional 30 min. The reaction was diluted with ethyl acetate and extracted twice with water. The organic phase was dried with sodium sulfate and solvents were evaporated under reduced pressure. The crude product was purified on silica gel (mobile phase at each experiment). 

*N*,*N*-dimethyl-(*20S*)-2α,3α-dihydroxy-6-oxo-5α-pregnane-20-carboxamide (**8a**)

The general procedure for dihydroxylation with amide **7a** and chromatography on silica gel (isopropanol/ethyl acetate—1/19) yielded 52 mg (85%) of the title compound **8a** as a white solid:

Mp > 230 °C (decomp.). ^1^H NMR (CDCl_3_) *δ* 0.68 (s, 3H, CH_3_); 0.73 (s, 3H, CH_3_); 1.10 (d, 3H, *J* = 6.7 Hz, CH_3_); 1.86 (dt, 1H, *J* = 15.2 and 3.4 Hz); 1.94–2.02 (m, 2H); 2.24 (dd, 1H, *J* = 13.1 and 4.6 Hz); 2.67 (dd, 1H, *J* = 12.5 and 3.1 Hz); 2.73 (dq, 1H, *J* = 9.7 and 6.8 Hz, H-20); 2.91 (s, 3H, NCH_3_); 3.05 (s, 3H, NCH_3_); 3.67 (m, 1H, Σ *J* = 19.6 Hz, H-2β); 3.98 (m, 1H, H-3β). ^13^C NMR *δ* 12.36, 13.43, 16.87, 21.06, 23.89, 26.21, 27.20, 35.66, 37.61 (2×C), 37.62, 39.13, 39.93, 42.56, 42.71, 46.59, 50.70, 52.78, 53.54, 55.98, 68.01, 68.06, 176.98, 212.76. HRMS: (API+) calculated for C_24_H_40_NO_4_ ([M + H]^+^) 406.2957, found 406.2957. Anal. Calcd. for C_24_H_39_NO_4_: C, 71.07; H, 9.69; N, 3.45. Found: C, 71.02; H, 9.73; N, 3.42%.

*N*,*N*-diethyl-(*20S*)-2α,3α-dihydroxy-6-oxo-5α-pregnane-20-carboxamide (**8b**)

The general procedure for dihydroxylation with amide **7b** and chromatography on silica gel (isopropanol/ethyl acetate—1/19) yielded 57 mg (88%) of the title compound **8b** as a white solid:

Mp > 200 °C (decomp.). ^1^H NMR (CDCl_3_) *δ* 0.72 (s, 3H, CH_3_); 0.78 (s, 3H, CH_3_); 1.10 (bt, 3H, *J* = 6.7 Hz, CH_3_); 1.16 (d, 3H, *J* = 6.7 Hz, CH_3_); 1.20 (bt, 3H, *J* = 6.7 Hz, CH_3_); 1.93 (dt, 1H, *J* = 15.0 and 3.4 Hz); 1.98–2.05 (m, 2H); 2.30 (dd, 1H, *J* = 13.1 and 4.6 Hz); 2.64 (dq, 1H, *J* = 9.9 and 6.8 Hz, H-20); 2.73 (dd, 1H, *J* = 12.7 and 2.9 Hz); 3.24–3.32 (m, 2H, NCH_2_); 3.39–3.47 (m, 2H, NCH_2_); 3.75 (m, 1H, Σ *J* = 19.6 Hz, H-2β); 4.08 (m, 1H, H-3β). ^13^C NMR *δ* 12.52, 12.93, 13.57, 14.86, 17.59, 21.19, 23.97, 26.31, 27.05, 37.59, 37.67, 39.34, 40.31, 40.82 (bs), 42.33 (bs), 42.67, 42.80, 46.76, 50.85, 52.96, 53.85, 56.21, 68.43, 68.50, 176.14, 212.06. HRMS: (API+) calculated for C_26_H_44_NO_4_ ([M + H]^+^) 434.3270, found 434.3270. Anal. Calcd. for C_26_H_43_NO_4_: C, 72.02; H, 10.00; N, 3.23. Found: C, 71.95; H, 10.06; N, 3.20%.

1-((*20S*)-2α,3α-dihydroxy-6-oxo-5α-pregnane-20-carbonyl)pyrrolidine (**8c**)

The general procedure for dihydroxylation with amide **7c** and chromatography on silica gel (isopropanol/ethyl acetate—1/19) yielded 55 mg (85%) of the title compound **8c** as a white solid:

Mp > 180 °C (decomp.). ^1^H NMR (CDCl_3_) *δ* 0.71 (s, 3H, CH_3_); 0.77 (s, 3H, CH_3_); 1.16 (d, 3H, *J* = 6.7 Hz, CH_3_); 1.92 (dt, 1H, *J* = 15.1 and 3.3 Hz); 1.93–2.03 (m, 4H); 2.29 (dd, 1H, *J* = 13.1 and 4.6 Hz); 2.55 (dq, 1H, *J* = 9.8 and 6.5 Hz, H-20); 2.73 (dd, 1H, *J* = 12.5 and 2.8 Hz); 3.43–3.55 (m, 4H, 2×NCH_2_); 3.75 (m, 1H, Σ *J* = 19.6 Hz, H-2β); 4.07 (m, 1H, H-3β). ^13^C NMR *δ* 12.46, 13.54, 16.81, 21.17, 23.98, 24.29, 26.02, 26.31, 27.20, 37.67, 39.30, 40.27, 40.33, 42.66, 42.78, 45.84, 46.74, 47.02, 50.84, 52.73, 53.84, 56.22, 68.41, 68.48, 175.45, 212.12. HRMS: (API+) calculated for C_26_H_42_NO_4_ ([M + H]^+^) 432.3114, found 432.3116. Anal. Calcd. for C_26_H_41_NO_4_: C, 72.35; H, 9.57; N, 3.25. Found: C, 72.29; H, 9.61; N, 3.22%.

1-((20S)-2α,3α-dihydroxy-6-oxo-5α-pregnane-20-carbonyl)piperidine (**8d**)

The general procedure for dihydroxylation with amide **7d** and chromatography on silica gel (isopropanol/ethyl acetate—1/19) yielded 58 mg (87%) of the title compound 8d as a white solid:

Mp > 230 °C (decomp.). ^1^H NMR (CDCl_3_) *δ* 0.71 (s, 3H, CH_3_); 0.77 (s, 3H, CH_3_); 1.14 (d, 3H, *J* = 6.7 Hz, CH_3_); 1.93 (dt, 1H, *J* = 15.1 and 3.5 Hz); 1.98–2.04 (m 2H); 2.29 (dd, 1H, *J* = 13.1 and 4.6 Hz); 2.73 (dd, 1H, *J* = 12.7 and 2.9 Hz); 2.76 (dq, 1H, *J* = 9.8 and 6.7 Hz, H-20); 3.48–3.60 (m, 4H, 2×NCH2); 3.74 (m, 1H, Σ *J* = 19.6 Hz, H-2β); 4.07 (m, 1H, H-3β). 13C NMR δ 12.41, 13.56, 17.37, 21.17, 24.01, 24.61, 26.29, ~26.76 (vbm 3×C), 37.64, 39.35, 40.30, 42.67, 42.83, 43.02 (vbs), 46.76, 46.88 (vbs), 50.84, 52.77, 53.26, 53.85, 56.28, 68.41, 68.44, 175.03, 212.08. HRMS: (API+) calculated for C_27_H_44_NO_4_ ([M + H]^+^) 446.3270, found 446.3271. Anal. Calcd. for C_27_H_43_NO_4_: C, 72.77; H, 9.73; N, 3.14. Found: C, 72.75; H, 9.78; N, 3.10%. 

4-((*20S*)-2α,3α-dihydroxy-6-oxo-5α-pregnane-20-carbonyl)morpholine (**8e**)

The general procedure for dihydroxylation with amide **7e** and chromatography on silica gel (isopropanol/ethyl acetate—1/9) yielded 56 mg (84%) of the title compound **8e** as a white solid:

Mp > 270 °C (decomp.). ^1^H NMR (CDCl_3_+MeOD) *δ* 0.68 (s, 3H, CH_3_); 0.73 (s, 3H, CH_3_); 1.13 (d, 3H, *J* = 6.7 Hz, CH_3_); 1.87 (dt, 1H, *J* = 15.3 and 3.5 Hz); 1.95–2.03 (m 2H); 2.26 (dd, 1H, *J* = 13.1 and 4.6 Hz); 2.65–2.71 (m, 2H); 3.54–3.68 (m, 8H, 8×Hmo_rpholine_); 3.69 (m, 1H, H-2β); 3.99 (m, 1H, H-3β). ^13^C NMR *δ* 12.34, 13.46, 17.22, 21.06, 23.92, 26.22, 27.51, 37.23 (bs), 37.59, 39.13, 39.97, 42.05 (bs), 42.54, 42.79, 46.26 (bs), 46.58, 50.69, 52.58, 53.54, 56.00, 66.77 (bs), 66.97 (bs), 68.00, 68.07, 175.38, 212.52. HRMS: (API+) calculated for C_26_H_42_NO_5_ ([M + H]^+^) 448.3063, found 448.3063. Anal. Calcd. for C_26_H_41_NO_5_: C, 69.77; H, 9.23; N, 3.13. Found: C, 69.72; H, 9.25; N, 3.09%.

Methyl (*20S*)-2α,3α-(1-methylethylidene)dioxy-6,6-ethylenedioxy-5α-pregnane-20-carboxylate (**11**)

p-Toluensulfonic acid (50 mg; 0.29 mmol) was added to a solution of ester **9** (1.0 g; 2.55 mmol) in dry acetone (100 mL). The reaction mixture was stirred at room temperature for 5 h. The excess acetone was removed under reduced pressure and the suspension was diluted in ethyl acetate and extracted with a saturated solution of sodium bicarbonate and twice with water. The organic phase was dried over sodium sulfate and solvents were evaporated under reduced pressure. Partially protected ester **10** was dissolved in 2,2-dimethyl-1,3-dioxolan (20 mL) and p-toluensulfonic acid (10 mg; 0.06 mmol) was added. The reaction mixture was stirred at 90 °C for 10 h. The mixture was diluted with ethyl acetate and extracted with a saturated solution of sodium bicarbonate and twice with water. The organic phase was dried over sodium sulfate and solvents were evaporated under reduced pressure. The crude product was purified on silica gel (mobile phase—30% ethyl acetate in cyclohexane) to afford protected methyl ester **11** (888 mg; 74% after 2 steps) as a colorless oil.

^1^H NMR (DMSO-*d*_6_) *δ* 0.62 (s, 3H, CH_3_); 0.76 (s, 3H, CH_3_); 1.10 (d, 3H, *J* = 6.7 Hz, CH_3_); 1.21 (s, 3H, CH_3_); 1.35 (s, 3H, CH_3_); 1.62–1.71 (m, 3H); 1.79 (dd, 1H, *J* = 12.5 and 7.0 Hz); 1.86 (m, 1H); 1.96 (m, 1H); 2.32 (dq, 1H, *J* = 10.3 and 6.9 Hz, H-20); 3.55 (s, 3H, CH_3_); 3.67 (m, 1H, CH_2_O); 3.80–3.91 (m, 3H, CH_2_O); 4.01 (m, 1H, H-2β); 4.18 (m, 1H, H-3β). ^13^C NMR *δ* 11.83, 13.18, 16.91, 20.34, 21.46, 23.87, 26.65, 26.74, 28.65, 32.53, 37.61, 38.82, 40.64, 41.83, 42.09, 42.44, 45.12, 51.21, 52.16, 52.65, 54.81, 63.72, 65.18, 72.04, 72.27, 106.80, 108.86, 176.27. HRMS: (API+) calculated for C_28_H_45_O_6_ ([M + H]^+^) 477.3216, found 477.3220.

(*20S*)-2α,3α-(1-methylethylidene)dioxy-6,6-ethylenedioxy-5α-pregnane-20-carboxylic acid (**12**)

Potassium hydroxide (500 mg; 8.9 mmol) was added to a solution of methyl ester 11 (850 mg; 1.79 mmol) in isopropanol (80 mL) and water (10 mL). The reaction mixture was stirred under reflux for 4 h. After cooling to room temperature, the excess of isopropanol was removed under reduced pressure and the residue was diluted in ethyl acetate and extracted with 5% aqueous solution of citric acid and twice with water. The organic phase was dried over sodium sulfate and solvents were evaporated under reduced pressure to obtain acid 12 (795 mg; 96%) in adequate purity for other reactions. 

^1^H NMR (DMSO-*d*_6_) *δ* 0.62 (s, 3H, CH_3_); 0.77 (s, 3H, CH_3_); 1.09 (d, 3H, *J* = 6.9 Hz, CH_3_); 1.22 (s, 3H, CH_3_); 1.36 (s, 3H, CH_3_); 1.62–1.74 (m, 3H); 1.79 (dd, 1H, *J* = 12.7 and 6.9 Hz); 1.87 (m, 1H); 1.97 (m, 1H); 2.20 (dq, 1H, *J* = 10.4 and 6.8 Hz, H-20); 3.67 (m, 1H, CH_2_O); 3.80–3.91 (m, 3H, CH_2_O); 4.01 (m, 1H, H-2β); 4.19 (m, 1H, H-3β); 11.94 (bs, 1H, COOH). ^13^C NMR *δ* 11.84, 13.15, 17.02, 20.32, 21.42, 23.90, 26.60, 26.95, 28.60, 32.48, 37.57, 38.92, 40.60, 41.95, 42.10, 42.40, 45.08, 52.15, 52.42, 54.87, 63.67, 65.13, 72.00, 72.22, 106.74, 108.82, 177.40. HRMS: (API+) calculated for C_27_H_43_O_6_ ([M + H]^+^) 463.3060, found 463.3059.

*N*,*N*-dimethyl-(*20S*)-2α,3α-(1-methylethylidene)dioxy-6,6-ethylenedioxy-5α-pregnane-20-carboxamide (**13a**)

The general procedure for amide formation with acid **12** and dimethylamine and chromatography on silica gel (40–60% ethyl acetate in cyclohexane) yielded 180 mg (85%) of the title compound **13a** as a colorless oil:

^1^H NMR (DMSO-*d*_6_) *δ* 0.67 (s, 3H, CH_3_); 0.77 (s, 3H, CH_3_); 0.98 (d, 3H, *J* = 6.7 Hz, CH_3_); 1.22 (s, 3H, CH_3_); 1.36 (s, 3H, CH_3_); 1.62–1.74 (m, 3H); 1.79 (dd, 1H, *J* = 12.7 and 6.9 Hz); 1.88 (m, 1H); 1.97 (m, 1H); 2.72 (m, 1H, H-20); 2.78 (s, 3H, CH_3_); 3.00 (s, 3H, CH_3_); 3.67 (m, 1H, CH_2_O); 3.80–3.90 (m, 3H, CH_2_O); 4.02 (m, 1H, H-2β); 4.19 (m, 1H, H-3β). ^13^C NMR *δ* 12.12, 13.16, 17.02, 20.34, 21.42, 23.87, 26.36, 26.62, 27.00, 28.61, 32.47, 35.02, 37.04, 37.57, 39.08, 40.64, 41.76, 42.40, 45.07, 52.18, 52.91, 54.90, 63.65, 65.15, 72.02, 72.23, 106.75, 108.86, 175.35. HRMS: (API+) calculated for C_29_H_48_NO_5_ ([M + H]^+^) 490.3532, found 490.3534.

*N*,*N*-diethyl-(*20S*)-2α,3α-(1-methylethylidene)dioxy-6,6-ethylenedioxy-5α-pregnane-20-carboxamide (**13b**)

The general procedure for amide formation with acid **12** and diethylamine and chromatography on silica gel (40–60% ethyl acetate in cyclohexane) yielded 186 mg (83%) of the title compound **13b** as a colorless oil:

^1^H NMR (DMSO-*d*_6_) *δ* 0.67 (s, 3H, CH_3_); 0.77 (s, 3H, CH_3_); 0.97 (t, 3H, *J* = 7.0 Hz, CH_3_); 1.00 (d, 3H, *J* = 6.7 Hz, CH_3_); 1.10 (t, 3H, *J* = 7.0 Hz, CH_3_); 1.22 (s, 3H, CH_3_); 1.36 (s, 3H, CH_3_); 1.62–1.74 (m, 3H); 1.80 (dd, 1H, *J* = 12.7 and 6.9 Hz); 1.89 (m, 1H); 1.97 (m, 1H); 2.58 (m, 1H, H-20); 3.13 (m, 1H, NCH); 3.24–3.40 (m, 3H, 3×NCH); 3.67 (m, 1H, CH_2_O); 3.80–3.90 (m, 3H, CH_2_O); 4.02 (m, 1H, H-2β); 4.19 (m, 1H, H-3β). ^13^C NMR *δ* 12.14, 12.97, 13.17, 14.94, 17.65, 20.35, 21.42, 23.78, 26.35, 26.61, 26.76, 28.60, 32.47, 36.70, 37.56, 39.09, 40.65, 41.52, 41.75, 42.39, 45.06, 52.18, 53.02, 54.87, 63.65, 65.15, 72.01, 72.22, 106.74, 108.86, 174.50. HRMS: (API+) calculated for C_31_H_52_NO_5_ ([M + H]^+^) 518.3845, found 518.3849.

1-((*20S*)-2α,3α-(1-methylethylidene)dioxy-6,6-ethylenedioxy-5α-pregnane-20-carbonyl)pyrrolidine (**13c**)

The general procedure for amide formation with acid **12** and pyrrolidine and chromatography on silica gel (40–60% ethyl acetate in cyclohexane) yielded 196 mg (88%) of the title compound **13c** as a colorless oil:

^1^H NMR (DMSO-*d*_6_) *δ* 0.66 (s, 3H, CH_3_); 0.77 (s, 3H, CH_3_); 1.00 (d, 3H, *J* = 6.7 Hz, CH_3_); 1.22 (s, 3H, CH_3_); 1.36 (s, 3H, CH_3_); 1.73–1.81 (m, 3H); 1.82–1.89 (m, 3H); 1.97 (m, 1H); 2.51 (m, 1H, H-20); 3.21–3.24 (m, 2H, 2×NCH); 3.40–3.46 (m, 2H, 2×NCH); 3.67 (m, 1H, CH_2_O); 3.80–3.90 (m, 3H, CH_2_O); 4.02 (m, 1H, H-2β); 4.19 (m, 1H, H-3β). ^13^C NMR *δ* 12.18, 13.20, 16.93, 20.38, 21.46, 23.89, 23.96 (2×C), 25.73, 26.65, 26.92, 28.65, 32.53, 37.61, 39.09, 40.68, 41.76, 42.43, 45.11, 45.25, 46.22, 52.22, 52.81, 54.91, 63.69, 65.19, 72.05, 72.27, 106.80, 108.89, 173.81. HRMS: (API+) calculated for C_31_H_50_NO_5_ ([M + H]^+^) 516.3689, found 516.3690. 

1-((*20S*)-2α,3α-(1-methylethylidene)dioxy-6,6-ethylenedioxy-5α-pregnane-20-carbonyl)piperidine (**13d**)

The general procedure for amide formation with acid **12** and piperidine and chromatography on silica gel (40–60% ethyl acetate in cyclohexane) yielded 192 mg (84%) of the title compound **13d** as a colorless oil:

^1^H NMR (DMSO-*d*_6_) *δ* 0.66 (s, 3H, CH_3_); 0.77 (s, 3H, CH_3_); 0.99 (d, 3H, *J* = 6.7 Hz, CH_3_); 1.22 (s, 3H, CH_3_); 1.36 (s, 3H, CH_3_); 1.64–1.74 (m, 3H); 1.79 (dd, 1H, *J* = 12.5 and 6.7 Hz); 1.89 (m, 1H); 1.97 (m, 1H); 2.72 (m, 1H, H-20); 3.39–3.50 (m, 4H, 4×NCH); 3.67 (m, 1H, CH_2_O); 3.80–3.90 (m, 3H, CH_2_O); 4.02 (m, 1H, H-2β); 4.19 (m, 1H, H-3β). ^13^C NMR *δ* 12.09, 13.17, 17.40, 20.34, 21.44, 23.89, 24.24, 25.55, 26.56, 26.63, 27.31, 28.62, 32.47, 37.58, 39.10, 39.27, 40.67, 41.81, 42.07, 42.40, 45.09, 46.11, 52.19, 52.75, 54.94, 63.67, 65.17, 72.03, 72.24, 106.76, 108.87, 173.64. HRMS: (API+) calculated for C_32_H_51_NO_5_ ([M + H]^+^) 530.3845, found 530.3846.

4-((*20S*)-2α,3α-(1-methylethylidene)dioxy-6,6-ethylenedioxy-5α-pregnane-20-carbonyl)morpholine (**13e**)

The general procedure for amide formation with acid **12** and morpholine and chromatography on silica gel (40–60% ethyl acetate in cyclohexane) yielded 193 mg (84%) of the title compound **13b** as a colorless oil:

^1^H NMR (DMSO-*d*_6_, 45 °C) *δ* 0.68 (s, 3H, CH_3_); 0.78 (s, 3H, CH_3_); 1.02 (d, 3H, *J* = 6.7 Hz, CH_3_); 1.23 (s, 3H, CH_3_); 1.37 (s, 3H, CH_3_); 1.61–1.75 (m, 5H); 1.80 (dd, 1H, *J* = 12.5 and 7.0 Hz); 1.89 (m, 1H); 1.98 (m, 1H); 2.73 (m, 1H, H-20); 3.44–3.56 (m, 8H, 8×Hmo_rpholine_); 3.68 (m, 1H, CH_2_O); 3.81–3.90 (m, 3H, CH_2_O); 4.02 (m, 1H, H-2β); 4.19 (m, 1H, H-3β). ^13^C NMR *δ* 12.10, 13.18, 17.29, 20.35, 21.45, 23.92, 26.64, 27.21, 28.64 (2×C), 32.49, 37.60, 39.10, 40.68, 41.59, 41.88, 42.42, 45.11, 45.76, 52.20, 52.71, 54.92, 63.69, 65.19, 66.32, 66.48, 72.04, 72.26, 106.79, 108.88, 174.22. HRMS: (API+) calculated for C_31_H_50_NO_6_ ([M + H]^+^) 532.3638, found 532.3639.

General procedure for reduction of amides with lithium aluminum hydride

An amount of 1M solution of lithium aluminum hydride in diethyl ether (1.0 mL; 1.0 mmol) was added dropwise to a solution of amide (150 mg) in dry dioxane (10 mL). The reaction mixture was stirred under reflux for 3 h. After cooling, a saturated aqueous solution of sodium potassium tartrate (1 mL) was added, and the mixture was stirred for an additional 10 min. Then, it was diluted with ethyl acetate and extracted twice with water. The organic phase was dried with sodium sulfate and solvents were evaporated under reduced pressure. The crude product was purified on silica gel (mobile phase at each experiment). 

*N*,*N*-dimethyl-((*20S*)-2α,3α-(1-methylethylidene)dioxy-6,6-ethylenedioxy-5α-pregnane-20-methyl)amine (**14a**)

The general procedure for amide reduction with amide **13a** (150 mg; 0.31 mmol) and chromatography on silica gel (2–4% methanol in ammonia chloroform) yielded 106 mg (73%) of the title compound **14a** as a colorless oil:

^1^H NMR (DMSO-*d*_6_, 55 °C) *δ* 0.66 (s, 3H, CH_3_); 0.79 (s, 3H, CH_3_); 0.96 (d, 3H, *J* = 6.5 Hz, CH_3_); 1.23 (s, 3H, CH_3_); 1.37 (s, 3H, CH_3_); 1.64–1.75 (m, 3H); 1.76 (m, 1H); 1.81 (dd, 1H, *J* = 12.5 and 7.0 Hz); 1.94–2.01 (m, 2H); 2.19 (bs, 6H, 2×CH_3_), 3.69 (m, 1H, CH_2_O); 3.81–3.91 (m, 3H, CH_2_O); 4.03 (m, 1H, H-2β); 4.20 (m, 1H, H-3β). ^13^C NMR *δ* 11.86, 13.16, 17.59, 20.34, 21.42, 24.00, 26.61, 27.65, 28.62, 32.47, 34.02 (vb), 37.56, 39.07, 40.65, 42.34, 42.42, 45.08, 45.33 (vb, 2×C), 52.20, 54.54 (b), 54.98, 59.78, 63.67, 65.14, 72.01, 72.23, 106.74, 108.86. HRMS: (API+) calculated for C_29_H_50_NO_4_ ([M + H]^+^) 476.3740, found 476.3742.

*N*,*N*-diethyl-((*20S*)-2α,3α-(1-methylethylidene)dioxy-6,6-ethylenedioxy-5α-pregnane-20-methyl)amine (**14b**)

The general procedure for amide reduction with amide **13b** (150 mg; 0.29 mmol) and chromatography on silica gel (2–4% methanol in ammonia chloroform) yielded 104 mg (71%) of the title compound **14b** as a colorless oil:

^1^H NMR (DMSO-*d*_6_, 55 °C) *δ* 0.64 (s, 3H, CH_3_); 0.75 (s, 3H, CH_3_); 0.89–1.00 (m, 9H, 3×CH_3_), 1.61–1.72 (m, 3H); 1.78 (dd, 1H, *J* = 12.7 and 6.9 Hz); 1.80 (m, 1H); 1.91–1.98 (m, 2H); 2.50–3.10 (vbs, 6H, 6×NCH), 3.66 (m, 1H, CH_2_O); 3.78–3.88 (m, 3H, CH_2_O); 3.99 (m, 1H, H-2β); 4.17 (m, 1H, H-3β). ^13^C NMR *δ* 11.76, 13.15, 14.55 (vb, 2×C), 17.65 (b), 20.32, 21.42, 23.90 (b), 26.61, 27.59 (b), 28.61, 32.47, 37.55, 39.08, 40.63, 42.34, 42.40, 45.06, 51.97 (vb, 2×C), 52.11, 55.01, 63.22 (vb, C), 63.66, 65.15, 71.99, 72.22, 106.74, 108.84, 2×C n. f. HRMS: (API+) calculated for C_31_H_54_NO_4_ ([M + H]^+^) 504.4053, found 504.4050.

1-((*20S*)-2α,3α-(1-methylethylidene)dioxy-6,6-ethylenedioxy-5α-pregnane-20-methyl)pyrrolidine (**14c**)

The general procedure for amide reduction with amide **13c** (150 mg; 0.29 mmol) and chromatography on silica gel (2–4% methanol in ammonia chloroform) yielded 110 mg (75%) of the title compound **14c** as a colorless oil:

^1^H NMR (DMSO-*d*_6_) *δ* 0.65 (s, 3H, CH_3_); 0.76 (s, 3H, CH_3_); 1.04 (d, 3H, *J* = 6.7 Hz, CH_3_); 1.21 (s, 3H, CH_3_); 1.35 (s, 3H, CH_3_); 1.62–1.72 (m, 3H); 1.73–1.81 (m, 2H); 1.91–1.98 (m, 2H); 2.66–3.14 (vbs, 6H, 6×NCH), 3.67 (m, 1H, CH_2_O); 3.80–3.84 (m, 2H, CH_2_O); 3.89 (m, 1H, CH_2_O); 4.01 (m, 1H, H-2β); 4.19 (m, 1H, H-3β). ^13^C NMR *δ* 11.81, 13.19, 17.29, 20.38, 21.45, 22.66 (2×C), 23.90, 26.65, 27.53, 28.66, 32.52, 37.60, 39.08, 40.65, 42.37, 42.46, 45.12, 52.14, 53.66 (vb, 2×C), 55.02, 60.49 (vb), 63.73, 65.19, 72.04, 72.27, 106.80, 108.88, 2×C n. f. HRMS: (API+) calculated for C_31_H_52_NO_4_ ([M + H]^+^) 502.3896, found 502.3895.

1-((*20S*)-2α,3α-(1-methylethylidene)dioxy-6,6-ethylenedioxy-5α-pregnane-20-methyl)piperidine (**14d**)

The general procedure for amide reduction with amide **13d** (150 mg; 0.28 mmol) and chromatography on silica gel (2–4% methanol in ammonia chloroform) yielded 107 mg (73%) of the title compound **14d** as a colorless oil:

^1^H NMR (DMSO-*d*_6_) *δ* 0.67 (s, 3H, CH_3_); 0.79 (s, 3H, CH_3_); 1.04 (d, 3H, *J* = 6.7 Hz, CH_3_); 1.23 (s, 3H, CH_3_); 1.37 (s, 3H, CH_3_); 1.64–1.83 (m, 5H); 1.93–2.01 (m, 2H); 2.65–3.00 (vbm, 4H, 4×NCH), 3.14–3.55 (vbs, 2H, 2×NCH), 3.70 (m, 1H, CH_2_O); 3.81–3.92 (m, 3H, CH_2_O); 4.02 (m, 1H, H-2β); 4.20 (m, 1H, H-3β). ^13^C NMR *δ* 11.76, 13.14, 17.67, 20.30, 21.41, 23.24, 23.90 (vb, 2×C), 26.60, 27.52, 28.61, 32.46, 37.55, 39.08, 40.60, 42.35, 42.39, 45.05, 52.08, 54.62 (vb, 2×C), 54.96, 60.23 (vb), 63.66, 65.12, 71.97, 72.20, 106.73, 108.82, 3×C n. f. HRMS: (API+) calculated for C_32_H_54_NO_4_ ([M + H]^+^) 516.4053, found 516.4055.

4-((*20S*)-2α,3α-(1-methylethylidene)dioxy-6,6-ethylenedioxy-5α-pregnane-20-methyl)morpholine (**14e**)

The general procedure for amide reduction with amide **13e** (150 mg; 0.28 mmol) and chromatography on silica gel (2–4% methanol in ammonia chloroform) yielded 115 mg (79%) of the title compound **14e** as a colorless oil:

^1^H NMR (DMSO-*d*_6_, 50 °C) *δ* 0.65 (s, 3H, CH_3_); 0.78 (s, 3H, CH_3_); 0.97 (d, 3H, *J* = 6.7 Hz, CH_3_); 1.23 (s, 3H, CH_3_); 1.37 (s, 3H, CH_3_); 1.63–1.72 (m, 3H); 1.78–1.82 (m, 2H); 1.89–2.00 (m, 3H); 2.13–2.19 (m, 3H, 3×NCH); 2.38–2.42 (m, 2H, 2×NCH); 3.51–3.59 (m, 4H, 2×CH_2_O_morpholine_); 3.69 (m, 1H, CH_2_O); 3.81–3.91 (m, 3H, CH_2_O); 4.02 (m, 1H, H-2β); 4.20 (m, 1H, H-3β). ^13^C NMR *δ* 11.67, 12.92, 17.72, 20.16, 21.29, 23.82, 26.17, 27.40, 28.38, 32.32, 32.99, 37.38, 38.97, 40.51, 42.21, 42.32, 44.97, 52.19, 53.80 (2×C), 54.59, 54.86, 63.43, 64.47, 64.95, 66.14 (2×C), 71.85, 72.08, 106.53, 108.72. HRMS: (API+) calculated for C_31_H_52_NO_5_ ([M + H]^+^) 518.3845, found 518.3849.

General procedure for deprotection of short side chain amine ketals 

A 5% aqueous solution of hydrochloric acid (1 mL) was added to a solution of ketals **14a**–**e** (80 mg) in tetrahydrofuran (5 mL) and the mixture was stirred at 45 °C for 2 h. After cooling, the saturated solution of sodium bicarbonate (2 mL) was added, and the reaction was stirred at room temperature for an additional 15 min. The mixture was diluted with ethyl acetate and extracted twice with water. The organic phase was dried with sodium sulfate and solvents were evaporated under reduced pressure. The crude product was washed with diethylether to obtain a pure product (no chromatography needed).

(*20S*)-20-(*N*,*N*-dimethylaminomethyl)-2α,3α-dihydroxy-5α-pregnane-6-one (**15a**)

The general procedure for deprotection with ketal **14a** (80 mg; 0.17 mmol) yielded 57 mg (88%) of the title compound **15a** as an off-white solid:

Mp 219–221 °C; >240 (decomp.). ^1^H NMR (DMSO-*d*_6_) *δ* 0.64 (s, 3H, CH_3_); 0.65 (s, 3H, CH_3_); 0.96 (d, 3H, *J* = 6.5 Hz, CH_3_); 1.67 (m, 1H); 1.79 (m, 1H); 1.97–2.13 (m, 3H); 2.19 (vbs, 6H, 2×NCH_3_); 2.60 (dd, 1H, *J* = 12.2 and 3.4 Hz); 3.46 (m, 1H, H-2β); 3.75 (m, 1H, H-3β); 4.20 (d, 1H, *J* = 2.4 Hz, OH); 4.34 (d, 1H, *J* = 6.1 Hz, OH). ^13^C NMR *δ* 11.90, 13.36, 17.55, 20.78, 23.67, 26.84, 27.57, 33.92 (vb), 36.98, 38.97, 41.84, 42.81, 45.22 (vb, 2×C), 46.00, 50.34, 52.91, 54.36 (vb), 55.61, 65.00 (vb), 67.11, 67.49, 211.50, 1×C n. f. HRMS: (API+) calculated for C_24_H_42_NO_3_ ([M + H]^+^) 392.3165, found 392.3164. Anal. Calcd. for C_24_H_41_NO_3_: C, 73.61; H, 10.55; N, 3.58. Found: C, 73.57; H, 10.59; N, 3.57%.

(*20S*)-20-(*N*,*N*-diethylaminomethyl)-2α,3α-dihydroxy-5α-pregnane-6-one (**15b**)

The general procedure for deprotection with ketal **14b** (80 mg; 0.16 mmol) yielded 56 mg (85%) of the title compound **15b** as an off-white solid:

Mp > 190 °C (decomp.). ^1^H NMR (DMSO-*d*_6_) *δ* 0.64 (s, 3H, CH_3_); 0.65 (s, 3H, CH_3_); 0.94–1.08 (m, 9H, 3×CH_3_); 1.67 (m, 1H); 1.83 (m, 1H); 1.97–2.13 (m, 3H); 2.37 (vbs, 2H, 2×NCH); 2.60 (dd, 1H, *J* = 12.1 and 3.2 Hz); 2.61 (vbs, 2H, 2×NCH); 3.46 (m, 1H, H-2β); 3.76 (m, 1H, H-3β); 4.21 (bs, 1H, OH); 4.35 (bs, 1H, OH). ^13^C NMR *δ* 11.88, 13.36, 17.80, 19.08 (vb, 2×C), 20.77, 23.69, 26.84, 27.62, 34.43 (vb), 37.00, 38.95, 41.84, 42.80, 46.01, 46.93 (vb, 2×C), 50.33, 52.91, 54.34 (vb), 55.65, 58.55 (vb), 67.11, 67.49, 211.52, 1×C n. f. HRMS: (API+) calculated for C_26_H_46_NO_3_ ([M + H]^+^) 420.3478, found 420.3476. Anal. Calcd. for C_26_H_45_NO_3_: C, 74.42; H, 10.81; N, 3.34. Found: C, 74.39; H, 10.86; N, 3.31%.

(*20S*)-20-(pyrrolidin-1-ylmethyl)-2α,3α-dihydroxy-5α-pregnane-6-one (**15c**)

The general procedure for deprotection with ketal **14c** (80 mg; 0.16 mmol) yielded 58 mg (88%) of the title compound **15c** as an off-white solid:

Mp > 240 °C (decomp.). ^1^H NMR (DMSO-*d*_6_) *δ* 0.64 (s, 3H, CH_3_); 0.65 (s, 3H, CH_3_); 1.01 (d, 3H, *J* = 6.5 Hz, CH_3_); 1.63–1.84 (m, 6H); 1.98–2.13 (m, 3H); 2.25–2.75 (vbm, 4H, 4×NCH); 2.60 (dd, 1H, *J* = 12.1 and 3.5 Hz); 3.46 (m, 1H, H-2β); 3.73 (m, 1H, H-3β); 4.21 (d, 1H, *J* = 2.4 Hz, OH); 4.34 (d, 1H, *J* = 6.1 Hz, OH). ^13^C NMR *δ* 11.85, 13.36, 17.69, 20.77, 22.94 (vb, 2×C), 23.63, 26.83, 27.59, 35.06 (vb), 36.94, 38.93, 41.82, 42.75, 45.98, 50.33, 52.88, 54.11 (vb, 3×C), 55.61, 61.35 (vb), 67.11, 67.48, 211.48, 1×C n. f. HRMS: (API+) calculated for C_26_H_44_NO_3_ ([M + H]^+^) 418.3321, found 418.3322. Anal. Calcd. for C_26_H_43_NO_3_: C, 74.77; H, 10.38; N, 3.35. Found: C, 74.74; H, 10.42; N, 3.30%.

(*20S*)-20-(piperidin-1-ylmethyl)-2α,3α-dihydroxy-5α-pregnane-6-one (**15d**)

The general procedure for deprotection with ketal **14d** (80 mg; 0.16 mmol) yielded 57 mg (85%) of the title compound **15d** as an off-white solid:

Mp > 210 °C (decomp.). ^1^H NMR (DMSO-*d*_6_) *δ* 0.64 (s, 6H, 2×CH_3_); 0.96 (d, 3H, *J* = 6.5 Hz, CH_3_); 1.66 (m, 1H); 1.79 (m, 1H); 1.92–2.13 (m, 5H, incl. 2×NCH); 2.10–2.33 (m, 2H, 2×NCH); 2.43–2.62 (m, 2H, 2×NCH); 2.60 (dd, 1H, *J* = 12.1 and 3.5 Hz); 3.47 (m, 1H, H-2β); 3.75 (m, 1H, H-3β); 4.20 (d, 1H, *J* = 2.1 Hz, OH); 4.34 (d, 1H, *J* = 5.8 Hz, OH). ^13^C NMR *δ* 11.89, 13.36, 17.90, 20.77, 23.69, 23.93 (vb), 25.32 (b, 2×C), 26.84, 27.58, 33.40 (vb), 36.99, 38.98, 41.84, 42.82, 46.01, 50.34, 52.91, 54.52 (vb, 2×C), 55.62, 64.52 (vb), 67.11, 67.49, 211.51, 1×C n. f. HRMS: (API+) calculated for C_27_H_46_NO_3_ ([M + H]^+^) 432.3478, found 432.3474. Anal. Calcd. for C_26_H_43_NO_3_: C, 74.77; H, 10.38; N, 3.35. Found: C, 74.74; H, 10.42; N, 3.30%.

(*20S*)-20-(morpholin-4-ylmethyl)-2α,3α-dihydroxy-5α-pregnane-6-one (**15e**)

The general procedure for deprotection with ketal **14e** (80 mg; 0.15 mmol) and chromatography on silica gel yielded 57 mg (86%) of the title compound **15e** as an off-white solid:

Mp > 210 °C (decomp.). ^1^H NMR (DMSO-*d*_6_) *δ* 0.64 (s, 6H, 2×CH_3_); 0.96 (d, 3H, *J* = 6.5 Hz, CH_3_); 1.66 (m, 1H); 1.80 (m, 1H); 1.92 (dd, 1H, *J* = 11.9 and 10.5 Hz); 1.98–2.19 (m, 7H, incl. 4×NCH); 2.39 (vbs, 2H, 2×NCH); 2.59 (dd, 1H, *J* = 12.2 and 3.4 Hz); 3.46 (m, 1H, H-2β); 3.50–3.58 (m, 4H, 2×CH_2_O_morpholine_); 3.75 (m, 1H, H-3β); 4.20 (d, 1H, *J* = 2.4 Hz, OH); 4.33 (d, 1H, *J* = 6.1 Hz, OH). ^13^C NMR *δ* 11.90, 13.35, 17.82, 20.76, 23.70, 26.83, 27.62, 33.11, 36.98, 38.95, 41.82, 42.82, 46.01, 50.33, 52.92, 53.92 (b, 2×C), 54.56, 55.61, 64.55, 66.30 (2×C), 67.10, 67.47, 211.48. HRMS: (API+) calculated for C_26_H_44_NO_4_ ([M + H]^+^) 434.3270, found 434.3267. Anal. Calcd. for C_26_H_43_NO_4_: C, 72.02; H, 10.00; N, 3.23. Found: C, 71.98; H, 10.04; N, 3.22%. 

General procedure for preparation of aminium chlorides

A 5% aqueous solution of hydrochloric acid (0.5 mL) was added to a solution of amine (20 mg; ~0.05 mmol) in methanol (2 mL). The mixture was shaken, evaporated to dryness and washed with diethyl ether. The prepared salts obtained in quantitative yield had sufficient purity. 

*N*,*N*-dimethyl-((*20S*)-2α,3α-dihydroxy-6-oxo-5α-pregnane-20-methyl)aminium chloride (**16a**)

Mp > 290 °C (decomp.). ^1^H NMR (D_2_O) *δ* 0.67 (s, 3H, CH_3_); 0.70 (s, 3H, CH_3_); 1.00 (d, 3H, *J* = 6.7 Hz, CH_3_); 1.75–1.83 (m, 2H); 1.88 (m, 1H); 1.99 (m, 1H); 2.17–2.20 (m, 2H); 2.68 (dd, 1H, *J* = 11.1 and 4.9 Hz); 2.77 (s, 3H, NCH_3_); 2.83 (s, 3H, NCH_3_); 2.87 (dd, 1H, *J* = 12.9 and 11.3 Hz, H-22a); 3.04 (dd, 1H, *J* = 12.9 and 3.2 Hz, H-22b); 3.73 (m, 1H, Σ*J* = 20.2 Hz, H-2β); 3.98 (m, 1H, H-3β). ^13^C NMR *δ* 11.22, 12.72, 15.69, 20.79, 23.43, 26.01, 27.23, 32.23, 38.03, 38.79, 38.92, 40.88, 43.04, 43.09, 45.18, 46.11, 50.87, 52.98, 53.00, 55.55, 63.62, 67.72, 68.11, 219.52. HRMS: (API+) calculated for C_24_H_42_NO_3_ ([M − Cl]^+^) 392.3165, found 392.3164. Anal. Calcd. for C_24_H_42_ClNO_3_: C, 67.34; H, 9.89; N, 3.27. Found: C, 67.28; H, 9.96; N, 3.23%.

*N*,*N*-diethyl-((*20S*)-2α,3α-dihydroxy-6-oxo-5α-pregnane-20-methyl)aminium chloride (**16b**)

Mp > 270 °C (decomp.). ^1^H NMR (CD_3_OD) *δ* 0.77 (s, 3H, CH_3_); 0.80 (s, 3H, CH_3_); 1.15 (d, 3H, *J* = 6.5 Hz, CH_3_); 1.87–2.00 (m, 2H); 2.09–2.15 (m, 2H); 2.21 (dd, 1H, *J* = 13.1 and 4.9 Hz); 2.73 (dd, 1H, *J* = 12.4 and 3.2 Hz); 2.89 (dd, 1H, *J* = 13.3 and 11.2 Hz, H-22a); 3.08 (dd, 1H, *J* = 13.3 and 2.4 Hz, H-22b); 3.15–3.22 (m, 2H, 2×NCH); 3.25–3.34 (m, 2H, 2×NCH); 3.66 (m, 1H, Σ*J* = 19.9 Hz, H-2β); 3.95 (m, 1H, H-3β). ^13^C NMR *δ* 8.41 (vb), 9.55 (vb), 12.51, 14.01, 17.69, 22.44, 25.07, 27.99, 29.03, 34.30, 39.16, 40.72, 41.10, 43.68, 44.71, 47.50, 47.77 (vb), 50.73 (vb), 52.22, 55.03, 55.14, 57.61, 59.51, 69.23, 69.61, 214.89. HRMS: (API+) calculated for C_26_H_46_NO_3_ ([M − Cl]^+^) 420.3478, found 420.3480. Anal. Calcd. for C_26_H_46_ClNO_3_: C, 68.47; H, 10.17; N, 3.07. Found: C, 68.40; H, 10.23; N, 3.02%.

1-((*20S*)-2α,3α-dihydroxy-6-oxo-5α-pregnane-20-methyl)pyrrolidin-1-ium chloride (**16c**)

Mp > 260 °C (decomp.). ^1^H NMR (DMSO-*d*_6_) *δ* 0.61 (s, 3H, CH_3_); 0.63 (s, 3H, CH_3_); 1.06 (d, 3H, *J* = 6.5 Hz, CH_3_); 1.83–1.97 (m, 5H); 2.00 (dd, 1H, *J* = 13.1 and 4.9 Hz); 2.08 (bt, 1H, *J* = 12.3 Hz); 2.57 (dd, 1H, *J* = 11.9 and 3.4 Hz); 2.86–3.00 (m, 4H, 4×NCH); 3.43 (m, 1H, H-2β); 3.46–3.53 (m, 2H, 2×NCH); 3.72 (m, 1H, H-3β); 4.18 (d, 1H, *J* = 2.4 Hz, OH); 4.32 (d, 1H, *J* = 6.1 Hz, OH); 9.62 (bs. 1H, NH). ^13^C NMR *δ* 11.79, 13.36, 17.26, 20.78, 22.41, 22.69, 23.49, 26.84, 27.39, 33.51, 36.93, 38.85, 41.81, 42.78, 45.91, 50.34, 52.11, 52.80, 53.23, 55.50, 55.61, 60.15, 67.11, 67.49, 211.46, 1×C n. f. HRMS: (API+) calculated for C_26_H_44_NO_3_ ([M − Cl]^+^) 418.3321, found 418.3321. Anal. Calcd. for C_26_H_44_ClNO_3_: C, 68.77; H, 9.77; N, 3.08. Found: C, 68.71; H, 9.83; N, 3.03%.

1-((*20S*)-2α,3α-dihydroxy-6-oxo-5α-pregnane-20-methyl)piperidin-1-ium chloride (**16d**)

Mp > 280 °C (decomp.). ^1^H NMR (DMSO-*d*_6_) *δ* 0.65 (s, 3H, CH_3_); 0.67 (s, 3H, CH_3_); 1.08 (d, 3H, *J* = 6.5 Hz, CH_3_); 1.83–1.93 (m, 2H); 1.99 (m, 1H); 2.04 (dd, 1H, *J* = 13.1 and 4.9 Hz); 2.11 (bt, 1H, *J* = 12.4 Hz); 2.60 (dd, 1H, *J* = 12.1 and 3.5 Hz); 2.73–2.80 (m, 2H, 2×NCH); 2.89–2.97 (m, 2H, 2×NCH); 3.29–3.34 (m, 2H, 2×NCH); 3.46 (m, 1H, H-2β); 3.76 (m, 1H, H-3β); 9.25 (bs. 1H, NH). ^13^C NMR *δ* 11.78, 13.36, 17.87, 20.76, 21.39, 22.01, 22.04, 23.48, 26.84, 27.41, 31.50, 36.93, 38.85, 41.81, 42.81, 45.91, 50.34, 50.76, 52.77, 53.33, 54.64, 55.62, 61.99, 67.11, 67.49, 211.47, 1×C n. f. HRMS: (API+) calculated for C_27_H_46_NO_3_ ([M − Cl]^+^) 432.3478, found 432.3475. Anal. Calcd. for C_27_H_46_ClNO_3_: C, 69.28; H, 9.90; N, 2.99. Found: C, 69.20; H, 9.98; N, 2.95%.

4-((*20S*)-2α,3α-dihydroxy-6-oxo-5α-pregnane-20-methyl)morpholin-4-ium chloride (**16e**)

Mp > 240 °C (decomp.). ^1^H NMR (DMSO-*d*_6_) *δ* 0.62 (s, 3H, CH_3_); 0.64 (s, 3H, CH_3_); 1.03 (d, 3H, *J* = 6.5 Hz, CH_3_); 1.65 (m, 1H); 1.76 (m, 1H); 1.84 (m, 1H); 1.96 (m, 1H); 2.03 (dd, 1H, *J* = 13.1 and 4.9 Hz); 2.08 (bt, 1H, *J* = 12.5 Hz); 2.58 (dd, 1H, *J* = 12.4 and 3.5 Hz); 2.83 (dd, 1H, *J* = 12.9 and 11.0 Hz, NCH); 2.96 (td, 1H, *J* = 12.2 and 3.8 Hz, NCH) 3.02 (dd, 1H, *J* = 12.9 and 2.3 Hz, NCH); 3.13 (td, 1H, *J* = 12.2 and 3.8 Hz, NCH); 3.30 (bd, 1H, *J* = 11.6 Hz, NCH); 3.43–3.48 (m, 2H, H-2β, NCH); 3.70 (m, 1H, OCH_morph_); 3.75–3.81 (m, 2H, H-3β and OCH_morph_); 3.88–3.93 (m, 2H, 2×OCH_morph_). ^13^C NMR *δ* 12.15, 13.75, 17.63, 21.19, 23.90, 27.12, 27.69, 31.43, 37.48, 40.23, 42.34, 43.28, 46.34, 50.38, 50.79, 53.20, 53.49, 53.73, 55.96, 62.65, 63.34, 63.46, 67.44, 67.79, 212.71, 1×C n. f. HRMS: (API+) calculated for C_26_H_44_NO_4_ ([M − Cl]^+^) 434.3270, found 434.3272. Anal. Calcd. for C_26_H_44_ClNO_4_: C, 66.43; H, 9.43; N, 2.98. Found: C, 66.38; H, 9.48; N, 2.95%.

(*20S*,*22R*,*23S*)-2α,3α;22,23-bis((1-methylethylidene)dioxy)-6,6-ethylenedioxy-5α-cholan-24-oic acid (**18**)

Potassium hydroxide (570 mg; 10.18 mmol) was added to a solution of ethyl ester **17** (1.2 g; 2.03 mmol) in methanol (80 mL) and water (10 mL). The reaction mixture was stirred under reflux for 1 h. After cooling to room temperature, the excess of methanol was removed under reduced pressure, and the residue was diluted in ethyl acetate and extracted with 5% aqueous solution of citric acid and twice with water. The organic phase was dried over sodium sulfate and solvents were evaporated under reduced pressure to obtain acid **18** (795 mg; 96%) as a colorless oil in adequate purity for other reactions. 

^1^H NMR (DMSO-*d*_6_) *δ* 0.61 (s, 3H, CH_3_); 0.76 (s, 3H, CH_3_); 0.89 (d, 3H, *J* = 6.5 Hz, CH_3_); 1.21 (s, 3H, CH_3_); 1.26 (s, 3H, CH_3_); 1.33 (s, 3H, CH_3_); 1.35 (s, 3H, CH_3_); 1.76–1.86 (m, 2H); 1.91 (m, 1H); 1.96 (m, 1H); 3.67 (m, 1H, CH_2_O); 3.78–3.90 (m, 3H, CH_2_O); 4.01 (m, 1H, H-2β); 4.11–4.14 (m, 2H, H-22, H-23); 4.18 (m, 1H, H-3β). HRMS: (API-) calculated for C_32_H_49_O_8_ ([M-H]^-^) 561.3427, found 561.3426.

*N*,*N*-dimethyl-(*20S*,*22R*,*23S*)-2α,3α;22,23-bis((1-methylethylidene)dioxy)-6,6-ethylenedioxy-5α-cholan-24-amide (**19a**)

The general procedure for amide formation with acid **18** and dimethylamine and chromatography on silica gel (40–60% ethyl acetate in cyclohexane) yielded 191 mg (91%) of the title compound **19a** as a colorless oil:

^1^H NMR (DMSO-*d*_6_) *δ* 0.60 (s, 3H, CH_3_); 0.77 (s, 3H, CH_3_); 0.91 (d, 3H, *J* = 6.7 Hz, CH_3_); 1.22 (s, 3H, CH_3_); 1.23 (s, 3H, CH_3_); 1.33 (s, 3H, CH_3_); 1.36 (s, 3H, CH_3_); 1.62–1.74 (m, 3H); 1.77–1.86 (m, 2H); 1.91 (td, 1H, *J* = 11.9 and 3.2 Hz); 1.97 (m, 1H); 2.83 (s, 3H, NCH_3_); 3.04 (s, 3H, NCH_3_); 3.67 (m, 1H, CH_2_O); 3.81–3.90 (m, 3H, CH_2_O); 4.02 (m, 1H, H-2β); 4.19 (m, 1H, H-3β); 4.33 (d, 1H, *J* = 7.6 Hz, H-23); 4.53 (dd, 1H, *J* = 7.6 and 1.1 Hz, H-22). ^13^C NMR *δ* 11.54, 12.61, 13.12, 20.36, 21.42, 23.73, 25.98, 26.60, 26.70, 27.14, 28.62, 32.54, 35.07, 35.55, 36.48, 37.55, 40.65, 41.93, 42.40, 45.08, 52.09, 53.23, 55.03, 63.64, 65.14, 72.00, 72.22, 73.65, 78.97, 106.73, 108.61, 108.85, 168.43, 1×C n. f. HRMS: (API+) calculated for C_34_H_56_NO_7_ ([M + H]^+^) 590.4057, found 590.4061.

*N,N*-diethyl-(*20S,22R,23S*)-2α,3α;22,23-bis((1-methylethylidene)dioxy)-6,6-ethylenedioxy-5α-cholan-24-amide (**19b**)

The general procedure for amide formation with acid **18** and diethylamine and chromatography on silica gel (40–60% ethyl acetate in cyclohexane) yielded 193 mg (88%) of the title compound **19b** as a colorless oil:

^1^H NMR (DMSO-*d*_6_) *δ* 0.60 (s, 3H, CH_3_); 0.77 (s, 3H, CH_3_); 0.91 (d, 3H, *J* = 6.7 Hz, CH_3_); 1.01 (t, 3H, *J* = 7.0 Hz, CH_3_); 1.11 (t, 3H, *J* = 7.0 Hz, CH_3_); 1.218 (s, 3H, CH_3_); 1.221 (s, 3H, CH_3_); 1.33 (s, 3H, CH_3_); 1.36 (s, 3H, CH_3_); 1.62–1.74 (m, 3H); 1.77–1.86 (m, 2H); 1.91 (td, 1H, *J* = 11.9 and 3.1 Hz); 1.97 (m, 1H); 3.18–3.41 (m, 4H, 2×NCH_2_); 3.67 (m, 1H, CH_2_O); 3.81–3.90 (m, 3H, CH_2_O); 4.02 (m, 1H, H-2β); 4.19 (m, 1H, H-3β); 4.27 (d, 1H, *J* = 7.2 Hz, H-23); 4.53 (dd, 1H, *J* = 7.2 and 1.2 Hz, H-22). ^13^C NMR *δ* 11.59, 12.65, 12.71, 13.17, 14.39, 20.40, 21.46, 23.77, 25.84, 26.65, 26.72, 27.25, 28.66, 32.58, 35.92, 37.60, 39.04, 39.57, 40.68, 41.07, 41.98, 42.45, 45.12, 52.12, 53.23, 55.07, 63.69, 65.18, 72.04, 72.27, 74.04, 79.09, 106.78, 108.70, 108.88, 168.14. HRMS: (API+) calculated for C_36_H_60_NO_7_ ([M + H]^+^) 618.4370, found 618.4375. 

1-((*20S*,*22R*,*23S*)-2α,3α;22,23-bis((1-methylethylidene)dioxy)-6,6-ethylenedioxy-5α-cholan-24-oyl) pyrrolidine (**19c**)

The general procedure for amide formation with acid **18** and pyrrolidine and chromatography on silica gel (40–60% ethyl acetate in cyclohexane) yielded 195 mg (89%) of the title compound **19c** as a colorless oil:

^1^H NMR (DMSO-*d*_6_) *δ* 0.60 (s, 3H, CH_3_); 0.76 (s, 3H, CH_3_); 0.91 (d, 3H, *J* = 6.7 Hz, CH_3_); 1.22 (s, 3H, CH_3_); 1.24 (s, 3H, CH_3_); 1.32 (s, 3H, CH_3_); 1.36 (s, 3H, CH_3_); 1.62–1.72 (m, 3H); 1.73–1.88 (m, 6H); 1.91 (td, 1H, *J* = 11.8 and 3.1 Hz); 1.97 (m, 1H); 3.25–3.33 (m, 2H, NCH_2_); 3.54 (t, 2H, *J* = 6.7 Hz, NCH_2_); 3.67 (m, 1H, CH_2_O); 3.81–3.90 (m, 3H, CH_2_O); 4.02 (m, 1H, H-2β); 4.19 (m, 1H, H-3β); 4.24 (d, 1H, *J* = 7.4 Hz, H-23); 4.49 (dd, 1H, *J* = 7.4 and 1.1 Hz, H-22). ^13^C NMR *δ* 11.56, 12.62, 13.14, 20.38, 21.43, 23.46, 23.73, 25.64, 25.92, 26.61, 26.70, 27.19, 28.63, 32.55, 35.76, 37.56, 40.65, 41.94, 42.41, 45.09, 45.83, 45.94, 52.10, 53.18, 55.04, 63.65, 65.15, 72.01, 72.23, 75.15, 79.03, 106.74, 108.75, 108.86, 167.32, 1×C n. f. HRMS: (API+) calculated for C_36_H_58_NO_7_ ([M + H]^+^) 616.4213, found 616.4218.

1-((*20S,22R,23S*)-2α,3α;22,23-bis((1-methylethylidene)dioxy)-6,6-ethylenedioxy-5α-cholan-24-oyl) piperidine (**19d**)

The general procedure for amide formation with acid **18** and piperidine and chromatography on silica gel (40–60% ethyl acetate in cyclohexane) yielded 188 mg (84%) of the title compound **19d** as a colorless oil:

^1^H NMR (DMSO-*d*_6_) *δ* 0.60 (s, 3H, CH_3_); 0.77 (s, 3H, CH_3_); 0.90 (d, 3H, *J* = 6.7 Hz, CH_3_); 1.22 (s, 6H, 2×CH_3_); 1.33 (s, 3H, CH_3_); 1.36 (s, 3H, CH_3_); 1.62–1.74 (m, 3H); 1.77–1.86 (m, 2H); 1.91 (m, 1H); 1.97 (m, 1H); 3.41–3.60 (m, 4H, 2×NCH_2_); 3.67 (m, 1H, CH_2_O); 3.81–3.90 (m, 3H, CH_2_O); 4.02 (m, 1H, H-2β); 4.19 (m, 1H, H-3β); 4.32 (d, 1H, *J* = 7.3 Hz, H-23); 4.58 (dd, 1H, *J* = 7.3 and 0.9 Hz, H-22). ^13^C NMR *δ* 11.56, 12.65, 13.13, 20.38, 21.42, 23.74, 24.05, 25.35, 25.91, 26.12, 26.61, 26.69, 27.18, 28.62, 32.54, 35.85, 37.56, 40.65, 41.95, 42.41, 42.48, 45.09, 45.86, 52.11, 53.23, 55.04, 63.64, 65.14, 72.01, 72.23, 73.84, 78.89, 106.74, 108.60, 108.86, 167.02, 1×C n. f. HRMS: (API+) calculated for C_37_H_60_NO_7_ ([M + H]^+^) 630.4370, found 630.4370.

4-((*20S*,*22R*,*23S*)-2α,3α;22,23-bis((1-methylethylidene)dioxy)-6,6-ethylenedioxy-5α-cholan-24-oyl) morpholine (**19e**)

The general procedure for amide formation with acid **18** and morpholine and chromatography on silica gel (40–60% ethyl acetate in cyclohexane) yielded 191 mg (85%) of the title compound **19e** as a colorless oil:

^1^H NMR (DMSO-*d*_6_) *δ* 0.62 (s, 3H, CH_3_); 0.78 (s, 3H, CH_3_); 0.92 (d, 3H, *J* = 6.7 Hz, CH_3_); 1.23 (s, 3H, CH_3_); 1.24 (s, 3H, CH_3_); 1.34 (s, 3H, CH_3_); 1.37 (s, 3H, CH_3_); 1.63–1.75 (m, 3H); 1.78–1.87 (m, 2H); 1.92 (td, 1H, *J* = 11.7 and 2.9 Hz); 1.99 (m, 1H); 3.43–3.63 (m, 8H, 8×H_morph_); 3.68 (m, 1H, CH_2_O); 3.81–3.91 (m, 3H, CH_2_O); 4.02 (m, 1H, H-2β); 4.20 (m, 1H, H-3β); 4.36 (d, 1H, *J* = 7.3 Hz, H-23); 4.58 (dd, 1H, *J* = 7.3 and 1.2 Hz, H-22). ^13^C NMR *δ* 11.60, 12.65, 13.15, 20.39, 21.46, 23.77, 25.92, 26.64, 26.67, 27.20, 28.65, 32.58, 35.87, 37.59, 40.68, 41.99, 42.05, 42.44, 45.12, 45.66, 52.13, 53.24, 55.07, 63.68, 65.18, 66.09, 66.32, 72.04, 72.26, 73.69, 78.85, 106.78, 108.81, 108.88, 167.63, 1×C n. f. HRMS: (API+) calculated for C_36_H_58_NO_8_ ([M − Cl]^+^) 632.4162, found 632.4168.

General procedure for deprotection of long side chain amide ketals 

A 5% aqueous solution of hydrochloric acid (1 mL) was added to a solution of ketals **19a**–**e** (20 mg) in tetrahydrofuran (5 mL) and the mixture was stirred at 45 °C for 2 h. After cooling, the mixture was diluted with ethyl acetate and extracted twice with water. The organic phase was dried with sodium sulfate and solvents were evaporated under reduced pressure. The crude product was purified on silica gel (mobile phase at each experiment).

*N*,*N*-dimethyl-(*20S*,*22R*,*23S*)-2α,3α,22,23-tetrahydroxy-6-oxo-5α-cholan-24-amide (**20a**)

The general procedure for amide deprotection with ketal **19a** (50 mg; 0.08 mmol) and chromatography on silica gel (5–10% isopropanol in chloroform) yielded 14 mg (86%) of the title compound **20a** as an off-white solid:

Mp > 210 °C (decomp.). ^1^H NMR (CD_3_OD+CDCl_3_) *δ* 0.65 (s, 3H, CH_3_); 0.73 (s, 3H, CH_3_); 0.99 (d, 3H, *J* = 6.7 Hz, CH_3_); 1.75–1.82 (m, 2H); 1.95 (m, 1H); 1.99–2.05 (m, 2H); 2.23 (dd, 1H, *J* = 13.1 and 4.6 Hz); 2.68 (dd, 1H, *J* = 12.4 and 2.9 Hz); 2.95 (s, 3H, NCH_3_); 3.08 (s, 3H, NCH_3_); 3.64 (m, 1H, H-2β); 3.77 (dd, 1H, *J* = 5.7 and 1.2 Hz, H-22); 3.94 (m, 1H, H-3β); 4.32 (d, 1H, *J* = 5.7 Hz, H-23). ^13^C NMR *δ* 12.33, 13.45, 14.00, 21.90, 24.57, 27.14, 28.42, 36.41, 37.56, 38.67, 39.89, 40.14, 40.47, 43.38, 43.53, 47.30, 51.65, 53.34, 54.42, 57.31, 68.10, 68.90, 71.50, 73.91, 173.67, 214.81. HRMS: (API+) calculated for C_26_H_44_NO_6_ ([M + H]^+^) 466.3169, found 466.3172. Anal. Calcd. for C_26_H_43_NO_6_: C, 67.07; H, 9.31; N, 3.01. Found: C, 68.40; H, 10.23; N, 3.02%.

*N*,*N*-diethyl-(*20S*,*22R*,*23S*)-2α,3α,22,23-tetrahydroxy-6-oxo-5α-cholan-24-amide (**20b**)

The general procedure for amide deprotection with ketal **19b** (50 mg; 0.08 mmol) and chromatography on silica gel (5–10% isopropanol in chloroform) yielded 14 mg (88%) of the title compound **20b** as an off-white solid:

Mp > 160 °C (decomp.). ^1^H NMR (CD_3_OD+CDCl_3_) *δ* 0.64 (s, 3H, CH_3_); 0.73 (s, 3H, CH_3_); 0.99 (d, 3H, *J* = 6.7 Hz, CH_3_); 1.10 (t, 3H, *J* = 7.2 Hz, CH_3_); 1.22 (t, 3H, *J* = 7.2 Hz, CH_3_); 1.94 (m, 1H), 1.99–2.05 (m, 2H); 2.23 (dd, 1H, *J* = 13.1 and 4.6 Hz); 2.68 (dd, 1H, *J* = 12.5 and 2.8 Hz); 3.27 (m, 1H, NCH); 3.37–3.42 (m, 2H, 2×NCH); 3.47 (m, 1H, NCH); 3.64 (m, 1H, H-2β); 3.76 (d, 1H, *J* = 6.2 Hz, H-22); 3.94 (m, 1H, H-3β); 4.24 (d, 1H, *J* = 6.2 Hz, H-23). ^13^C NMR *δ* 12.27, 13.01, 13.61, 13.95, 14.78, 21.81, 24.49, 27.03, 28.29, 38.58, 39.21, 40.03, 40.38, 41.22, 42.50, 43.30, 43.46, 47.23, 51.56, 53.27, 54.32, 57.22, 68.57, 68.80, 71.21, 74.44, 172.76, 214.71. HRMS: (API+) calculated for C_28_H_48_NO_6_ ([M + H]^+^) 494.3482, found 494.3481. Anal. Calcd. for C_28_H_47_NO_6_: C, 68.12; H, 9.60; N, 2.84. Found: C, 68.06; H, 9.66; N, 2.80%.

1-((*20S*,*22R*,*23S*)-2α,3α,22,23-tetrahydroxy-6-oxo-5α-cholan-24-oyl)pyrrolidine (**20c**)

The general procedure for amide deprotection with ketal **19c** (50 mg; 0.08 mmol) and chromatography on silica gel (5–10% isopropanol in chloroform) yielded 13 mg (83%) of the title compound **20c** as an off-white solid:

Mp > 200 °C (decomp.). ^1^H NMR (CD_3_OD+CDCl_3_) *δ* 0.69 (s, 3H, CH_3_); 0.76 (s, 3H, CH_3_); 1.00 (d, 3H, *J* = 6.7 Hz, CH_3_); 1.84–2.07 (m, 6H); 2.10 (bt, 1H, *J* = 12.5 Hz); 2.22 (dd, 1H, *J* = 12.8 and 4.8 Hz); 2.78 (dd, 1H, *J* = 12.5 and 3.1 Hz); 3.38–3.48 (m, 2H, 2×NCH); 3.56 (m, 1H, NCH); 3.64-3.71 (m, 2H, H-2β, NCH); 3.83 (d, 1H, *J* = 7.1 Hz, H-22); 3.95 (m, 1H, H-3β); 4.22 (d, 1H, *J* = 7.1 Hz, H-23). ^13^C NMR *δ* 12.40, 13.93, 14.02, 22.48, 25.04 (2×C); 27.23, 27.97, 28.81, 39.31, 39.55, 40.95, 41.11, 43.76, 44.07, 47.45, 47.63, 47.98, 52.21, 54.12, 55.16, 58.01, 69.26, 69.90, 74.01, 74.82, 173.02, 215.17. HRMS: (API+) calculated for C_28_H_46_NO_6_ ([M + H]^+^) 492.3325, found 492.3325. Anal. Calcd. for C_28_H_45_NO_6_: C, 68.40; H, 9.23; N, 2.85. Found: C, 68.35; H, 9.30; N, 2.81%.

1-((*20S*,*22R*,*23S*)-2α,3α,22,23-tetrahydroxy-6-oxo-5α-cholan-24-oyl)piperidine (**20d**)

The general procedure for amide deprotection with ketal **19d** (50 mg; 0.08 mmol) and chromatography on silica gel (5–10% isopropanol in chloroform) yielded 14 mg (85%) of the title compound **20d** as an off-white solid:

Mp > 200 °C (decomp.). ^1^H NMR (DMSO-*d*_6_) *δ* 0.58 (s, 3H, CH_3_); 0.63 (s, 3H, CH_3_); 0.87 (d, 3H, *J* = 6.5 Hz, CH_3_); 1.80–1.86 (m, 2H); 1.94 (m, 1H); 2.03 (dd, 1H, *J* = 13.0 and 4.7 Hz); 2.1 (m, 1H); 2.60 (dd, 1H, *J* = 12.1 and 3.2 Hz); 3.34–3.52 (m, 5H, H-2β, 2×NCH_2_); 3.60 (d, 1H, *J* = 6.8 Hz, H-22); 3.75 (bs, 1H, H-3β); 4.16–4.21 (m, 2H, H-23, OH); 4.34 (bd, 1H, *J* = 5.9 Hz, OH); 4.37 (bd, 1H, *J* = 4.6 Hz, OH); 4.94 (bd, 1H, *J* = 4.9 Hz, OH). ^13^C NMR *δ* 11.73, 13.00, 13.37, 20.82, 23.48, 24.08, 25.45, 26.20, 26.85, 27.21, 29.03, 37.08, 37.90, 41.85, 42.21, 42.40, 45.69, 46.01, 50.33, 52.23, 52.88, 55.98, 67.11, 67.49, 71.47, 72.48, 170.11, 211.61, 1×C n. f. HRMS: (API+) calculated for C_29_H_48_NO_6_ ([M + H]^+^) 506.3482, found 506.3479. Anal. Calcd. for C_29_H_47_NO_6_: C, 68.88; H, 9.37; N, 2.77. Found: C, 68.82; H, 9.42; N, 2.75%.

4-((*20S*,*22R*,*23S*)-2α,3α,22,23-tetrahydroxy-6-oxo-5α-cholan-24-oyl)morpholine (**20e**)

The general procedure for amide deprotection with ketal **19e** (50 mg; 0.08 mmol) and chromatography on silica gel (5–10% isopropanol in chloroform) yielded 14 mg (87%) of the title compound **20e** as an off-white solid:

Mp > 210 °C (decomp.). ^1^H NMR (CD_3_OD+CDCl_3_) *δ* 0.66 (s, 3H, CH_3_); 0.74 (s, 3H, CH_3_); 0.98 (d, 3H, *J* = 6.7 Hz, CH_3_); 1.76–1.81 (m, 2H); 1.96 (m, 1H); 2.00–2.06 (m, 2H); 2.23 (dd, 1H, *J* = 13.0 and 4.6 Hz); 2.69 (dd, 1H, *J* = 12.4 and 2.9 Hz); 3.56–3.69 (m, 9H, H-2β, 8×Hmorph); 3.78 (dd, 1H, *J* = 6.2 and 0.9 Hz, H-22); 3.95 (m, 1H, H-3β); 4.30 (d, 1H, *J* = 6.2 Hz, H-23). 13C NMR δ 12.42, 13.42, 14.01, 21.95, 24.62, 27.20, 28.49, 30.39, 38.72, 39.45, 40.20, 40.51, 43.41, 43.46, 43.59, 48.95, 47.33, 51.70, 53.35, 54.47, 57.37, 67.48 (2×C), 68.69, 68.94, 72.08, 74.14, 172.27, 214.87. HRMS: (API+) calculated for C28H46NO7 ([M + H]+) 508.3274, found 508.3272. Anal. Calcd. for C28H45NO7: C, 66.25; H, 8.93; N, 2.76. Found: C, 66.20; H, 8.99; N, 2.73%.

4-((*20S*,*22R*,*23S*)-2α,3α-dihydroxy-22,23-(1-methylethylidene)dioxy-6-oxo-5α-cholan-24-oyl)morpholine (**23**)

The general procedure for amide deprotection (at room temperature) with ketal **19e** (50 mg; 0.08 mmol) and chromatography on silica gel (isopropanol/ethyl acetate—1/19) yielded 31 mg (72%) of the title compound 23 as a colorless oil. The product was obtained during optimization of the reaction condition.

^1^H NMR (DMSO-*d*_6_, 45 °C) *δ* 0.63 (s, 3H, CH_3_); 0.65 (s, 3H, CH_3_); 0.93 (d, 3H, *J* = 6.7 Hz, CH_3_); 1.25 (s, 3H, CH_3_); 1.34 (s, 3H, CH_3_); 1.68 (qd, 1H, *J* = 10.9 and 5.7 Hz); 1.87 (m, 1H); 1.97 (m, 1H); 2.03–2.11 (m, 2H); 2.61 (dd, 1H, *J* = 12.4 and 3.5 Hz); 3.44–3.64 (m, 9H, H-2β, 8×H_morph_); 3.77 (m, 1H, H-3β); 4.06 (bd, 1H, *J* = 2.4 Hz, OH); 4.21 (bd, 1H, *J* = 6.1 Hz, OH); 4.37 (d, 1H, *J* = 7.2 Hz, H-23); 4.59 (dd, 1H, *J* = 7.2 and 1.4 Hz, H-22). ^13^C NMR *δ* 11.61, 12.63, 13.35, 20.82, 23.39, 25.92, 26.63, 26.83, 27.09, 31.31, 35.93, 37.09, 38.91, 41.83, 42.06, 42.45, 45.65, 46.02, 50.34, 52.88, 53.08, 55.72, 66.08, 66.29, 66.98, 67.51, 73.77, 78.85, 108.82, 167.65, 211.50. HRMS: (API+) calculated for C_31_H_50_NO_7_ ([M + H]^+^) 548.3587, found 548.3586.

*N*,*N*-dimethyl-((*20S*,*22R*,*23S*)-2α,3α;22,23-bis((1-methylethylidene)dioxy)-6,6-ethylenedioxy-5α-cholan-24-yl)amine (**21a**)

The general procedure for amide reduction with amide **19a** (150 mg; 0.25 mmol) and chromatography on silica gel (0–5% methanol in ethyl acetate) yielded 114 mg (78%) of the title compound **21a** as a colorless oil:

^1^H NMR (DMSO-*d*_6_) *δ* 0.59 (s, 3H, CH_3_); 0.73 (s, 3H, CH_3_); 0.87 (d, 3H, *J* = 6.5 Hz, CH_3_); 1.18 (s, 3H, CH_3_); 1.22 (s, 3H, CH_3_); 1.23 (s, 3H, CH_3_); 1.32 (s, 3H, CH_3_); 1.58–1.70 (m, 3H); 1.74–1.82 (m, 2H); 1.86–1.94 (m, 2H); 2.13 (s, 6H, 2×NCH3); 2.24 (dd, 1H, *J* = 12.9 and 6.7 Hz, H-24a); 2.34 (dd, 1H, *J* = 12.9 and 3.4 Hz, H-24b); 3.61–3.66 (m, 2H, CH2O, H-22); 3.72–3.87 (m, 4H, 3×CH2O, H-23); 3.99 (m, 1H, H-2β); 4.15 (m, 1H, H-3β). ^13^C NMR *δ* 11.45, 12.53, 13.04, 20.29, 21.34, 23.62, 26.46, 26.93, 26.96, 27.25, 28.47, 32.42, 35.14, 37.47, 40.53, 41.89, 42.32, 44.96, 45.26 (b, 2×C), 52.18, 52.92, 55.12, 61.27, 63.55, 65.03, 71.99, 72.19, 74.76 (b), 80.64, 106.63, 107.74, 108.81, 1×C n. f. HRMS: (API+) calculated for C_34_H_58_NO_6_ ([M + H]^+^) 576.4264, found 576.4369.

*N*,*N*-diethyl-((*20S*,*22R*,*23S*)-2α,3α;22,23-bis((1-methylethylidene)dioxy)-6,6-ethylenedioxy-5α-cholan-24-yl)amine (**21b**)

The general procedure for amide reduction with amide **19b** (150 mg; 0.24 mmol) and chromatography on silica gel (0–5% methanol in ethyl acetate) yielded 110 mg (75%) of the title compound **21b** as a colorless oil:

^1^H NMR (DMSO-*d*_6_) *δ* 0.62 (s, 3H, CH_3_); 0.76 (s, 3H, CH_3_); 0.90 (d, 3H, *J* = 6.7 Hz, CH_3_); 0.92–0.99 (m, 6H, 2×CH3); 1.22 (s, 3H, CH3); 1.27 (s, 6H, 2×CH3); 1.36 (s, 3H, CH3); 1.60–1.74 (m, 4H); 1.77–1.85 (m, 2H); 1.90–1.98 (m, 2H); 2.34–2.67 (vb m, 6H, 6×NCH); 3.67 (m, 1H, CH2O); 3.72 (d, 1H, *J* = 8.3 Hz, H-22); 3.80–3.86 (m, 3H, 2×CH2O, H-23); 3.89 (m, 1H, CH2O); 4.02 (m, 1H, H-2β); 4.19 (m, 1H, H-3β). 13C NMR δ 11.53, 11.74 (vb, 2×C), 12.64, 13.15, 20.40, 21.44, 23.70, 26.62, 27.04, 27.14, 27.19, 28.64, 32.58, 35.22, 37.56, 39.11, 40.65, 41.95, 42.42, 45.11, 47.07, 52.14, 53.18, 55.09, 55.67 (vb), 63.67, 65.15, 72.01, 72.23, 74.84 (vb) 81.34 (vb), 106.75, 107.32 (vb), 108.87, 1×C n. f. HRMS: (API+) calculated for C_36_H_62_NO_6_ ([M + H]^+^) 604.4577, found 604.4579.

1-((*20S*,*22R*,*23S*)-2α,3α;22,23-bis((1-methylethylidene)dioxy)-6,6-ethylenedioxy-5α-cholan-24-yl) pyrrolidine (**21c**)

The general procedure for amide reduction with amide **19c** (150 mg; 0.24 mmol) and chromatography on silica gel (0–5% methanol in ethyl acetate) yielded 113 mg (77%) of the title compound **21c** as a colorless oil:

^1^H NMR (DMSO-*d*_6_) *δ* 0.62 (s, 3H, CH_3_); 0.76 (s, 3H, CH_3_); 0.91 (d, 3H, *J* = 6.7 Hz, CH_3_); 1.22 (s, 3H, CH_3_); 1.28 (s, 6H, 2×CH3); 1.35 (s, 3H, CH3); 1.62–1.74 (m, 7H); 1.76–1.84 (m, 2H); 1.89–1.99 (m, 2H); 2.55–2.71 (vb m, 6H, 6×NCH); 3.65–3.69 (m, 2H, CH2O, H-22); 3.80–3.90 (m, 4H, 3×CH2O, H-23); 4.01 (m, 1H, H-2β); 4.19 (m, 1H, H-3β). 13C NMR δ 11.51, 12.70, 13.14, 20.38, 21.43, 23.01 (2×C); 23.70, 26.61, 27.04, 27.14, 27.27, 28.63, 32.55, 35.17, 37.56, 40.65, 41.95, 42.42, 45.10, 52.11, 53.11, 54.13 (2×C), 55.07, 63.66, 65.15, 72.00, 72.23, 75.46, 80.77, 106.74, 107.82 (vb), 108.86, 2×C n. f. HRMS: (API+) calculated for C_36_H_60_NO_6_ ([M + H]^+^) 602.4421, found 602.4426.

1-((*20S*,*22R*,*23S*)-2α,3α;22,23-bis((1-methylethylidene)dioxy)-6,6-ethylenedioxy-5α-cholan-24-yl) piperidine (**21d**)

The general procedure for amide reduction with amide **19d** (150 mg; 0.24 mmol) and chromatography on silica gel (0–5% methanol in ethyl acetate) yielded 106 mg (72%) of the title compound **21d** as a colorless oil:

^1^H NMR (DMSO-*d*_6_) *δ* 0.65 (s, 3H, CH_3_); 0.78 (s, 3H, CH_3_); 0.91 (d, 3H, *J* = 6.7 Hz, CH_3_); 1.23 (s, 3H, CH_3_); 1.268 (s, 3H, CH_3_); 1.274 (s, 3H, CH_3_); 1.37 (s, 3H, CH_3_); 1.61–1.75 (m, 4H); 1.79–1.87 (m, 2H); 1.91–2.01 (m, 2H); 2.33–2.50 (vb m, 6H, 6×NCH); 3.67–3.72 (m, 2H, CH2O, H-22); 3.77–3.91 (m, 4H, 3×CH2O, H-23); 4.02 (m, 1H, H-2β); 4.20 (m, 1H, H-3β). 13C NMR δ 11.55, 12.74, 13.15, 20.39, 21.43, 23.75, 25.50 (2×C), 26.62, 27.10, 27.18, 27.26, 28.63, 32.57, 35.20, 37.56, 40.65, 41.98, 42.43, 45.11, 52.13, 53.20, 54.73 (b, 2×C), 55.10, 61.34 (vb), 63.67, 65.14, 72.02, 72.24, 74.80 (vb), 81.34 (b), 106.75, 107.52, 108.87, 1×C n. f. HRMS: (API+) calculated for C_37_H_62_NO_6_ ([M + H]^+^) 616.4577, found 616.4578.

4-((*20S*,*22R*,*23S*)-2α,3α;22,23-bis((1-methylethylidene)dioxy)-6,6-ethylenedioxy-5α-cholan-24-yl) morpholine (**21e**)

The general procedure for amide reduction with amide **19e** (150 mg; 0.24 mmol) and chromatography on silica gel (0–5% methanol in ethyl acetate) yielded 109 mg (74%) of the title compound **21e** as a colorless oil:

^1^H NMR (DMSO-*d*_6_) *δ* 0.64 (s, 3H, CH_3_); 0.77 (s, 3H, CH_3_); 0.90 (d, 3H, *J* = 6.7 Hz, CH_3_); 1.22 (s, 3H, CH_3_); 1.26 (s, 6H, 2×CH3); 1.36 (s, 3H, CH3); 1.62–1.74 (m, 3H); 1.78–1.85 (m, 2H); 1.90–1.99 (m, 2H); 2.37–2.45 (m, 6H, 6×NCH); 3.52–3.55 (m, 4H, 4×CH2Omorph); 3.65–3.70 (m, 2H, CH2O, H-22); 3.80–3.91 (m, 4H, 3×CH2O, H-23); 4.02 (m, 1H, H-2β); 4.19 (m, 1H, H-3β). 13C NMR δ 11.55, 12.75, 13.15, 20.39, 21.44, 23.74, 26.62, 27.12, 27.18, 27.32, 28.64, 32.57, 35.22, 37.57, 40.65, 41.98, 42.42, 45.11, 52.13, 53.21, 54.04 (2×C), 55.10, 60.99, 63.67, 65.16, 66.23 (2×C), 72.02, 72.24, 75.05, 81.06, 106.75, 107.58, 108.87, 1×C n. f. HRMS: (API+) calculated for C_36_H_60_NO_7_ ([M + H]^+^) 618.4370, found 618.4376.

General procedure for deprotection of long side chain amine ketals 

A 5% aqueous solution of hydrochloric acid (1 mL) was added to a solution of ketals **21a**–**e** (20 mg; 0.03 mmol) in tetrahydrofuran (5 mL) and the mixture was stirred at 45 °C for 2 h. During the reaction, white crystals appeared. After cooling, solvents were evaporated under reduced pressure and the crude product was washed twice with ethyl acetate. The prepared salts obtained in quantitative yield had sufficient purity.

*N*,*N*-dimethyl-((*20S*,*22R*,*23S*)-2α,3α,22,23-tetrahydroxy-6-oxo-5α-cholan-24-yl)aminium chloride (**22a**)

Mp > 270 °C (decomp.). ^1^H NMR (CD_3_OD+D_2_O) *δ* 0.62 (s, 3H, CH_3_); 0.67 (s, 3H, CH_3_); 0.87 (d, 3H, *J* = 6.4 Hz, CH_3_); 1.85 (m, 1H); 1.95 (m, 1H); 2.07–2.16 (m, 2H); 2.65 (dd, 1H, *J* = 11.9 and 2.8 Hz); 3.00 (bt, 1H, *J* = 12.5 Hz, H-24a); 3.10 (dd, 1H, *J* = 12.5 and 2.9 Hz. H-24b); 3.38 (bd, 1H, *J* = 7.0 Hz, H-22); 3.64 (m, 1H, H-2β); 3.84 (ddd, 1H, *J* = 10.7, 7.5, and 2.9 Hz, H-23); 3.92 (m, 1H, H-3β). ^13^C NMR *δ* 12.30, 12.89, 13.90, 22.17, 24.69, 27.45, 28.47, 39.00, 39.31, 40.46 (2×C), 42.01, 43.82, 44.00, 45.89, 47.39, 52.10, 53.22, 54.56, 57.44, 60.28, 68.34, 69.00, 69.33, 75.87, 218.18. HRMS: (API+) calculated for C_26_H_46_NO_5_ ([M − Cl]^+^) 452.3376, found 452.3377. Anal. Calcd. for C_26_H_46_ClNO_5_: C, 63.98; H, 9.50; N, 2.87. Found: C, 63.95; H, 9.55; N, 2.85%.

*N*,*N*-diethyl-((*20S*,*22R*,*23S*)-2α,3α,22,23-tetrahydroxy-6-oxo-5α-cholan-24-yl)aminium chloride (**22b**)

Mp > 250 °C (decomp.). ^1^H NMR (CD_3_OD) *δ* 0.75 (s, 3H, CH_3_); 0.77 (s, 3H, CH_3_); 1.00 (d, 3H, *J* = 6.7 Hz, CH_3_); 1.33 (t, 3H, *J* = 7.3 Hz, CH_3_); 1.34 (t, 3H, *J* = 7.3 Hz, CH_3_); 1.99 (m, 1H); 2.05–2.14 (m, 2H); 2.22 (dd, 1H, *J* = 13.1 and 4.9 Hz); 2.73 (dd, 1H, *J* = 12.4 and 3.2 Hz); 3.05 (dd, 1H, *J* = 13.2 and 11.0 Hz, H-24a); 3.17 (dd, 1H, *J* = 13.2 and 3.2 Hz, H-24b); 3.21–3.37 (m, 4H, 4×NCH); 3.44 (dd, 1H, *J* = 6.7 and 1.4 Hz, H-22); 3.66 (ddd, 1H, *J* = 11.8, 4.8, and 3.2 Hz, H-2β); 3.87 (ddd, 1H, *J* = 11.1, 6.7, and 3.1 Hz, H-23); 3.95 (m, 1H, H-3β). ^13^C NMR *δ* 8.90, 9.39, 12.50, 13.20, 14.02, 22.51, 25.05, 27.99, 28.78, 39.33, 39.78, 41.04, 41.12, 43.76, 44.11, 47.61, 47.87, 50.30, 52.23, 53.72, 55.16, 55.35, 58.01, 68.59, 69.26, 69.63, 75.77, 215.17. HRMS: (API+) calculated for C_28_H_50_NO_5_ ([M − Cl]^+^) 480.3689, found 480.3689. Anal. Calcd. for C_28_H_50_ClNO_5_: C, 65.15; H, 9.76; N, 2.71. Found: C, 65.09; H, 9.81; N, 2.68%.

1-((*20S*,*22R*,*23S*)-2α,3α,22,23-tetrahydroxy-6-oxo-5α-cholan-24-yl)pyrrolidinium chloride (**22c**)

Mp > 250 °C (decomp.). ^1^H NMR (CD_3_OD+D_2_O) *δ* 0.63 (s, 3H, CH_3_); 0.67 (s, 3H, CH_3_); 0.88 (d, 3H, *J* = 6.4 Hz, CH_3_); 1.86 (m, 1H); 1.93–1.99 (m, 3H); 2.05–2.16 (m, 3H); 2.66 (dd, 1H, *J* = 12.4 and 3.0 Hz); 2.97–3.15 (m, 4H, 4×NCH); 3.39 (bd, 1H, *J* = 7.0 Hz, H-22); 3.58–3.66 (m, 3H, 2×NCH, H-2β); 3.81 (m, H-23); 3.92 (m, 1H, H-3β). ^13^C NMR *δ* 12.32, 12.95, 13.90, 22.19, 23.78, 23.96, 24.72, 27.49, 28.51, 39.13, 39.31, 40.52, 43.85, 43.98, 47.39, 49.78, 52.12, 53.30, 54.08, 54.61, 56.83, 57.50, 58.04, 69.03, 69.37, 69.73, 75.79, 217.93. HRMS: (API+) calculated for C_28_H_48_NO_5_ ([M − Cl]^+^) 478.3532, found 478.3535. Anal. Calcd. for C_28_H_48_ClNO_5_: C, 65.41; H, 9.41; N, 2.72. Found: C, 65.36; H, 9.48; N, 2.69%.

1-((*20S*,*22R*,*23S*)-2α,3α,22,23-tetrahydroxy-6-oxo-5α-cholan-24-yl)piperidinium chloride (**22d**)

Mp > 250 °C (decomp.). ^1^H NMR (CD_3_OD) *δ* 0.74 (s, 3H, CH_3_); 0.76 (s, 3H, CH_3_); 0.99 (d, 3H, *J* = 6.7 Hz, CH_3_); 1.93–2.02 (m, 2H); 2.05–2.14 (m, 2H); 2.20 (dd, 1H, *J* = 13.4 and 4.8 Hz); 2.73 (dd, 1H, *J* = 12.2 and 3.1 Hz); 2.92 (td, 1H, *J* = 12.0 and 2.9 Hz, NCH); 3.01 (dd, 1H, *J* = 12.9 and 11.5 Hz, H-24a); 3.05 (td, 1H, *J* = 12.0 and 4.4 Hz, NCH); 3.15 (dd, 1H, *J* = 12.9 and 2.4 Hz, H-24b); 3.41 (dd, 1H, *J* = 6.9 and 1.4 Hz, H-22); 3.49 (bd, 1H, *J* = 11.9 Hz, NCH); 3.62–3.68 (m, 2H, NCH, H-2β); 3.92 (m, 1H, H-23); 3.95 (m, 1H, H-3β). ^13^C NMR *δ* 12.48, 13.05, 14.01, 22.50, 22.89, 24.03, 24.08, 25.03, 27.99, 28.80, 39.32, 39.68, 41.03, 41.12, 43.76, 44.11, 47.60, 52.24, 52.74, 53.64, 55.16, 56.36, 57.96, 60.09, 67.88, 69.26, 69.62, 75.86, 215.24. HRMS: (API+) calculated for C_28_H_48_NO_6_ ([M − Cl]^+^) 494.3482, found 494.3485. Anal. Calcd. for C_29_H_50_ClNO_5_: C, 65.95; H, 9.54; N, 2.65. Found: C, 65.89; H, 9.60; N, 2.63%.

4-((*20S*,*22R*,*23S*)-2α,3α,22,23-tetrahydroxy-6-oxo-5α-cholan-24-yl)morpholinium chloride (**22e**)

Mp > 270 °C (decomp.). ^1^H NMR (CD_3_OD+D_2_O) *δ* 0.59 (s, 3H, CH_3_); 0.64 (s, 3H, CH_3_); 0.84 (d, 3H, *J* = 6.7 Hz, CH_3_); 1.71 (qd, 1H, *J* = 10.3 and 7.2 Hz); 1.79 (m, 1H); 1.92 (m, 1H); 2.07–2.13 (m, 2H); 2.63 (dd, 1H, *J* = 11.6 and 4.3 Hz); 3.03 (dd, 1H, *J* = 13.0 and 11.3 Hz, H-24a); 3.08 (m, 1H, NCH); 3.14 (dd, 1H, *J* = 13.0 and 3.1 Hz, H-24b); 3.18 (m, 1H, NCH); 3.38 (dd, 1H, *J* = 6.9 and 1.2 Hz, H-22); 3.39 (m, 1H, NCH); 3.52 (bd, 1H, *J* = 12.2 Hz, NCH); 3.64 (m, 1H, H-2β); 3.73–3.81 (m, 2H, CH-O); 3.89–3.94 (m, 2H, H-3β, H-23); 3.96–4.02 (m, 2H, CH-O). ^13^C NMR *δ* 12.25, 12.84, 13.85, 22.05, 24.56, 27.31, 28.37, 38.79, 39.28, 40.27, 40.30, 43.73, 44.09, 47.32, 51.57, 52.05, 53.11, 54.37, 54.53, 57.26, 59.87, 64.66 (2×C), 67.44, 68.90, 69.26, 75.89, 219.27. HRMS: (API+) calculated for C_28_H_48_NO_6_ ([M − Cl]^+^) 494.3482, found 494.3482. Anal. Calcd. for C_28_H_48_ClNO_6_: C, 63.44; H, 9.13; N, 2.64. Found: C, 63.40; H, 9.18; N, 2.61%.

4-((*20S*,*22R*,*23S*)-2α,3α-dihydroxy-22,23-(1-methylethylidene)dioxy-6-oxo-5α-cholan-24-yl) morpholinium chloride (**24**)

The product was obtained during optimization of the reaction condition (deprotection of long side chain amine ketals at room temperature) as white crystals. 

Mp > 210 °C (decomp.). ^1^H NMR (D_2_O) *δ* 0.63 (s, 3H, CH_3_); 0.70 (s, 3H, CH_3_); 0.95 (d, 3H, *J* = 6.7 Hz, CH_3_); 1.37 (s, 3H, CH_3_); 1.38 (s, 3H, CH_3_); 1.77–1.90 (m, 2H); 1.98 (m, 1H); 2.20 (d, 2H, *J* = 8.3 Hz); 2.69 (dd, 1H, *J* = 11.2 and 5.0 Hz); 3.10–3.63 (vb, 4H, 4×NCH); 3.30 (dd, 1H, *J* = 13.6 and 1.9 Hz, H-24a); 3.35 (dd, 1H, *J* = 13.6 and 9.5 Hz, H-24b); 3.73 (m, 1H, H-2β); 3.78–4.13 (vb, 4H, 4×CH-O); 3.94 (d, 1H, *J* = 8.3 Hz, H-22); 3.98 (m, 1H, H-3β); 4.22 (m, 1H, H-23). 13C NMR δ 10.91, 12.09, 12.72, 20.82, 23.32, 25.93, 25.94, 26.02, 27.21, 35.11, 38.17, 38.85, 38.92, 42.75, 43.09, 46.14, 50.88, 52.77 (2×C), 53.01 (2×C), 55.78, 59.34, 63.64 (2×C), 67.74, 68.12, 71.64, 81.16, 110.75, 219.66. HRMS: (API+) calculated for C_31_H_52_NO_6_ ([M − Cl]^+^) 534.3795, found 534.3796.

### 3.3. Plant Bioassay 

The biological activity of the new BRs derivatives was tested by an Arabidopsis sensitivity bioassay which was performed according to [28]. Briefly, seedlings of Arabidopsis thaliana L. (Columbia ecotype, Col-0) were stratified for 2 days at 4 °C and germinated on vertical half-strength Murashige and Skoog (1% *w*/*v* sucrose) agar at 22 °C in a 16 h/8 h light–dark cycle. The plates contained concentrations of 24-epiBL (as positive control) and BR derivatives ranging from 0.1 to 10 nM, respectively 100 nM (for selected derivatives only). After 7 days, roots were straightened on solid media plates and scanned with an Epson high-resolution scanner. The entire root length was measured with ImageJ (http://rsbweb.nih.gov/ij/). For each treatment, more than 15 seedlings were analyzed in two biological repeats, and p-values were calculated with the two-tailed Student t-test using Excel software.

### 3.4. Cell Cultures and Cytotoxicity Assay

The screening cell lines: T-lymphoblastic leukemia CEM; breast carcinoma MCF7 (estrogen-sensitive); cervical carcinoma cell line HeLa; and human foreskin fibroblasts BJ, were obtained from the American Type Culture Collection (Manassas, VA, USA). Cells were cultured in DMEM (Dulbecco’s Modified Eagle Medium, Sigma, St. Louis, MO, USA). Media used were supplemented with 10% fetal bovine serum, 2mM L-glutamine and 1% penicillin–streptomycin. The cell lines were maintained under standard cell culture conditions at 37 °C and 5% CO2 in a humid environment. 

Cells were subcultured twice or three times a week using the standard trypsinization procedure. Cytotoxicity in cancer cell lines after 72 h was determined as described earlier [43]. Control cultures were treated with DMSO alone, and the final concentration of DMSO in the mixture never exceeded 0.6%. Six serial 3-fold dilutions of the test substances were added at time zero in 20 μL aliquots to the microtiter plate wells and the highest final concentration in the wells was 50 μM. 

### 3.5. Molcular Docking

We used AutoDock Vina 1.05 [44] in this study of Brassinosteroid receptor BRI1 (PDBID: 3RGZ). Datasets of 3D structures of brassinolide derivatives were prepared using Marvin 15.1.5.0, a software used for the drawing, displaying and characterization of chemical structures, substructures and reactions. AutoDock Tools [45] (ADT) was used for preparation of input data for molecular docking.

## 4. Conclusions

In this study, 25 brassinosteroid analogues with a nitrogen-containing side chain were synthesized based on molecular docking and tested using an Arabidopsis sensitivity bioassay and Calcein AM cytotoxicity assay. New analogues were divided into two groups—short side chain BRs and long side chain BRs. As a nitrogen-containing functional group, tertiary amides, tertiary amines and ammonium chlorides were prepared. The synthesized substances showed no significant inhibitory activity compared to natural 24-epibrassinolide. In contrast, several compounds showed interesting growth-promoting activity. The cytotoxicity assay showed no toxicity of the prepared compounds on cancer or normal cell lines. Unfortunately, the results of the molecular docking, which proved the good binding affinity of the tested compounds to the BRI1 receptor, were not confirmed by plant experiments. However, the very low or no biological activity of nitrogen derivatives can also be caused by different reasons other than low affinity to the receptor, e.g., by an unknown mechanism that prevents transport of these nitrogen compounds to the receptor. Based on these observations, it is possible to state that the presence of an amide or amine in the side chain of BRs analogues significantly reduces the biological activity in plants.

## Data Availability

Samples of the compounds are not available from the authors.

## Supplementary Materials

Supplementary materials can be found at https://www.mdpi.com/1422-0067/22/1/155/s1. The following are available online. Table S1: Resulting binding free energies obtained for molecular docking experiments. Figures S1–S20: Poses of brassinolide and new nitrogen analogues within the BRI1 binding site. Figure S21: Plant bioassay with concentration of 100 nmol/L. Table S2: Data for Figure 2, Figure 3 and Figure S21. Table S3: Cytotoxicity data. NMR/HR-MS spectra of all new compounds.
