# Supplementary material for: Synthesis and Biological Activity of Brassinosteroid Analogues with a Nitrogen-Containing Side Chain
Source: Int J Mol Sci. 2020 Dec 25;22(1):155. doi: 10.3390/ijms22010155 (PMC7795425; doi:10.3390/ijms22010155)
Supplement: Supplementary file 1 [file ijms-22-00155-s001.pdf]

Supplementary Materials

Synthesis and biological activity of brassinosteroid analogues with a nitrogen-containing side chain

Mikhail V. Diachkov [1], Karoll Ferrer [1], Jana Oklestkova [1], Lucie Rarova [1], Vaclav Bazgier [1,2], and Miroslav Kvasnica [1,*]

[1] Laboratory of Growth Regulators, The Czech Academy of Sciences, Institute of Experimental Botany & Palacký University, Šlechtitelů 27, 78371 Olomouc, Czech Republic;
[2] Department of Physical Chemistry, Faculty of Science, Palacký University Olomouc, Šlechtitelů 241/27, 77900 Olomouc, Czech Republic

* Correspondence: kvasnica@ueb.cas.cz; Tel.: +420-585-639-446

Table of Contents

1) Docking studies
2) Plant bioassay with concentration of 100 nmol/L and data for figures 2, 3, and S21
3) Cytotoxicity
4) ^{1}H and ^{13}C NMR and MS spectra of new compounds

1) Docking studies

Table S1: Resulting binding free energies ($\Delta G_{bind\ best}$) for best crystal-like poses (i.e. similar position with natural ligand – brassinolide). Binding energies for castasterone and brassinolide used for comparison.

Compound #	Compound structure	Energy (kcal/mol)
castasterone	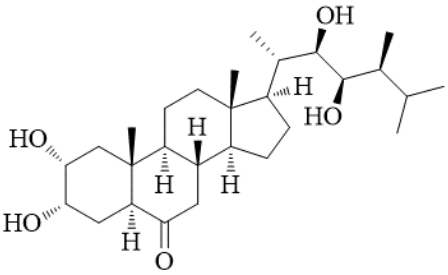	**-10,2**
brassinolide	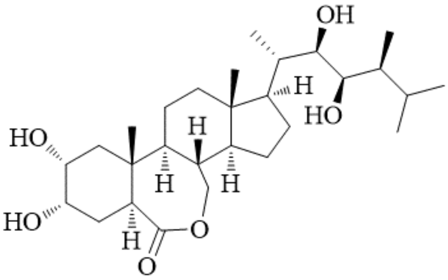	**-10,6**
8d	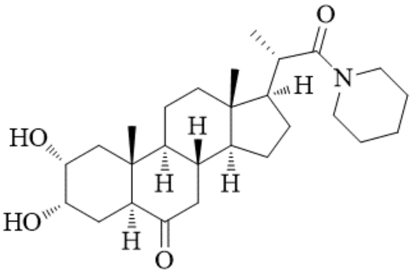	-11,8
8c	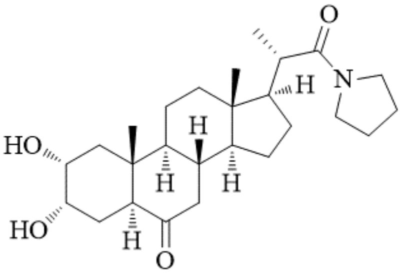	-11,2
8e	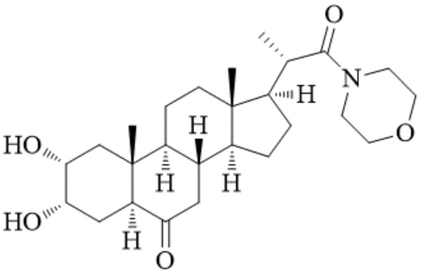	-11,1

15d	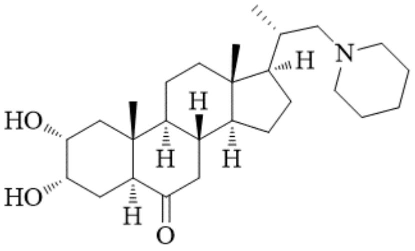	-11,1
20c	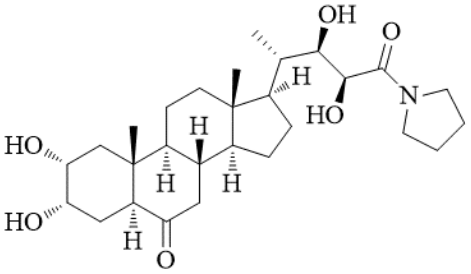	-11,1
22d (not protonated)	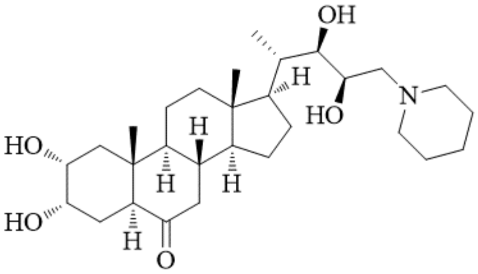	-11
22c (not protonated)	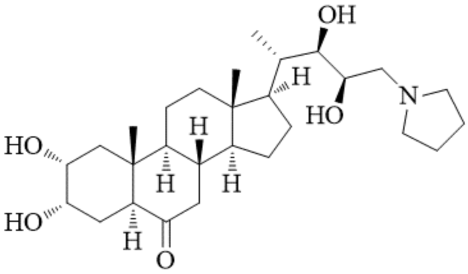	-10,8
22e (not protonated)	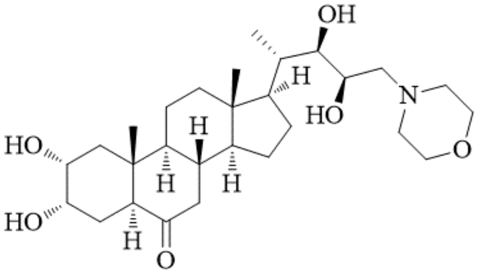	-10,5
20b	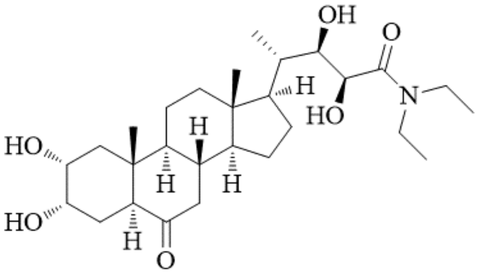	-10,5

8a	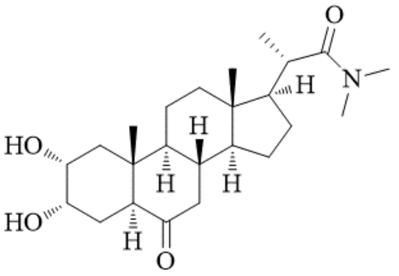	-10,3
15e	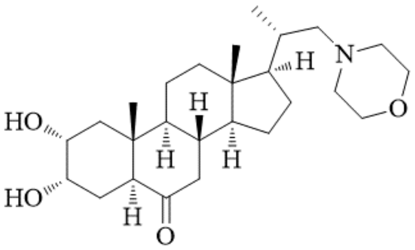	-10,2
20a	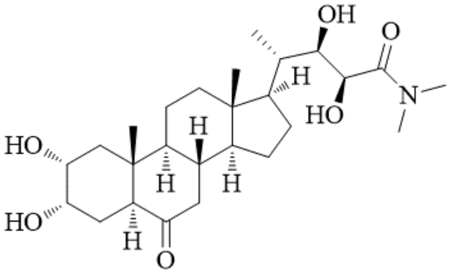	-10,2
15c	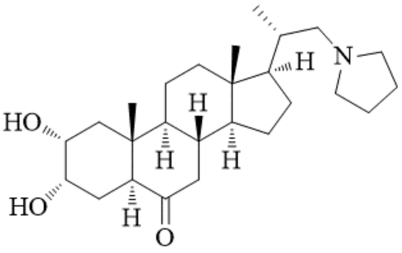	-10,2
8b	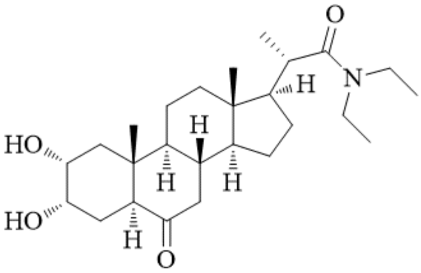	-10,1
20d	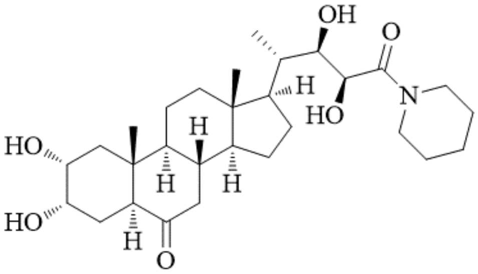	-9,8

15b	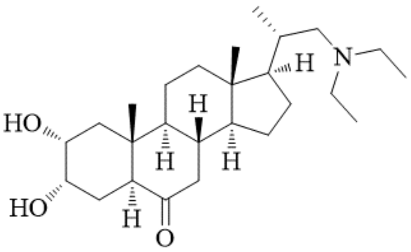	-9,8
20e	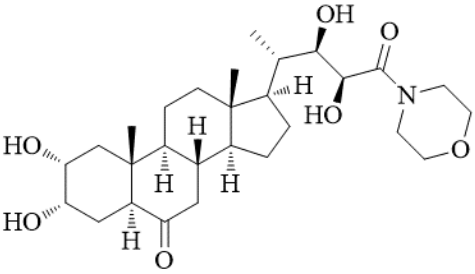	-9,8
22a (not protonated)	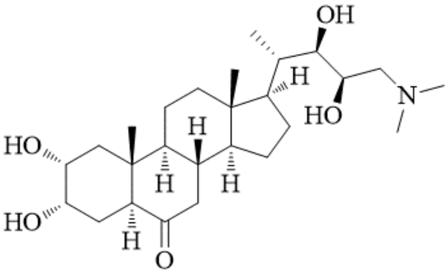	-9,8
15a	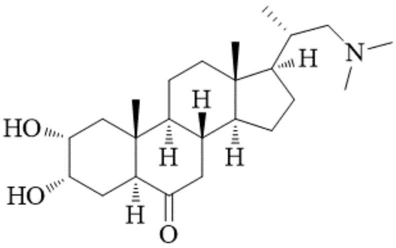	-9,5
22b (not protonated)	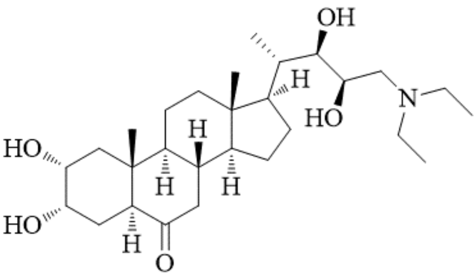	-8,8

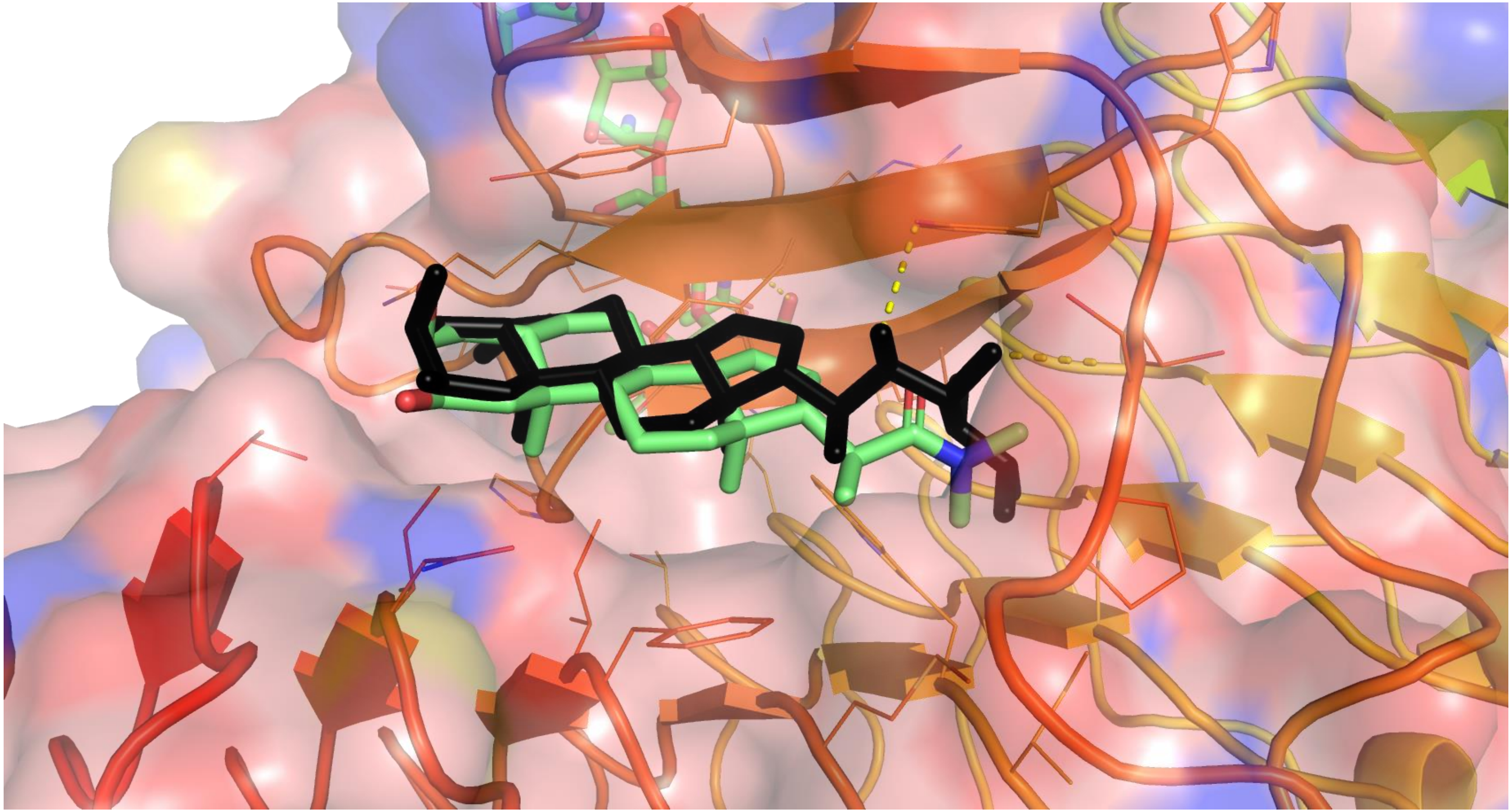

Fig. S1. Poses of brassinolide (black) and new nitrogen analogue **8a** within the BRI1 binding site.

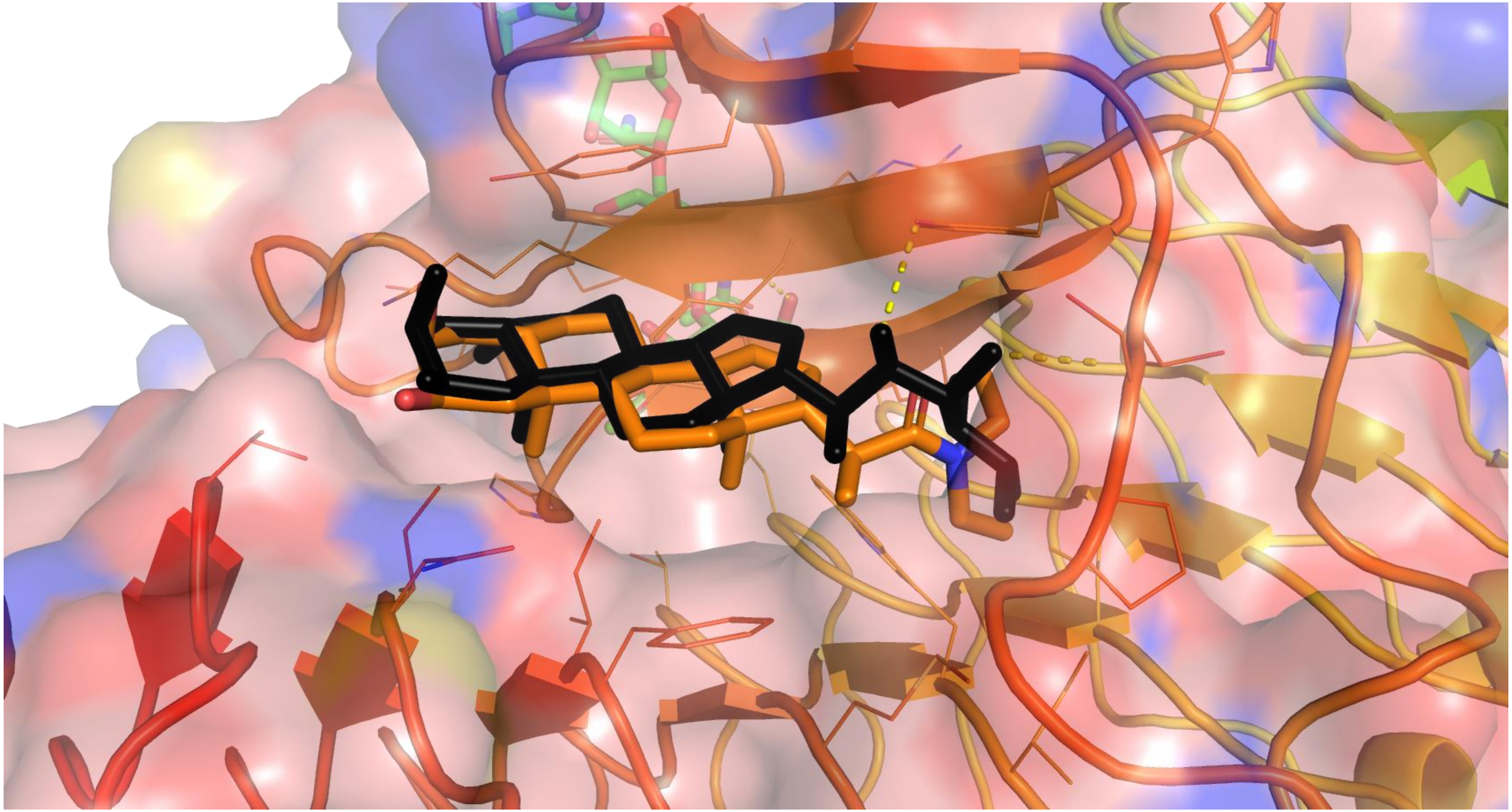

Fig. S2. Poses of brassinolide (black) and new nitrogen analogue **8b** within the BRI1 binding site.

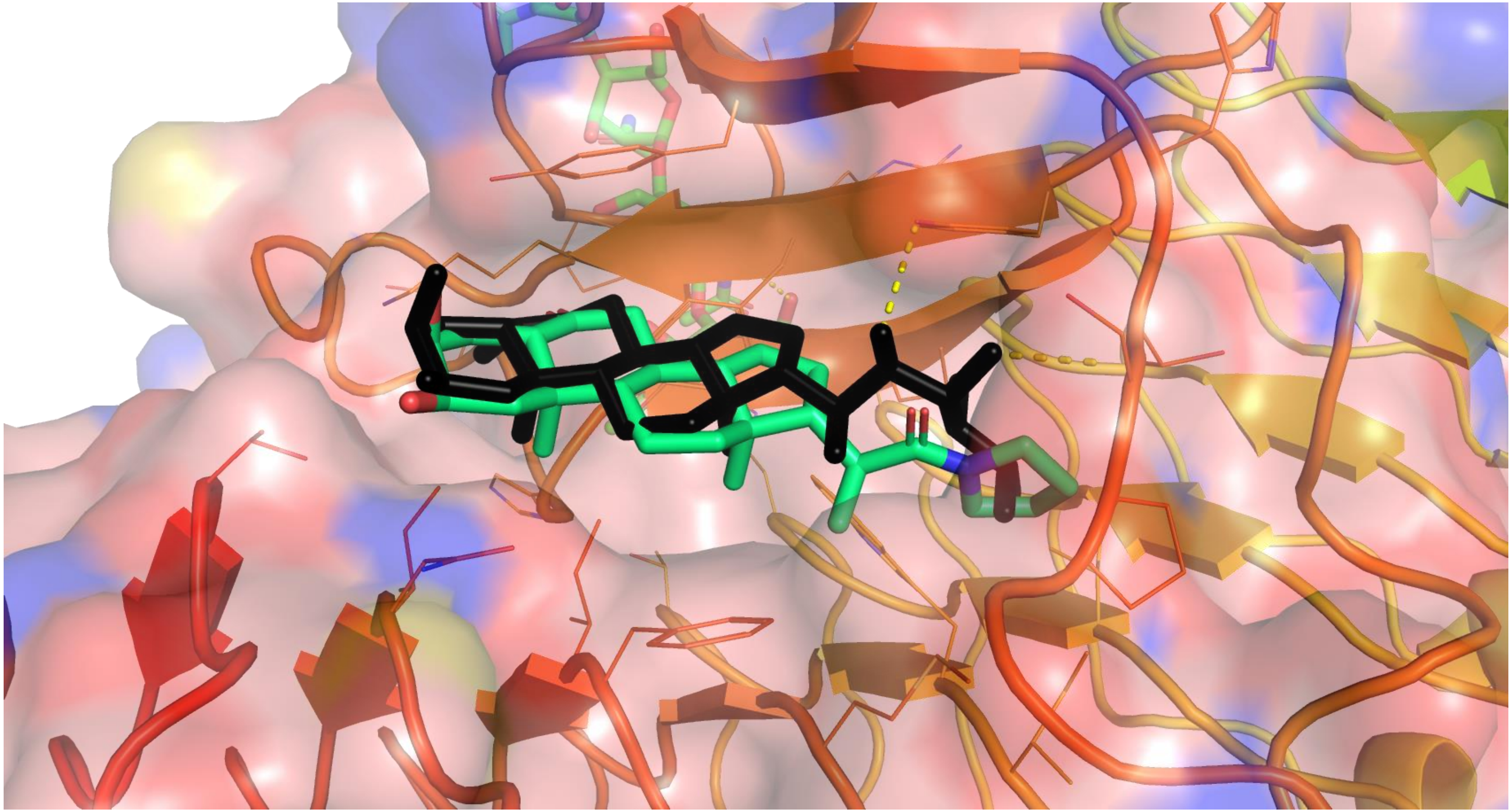

Fig. S3. Poses of brassinolide (black) and new nitrogen analogue **8c** within the BRI1 binding site.

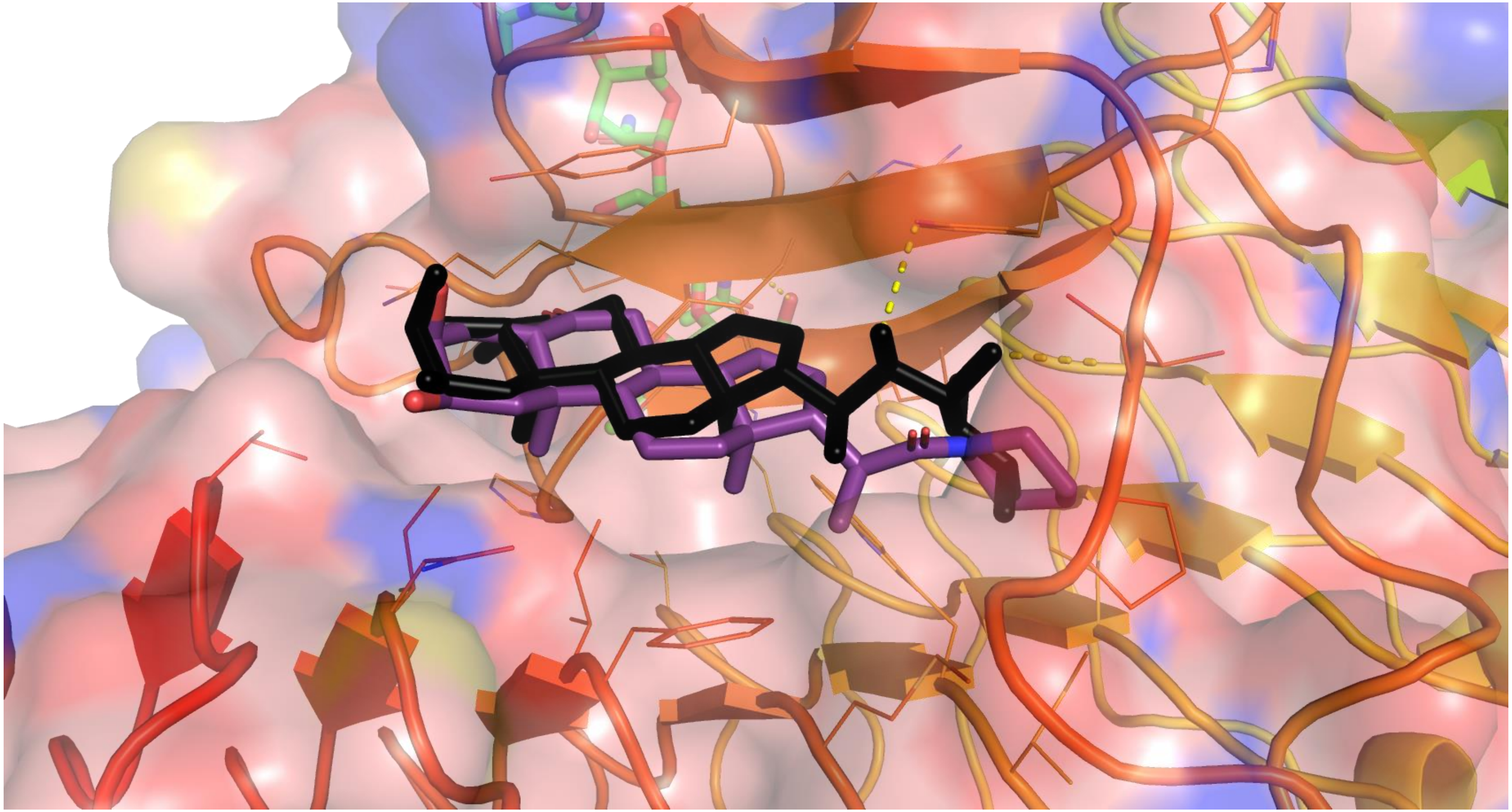

Fig. S4. Poses of brassinolide (black) and new nitrogen analogue **8d** within the BRI1 binding site.

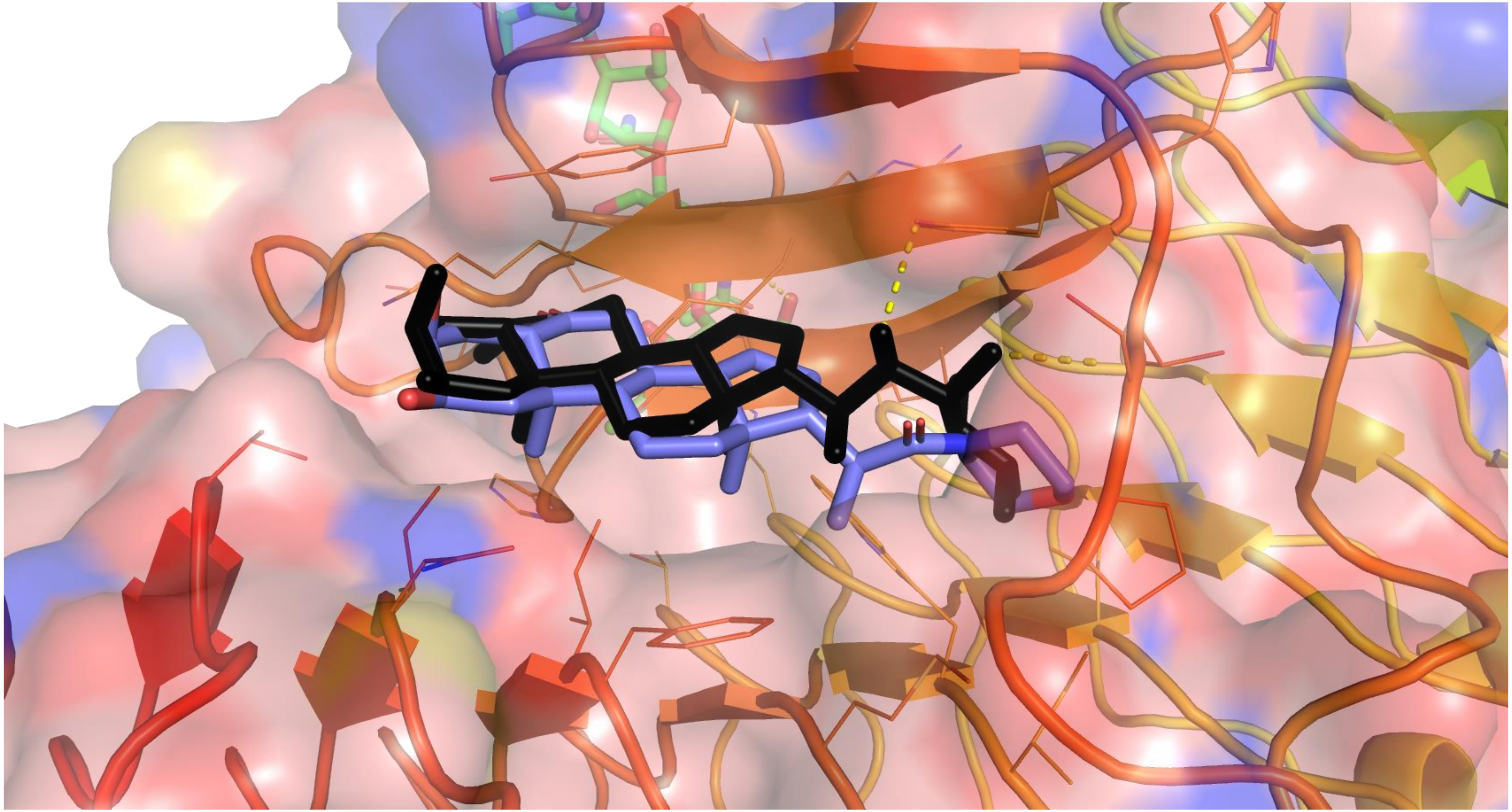

Fig. S5. Poses of brassinolide (black) and new nitrogen analogue **8e** within the BRI1 binding site.

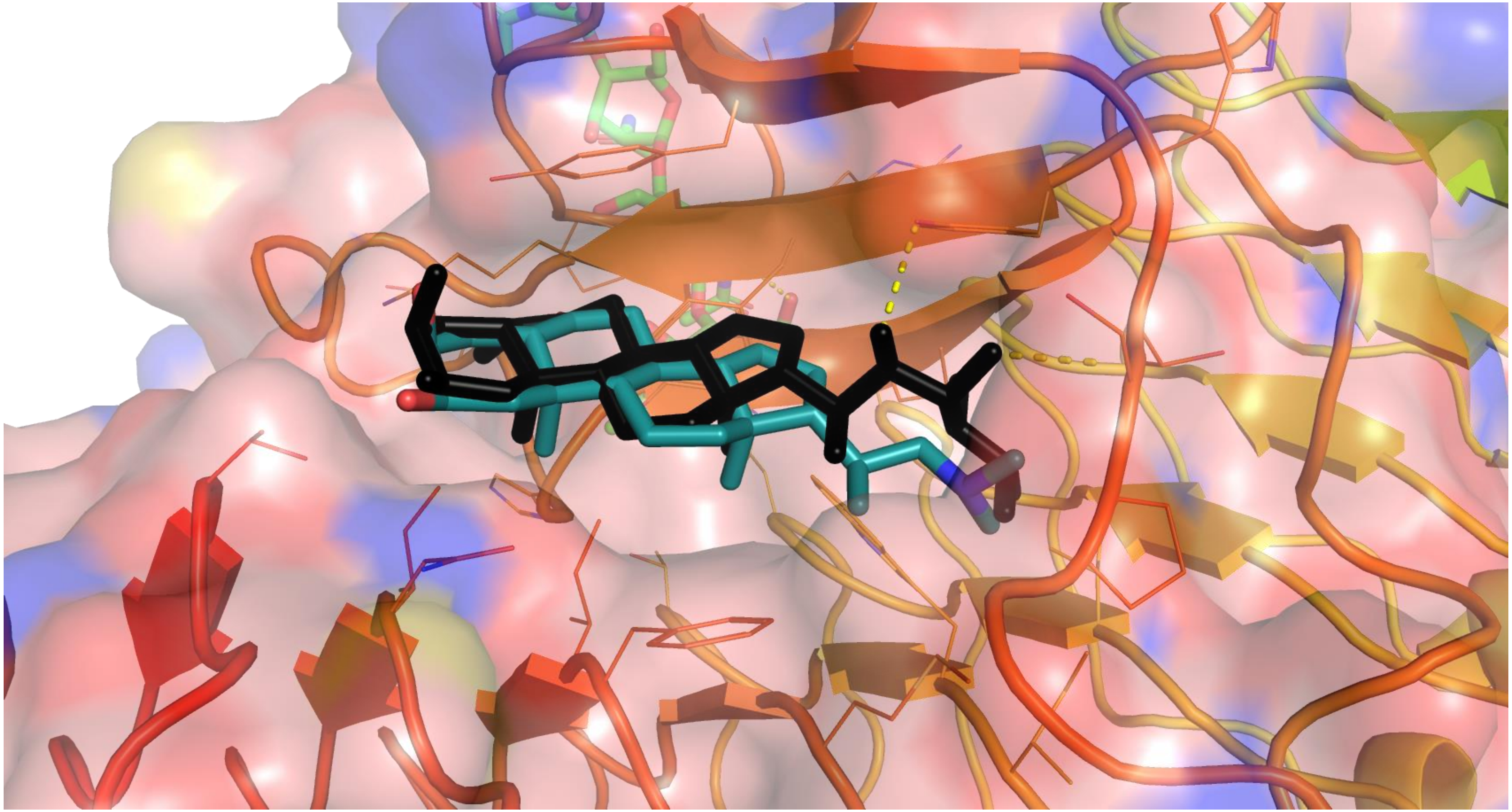

Fig. S6. Poses of brassinolide (black) and new nitrogen analogue **15a** within the BRI1 binding site.

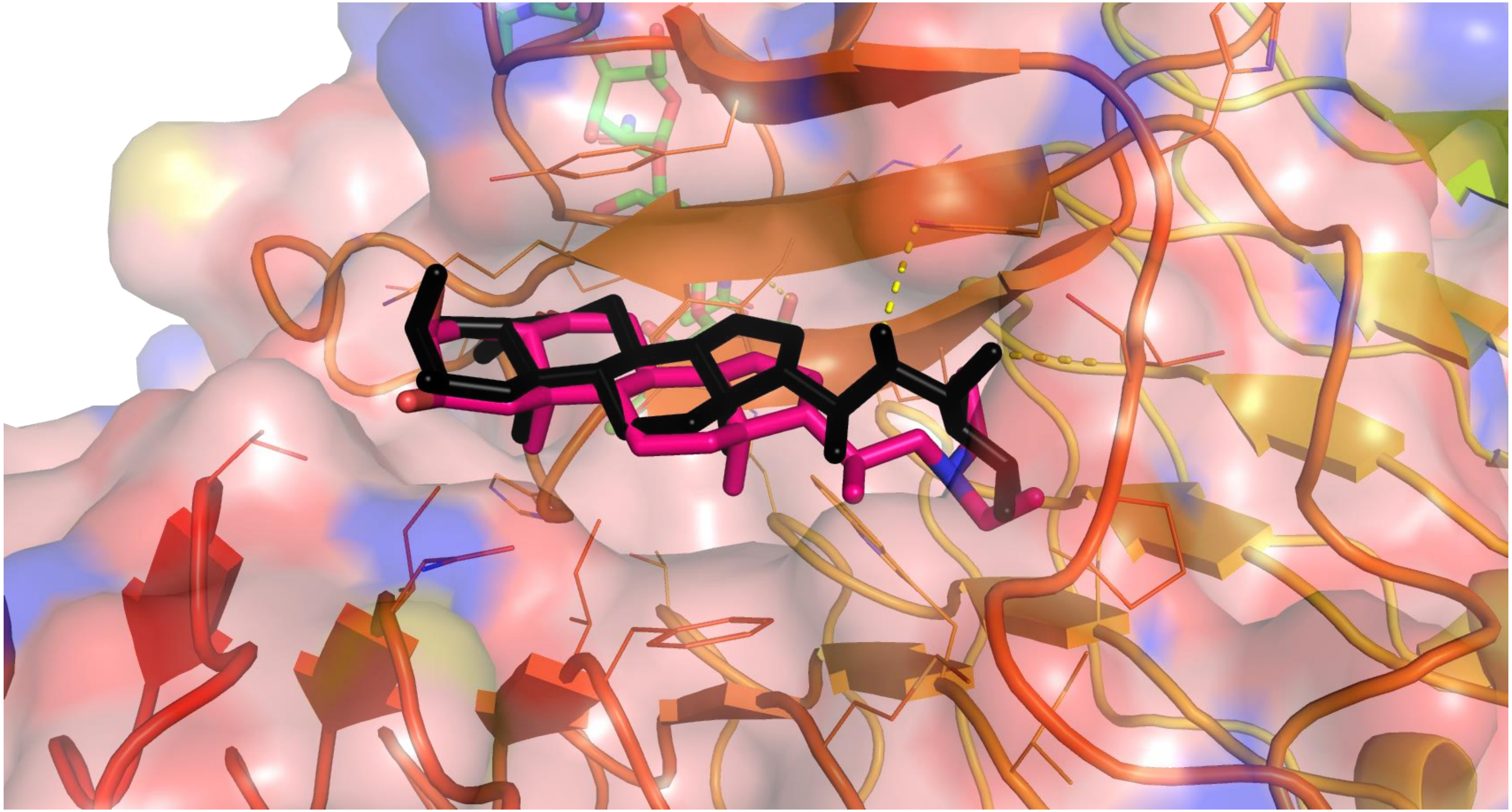

Fig. S7. Poses of brassinolide (black) and new nitrogen analogue **15b** within the BRI1 binding site.

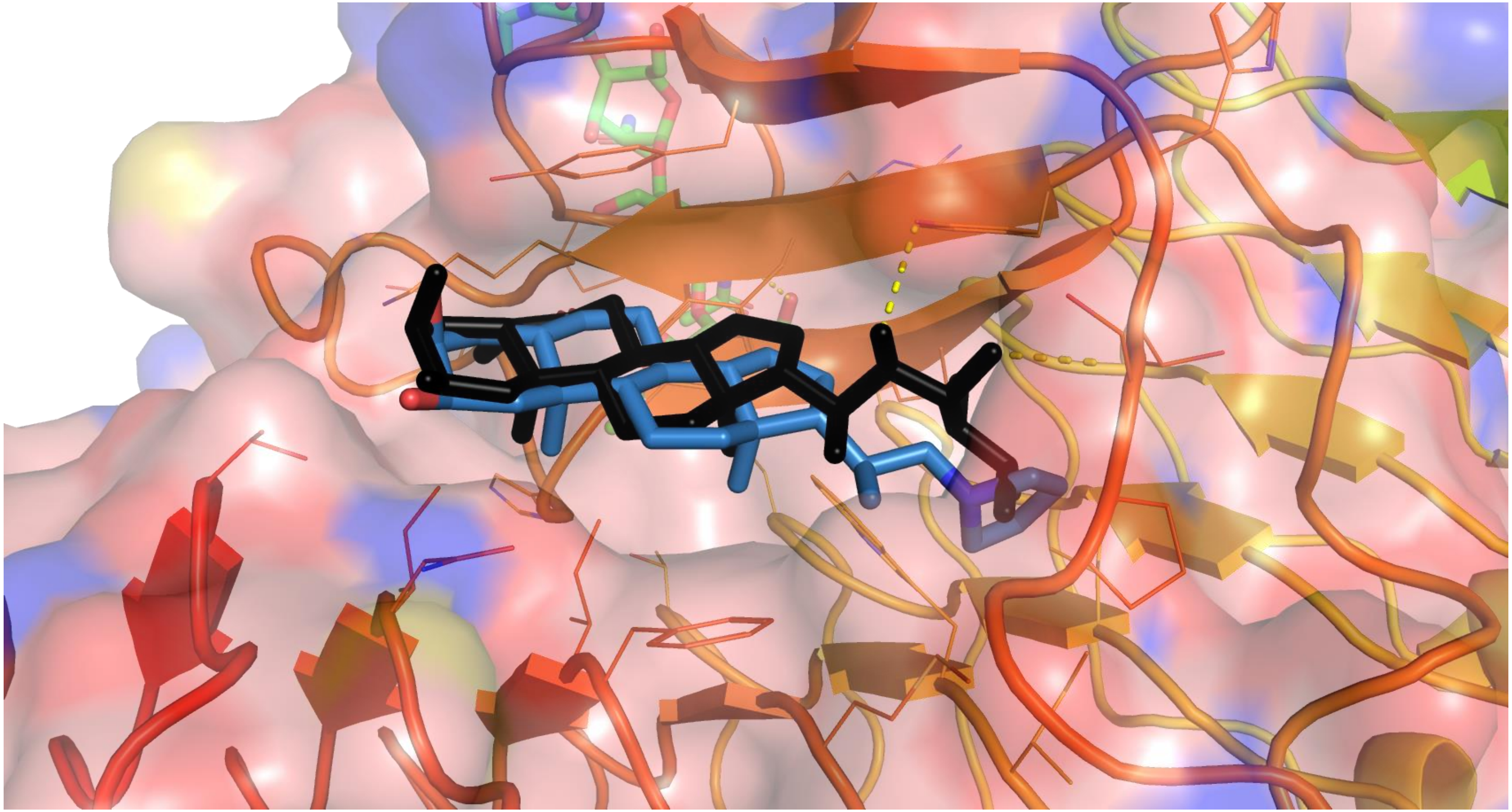

Fig. S8. Poses of brassinolide (black) and new nitrogen analogue **15c** within the BRI1 binding site.

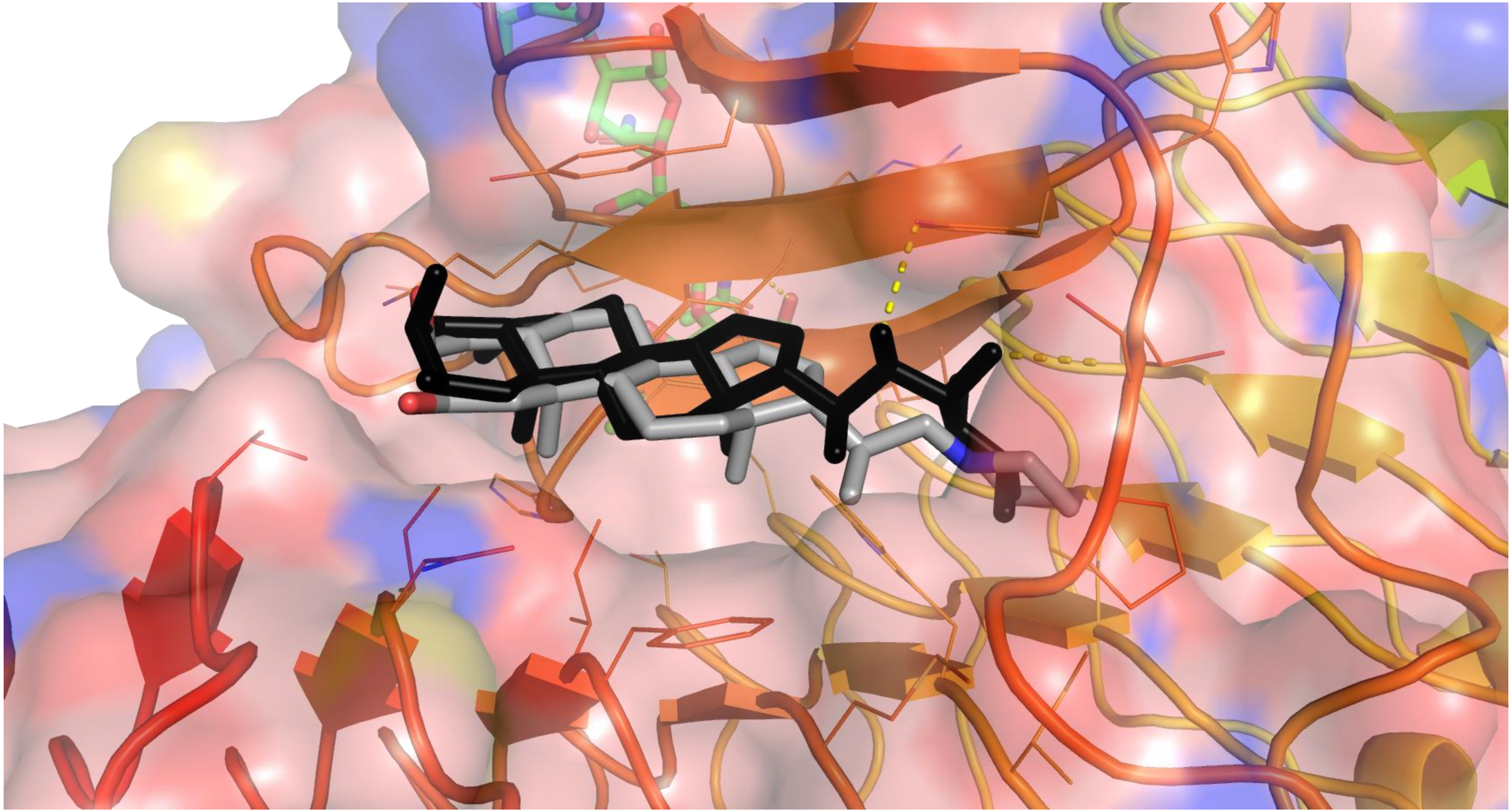

Fig. S9. Poses of brassinolide (black) and new nitrogen analogue **15d** within the BRI1 binding site.

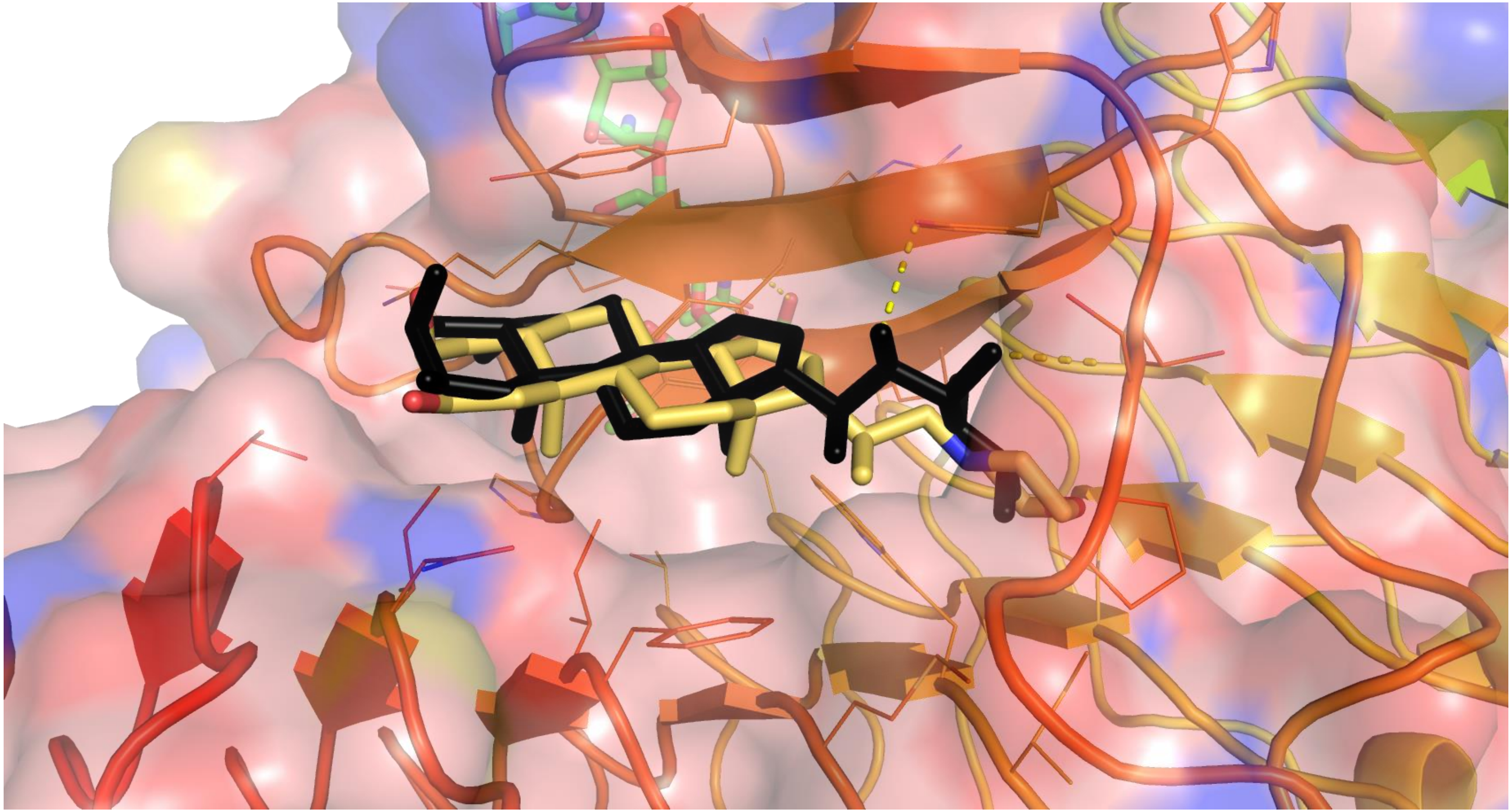

Fig. S10. Poses of brassinolide (black) and new nitrogen analogue **15e** within the BRI1 binding site.

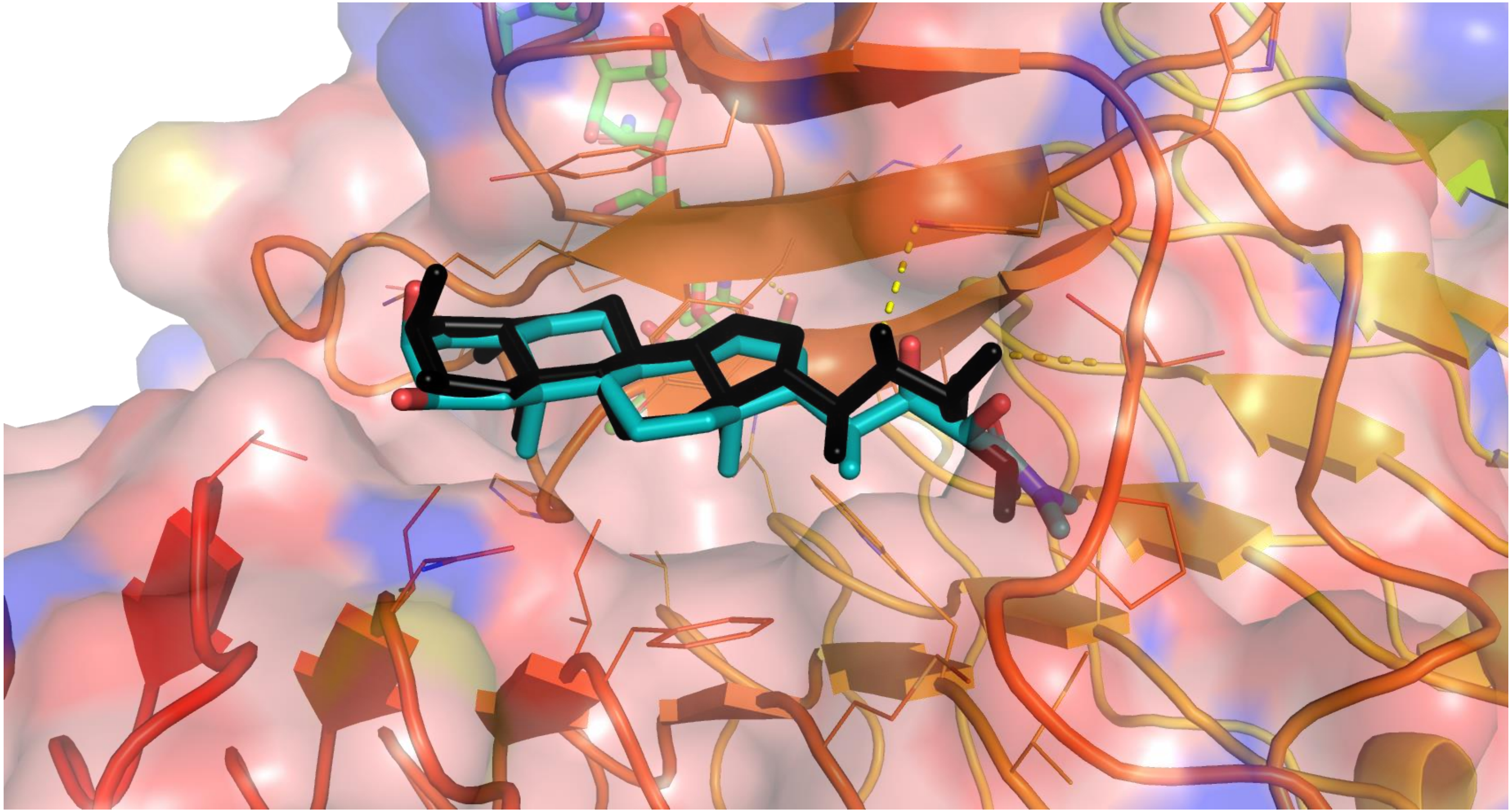

Fig. S11. Poses of brassinolide (black) and new nitrogen analogue **20a** within the BRI1 binding site.

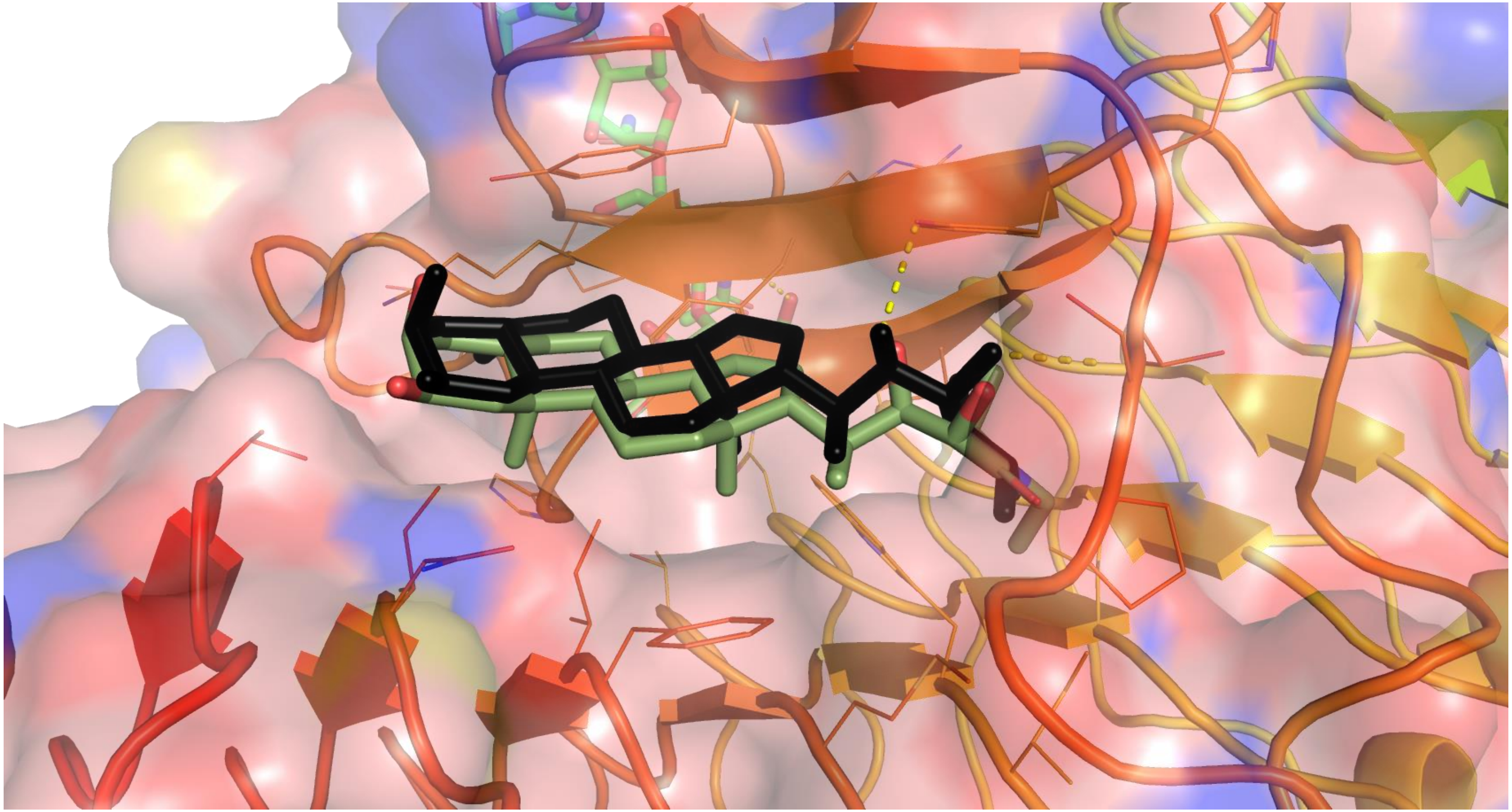

Fig. S12. Poses of brassinolide (black) and new nitrogen analogue **20b** within the BRI1 binding site.

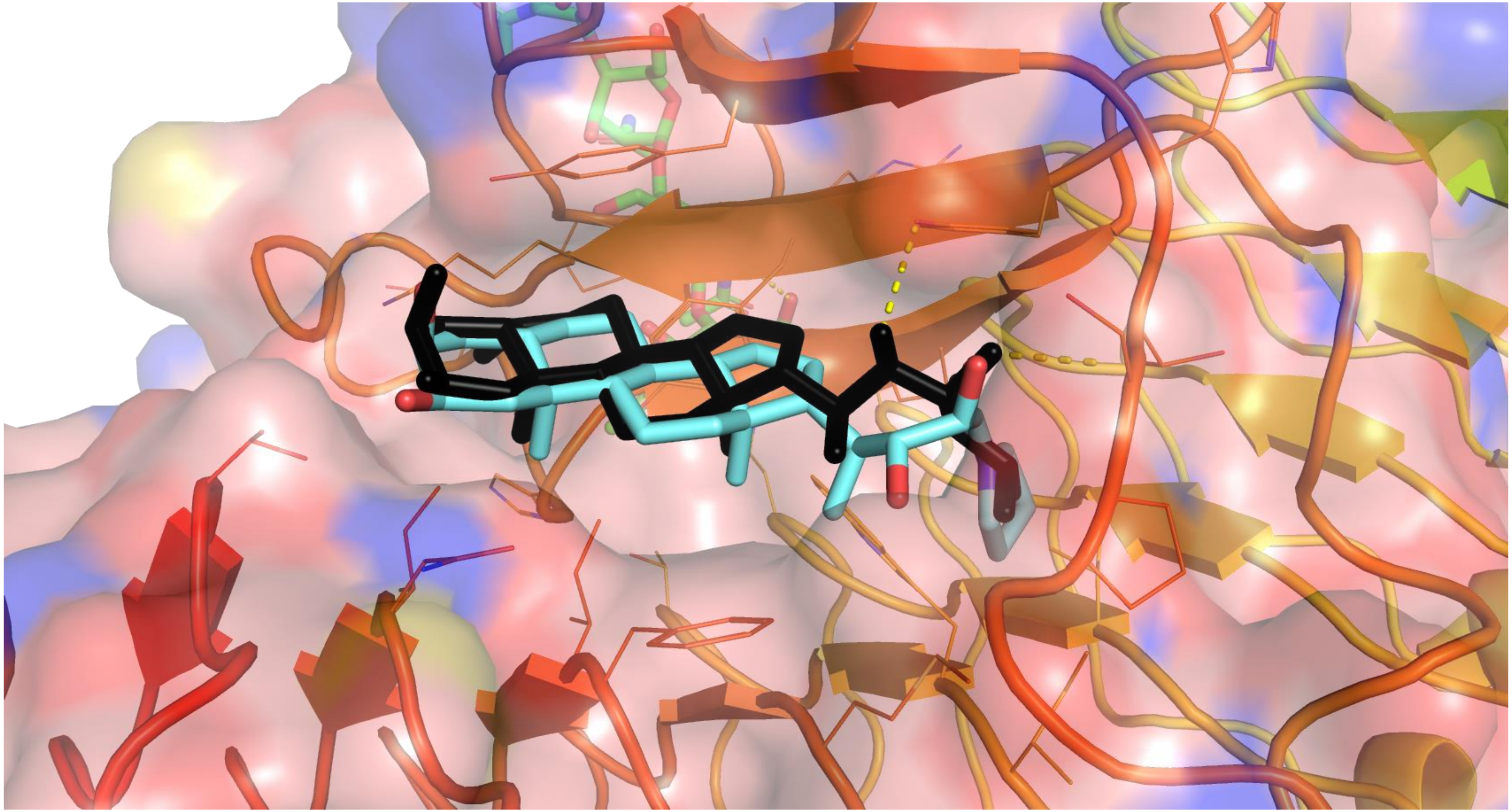

Fig. S13. Poses of brassinolide (black) and new nitrogen analogue **20c** within the BRI1 binding site.

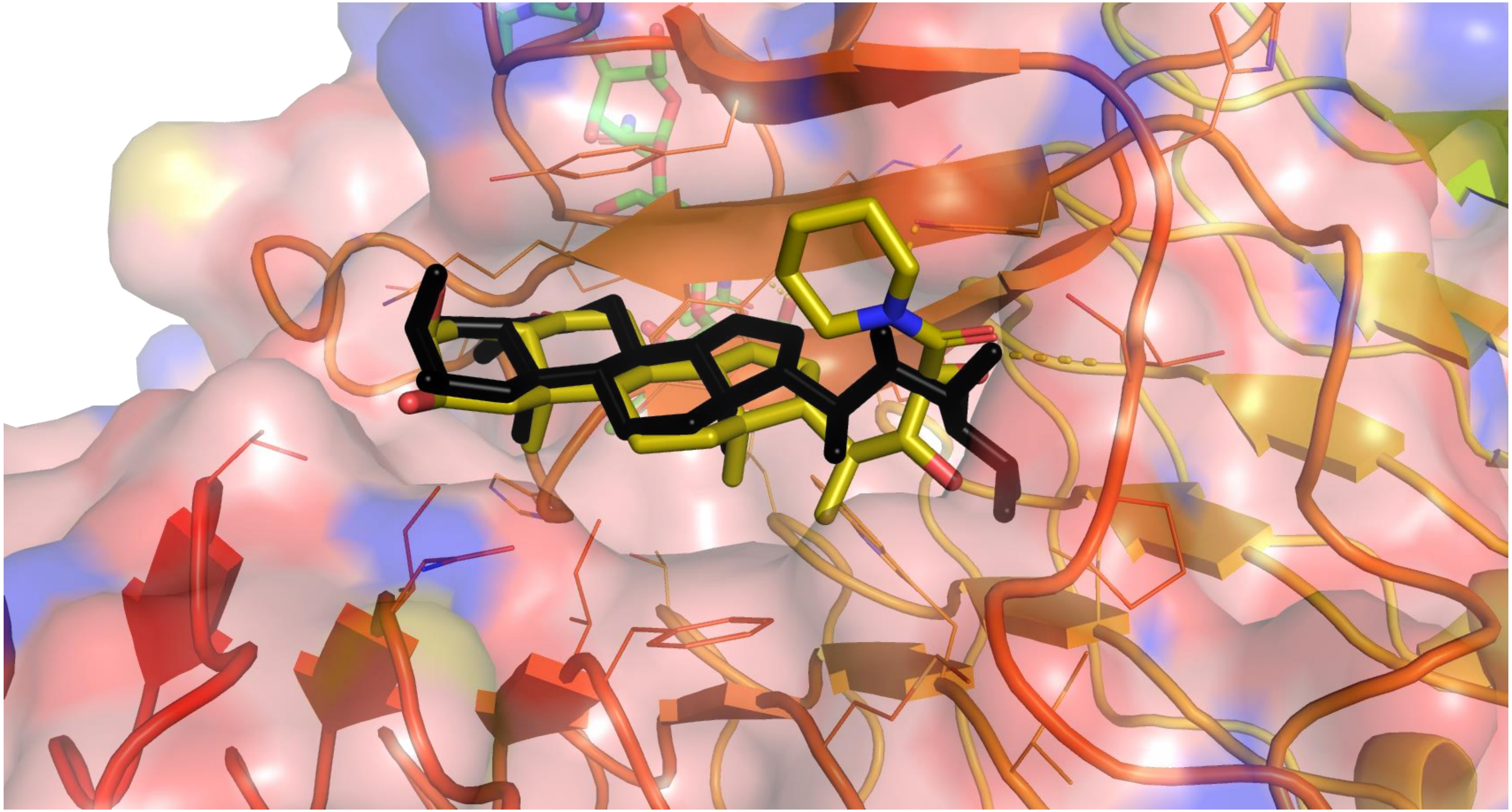

Fig. S14. Poses of brassinolide (black) and new nitrogen analogue **20d** within the BRI1 binding site.

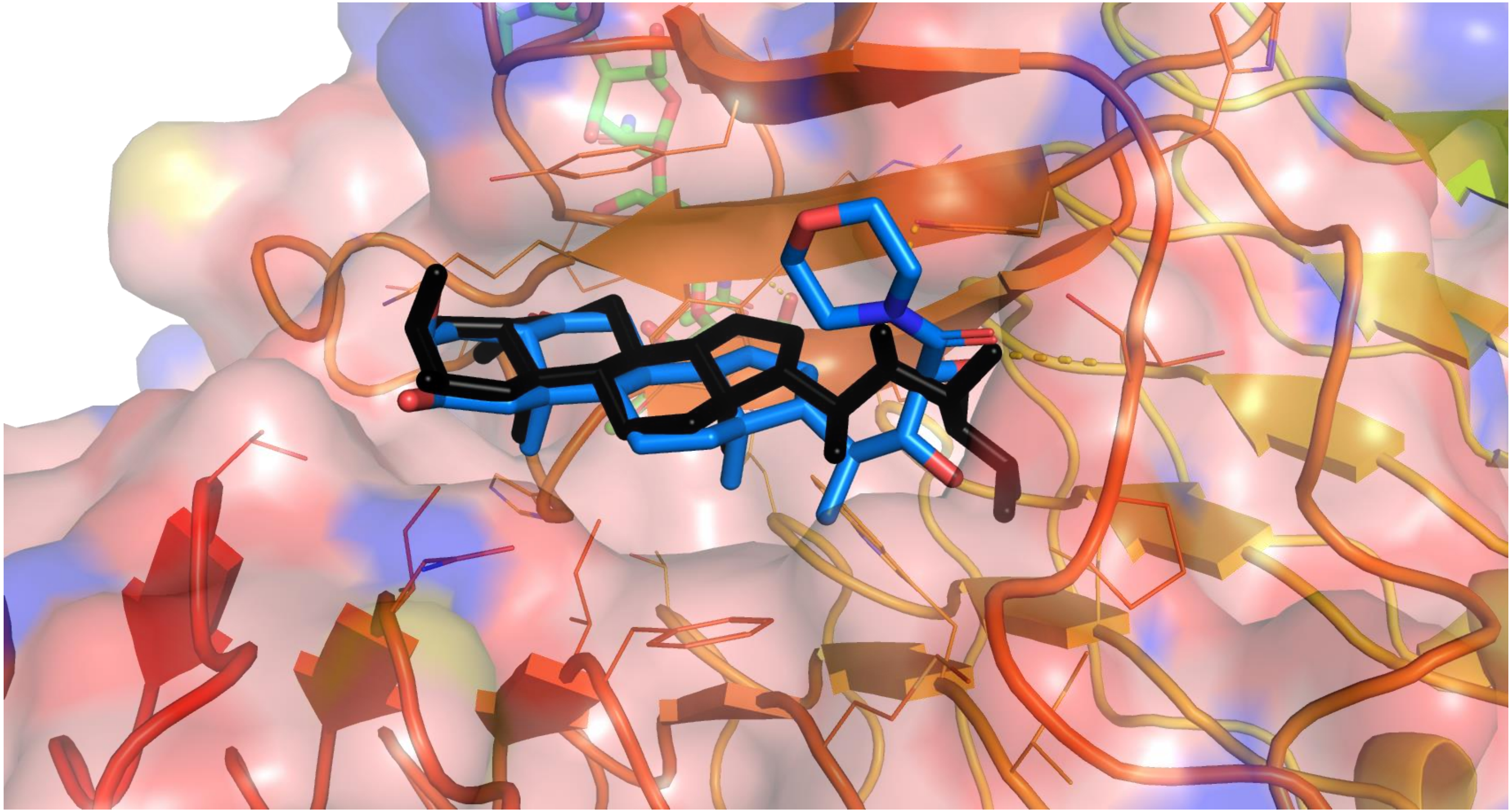

Fig. S15. Poses of brassinolide (black) and new nitrogen analogue **20e** within the BRI1 binding site.

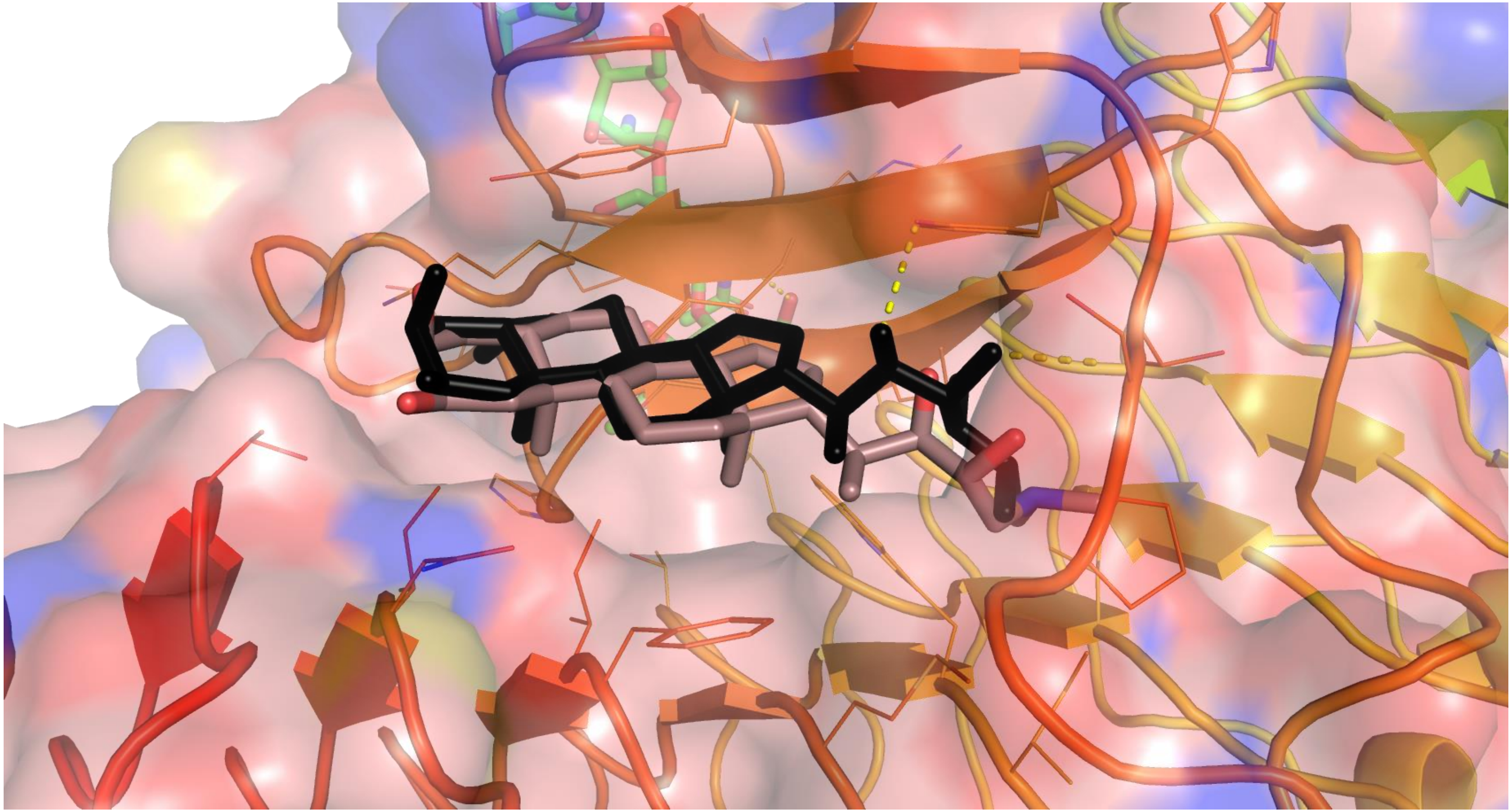

Fig. S16. Poses of brassinolide (black) and new nitrogen analogue **22a** (as a free base) within the BRI1 binding site.

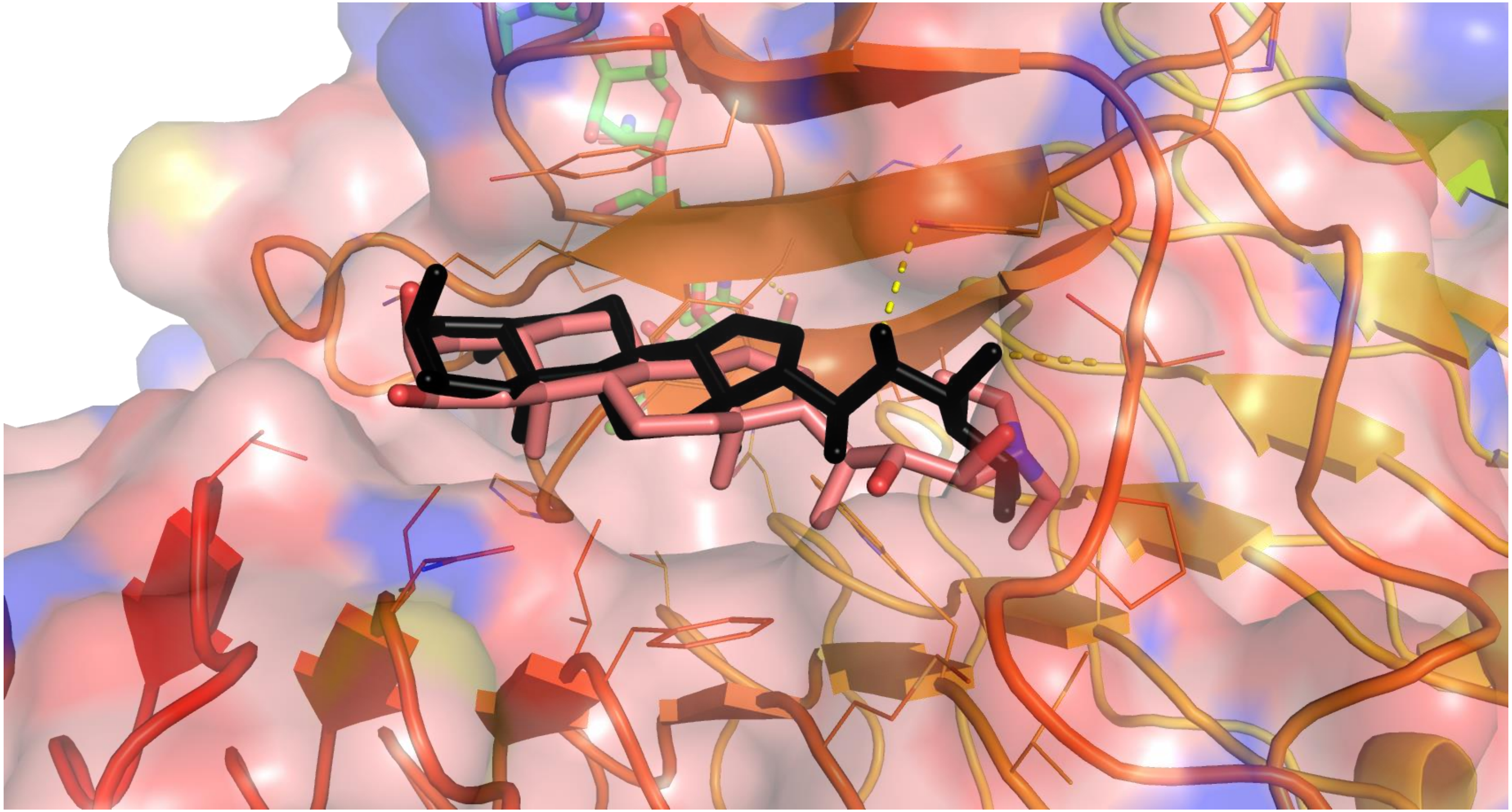

Fig. S17. Poses of brassinolide (black) and new nitrogen analogue **22b** (as a free base) within the BRI1 binding site.

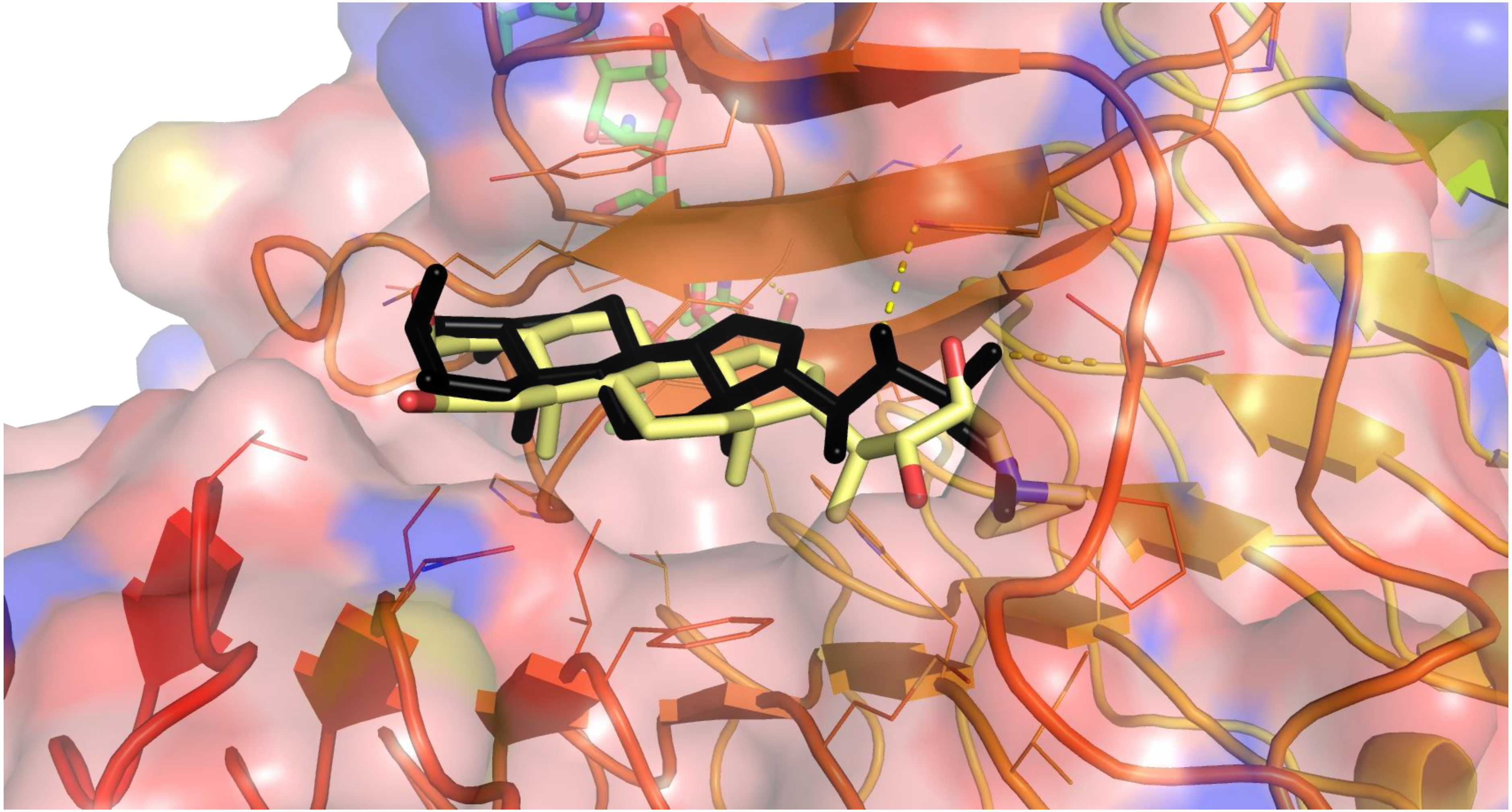

Fig. S18. Poses of brassinolide (black) and new nitrogen analogue **22c** (as a free base) within the BRI1 binding site.

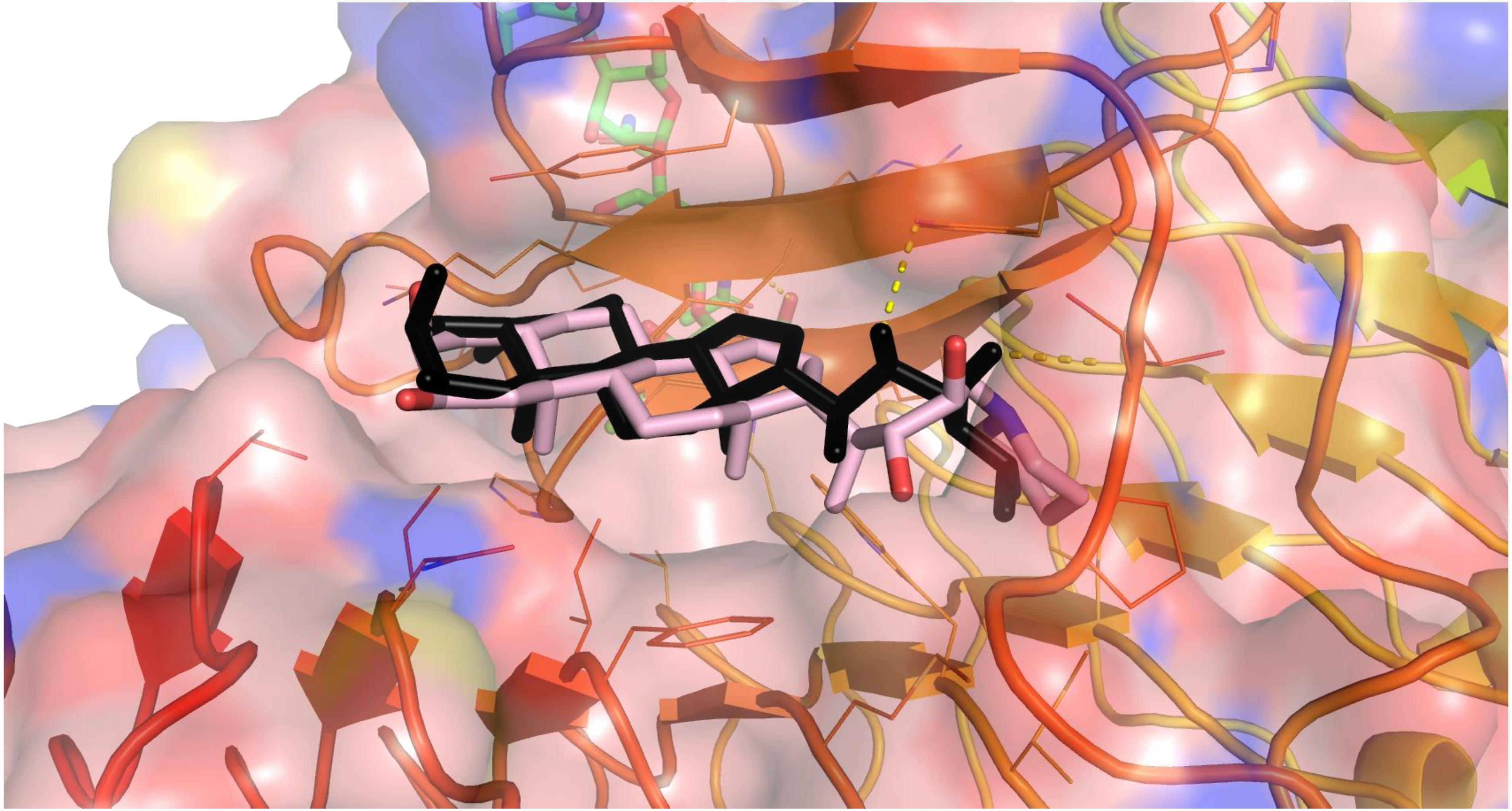

Fig. S19. Poses of brassinolide (black) and new nitrogen analogue **22d** (as a free base) within the BRI1 binding site.

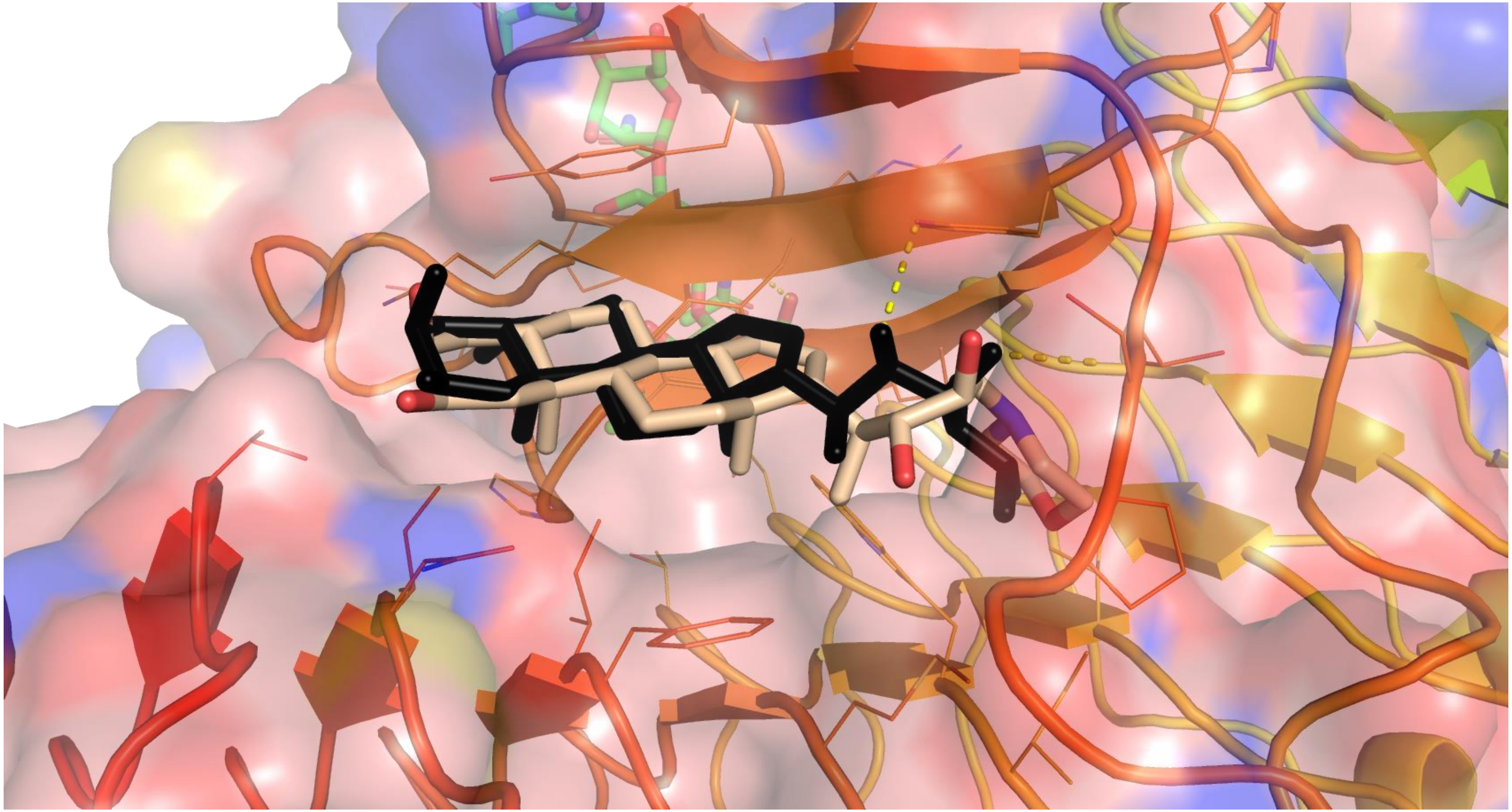

Fig. S20. Poses of brassinolide (black) and new nitrogen analogue **22e** (as a free base) within the BRI1 binding site.

2) Plant bioassay with concentration of 100 nmol/L and data for figures 2, 3, and S21

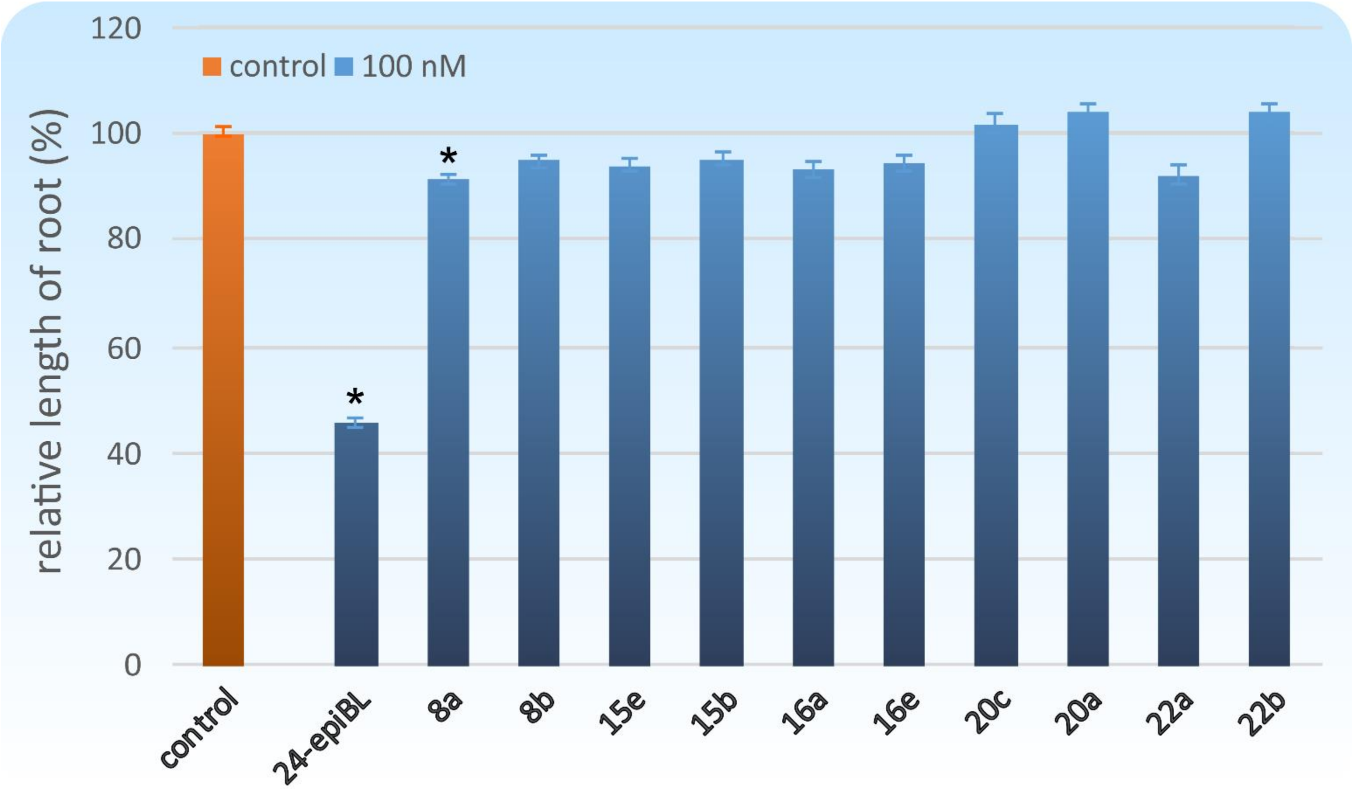

Fig. S21: Effect of selected brassinosteroid derivatives on the inhibition of Arabidopsis root lenght. For each treatment more than 15 seedlings were analyzed in two biological repeats. Error bars represent S.D. Asterisks represent significant changes (t-test), *represents p value <0.05, comparison to control.

Table S2: Data for figure 2, 3, and S21. P value <0.05(t-test), comparison to control.

compound	0.1 nM			1 nM			10 nM			100 nM*		
	mean(mm)	SD	t- test	mean (mm)	SD	t- test	mean (mm)	SD	t- test	mean (mm)	SD	t- test
24-epiBL	15.811	2.001	0.641	13.855	1.73	0,045	7.342	1.29	1.80E-10	6.685	0.761	1.8389E-10
8a	16.12	1.691	0.018	18.41	0.795	0.002	16.46	1.138	0.001	13.329	1.018	0.006
8b	18.41	1.592	0.009	15.15	1,13	0.005	16.95	1.612	0.018	13.836	1.358	0.143
8c	17.61	1.799	0.071	18.58	2.212	0.676	15.97	1.003	0.141			
8d	17.11	1.869	0.004	15.97	1.400	0.098	14.18	2.437	0.618			
8e	17.77	1.303	0.004	17.44	1.886	0.040	15.16	2.54	0.385			
15a	14.83	2,058	0.288	14.29	1.966	0.939	14.34	1.167	0.110			
15b	14.93	1.922	0.008	16.13	2.108	0.011	14.67	1.749	0.002	13.903	1.294	0.169
15c	16.78	0.953	0.076	14.37	3.418	0.002	17.21	2.256	0.449			
15d	14.18	1.532	0.909	14.11	1.216	0.273	15.15	1.518	0.002			
15e	17.21	2.871	0.19	17.61	2.784	0.168	16.21	2.261	0.211	13.731	1.411	0.108
16a	18.91	3.124	0.926	15.64	3.044	0.004	15.32	2.031	0.133	13.626	1.477	0.084
16b	17.27	1.909	0.282	17.61	2.282	0.213	16.13	1.916	0.094			
16c	15.97	2.261	0.389	14.99	2.457	0.090	15.16	3.018	0.051			
16d	15.32	2.407	0.341	15.65	2.383	0.136	17.61	2.376	0.006			
16e	17.21	2.922	0.009	17.61	2.105	0.002	16.13	1.97	0.146	13.771	1.689	0.177
20a	16.13	1.372	0.017	14.67	2.714	0.006	14.18	2.841	0.013	15.192	1.609	0.322
20b	16.78	1.248	0.109	15.32	1.467	0.001	14.99	1.975	0.005			
20c	15.97	1.15	0.032	15.81	1.813	0.002	15.15	1.607	0.002	14.873	1.725	0.668
20d	14.37	1.478	0.083	14.50	1.372	0.007	14.51	1.90	0.073			
20e	17.44	1.832	0.523	16.78	1.203	0.003	15.97	2,688	0.554			
22a	19.56	0.982	0.005	18.17	1.284	0.281	17.11	1.823	0.665	13.464	1.712	0.073
22b	14.51	1.166	0.001	15.32	1.425	0.007	15.48	1.762	0.058	15.160	1.655	0.359
22c	15.97	1.502	0.483	14.83	1,46	0.125	15.81	1.277	0.779			
22d	15.48	1.756	0.893	15.97	1.094	0.216	16.62	1.893	0.829			
22e	17.93	1.683	0.007	17.27	1.372	0.647	16.95	1.803	0.133			

control (mean ± SD) 16.3 ± 1.21
control - 100 nM (mean ± SD) 14.61 ± 0.85
*only selected compounds were tested

3) Cytotoxicity

Table S3: Cytotoxic activities, IC_{50} (µM/L)[a], of compounds in three tumor and one normal fibroblast cell line. $IC_{50} > 50$ means that compound is inactive.

Compound	CEM	MCF7	HeLa	BJ
**8a**	>50	>50 (160 %)[b]	>50	>50
**8b**	>50	>50 (130 %)	>50	>50
**8c**	>50	>50 (150 %)	>50	>50
**8d**	>50	>50 (150 %)	>50	>50
**8e**	>50	>50 (160 %)	>50	>50
**15a**	>50	>50 (130 %)	>50	>50
**15b**	>50	>50 (120 %)	>50	>50
**15c**	>50	>50 (130 %)	>50	>50
**15d**	>50	>50 (120 %)	>50	>50
**15e**	>50	>50 (150 %)	>50	>50
**16a**	>50	>50 (130 %)	>50	>50
**16b**	>50	>50 (130 %)	>50	>50
**16c**	>50	>50 (120 %)	>50	>50
**16d**	>50	>50 (140 %)	>50	>50
**16e**	>50	>50 (135 %)	>50	>50
**20a**	>50	>50	>50	>50
**20b**	>50	>50	>50	>50
**20c**	>50	>50	>50	>50
**20d**	>50	>50	>50	>50
**20e**	>50	>50	>50	>50
**22a**	>50	>50	>50	>50
**22b**	>50	>50	>50	>50
**22c**	>50	>50	>50	>50
**22d**	>50	>50	>50	>50
**22e**	>50	>50	>50	>50

[a] The lowest concentration that kills 50% of cells.

[b] Increase of cell proliferation.

4) ^{1}H and ^{13}C NMR and MS spectra of new compounds

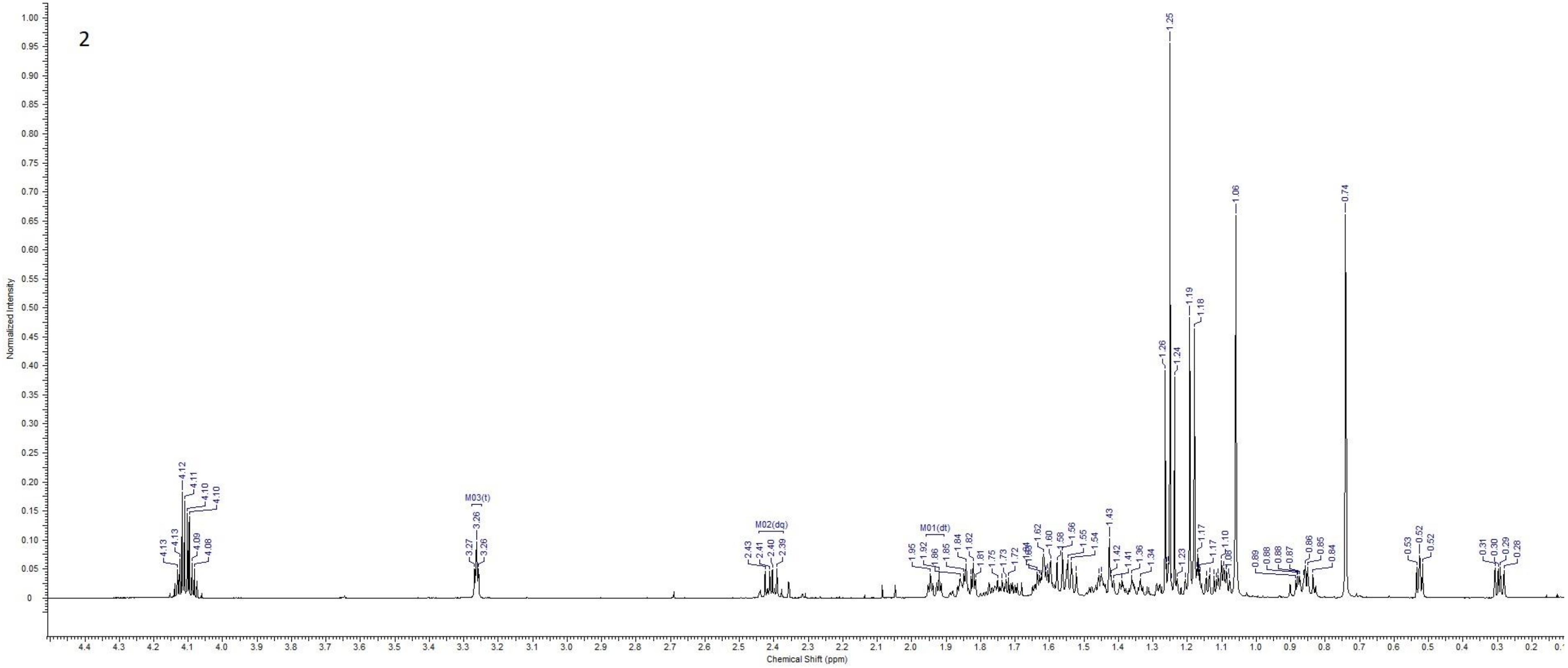

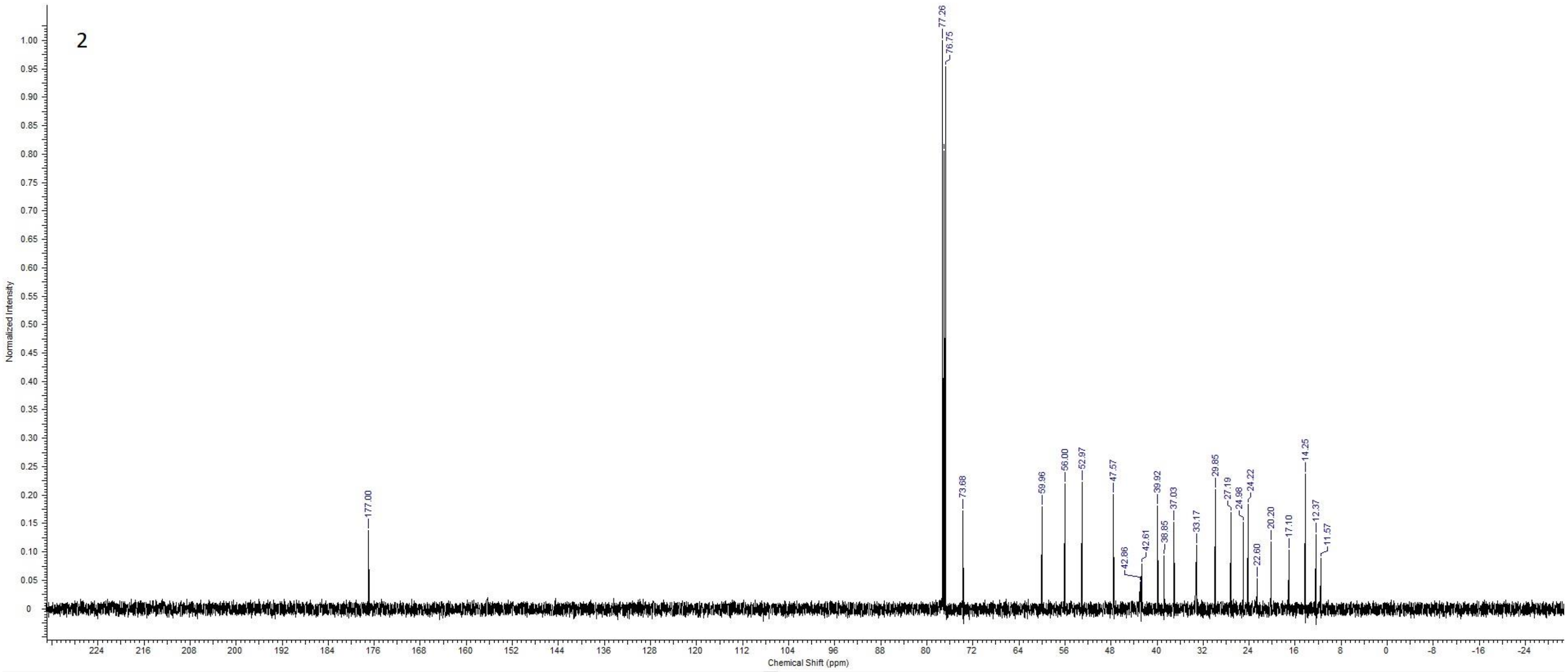

2
Normalized Intensity
Chemical Shift (ppm)
177.00
77.26
76.75
73.68
59.96
56.00
52.97
47.57
42.86
42.61
39.92
38.85
37.03
33.17
29.85
27.19
24.98
24.22
22.60
20.20
17.10
14.25
12.37
11.57

Elemental Composition

File Edit View Process Help

Single Mass Analysis
Tolerance = 5.0 PPM / DBE: min = -1.5, max = 50.0
Element prediction: Off
Number of isotope peaks used for i-FIT = 3
Monoisotopic Mass, Even Electron Ions
52 formula(e) evaluated with 1 results within limits (up to 50 best isotopic matches for each mass)
Elements Used:

Mass	Calc. Mass	mDa	PPM	DBE	Formula	i-FIT	i-FIT Norm	Fit Conf %	C	H	O
375.2899	375.2899	0.0	0.0	5.5	C24 H39 O3	784.6	n/a	n/a	24	39	3

2

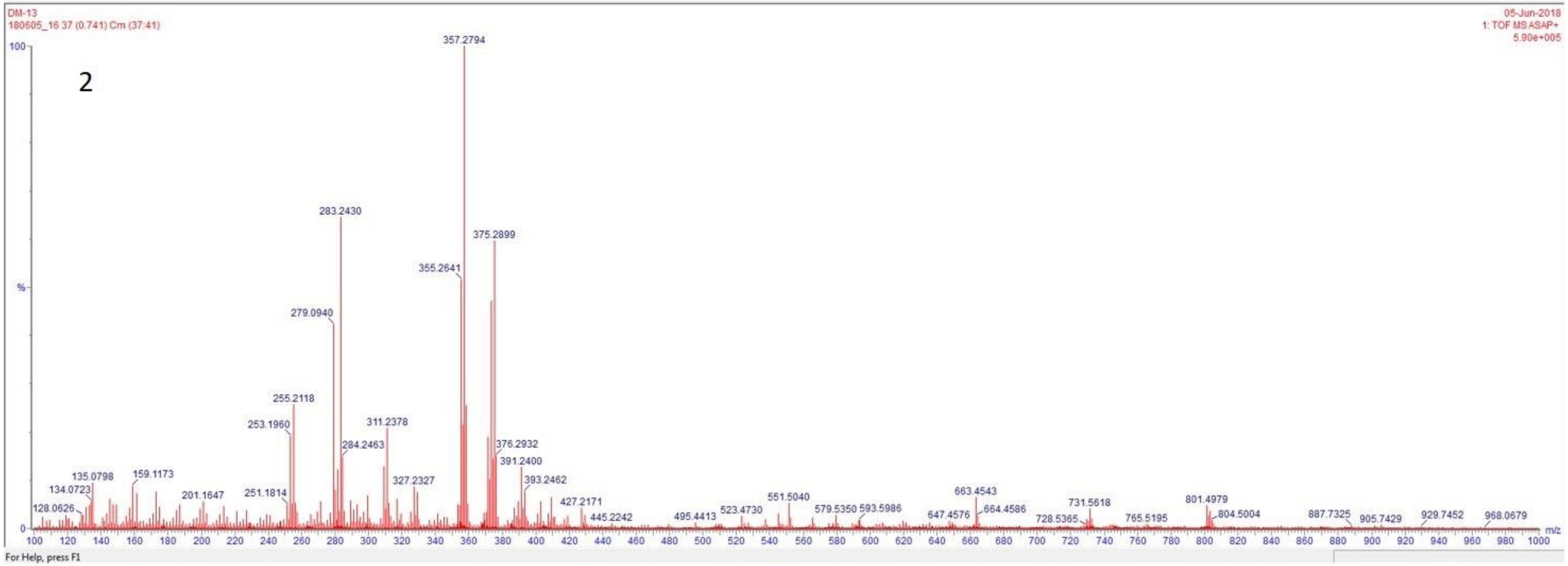

For Help, press F1

3

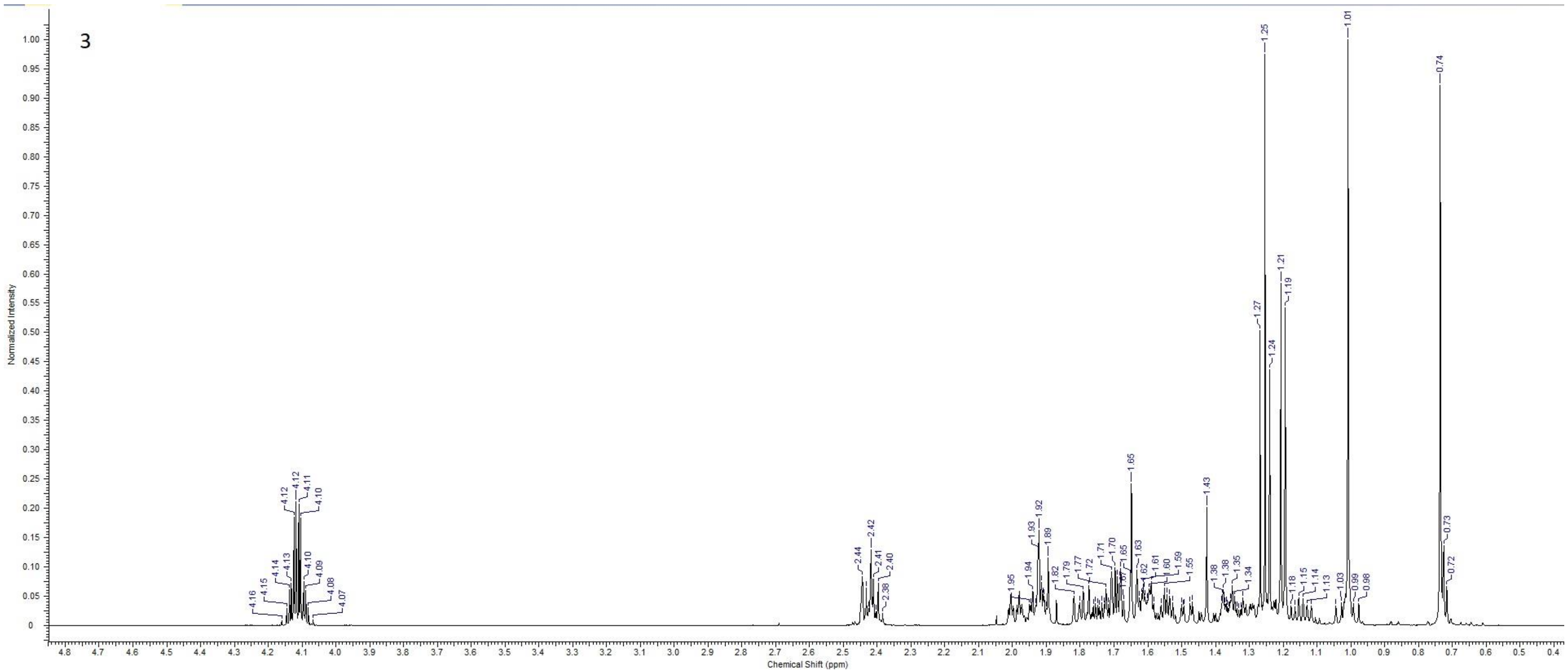

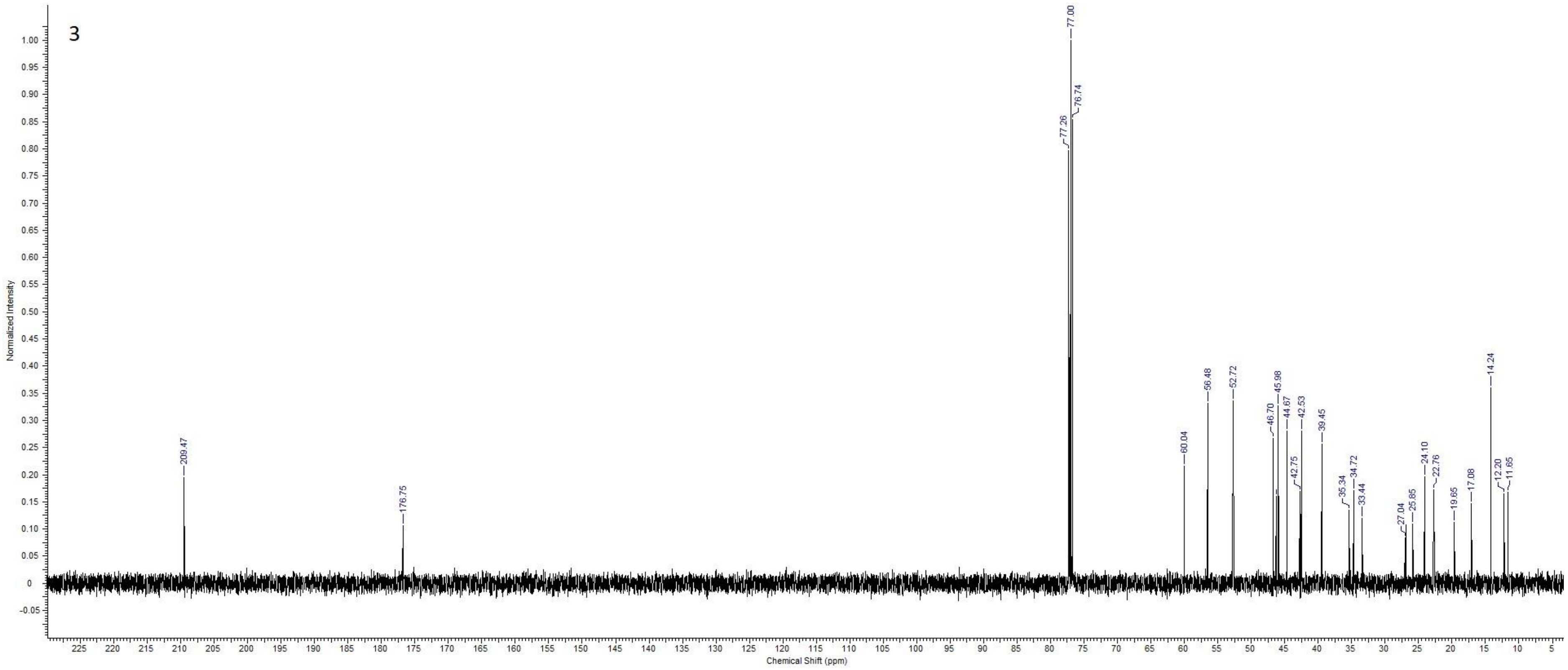
3
Normalized Intensity
1.00
0.95
0.90
0.85
0.80
0.75
0.70
0.65
0.60
0.55
0.50
0.45
0.40
0.35
0.30
0.25
0.20
0.15
0.10
0.05
0
-0.05
209.47
176.75
77.26
77.00
76.74
60.04
56.48
52.72
46.70
45.98
44.67
42.75
42.53
39.45
35.34
34.72
33.44
27.04
25.85
24.10
22.76
19.65
17.08
14.24
12.20
11.65
225 220 215 210 205 200 195 190 185 180 175 170 165 160 155 150 145 140 135 130 125 120 115 110 105 100 95 90 85 80 75 70 65 60 55 50 45 40 35 30 25 20 15 10 5
Chemical Shift (ppm)

Elemental Composition

File Edit View Process Help

Single Mass Analysis

Tolerance = 10.0 PPM / DBE: min = -1.5, max = 50.0

Element prediction: Off

Number of isotope peaks used for i-FIT = 3

Monoisotopic Mass, Even Electron Ions

49 formula(e) evaluated with 1 results within limits (up to 50 best isotopic matches for each mass)

Elements Used:

Mass	Calc. Mass	mDa	PPM	DBE	Formula	i-FIT	i-FIT Norm	Fit Conf %	C	H	O
373.2744	373.2743	0.1	0.3	6.5	C24 H37 O3	1071.4	n/a	n/a	24	37	3

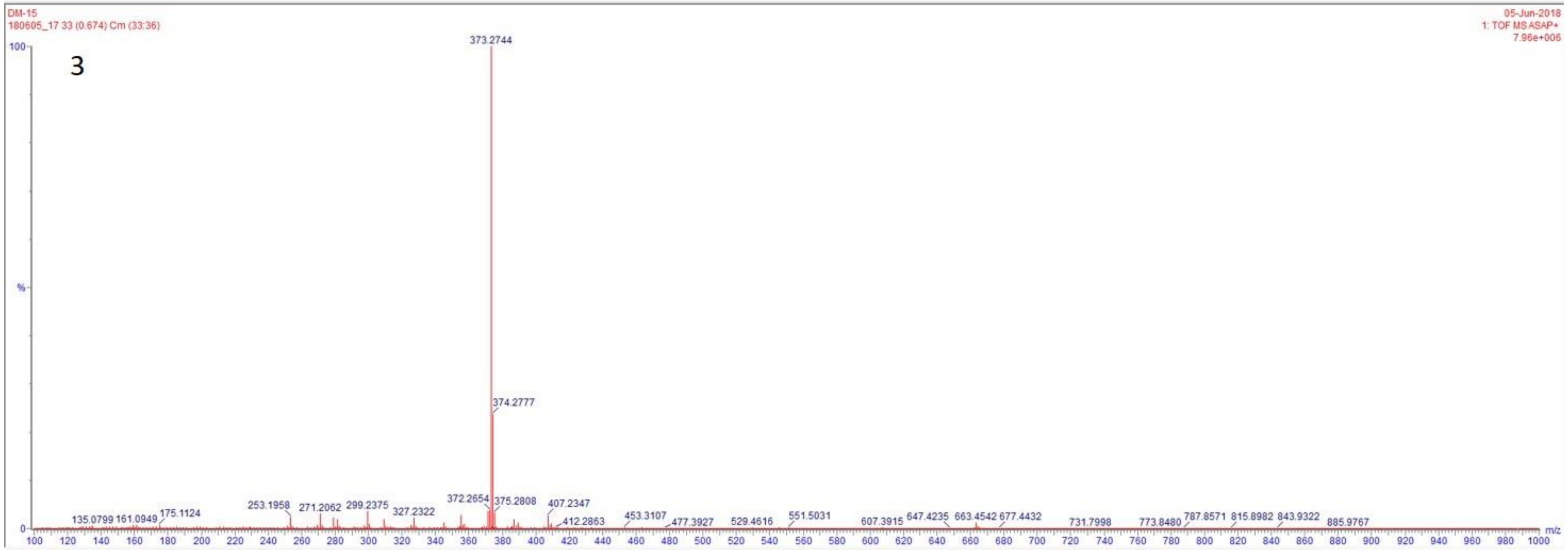

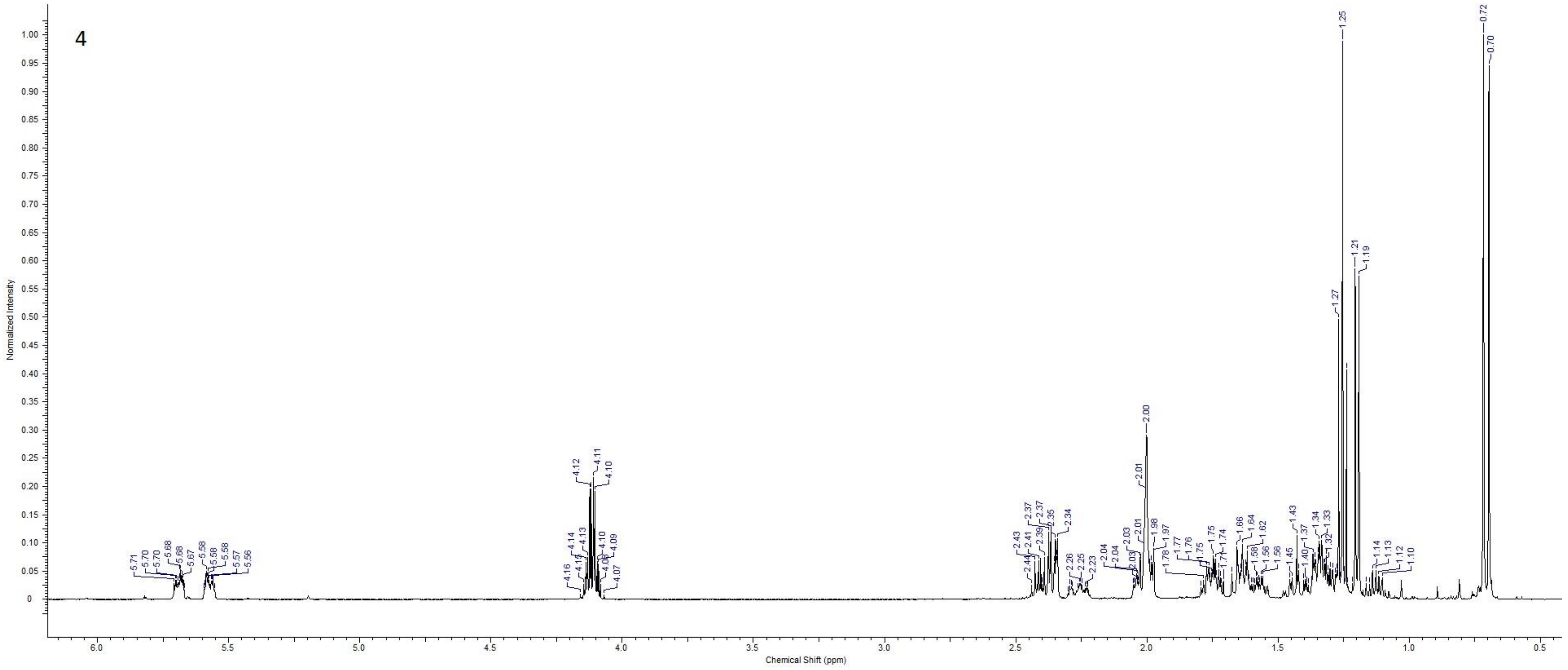
4
Normalized Intensity
Chemical Shift (ppm)

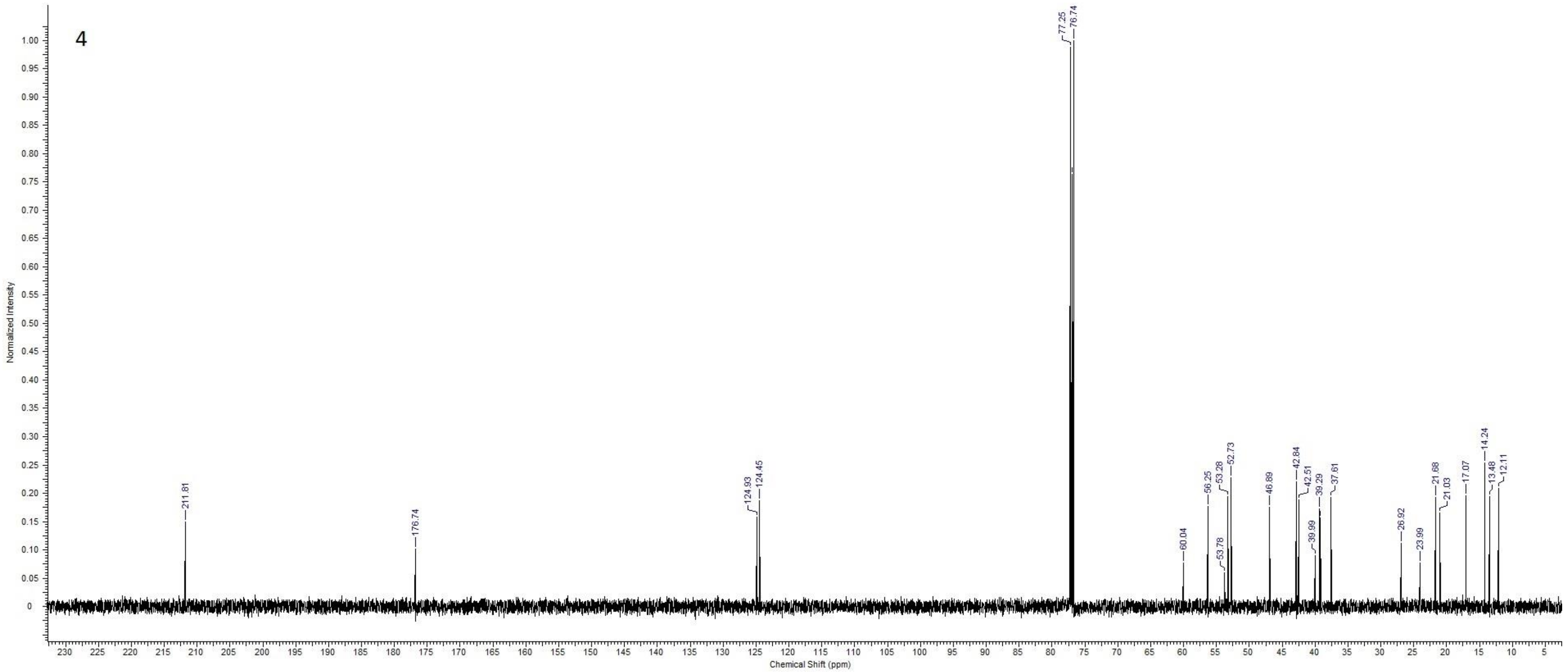
4
211.81
176.74
124.93
124.45
77.25
76.74
60.04
56.25
53.78
53.28
52.73
46.89
42.84
42.51
39.99
39.29
37.61
26.92
23.99
21.68
21.03
17.07
14.24
13.48
12.11
Normalized Intensity
Chemical Shift (ppm)

Elemental Composition

File Edit View Process Help

Single Mass Analysis

Tolerance = 10.0 PPM / DBE: min = -1.5, max = 50.0

Element prediction: Off

Number of isotope peaks used for i-FIT = 3

Monoisotopic Mass, Even Electron Ions

49 formula(e) evaluated with 1 results within limits (up to 50 best isotopic matches for each mass)

Elements Used:

Mass	Calc. Mass	mDa	PPM	DBE	Formula	i-FIT	i-FIT Norm	Fit Conf %	C	H	O
373.2745	373.2743	0.2	0.5	6.5	C24 H37 O3	966.6	n/a	n/a	24	37	3

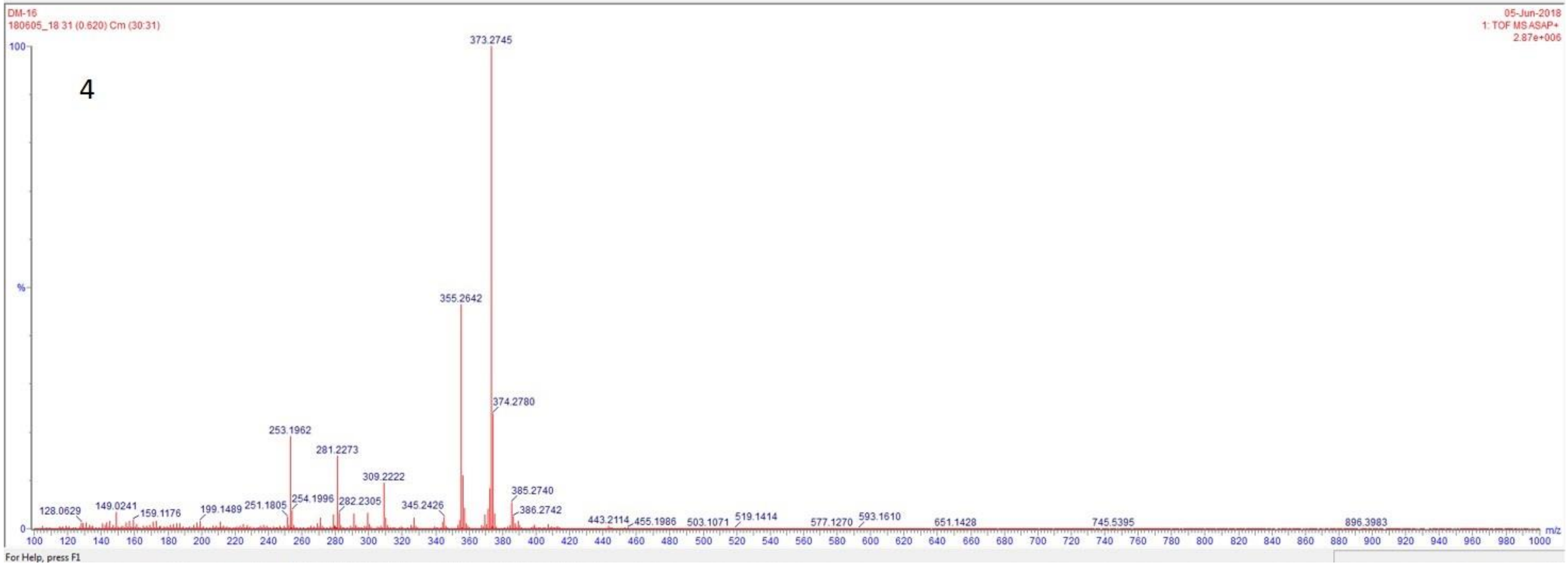

For Help, press F1

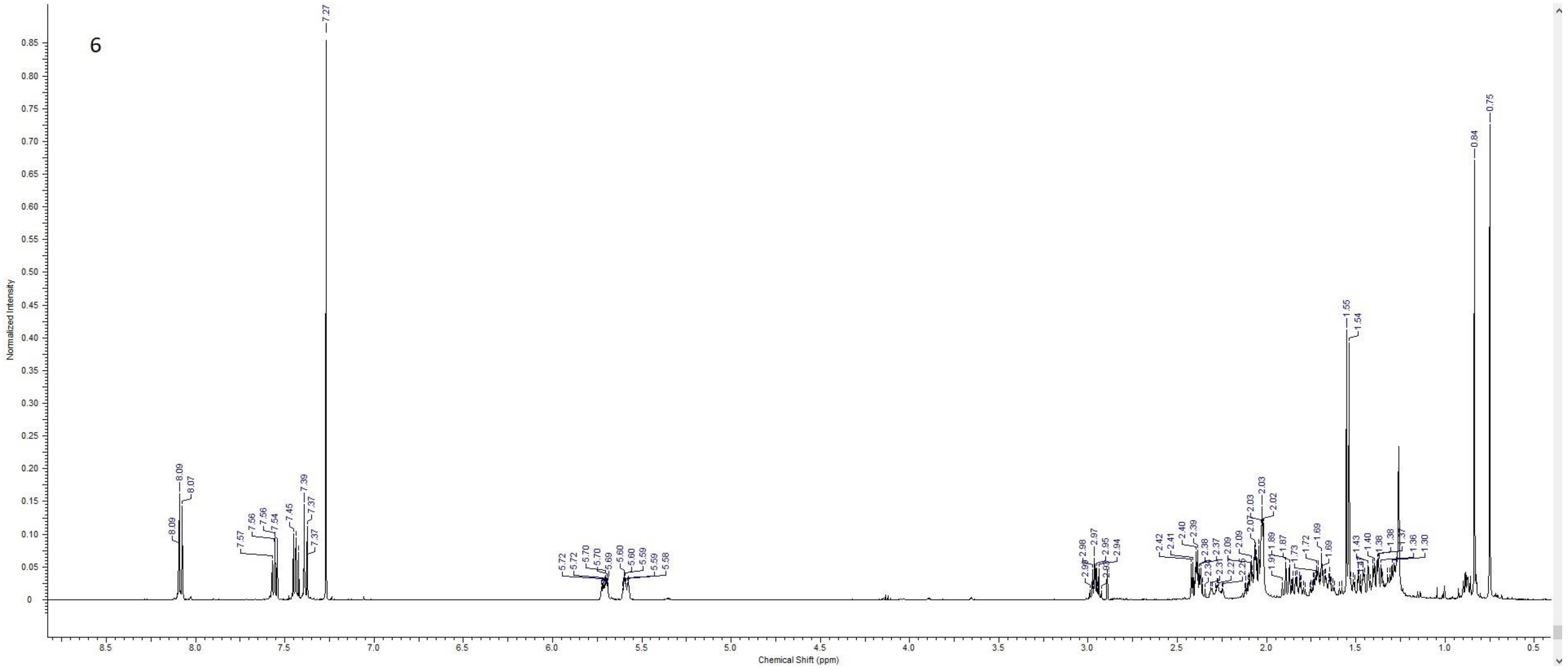
6
Normalized Intensity
Chemical Shift (ppm)

6

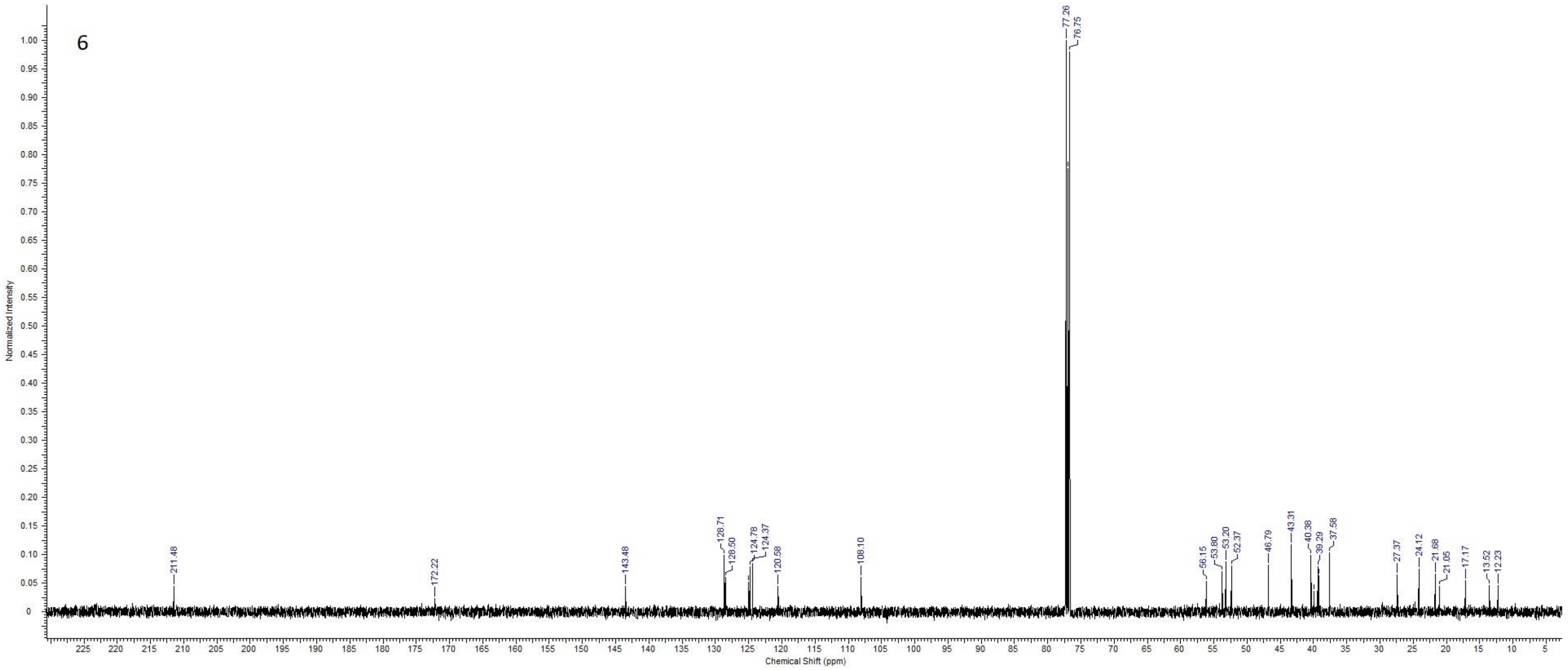

Normalized Intensity
Chemical Shift (ppm)
211.48
172.22
143.48
128.71
128.50
124.78
124.37
120.58
108.10
77.26
76.75
56.15
53.80
53.20
52.37
46.79
43.31
40.38
39.29
37.58
27.37
24.12
21.68
21.05
17.17
13.52
12.23

Elemental Composition

File Edit View Process Help

Single Mass Analysis

Tolerance = 1.0 PPM / DBE: min = -1.5, max = 50.0

Element prediction: Off

Number of isotope peaks used for i-FIT = 3

Monoisotopic Mass, Even Electron Ions

610 formula(e) evaluated with 1 results within limits (up to 50 best isotopic matches for each mass)

Elements Used:

Mass	Calc. Mass	mDa	PPM	DBE	Formula	i-FIT	i-FIT Norm	Fit Conf %	C	H	N	O
462.2758	462.2757	0.1	0.2	12.5	C28 H36 N3 O3	1076.1	n/a	n/a	28	36	3	3

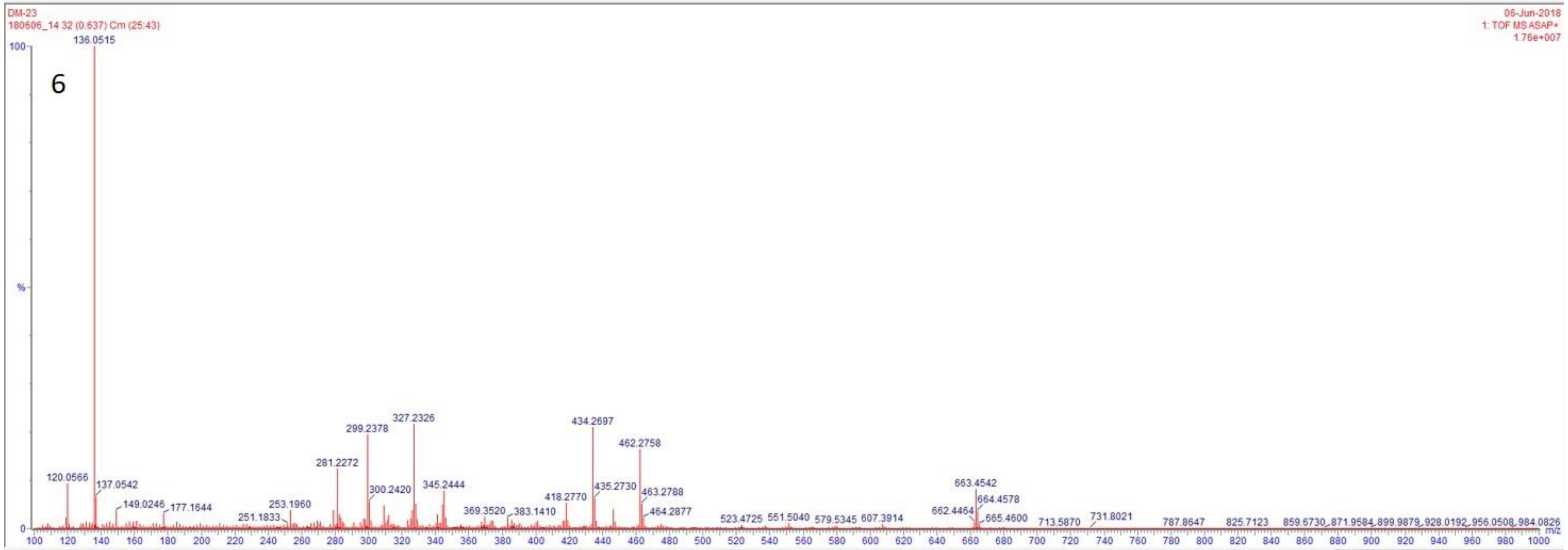

For Help, press F1

7a

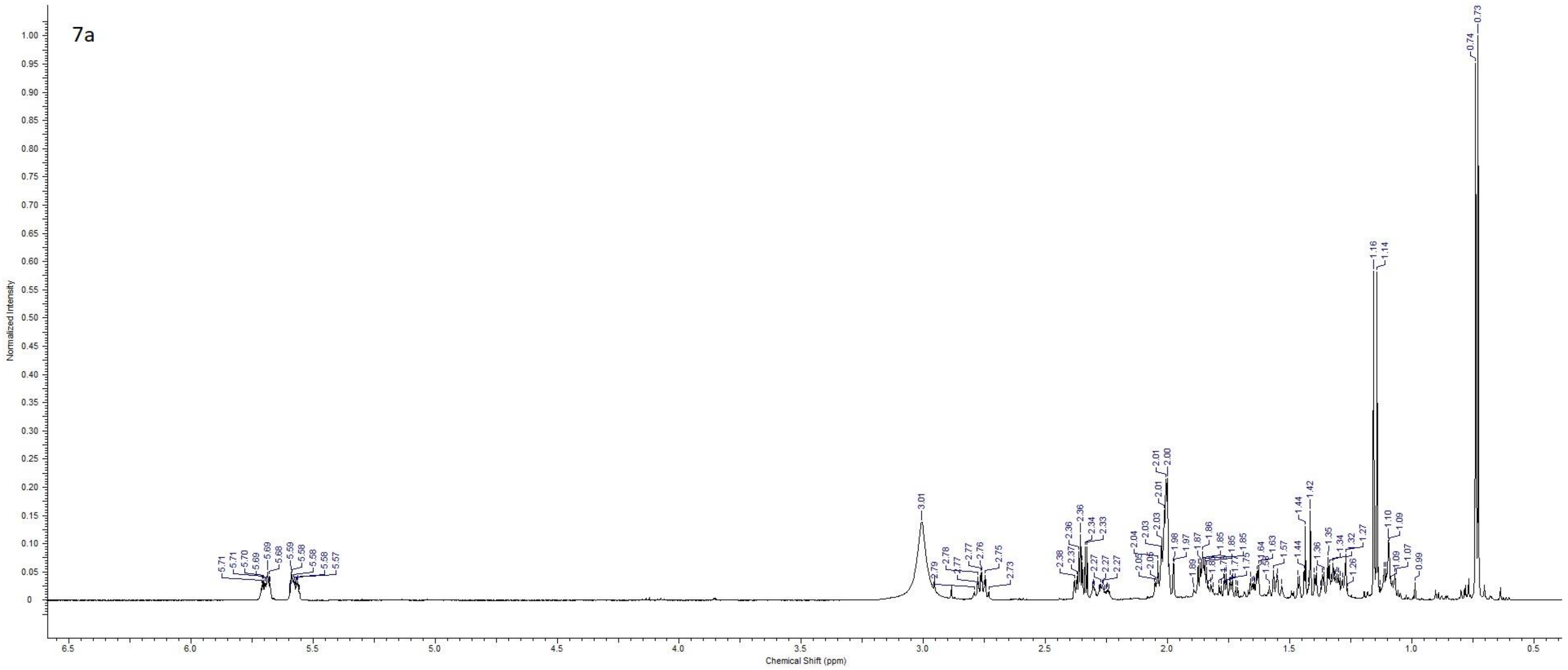

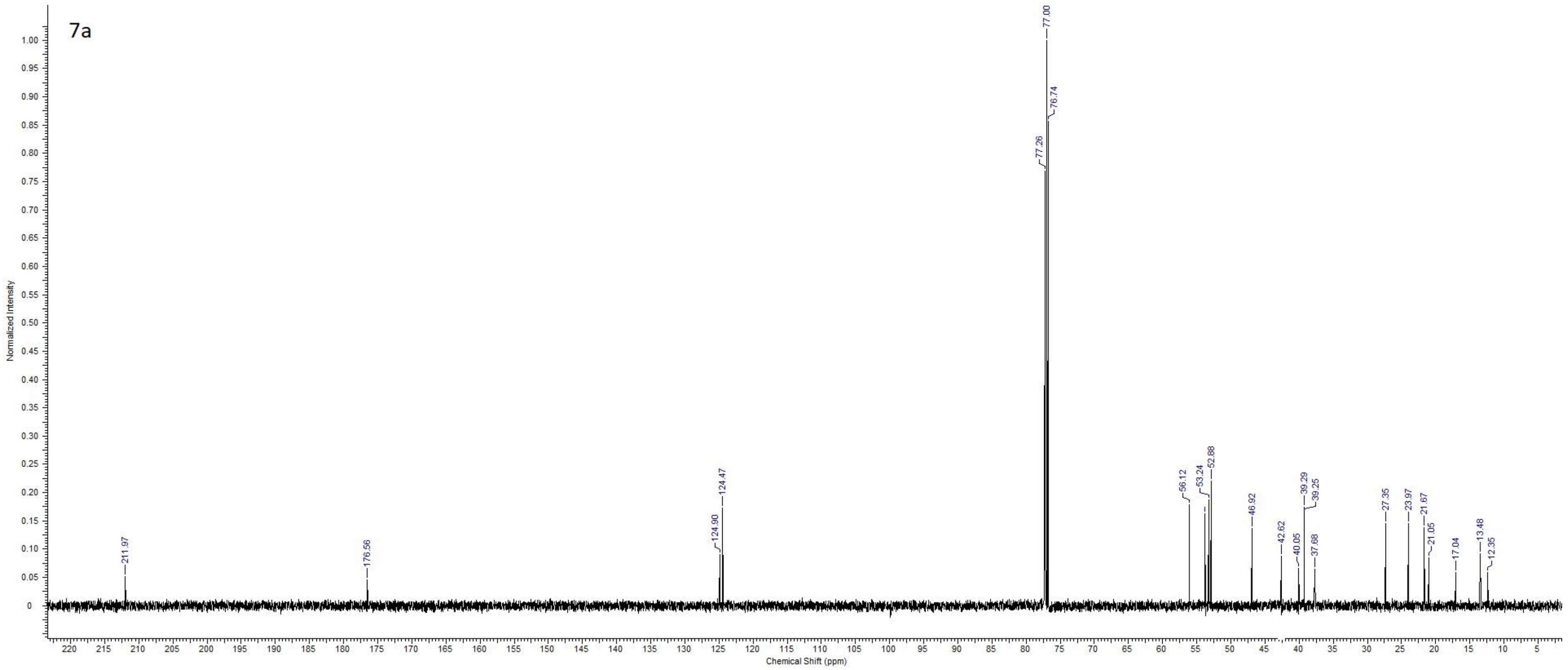
7a
211.97
176.56
124.90
124.47
77.26
77.00
76.74
56.12
53.24
52.88
46.92
42.62
40.05
39.29
39.25
37.68
27.35
23.97
21.67
21.05
17.04
13.48
12.35
Normalized Intensity
Chemical Shift (ppm)

Elemental Composition

File Edit View Process Help

Single Mass Analysis
Tolerance = 1.0 PPM / DBE: min = -1.5, max = 50.0
Element prediction: Off
Number of isotope peaks used for i-FIT = 3
Monoisotopic Mass, Even Electron Ions
477 formula(e) evaluated with 1 results within limits (up to 50 best isotopic matches for each mass)
Elements Used:

Mass	Calc. Mass	mDa	PPM	DBE	Formula	i-FIT	i-FIT Norm	Fit Conf %	C	H	N	O
372.2903	372.2903	0.0	0.0	6.5	C24 H38 N O2	1024.2	n/a	n/a	24	38	1	2

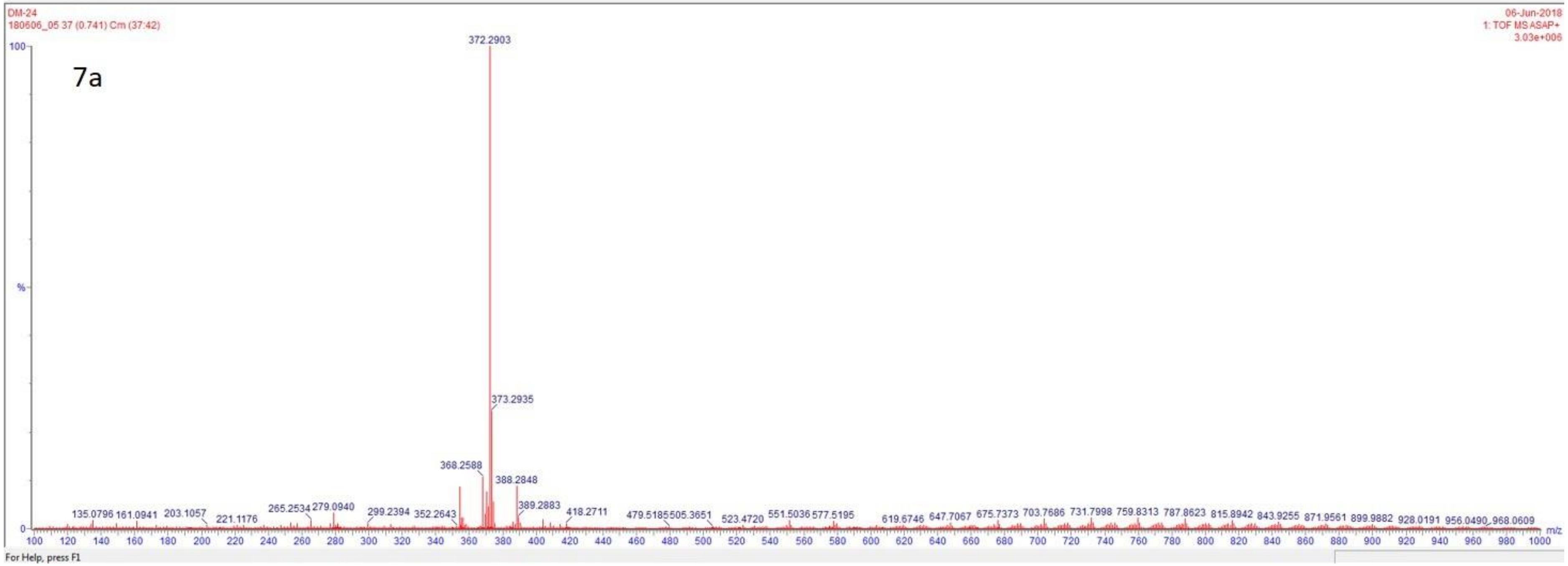

For Help, press F1

7b

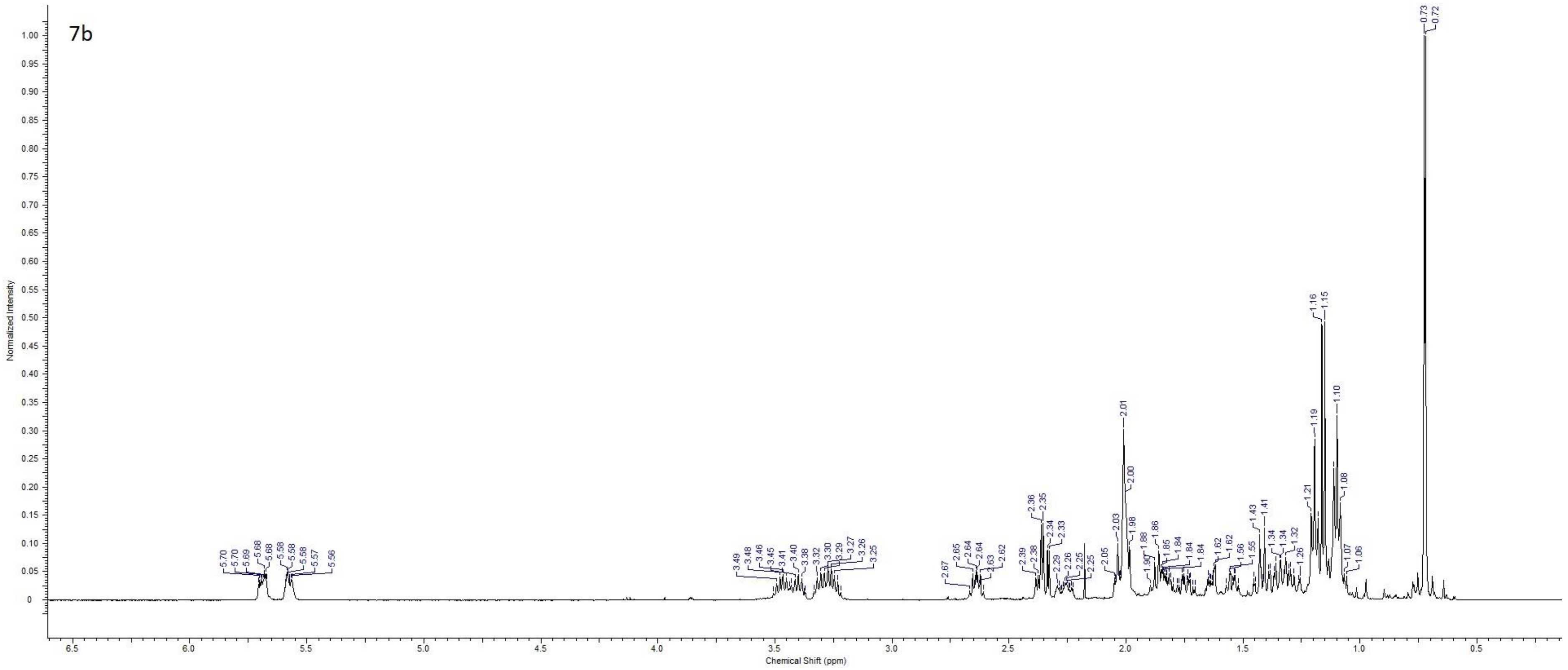

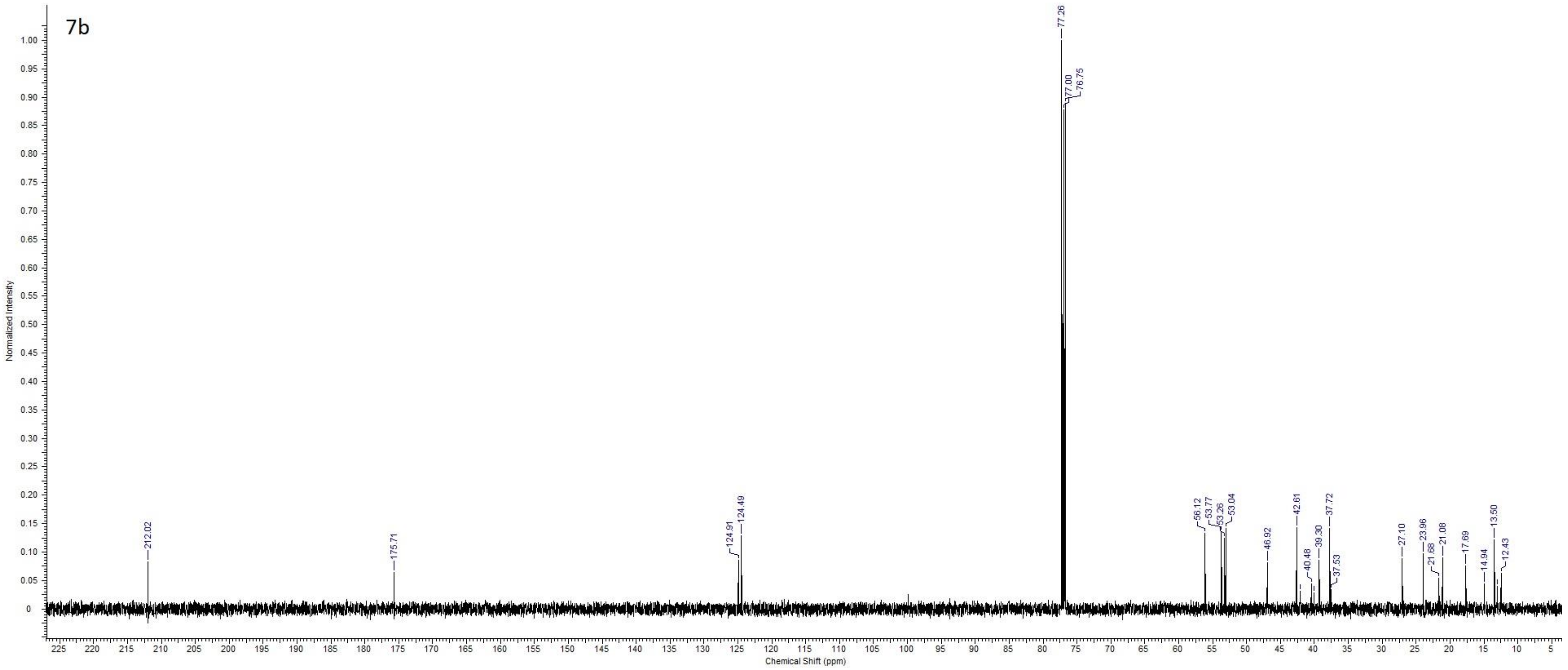

7b
212.02
175.71
124.91
124.49
77.26
77.00
76.75
56.12
53.77
53.26
53.04
46.92
42.61
40.48
39.30
37.72
37.53
27.10
23.96
21.68
21.08
17.69
14.94
13.50
12.43
Normalized Intensity
Chemical Shift (ppm)

Elemental Composition

File Edit View Process Help

Single Mass Analysis

Tolerance = 1.0 PPM / DBE: min = -1.5, max = 50.0

Element prediction: Off

Number of isotope peaks used for i-FIT = 3

Monoisotopic Mass, Even Electron Ions

517 formula(e) evaluated with 1 results within limits (up to 50 best isotopic matches for each mass)

Elements Used:

Mass	Calc. Mass	mDa	PPM	DBE	Formula	i-FIT	i-FIT Norm	Fit Conf %	C	H	N	O
400.3215	400.3216	-0.1	-0.2	6.5	C26 H42 N O2	892.1	n/a	n/a	26	42	1	2

7b

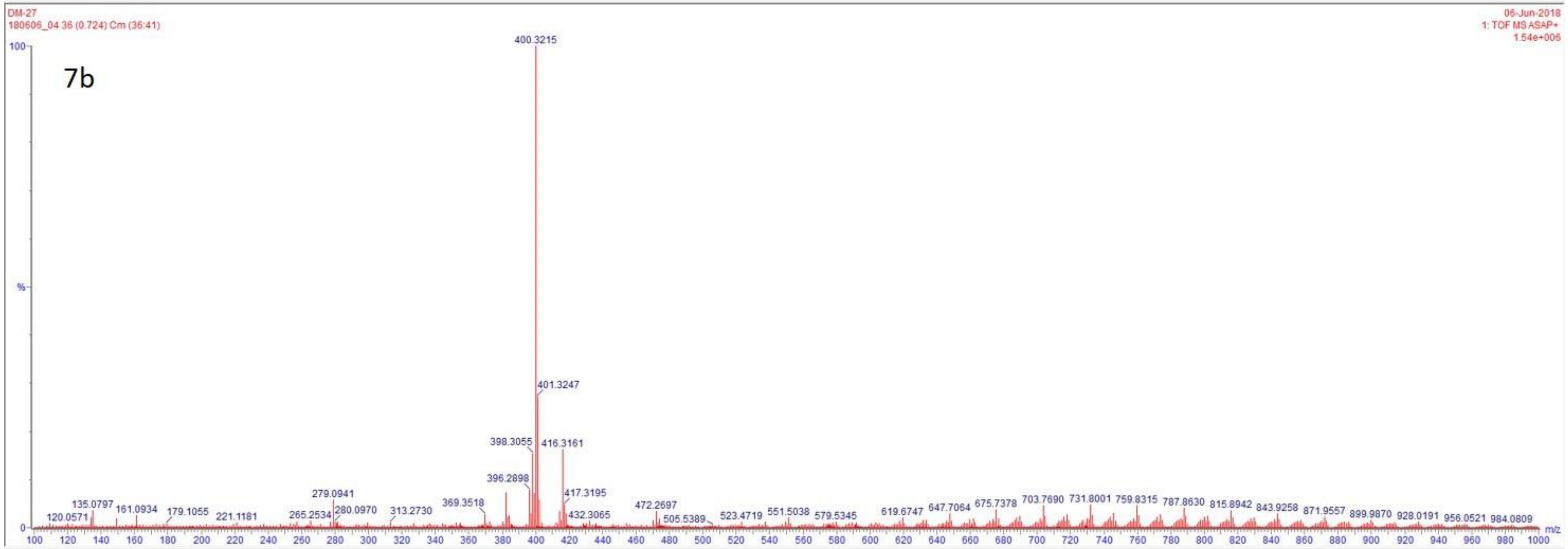

For Help, press F1

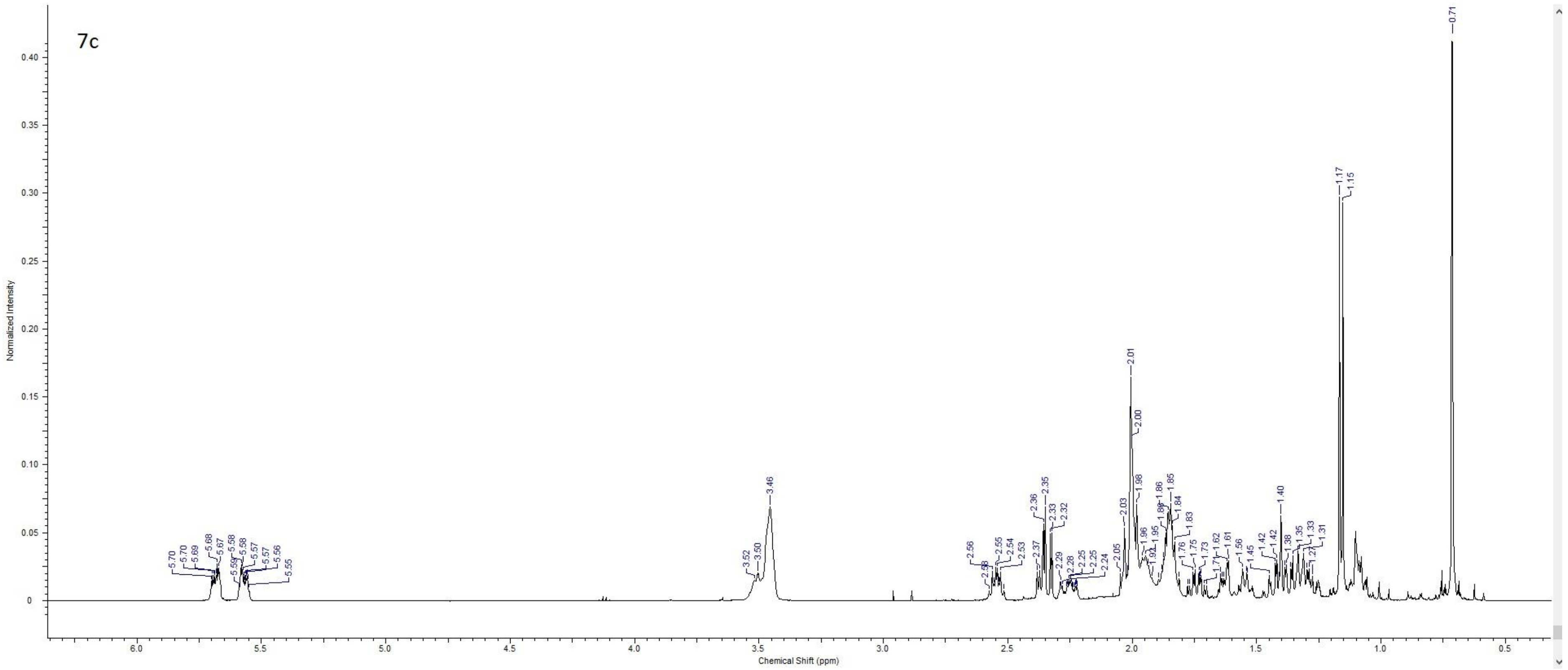
7c
Normalized Intensity
Chemical Shift (ppm)

7c

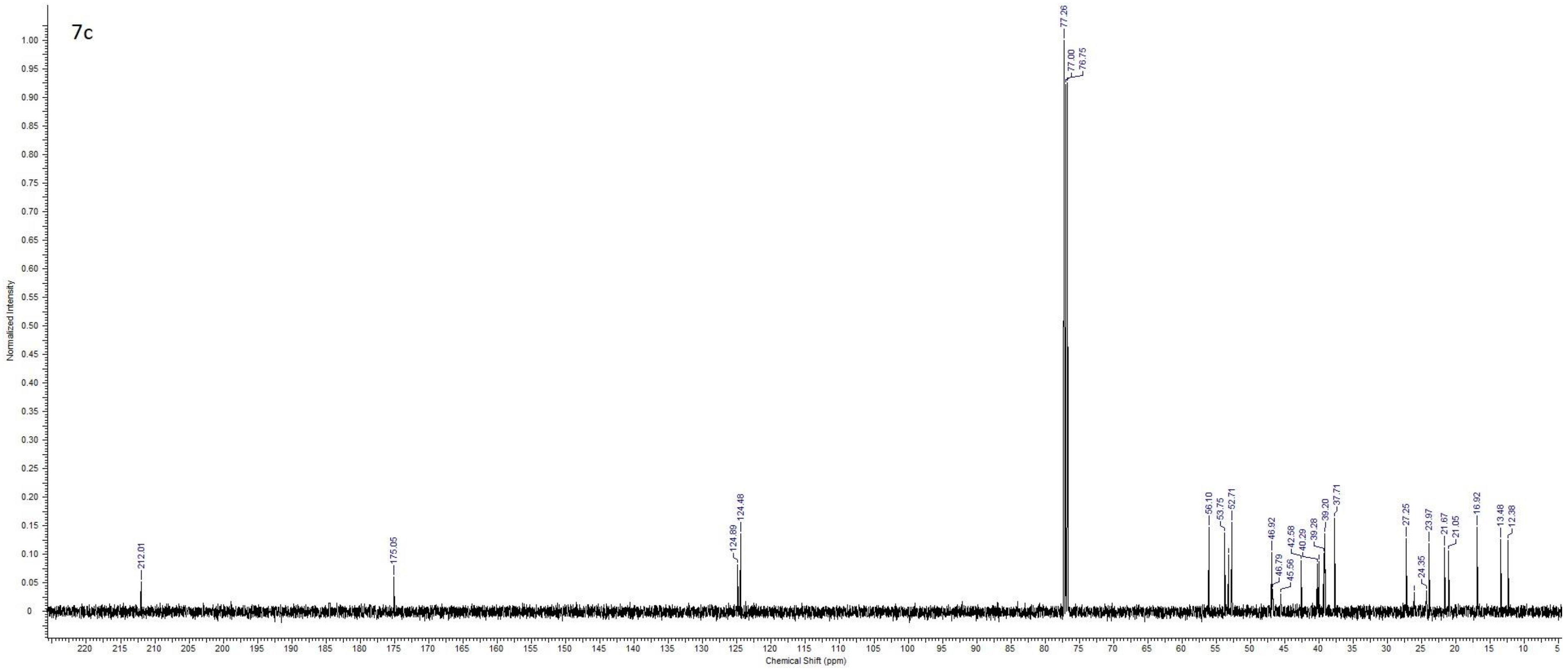

212.01
175.05
124.89
124.48
77.26
77.00
76.75
56.10
53.75
52.71
46.92
46.79
45.56
42.58
40.29
39.28
39.20
37.71
27.25
24.35
23.97
21.67
21.05
16.92
13.48
12.38
Normalized Intensity
Chemical Shift (ppm)

Elemental Composition

File Edit View Process Help

Single Mass Analysis
Tolerance = 1.0 PPM / DBE: min = -1.5, max = 50.0
Element prediction: Off
Number of isotope peaks used for i-FIT = 3
Monoisotopic Mass, Even Electron Ions
513 formula(e) evaluated with 1 results within limits (up to 50 best isotopic matches for each mass)
Elements Used:

Mass	Calc. Mass	mDa	PPM	DBE	Formula	i-FIT	i-FIT Norm	Fit Conf %	C	H	N	O
398.3062	398.3059	0.3	0.8	7.5	C26 H40 N O2	1026.4	n/a	n/a	26	40	1	2

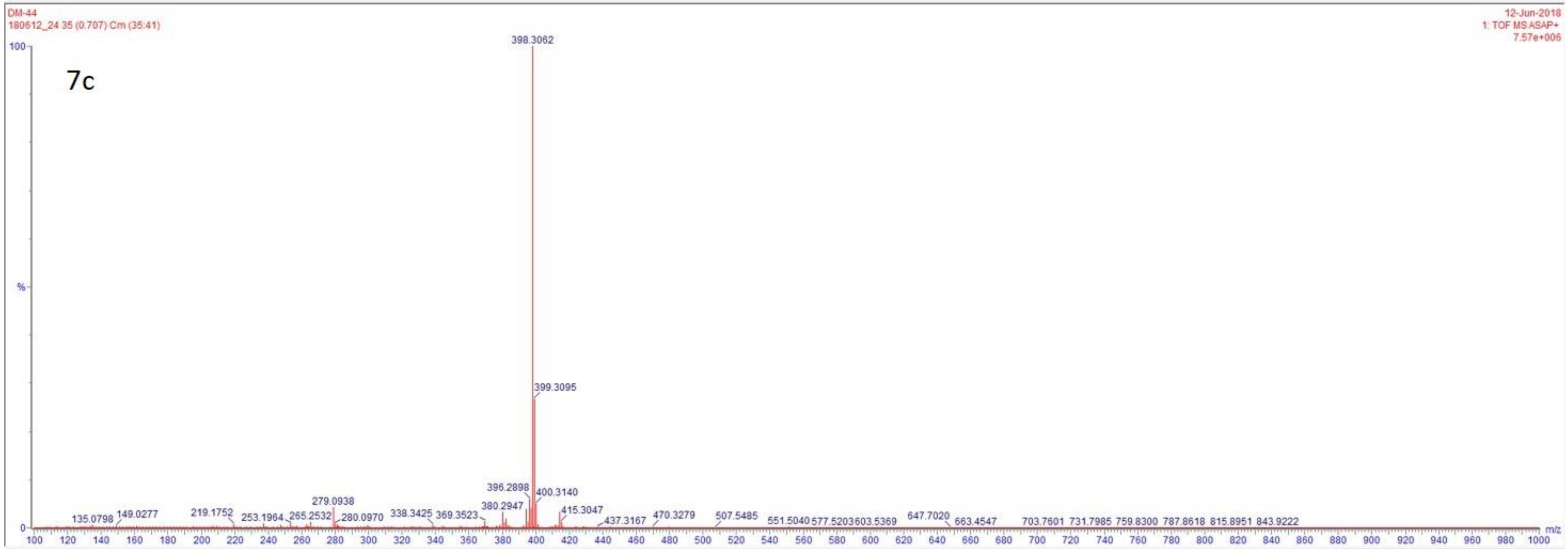

For Help, press F1

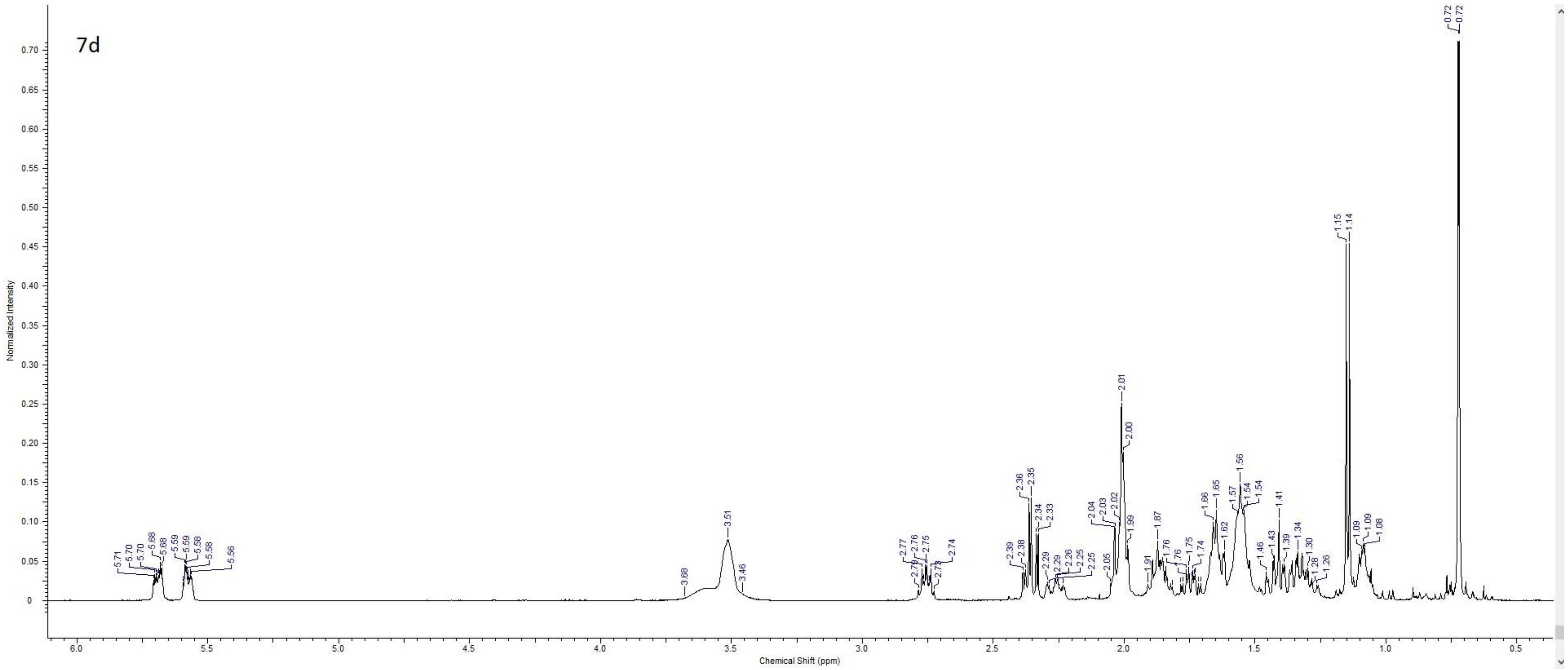
7d
Normalized Intensity
Chemical Shift (ppm)
5.71
5.70
5.70
5.68
5.68
5.59
5.59
5.58
5.58
5.56
3.68
3.51
3.46
2.77
2.76
2.75
2.74
2.39
2.38
2.36
2.35
2.34
2.33
2.29
2.29
2.26
2.25
2.25
2.05
2.04
2.03
2.02
2.01
2.00
1.99
1.91
1.87
1.76
1.76
1.75
1.74
1.66
1.65
1.62
1.57
1.56
1.54
1.54
1.46
1.43
1.41
1.39
1.34
1.30
1.28
1.26
1.15
1.14
1.09
1.09
1.08
0.72
0.72

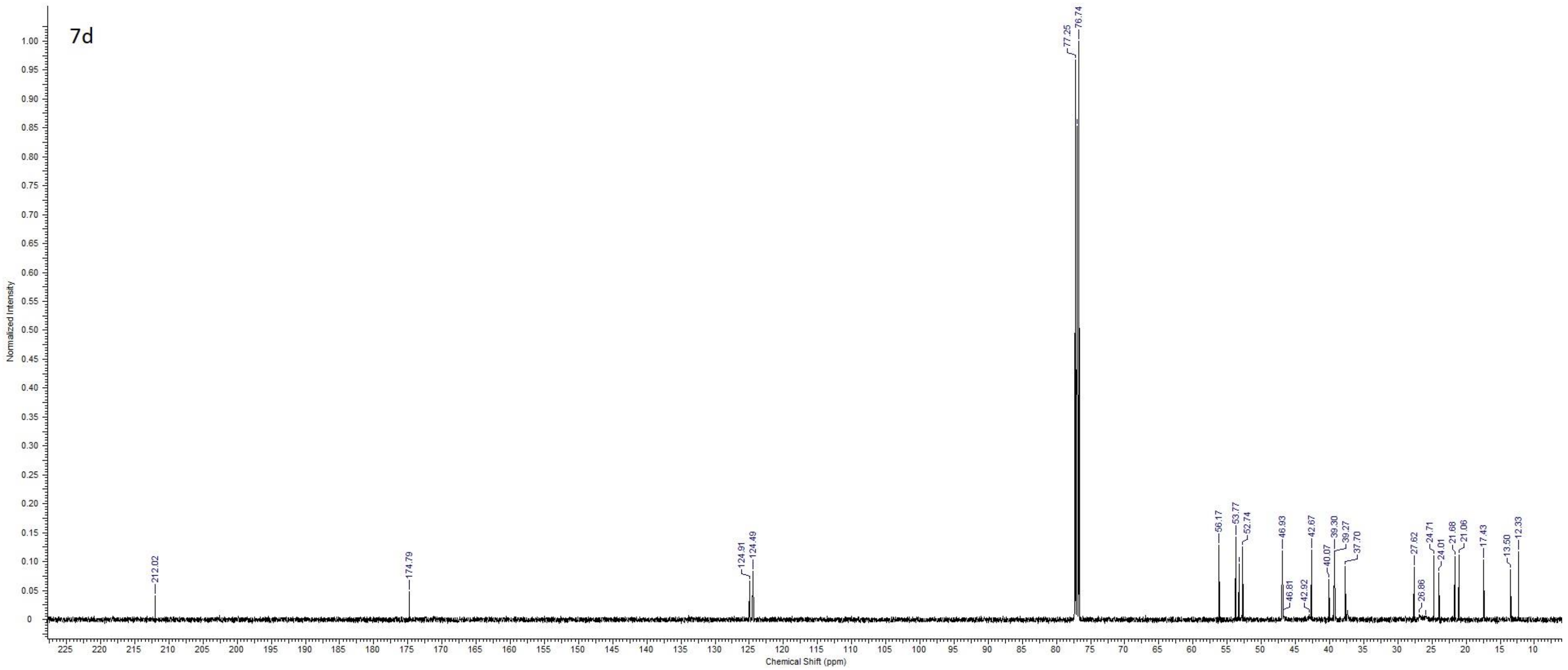
7d
Normalized Intensity
Chemical Shift (ppm)
212.02
174.79
124.91
124.49
77.25
76.74
56.17
53.77
52.74
46.93
46.81
42.92
42.67
40.07
39.30
39.27
37.70
27.62
26.86
24.71
24.01
21.68
21.06
17.43
13.50
12.33

Elemental Composition

File Edit View Process Help

Single Mass Analysis
Tolerance = 1.0 PPM / DBE: min = -1.5, max = 50.0
Element prediction: Off
Number of isotope peaks used for i-FIT = 3
Monoisotopic Mass, Even Electron Ions
535 formula(e) evaluated with 1 results within limits (up to 50 best isotopic matches for each mass)
Elements Used:

Mass	Calc. Mass	mDa	PPM	DBE	Formula	i-FIT	i-FIT Norm	Fit Conf %	C	H	N	O
412.3216	412.3216	0.0	0.0	7.5	C27 H42 N O2	996.5	n/a	n/a	27	42	1	2

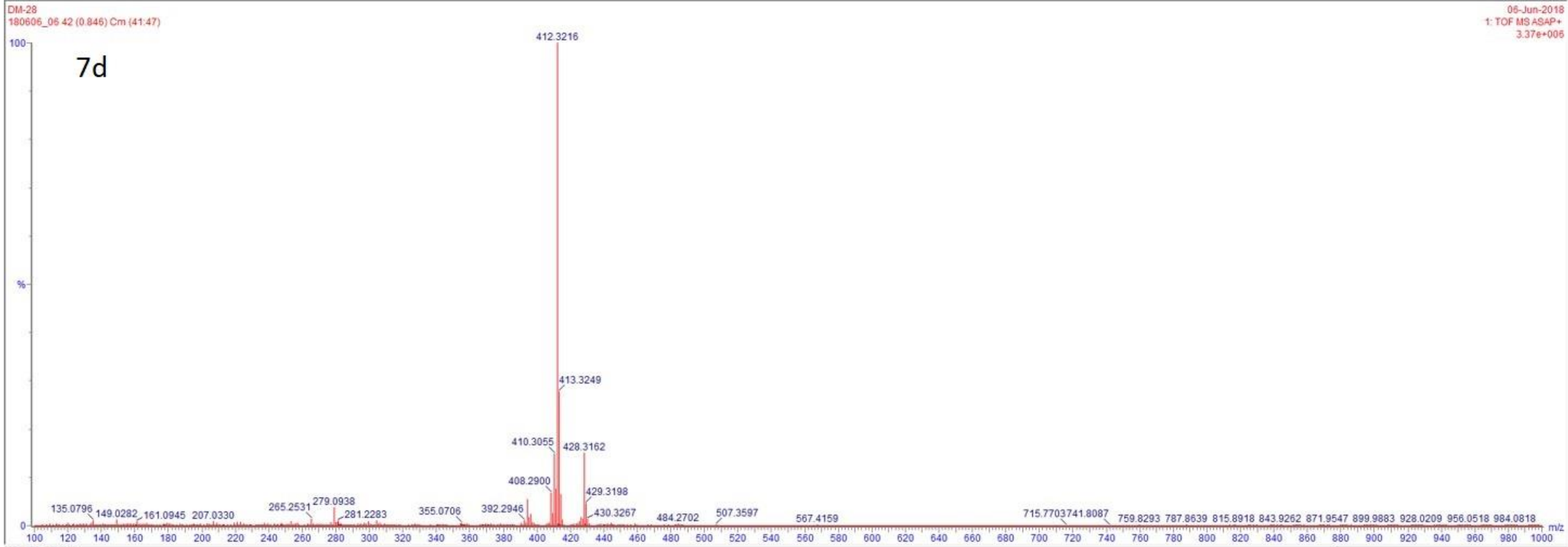

For Help, press F1

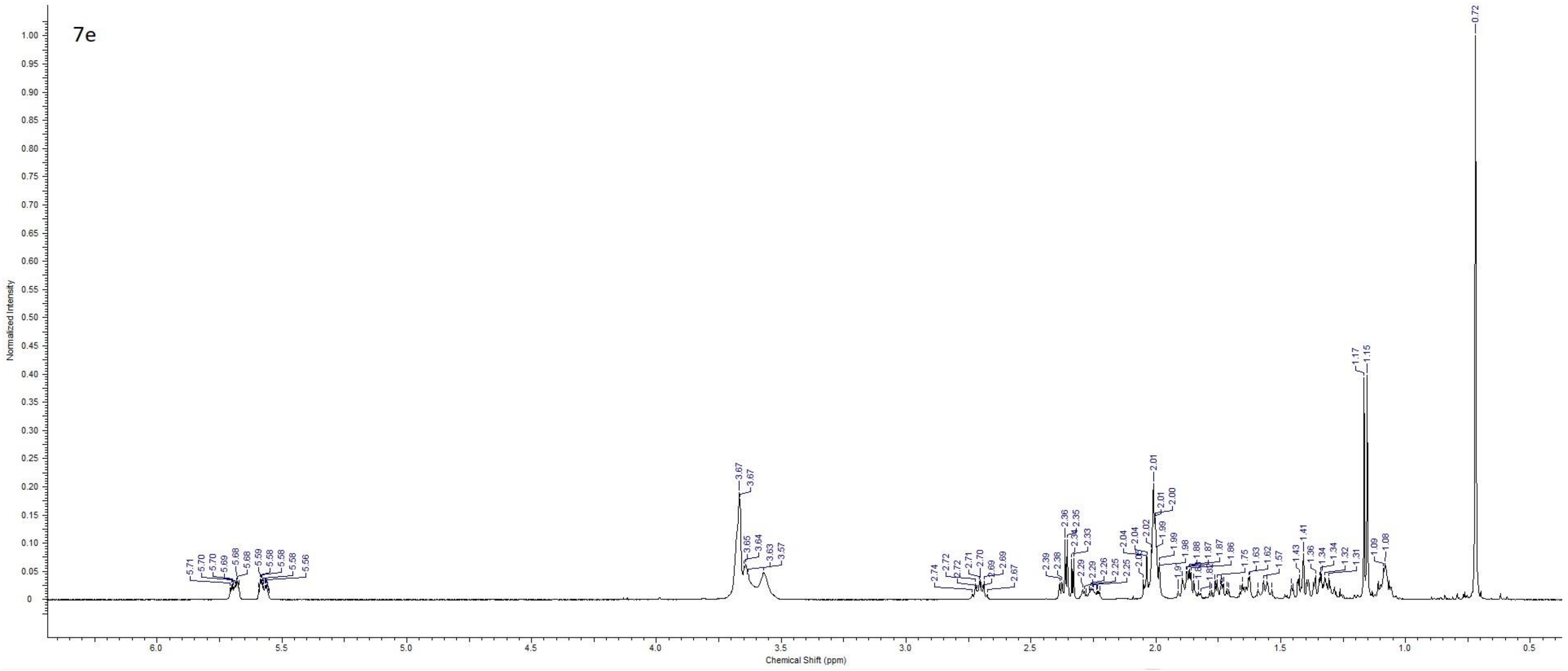
7e
Normalized Intensity
Chemical Shift (ppm)

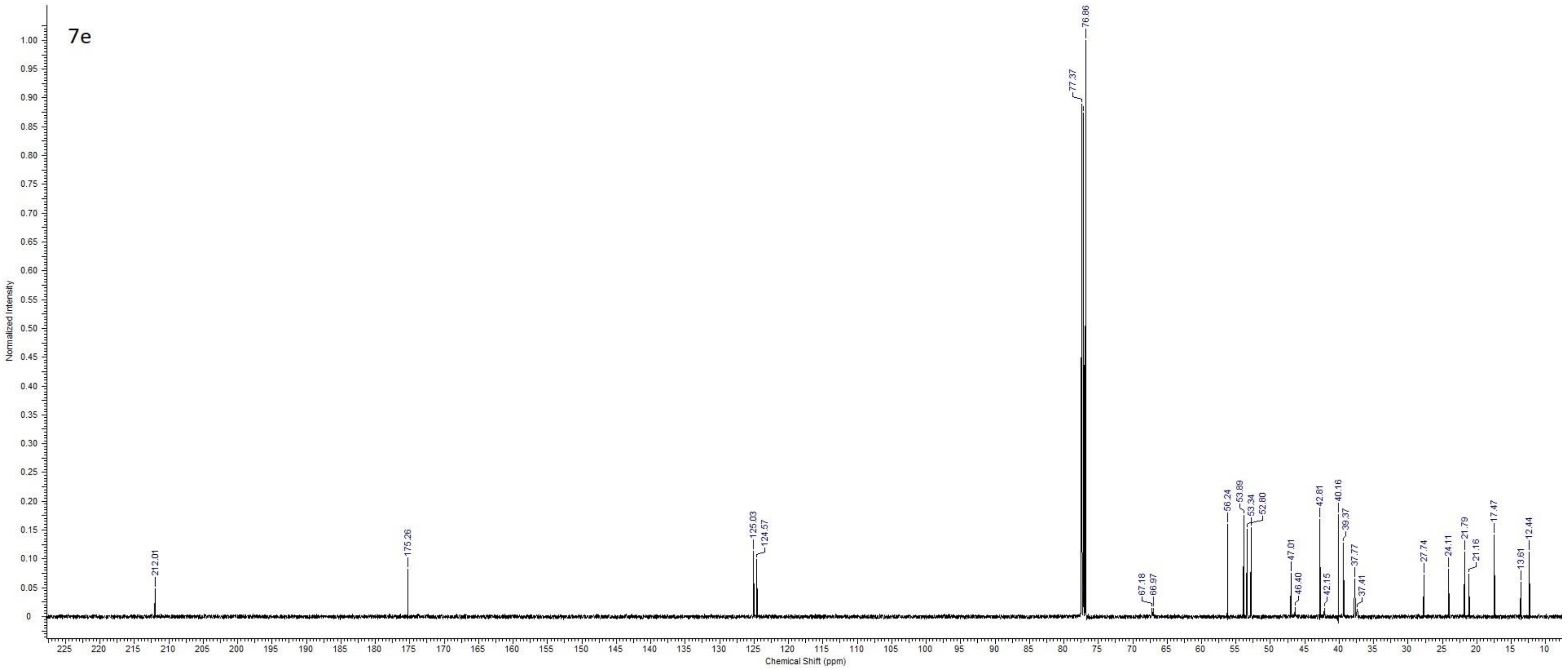

7e
212.01
175.26
125.03
124.57
77.37
76.86
67.18
66.97
56.24
53.89
53.34
52.80
47.01
46.40
42.81
42.15
40.16
39.37
37.77
37.41
27.74
24.11
21.79
21.16
17.47
13.61
12.44
Normalized Intensity
Chemical Shift (ppm)

Elemental Composition

File Edit View Process Help

Single Mass Analysis

Tolerance = 1.0 PPM / DBE: min = -1.5, max = 50.0

Element prediction: Off

Number of isotope peaks used for i-FIT = 3

Monoisotopic Mass, Even Electron Ions

536 formula(e) evaluated with 1 results within limits (up to 50 best isotopic matches for each mass)

Elements Used:

Mass	Calc. Mass	mDa	PPM	DBE	Formula	i-FIT	i-FIT Norm	Fit Conf %	C	H	N	O
414.3012	414.3008	0.4	1.0	7.5	C26 H40 N O3	971.6	n/a	n/a	26	40	1	3

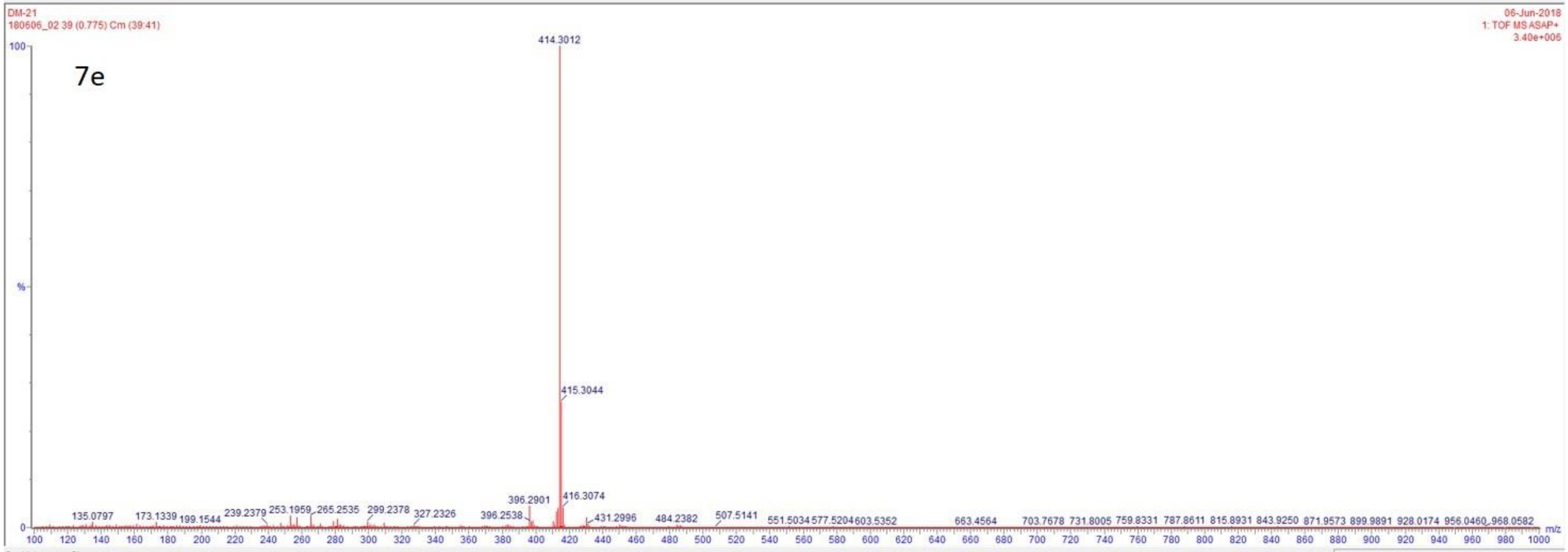

For Help, press F1

8a

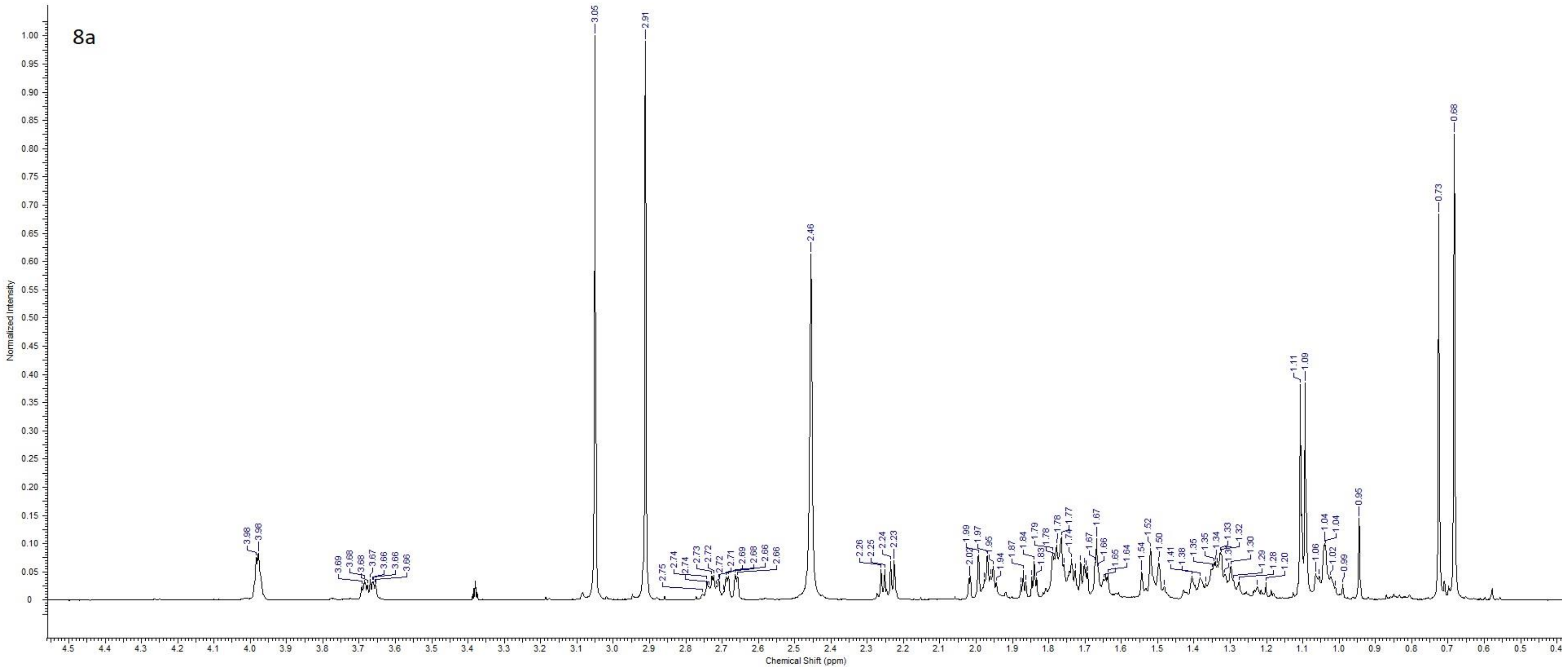
Normalized Intensity
Chemical Shift (ppm)
3.98
3.05
2.91
2.46
1.11
1.09
0.95
0.73
0.68

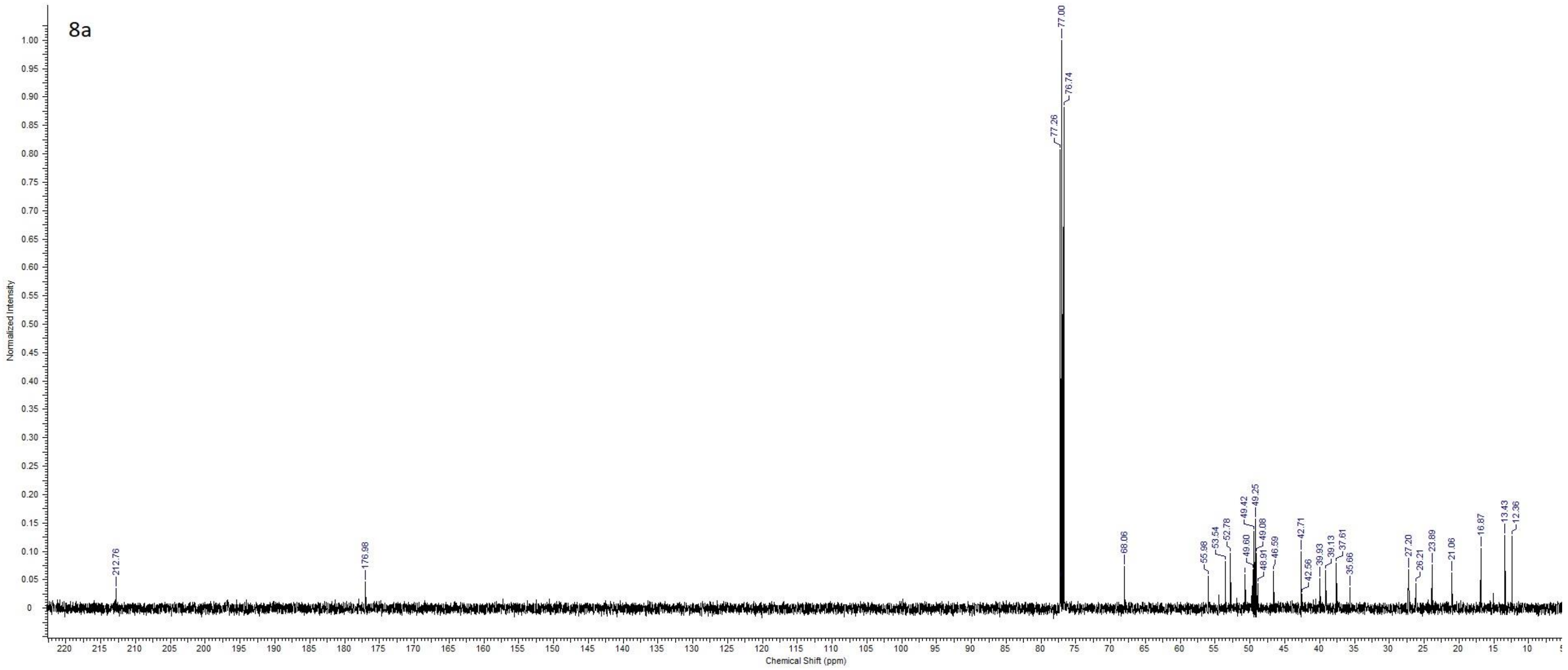
8a
Normalized Intensity
Chemical Shift (ppm)
212.76
176.98
77.26
77.00
76.74
68.06
55.98
53.54
52.78
49.60
49.42
49.25
49.08
48.91
46.59
42.71
42.56
39.93
39.13
37.61
35.66
27.20
26.21
23.89
21.06
16.87
13.43
12.36

Elemental Composition

File Edit View Process Help

Single Mass Analysis

Tolerance = 1.0 PPM / DBE: min = -1.5, max = 50.0

Element prediction: Off

Number of isotope peaks used for i-FIT = 3

Monoisotopic Mass, Even Electron Ions

529 formula(e) evaluated with 1 results within limits (up to 50 best isotopic matches for each mass)

Elements Used:

Mass	Calc. Mass	mDa	PPM	DBE	Formula	i-FIT	i-FIT Norm	Fit Conf %	C	H	N	O
406.2957	406.2957	0.0	0.0	5.5	C24 H40 N O4	972.0	n/a	n/a	24	40	1	4

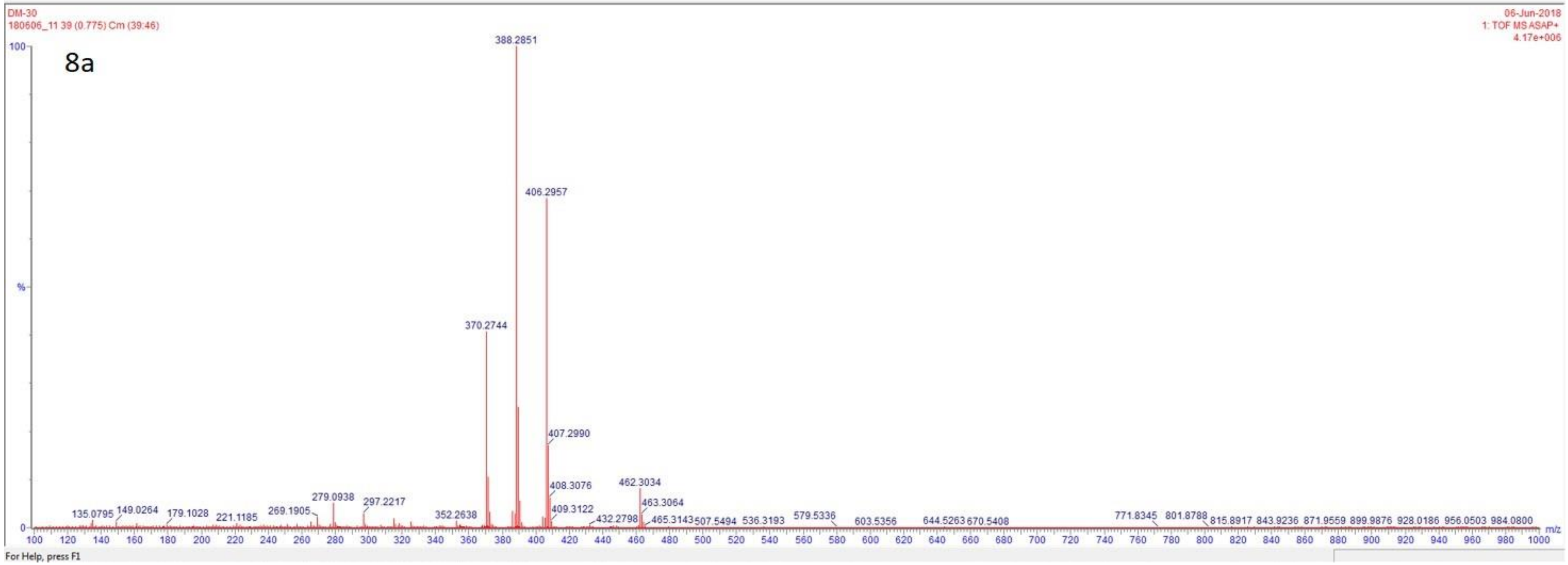

For Help, press F1

8b

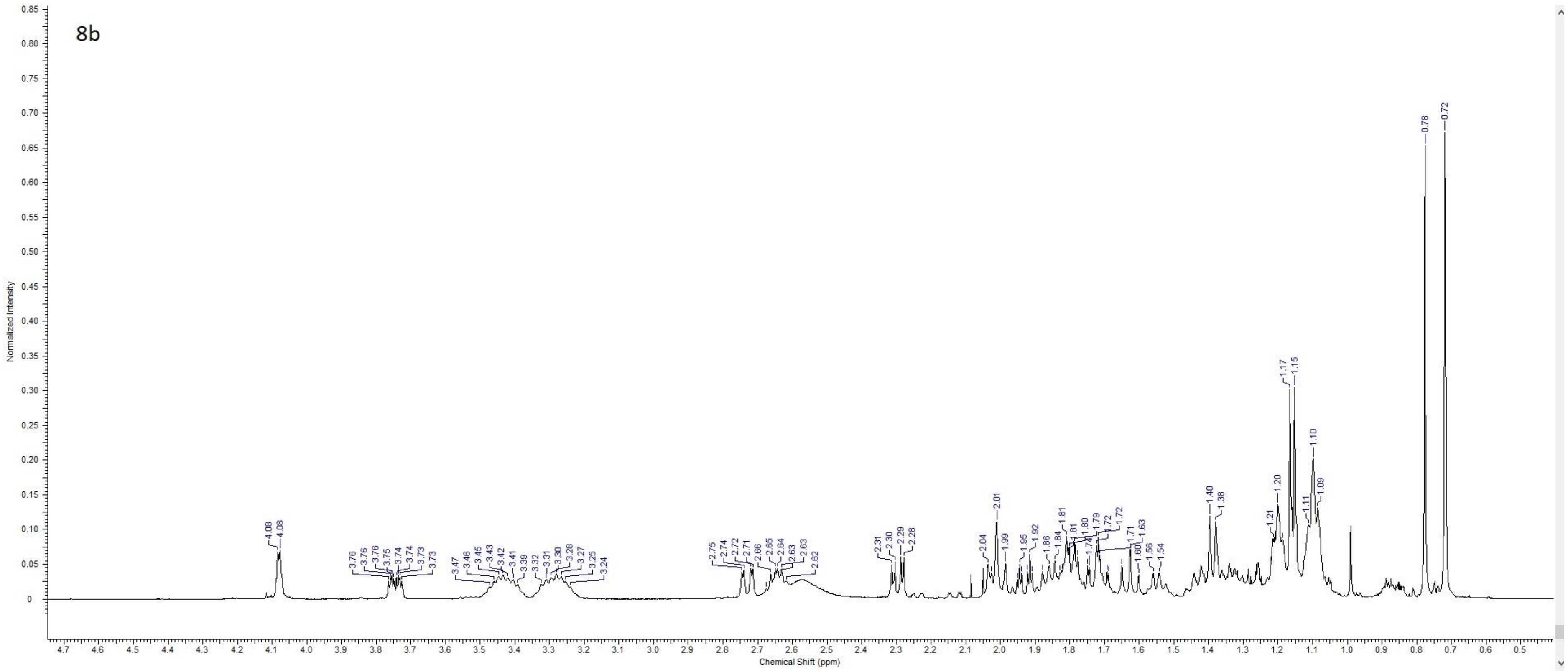

Normalized Intensity
Chemical Shift (ppm)
4.08
4.08
3.76
3.76
3.75
3.74
3.74
3.73
3.73
3.47
3.46
3.45
3.43
3.42
3.41
3.39
3.32
3.31
3.30
3.28
3.27
3.25
3.24
2.75
2.74
2.72
2.71
2.66
2.65
2.64
2.63
2.63
2.62
2.31
2.30
2.29
2.28
2.04
2.01
1.99
1.95
1.92
1.86
1.84
1.81
1.81
1.80
1.79
1.74
1.72
1.72
1.71
1.63
1.60
1.56
1.54
1.40
1.38
1.21
1.20
1.17
1.15
1.11
1.10
1.09
0.78
0.72

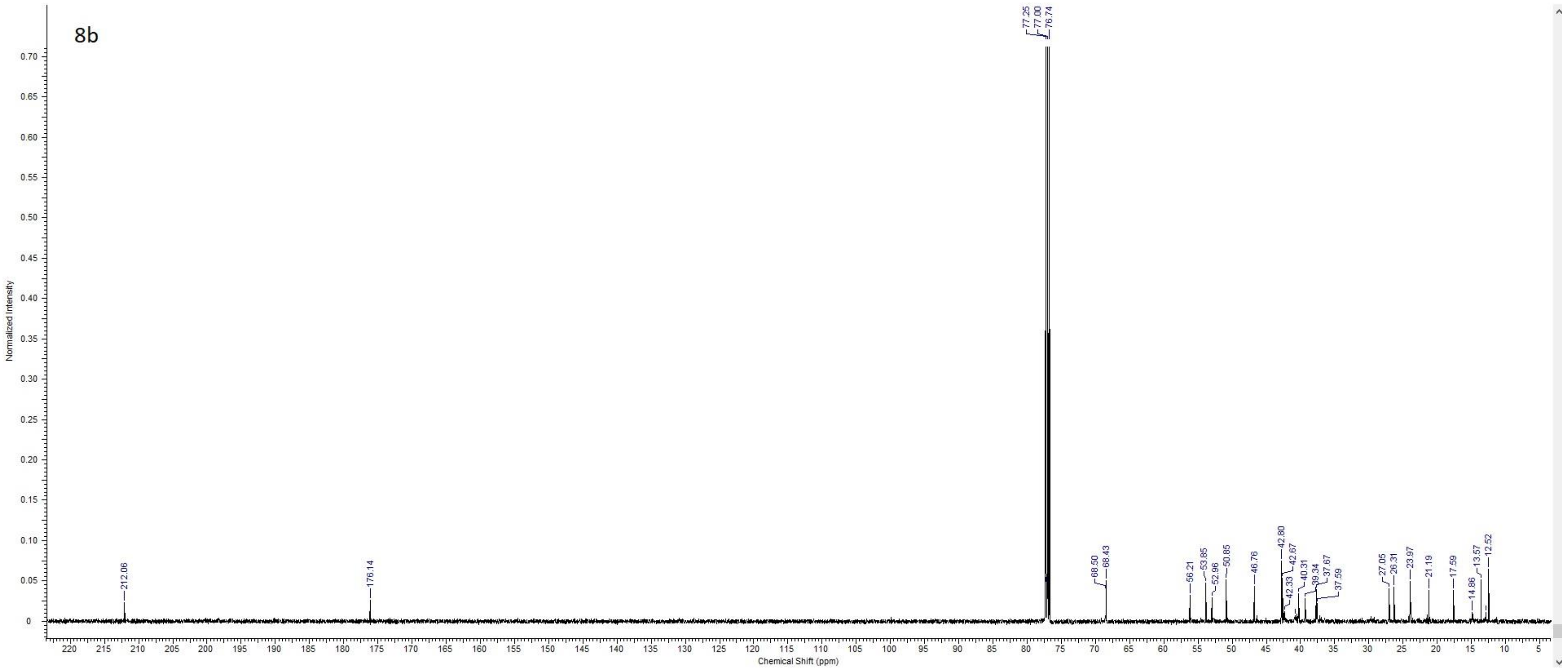
8b
Normalized Intensity
Chemical Shift (ppm)
212.06
176.14
77.25
77.00
76.74
68.50
68.43
56.21
53.85
52.96
50.85
46.76
42.80
42.67
42.33
40.31
39.34
37.67
37.59
27.05
26.31
23.97
21.19
17.59
14.86
13.57
12.52

Elemental Composition

File Edit View Process Help

Single Mass Analysis

Tolerance = 1.0 PPM / DBE: min = -1.5, max = 50.0

Element prediction: Off

Number of isotope peaks used for i-FIT = 3

Monoisotopic Mass, Even Electron Ions

569 formula(e) evaluated with 1 results within limits (up to 50 best isotopic matches for each mass)

Elements Used:

Mass	Calc. Mass	mDa	PPM	DBE	Formula	i-FIT	i-FIT Norm	Fit Conf %	C	H	N	O
434.3270	434.3270	0.0	0.0	5.5	C26 H44 N O4	904.6	n/a	n/a	26	44	1	4

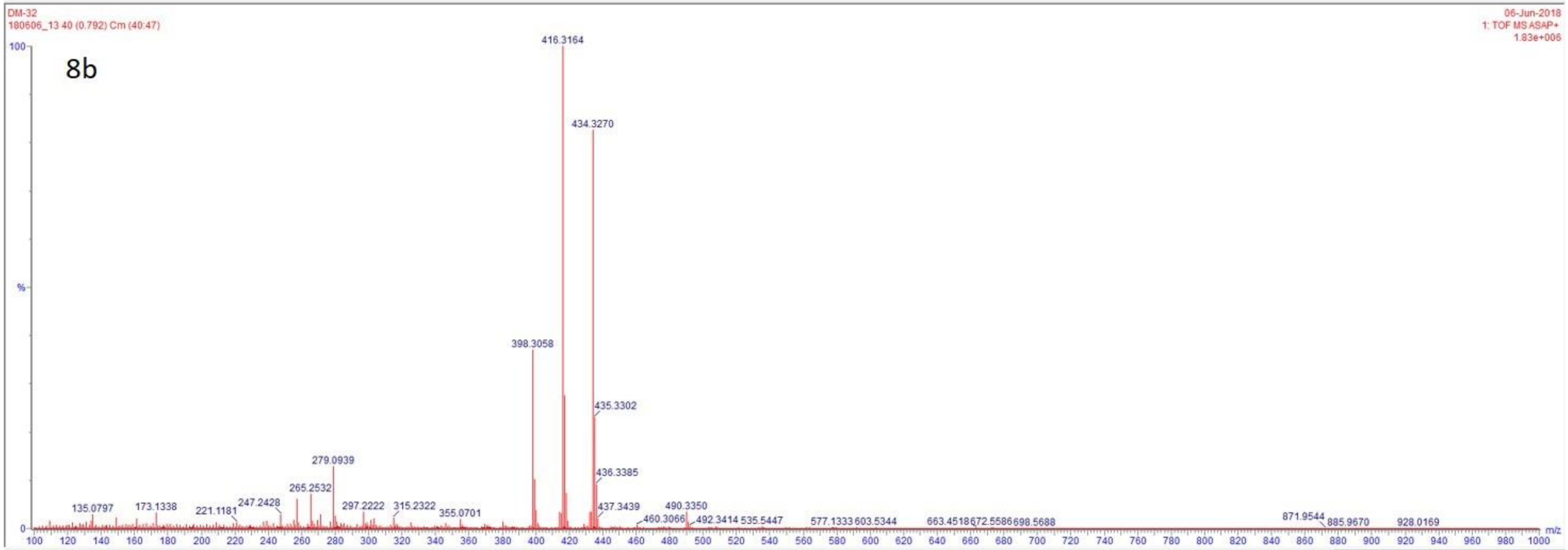

For Help, press F1

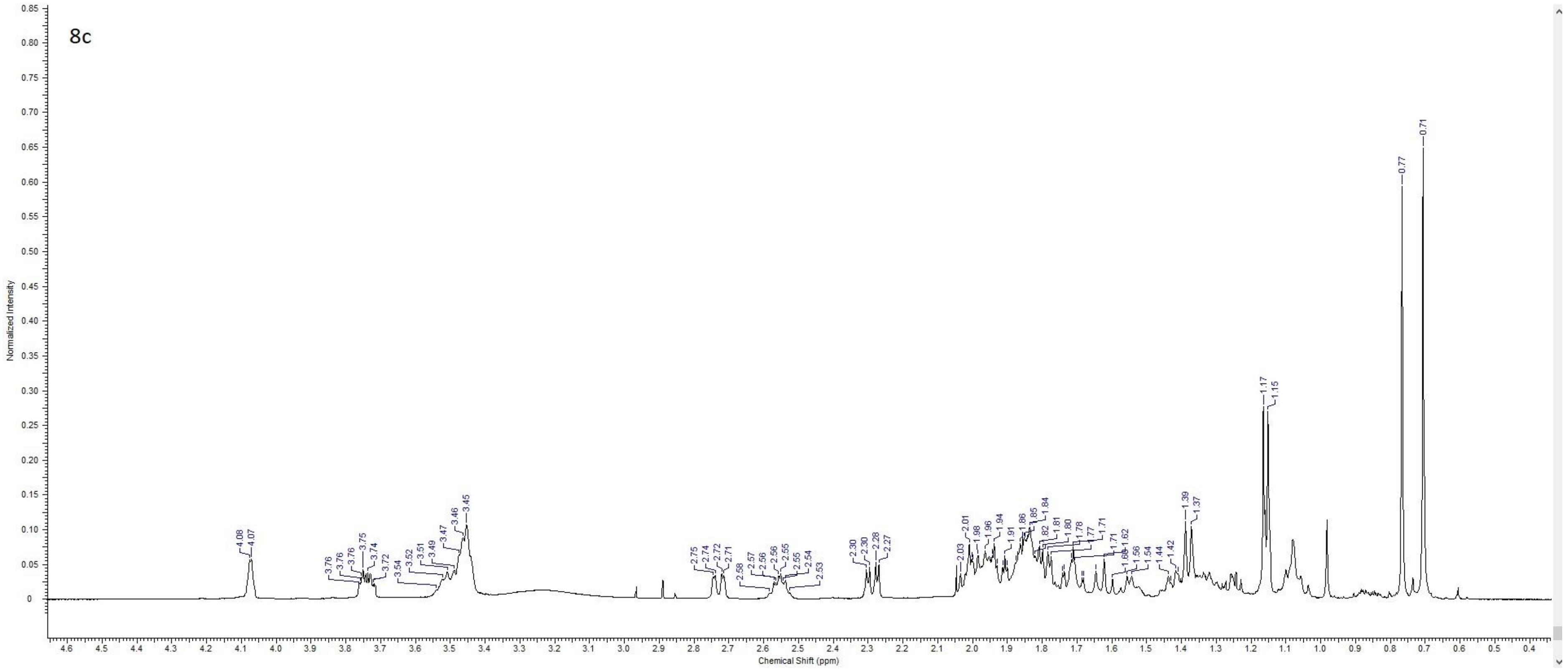
8c
Normalized Intensity
Chemical Shift (ppm)

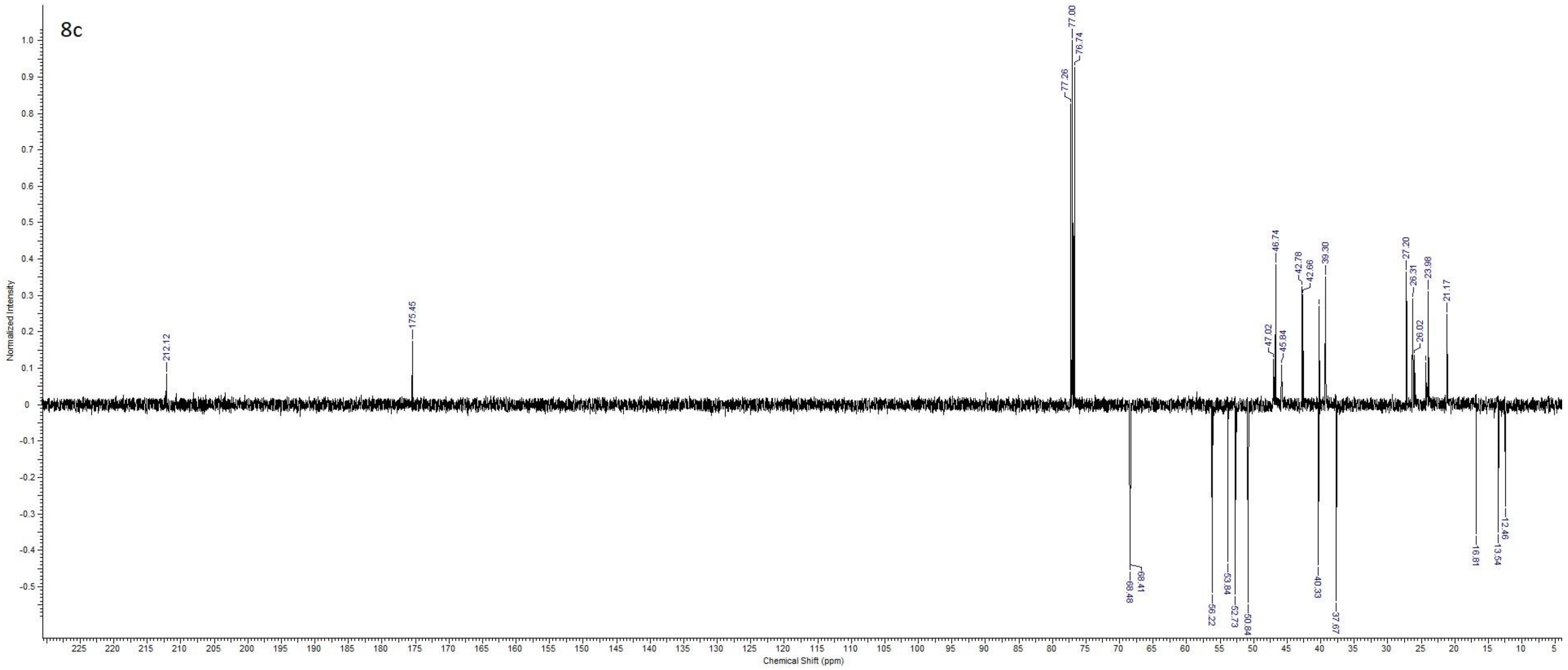
8c
Normalized Intensity
Chemical Shift (ppm)
212.12
175.45
77.26
77.00
76.74
68.41
68.48
56.22
53.84
52.73
50.84
47.02
46.74
45.84
42.78
42.66
40.33
39.30
37.67
27.20
26.31
26.02
23.98
21.17
16.81
13.54
12.46

Elemental Composition

File Edit View Process Help

Single Mass Analysis

Tolerance = 1.0 PPM / DBE: min = -1.5, max = 50.0

Element prediction: Off

Number of isotope peaks used for i-FIT = 3

Monoisotopic Mass, Even Electron Ions

566 formula(e) evaluated with 1 results within limits (up to 50 best isotopic matches for each mass)

Elements Used:

Mass	Calc. Mass	mDa	PPM	DBE	Formula	i-FIT	i-FIT Norm	Fit Conf %	C	H	N	O
432.3116	432.3114	0.2	0.5	6.5	C26 H42 N O4	920.7	n/a	n/a	26	42	1	4

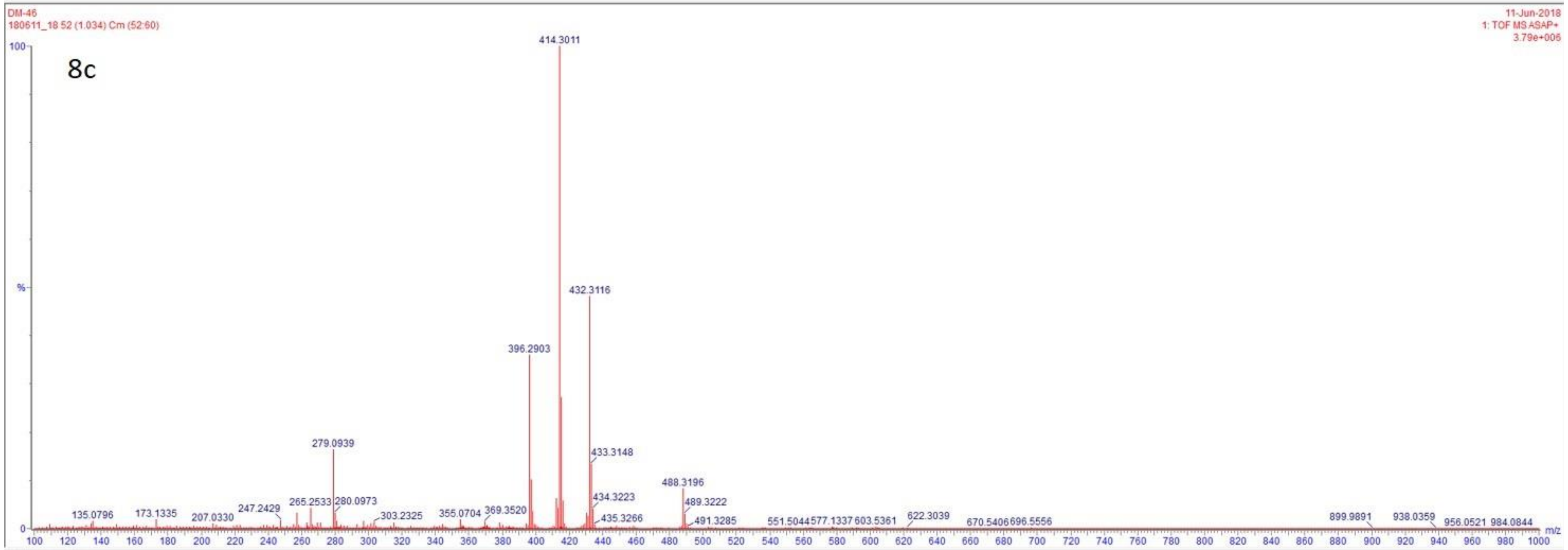

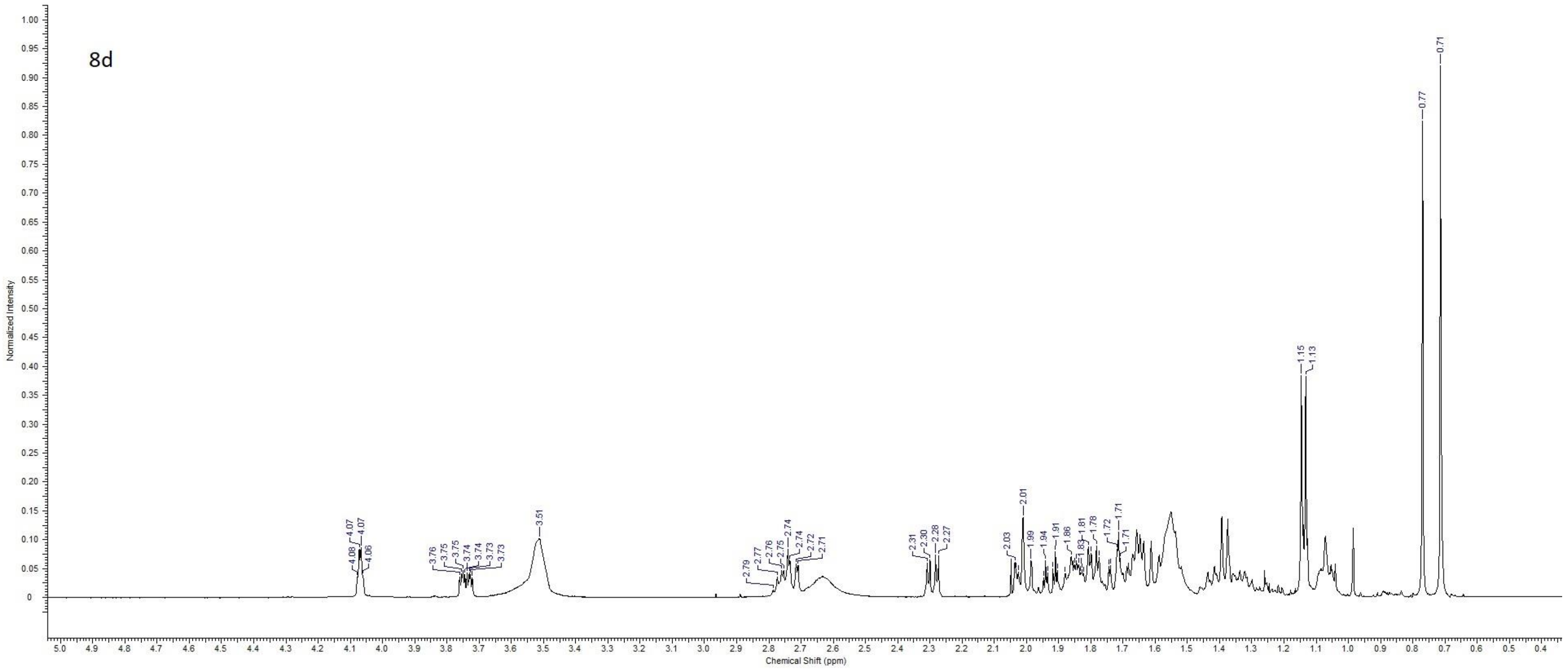
8d
Normalized Intensity
Chemical Shift (ppm)

8d

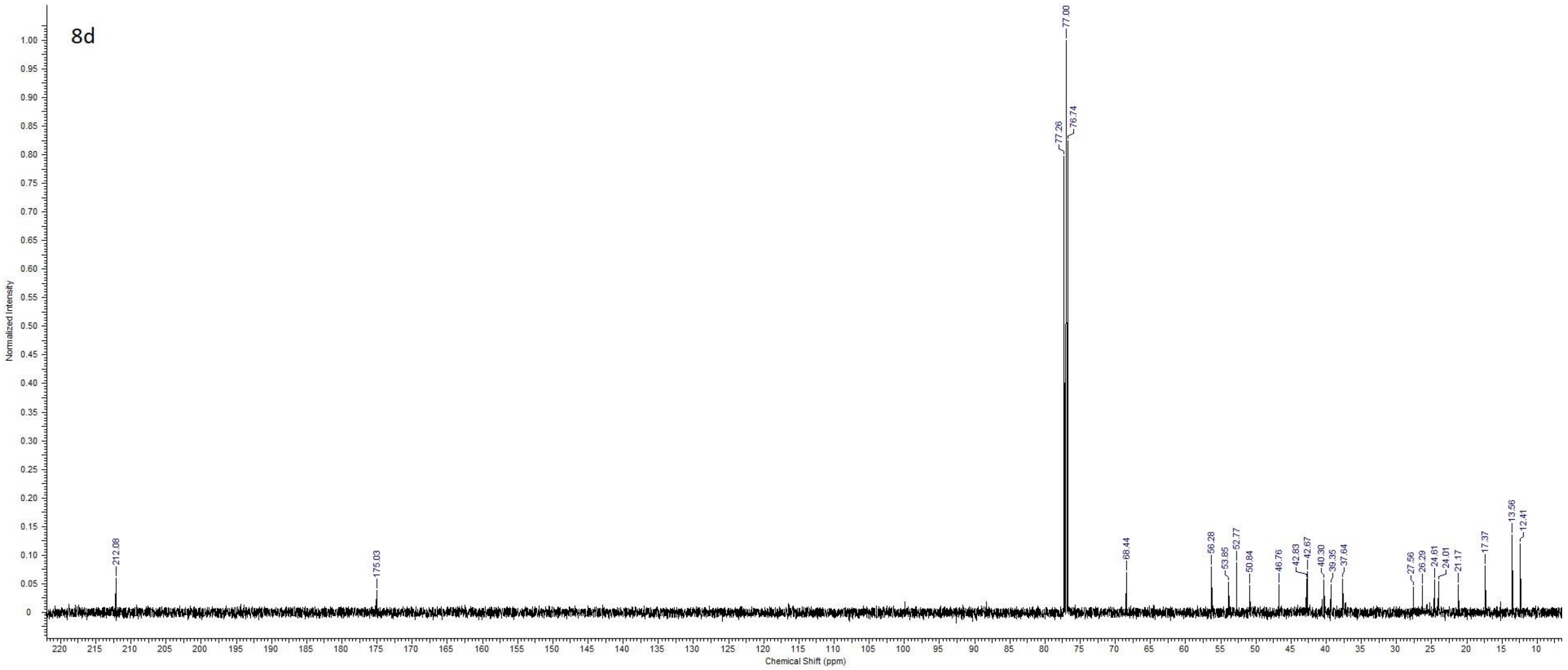

212.08
175.03
77.26
77.00
76.74
68.44
56.28
53.85
52.77
50.84
46.76
42.83
42.67
40.30
39.35
37.64
27.56
26.29
24.61
24.01
21.17
17.37
13.56
12.41
Normalized Intensity
Chemical Shift (ppm)

8d

Qualitative Compound Report

**Data File**	201119_DM-33_01.d	**Sample Name**	DM-33
**Sample Type**	Sample	**Position**	P1-A2
**Instrument Name**	G6230B TOF	**User Name**	
**Acq Method**	HRMS_Jakub_ref.m	**Acquired Time**	19-Nov-20 11:58:41 AM
**IRM Calibration Status**	Success	**DA Method**	test.m
**Comment**			
**Sample Group**		**Info.**	
**Stream Name**		**Acquisition SW Version**	6200 series TOF/6500 series Q-TOF B.09.00 (B9044.1 SP1)

Compound Table

Compound Label	RT	Mass	Abund	Formula	Tgt Mass	Diff (ppm)
Cpd 1: C27 H43 N O4	6.13	445.3199	1129405	C27 H43 N O4	445.3192	1.65

Compound Label	m/z	RT	Algorithm	Mass
Cpd 1: C27 H43 N O4	446.3271	6.13	Find By Formula	445.3199

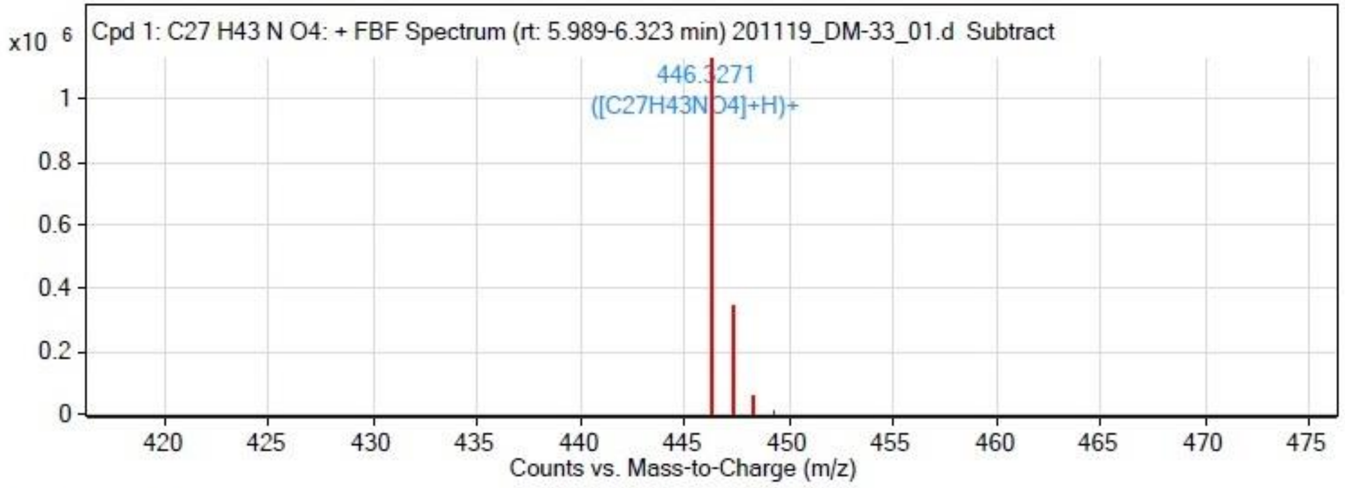

MS Spectrum Peak List

m/z	z	Abund	Formula	Ion
446.3271	1	1129404.63	C27H43NO4	(M+H)+
447.3308	1	343660.72	C27H43NO4	(M+H)+
448.3339	1	60660.51	C27H43NO4	(M+H)+
449.336	1	9387.19	C27H43NO4	(M+H)+

--- End Of Report ---

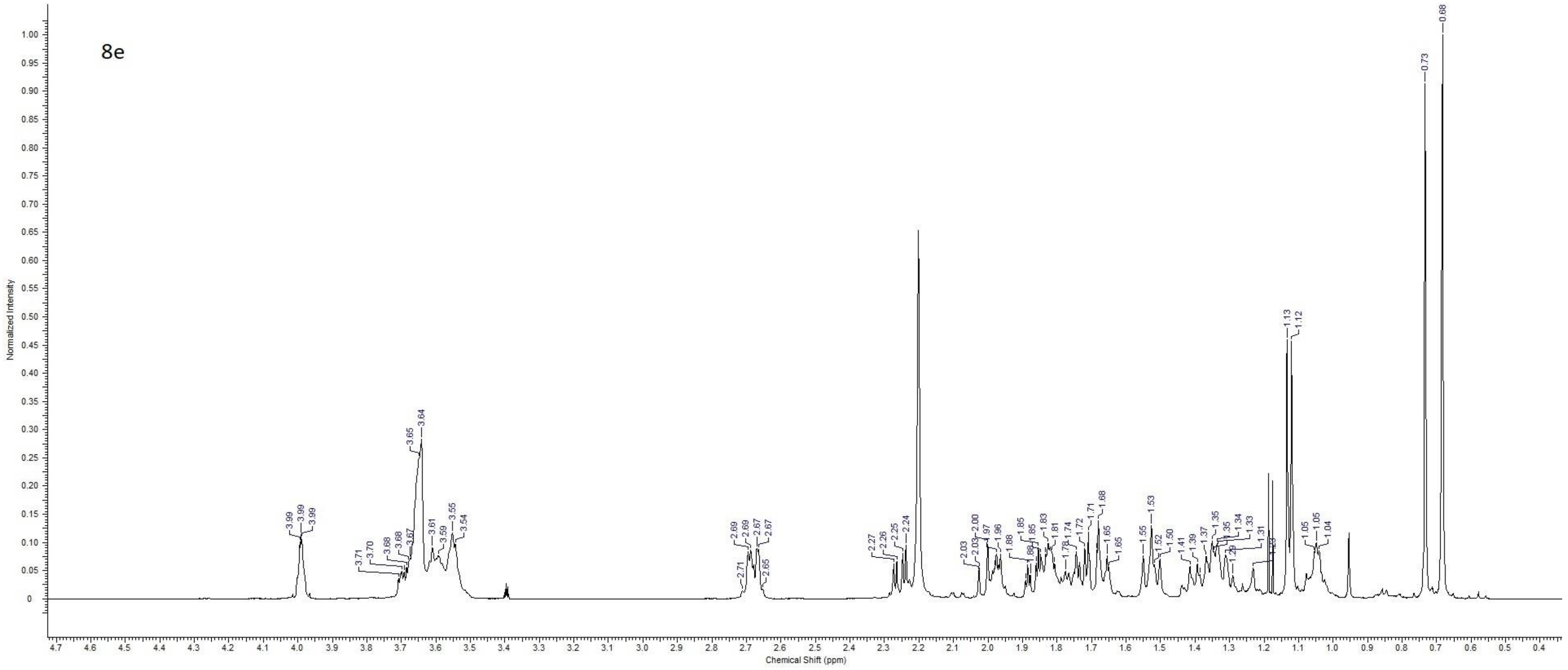
8e
Normalized Intensity
Chemical Shift (ppm)

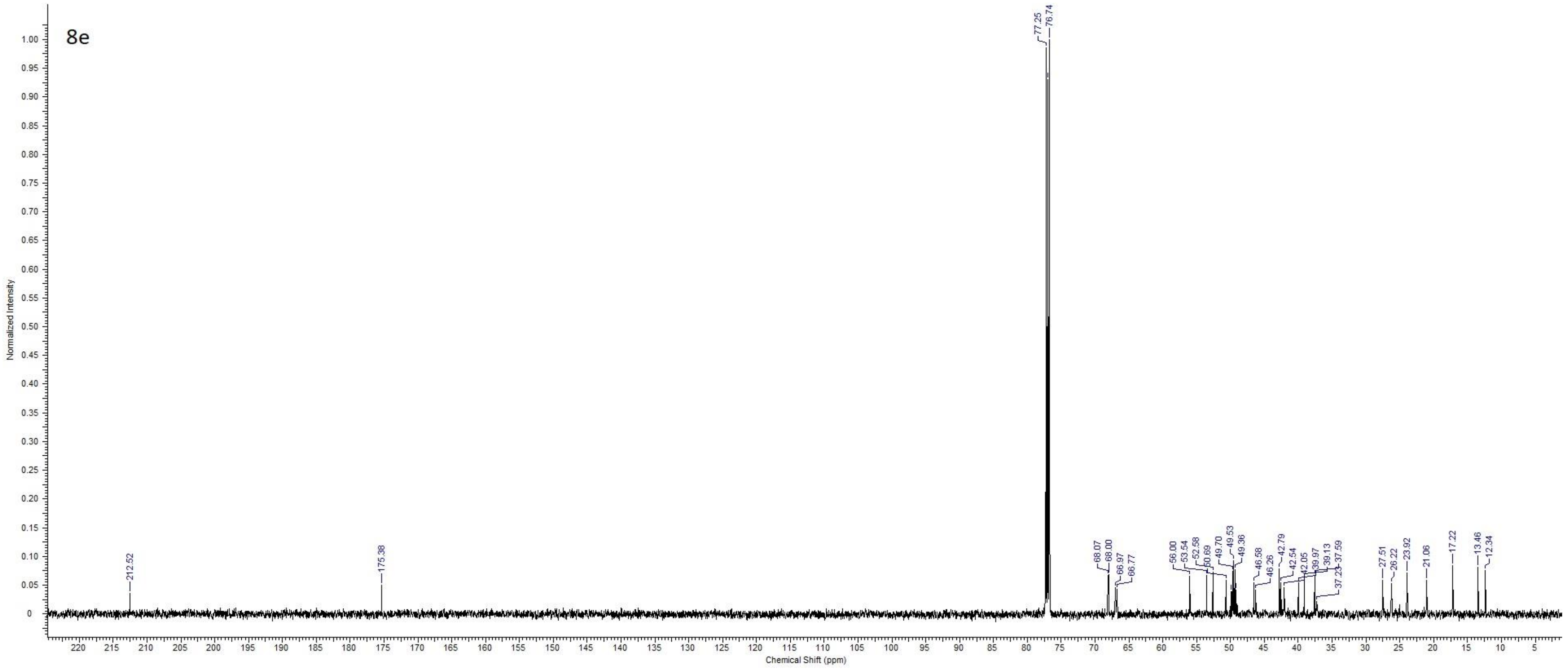
8e
Normalized Intensity
Chemical Shift (ppm)
212.52
175.38
77.25
76.74
68.07
68.00
66.97
66.77
56.00
53.54
52.58
50.69
49.70
49.53
49.36
46.58
46.26
42.79
42.54
42.05
39.97
39.13
37.59
37.23
27.51
26.22
23.92
21.06
17.22
13.46
12.34

Elemental Composition

File Edit View Process Help

Single Mass Analysis

Tolerance = 1.0 PPM / DBE: min = -1.5, max = 50.0

Element prediction: Off

Number of isotope peaks used for i-FIT = 3

Monoisotopic Mass, Even Electron Ions

591 formula(e) evaluated with 1 results within limits (up to 50 best isotopic matches for each mass)

Elements Used:

Mass	Calc. Mass	mDa	PPM	DBE	Formula	i-FIT	i-FIT Norm	Fit Conf %	C	H	N	O
448.3063	448.3063	0.0	0.0	6.5	C26 H42 N O5	872.6	n/a	n/a	26	42	1	5

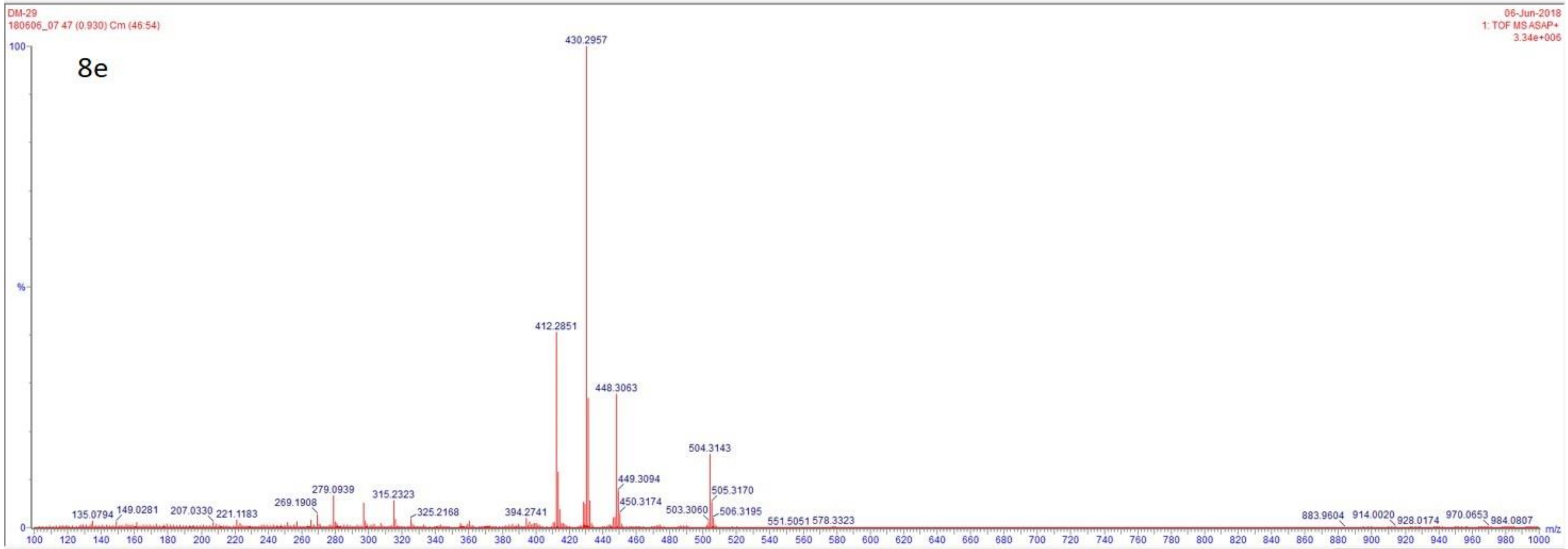

For Help, press F1

11

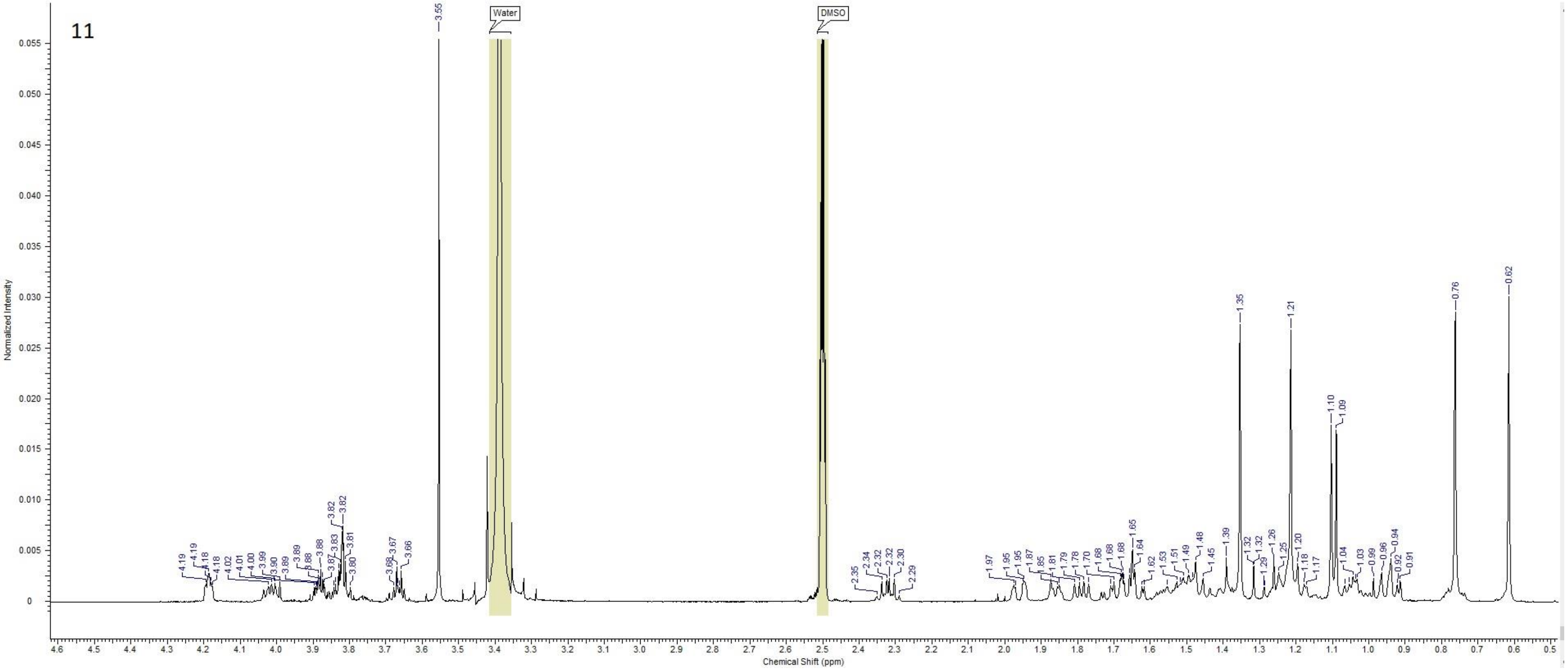

11

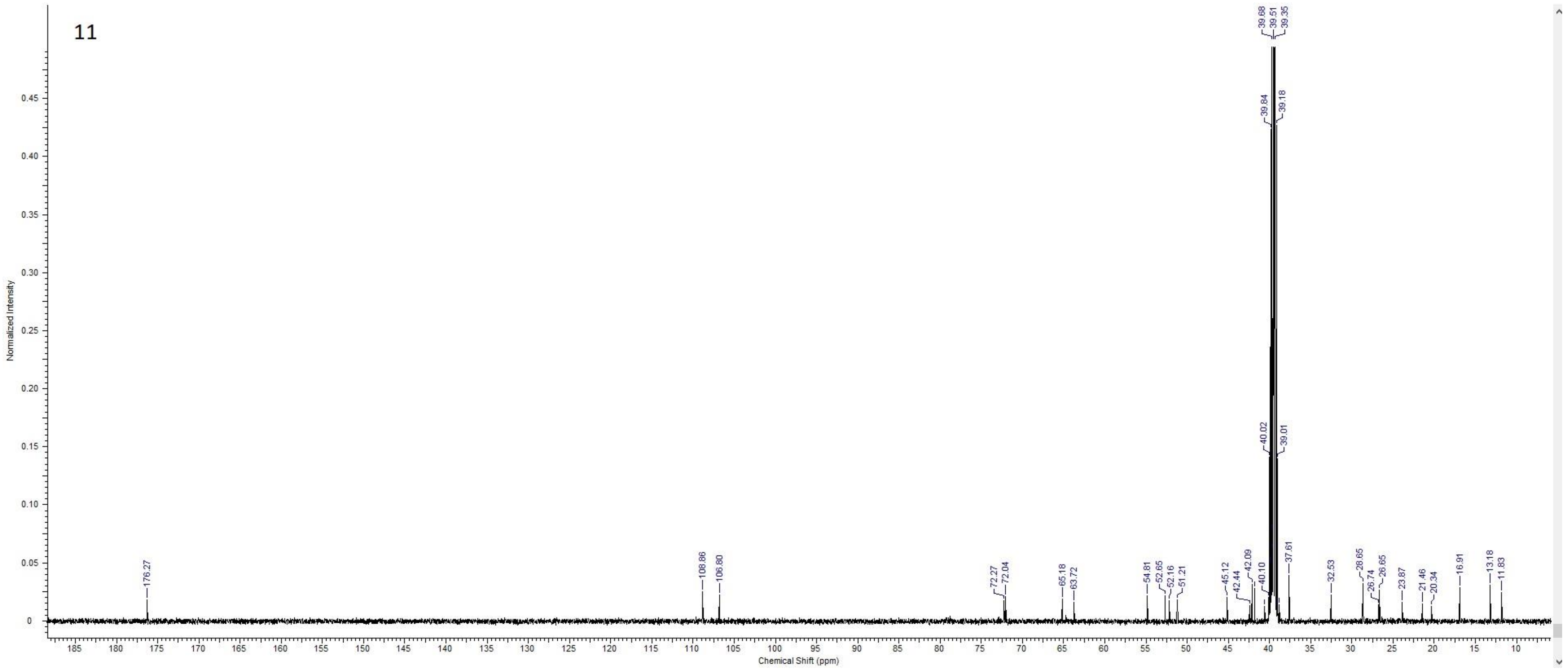
176.27
108.86
106.80
72.27
72.04
65.18
63.72
54.81
52.65
52.16
51.21
45.12
42.44
42.09
40.10
40.02
39.84
39.68
39.51
39.35
39.18
39.01
37.61
32.53
28.65
26.74
26.65
23.87
21.46
20.34
16.91
13.18
11.83
Normalized Intensity
Chemical Shift (ppm)

Elemental Composition

File Edit View Process Help

Single Mass Analysis

Tolerance = 1.0 PPM / DBE: min = -1.5, max = 50.0

Element prediction: Off

Number of isotope peaks used for i-FIT = 3

Monoisotopic Mass, Even Electron Ions

642 formula(e) evaluated with 1 results within limits (up to 50 best isotopic matches for each mass)

Elements Used:

Mass	Calc. Mass	mDa	PPM	DBE	Formula	i-FIT	i-FIT Norm	Fit Conf %	C	H	N	O
477.3220	477.3216	0.4	0.8	6.5	C28 H45 O6	886.7	n/a	n/a	28	45		6

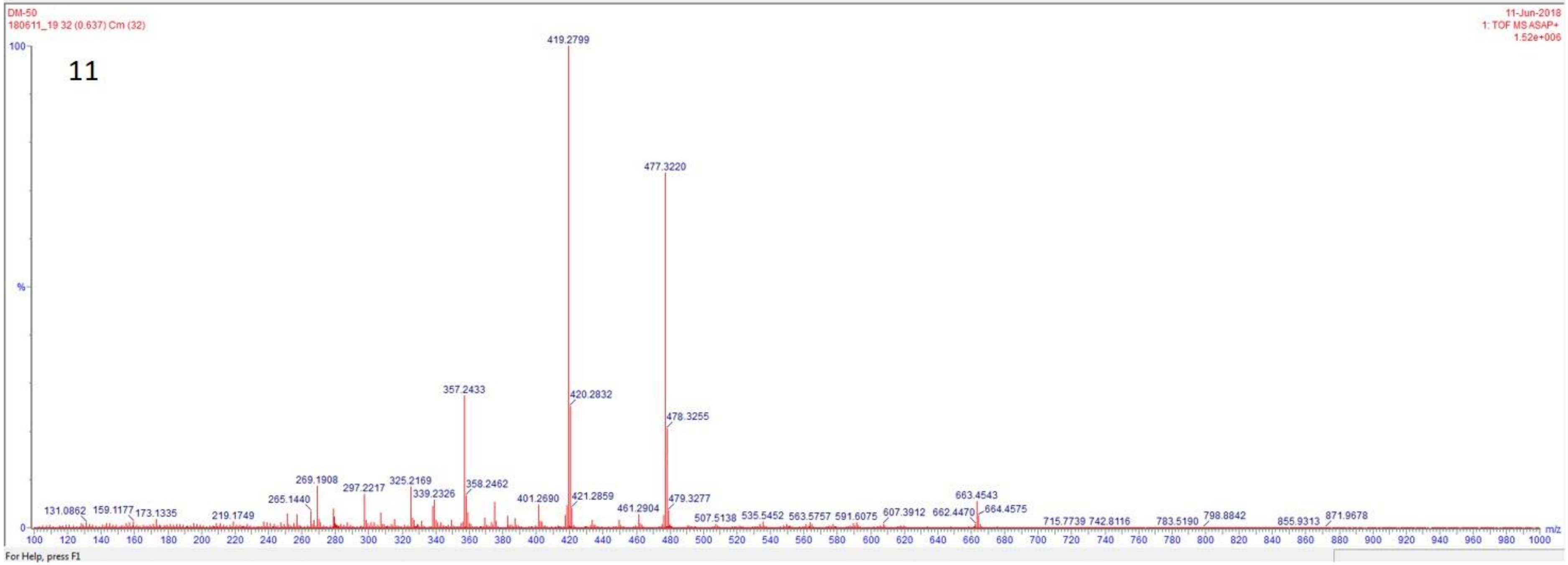

For Help, press F1

12

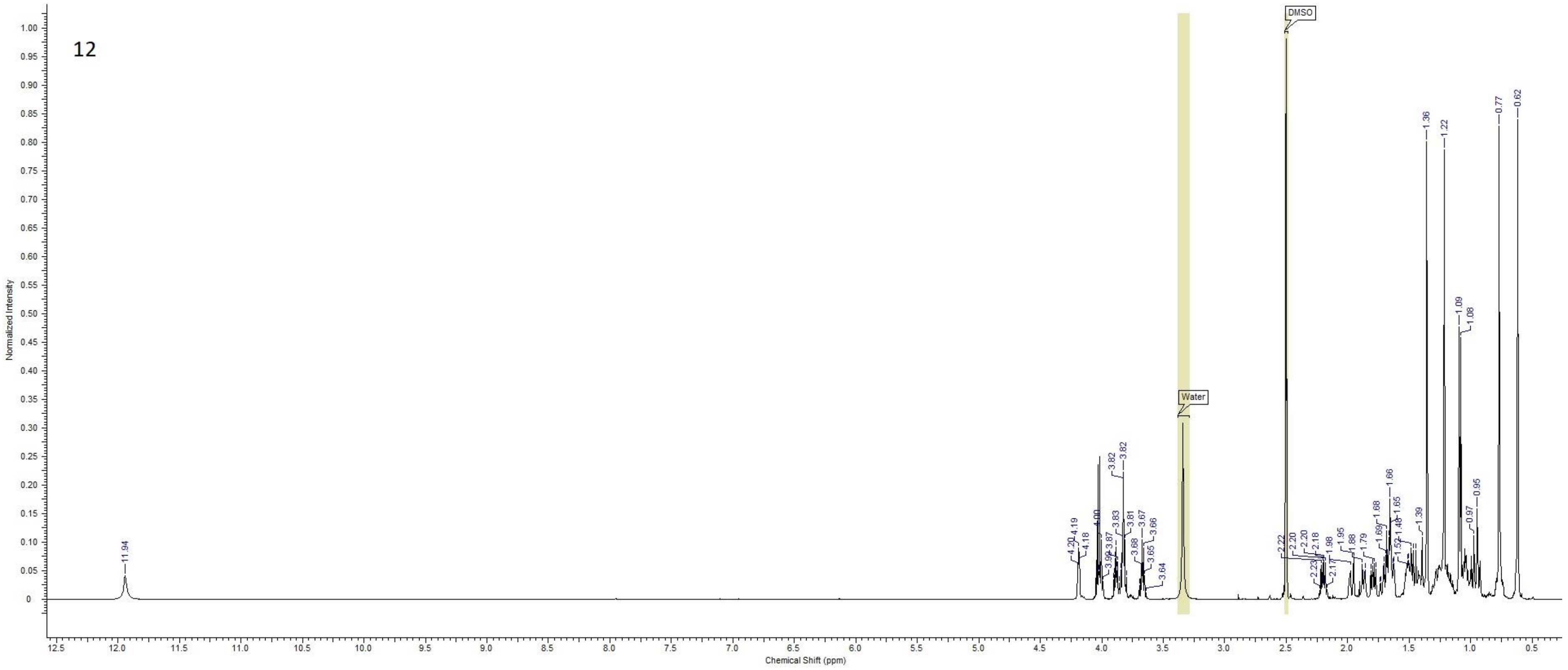

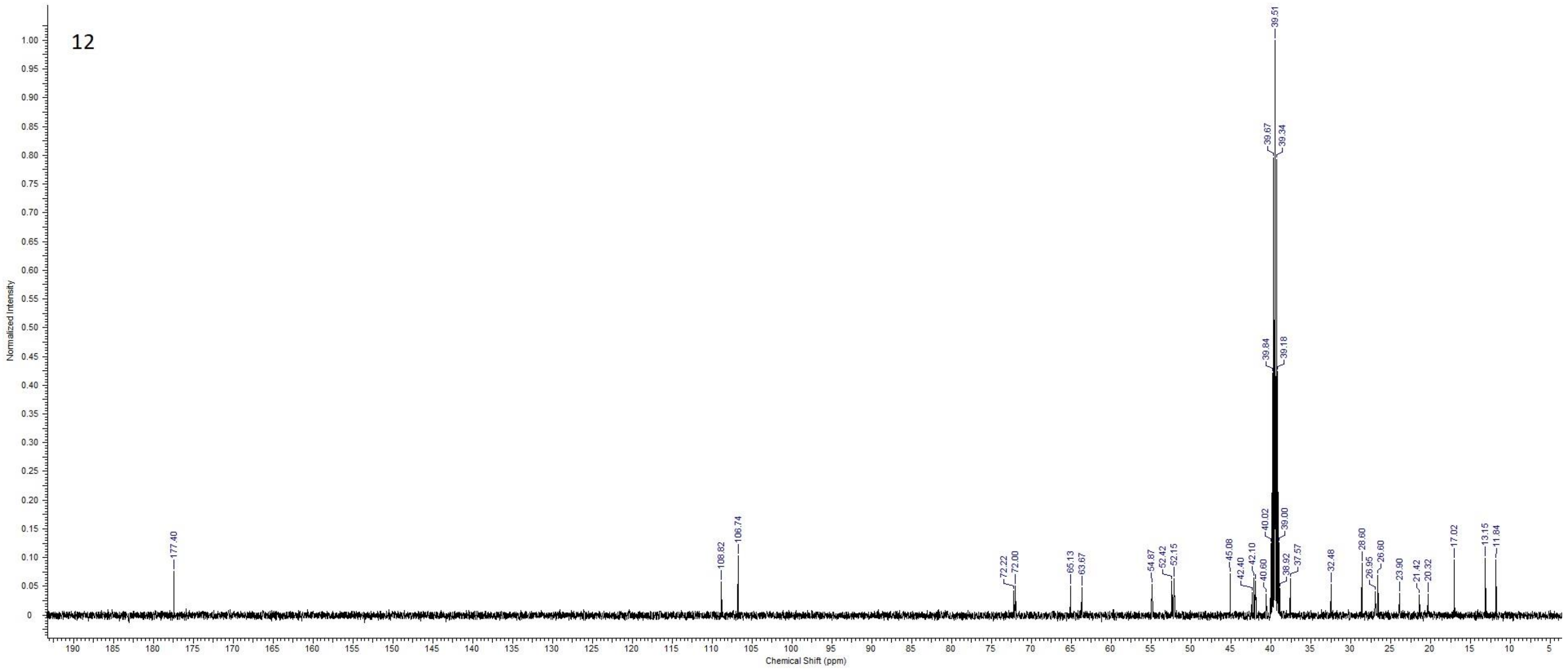
12
Normalized Intensity
Chemical Shift (ppm)
177.40
108.82
106.74
72.22
72.00
65.13
63.67
54.87
52.42
52.15
45.08
42.40
42.10
40.60
40.02
39.84
39.67
39.51
39.34
39.18
39.00
38.92
37.57
32.48
28.60
26.95
26.60
23.90
21.42
20.32
17.02
13.15
11.84

Elemental Composition

File Edit View Process Help

Single Mass Analysis

Tolerance = 1.0 PPM / DBE: min = -1.5, max = 50.0

Element prediction: Off

Number of isotope peaks used for i-FIT = 3

Monoisotopic Mass, Even Electron Ions

624 formula(e) evaluated with 1 results within limits (up to 50 best isotopic matches for each mass)

Elements Used:

Mass	Calc. Mass	mDa	PPM	DBE	Formula	i-FIT	i-FIT Norm	Fit Conf %	C	H	N	O
463.3059	463.3060	-0.1	-0.2	6.5	C27 H43 O6	883.8	n/a	n/a	27	43		6

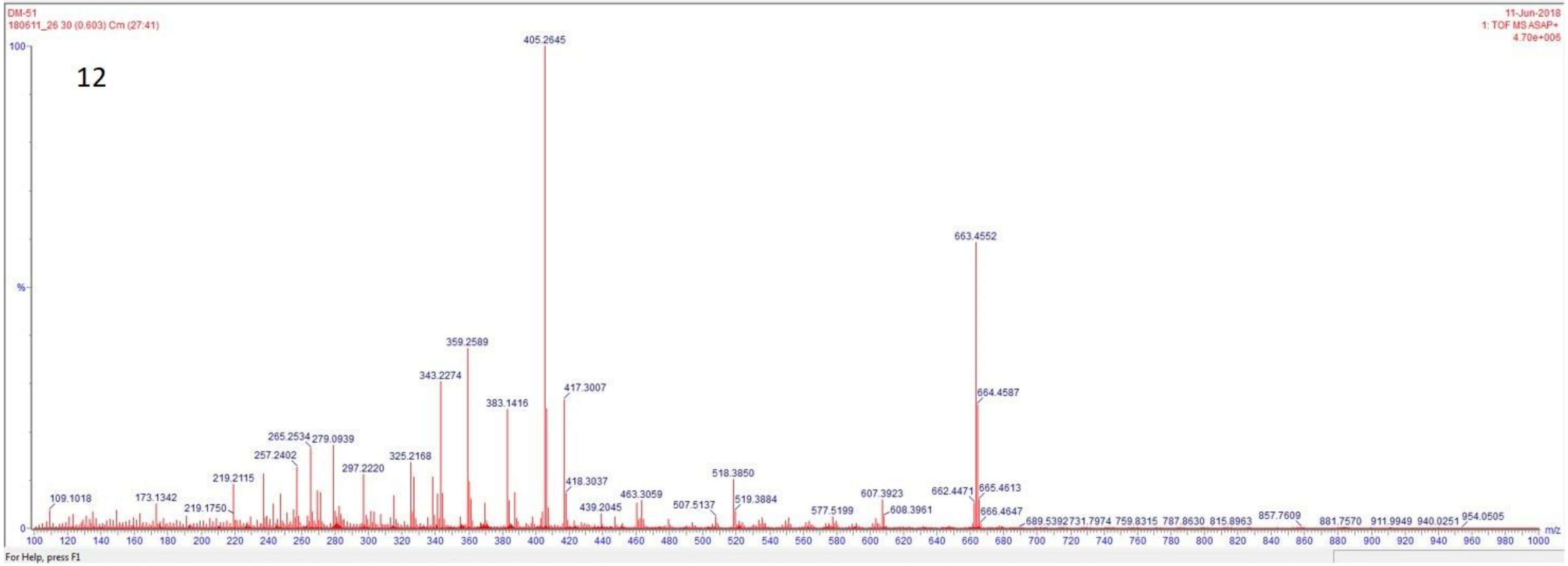

For Help, press F1

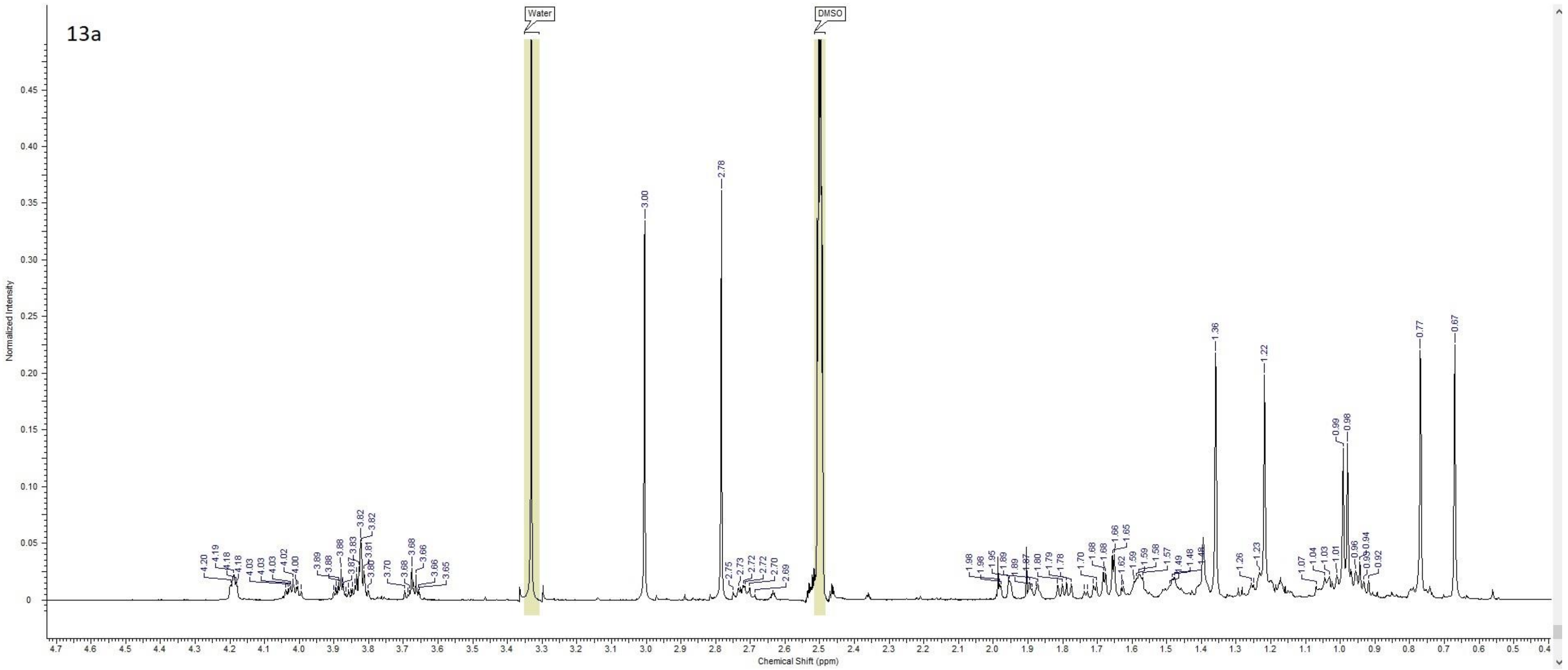

13a
Water
DMSO
Normalized Intensity
Chemical Shift (ppm)
3.00
2.78
1.36
1.22
0.77
0.67
0.99
0.98

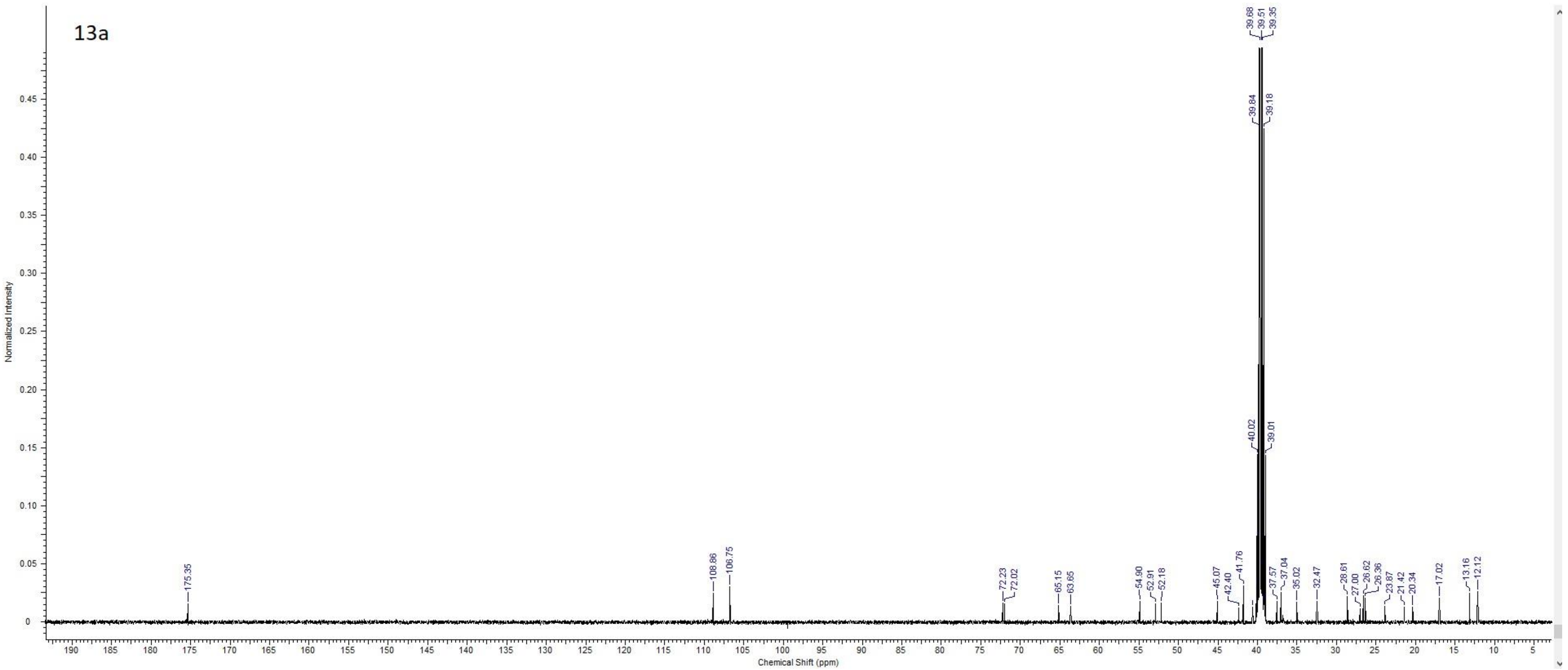
13a
Normalized Intensity
Chemical Shift (ppm)
175.35
108.86
106.75
72.23
72.02
65.15
63.65
54.90
52.91
52.18
45.07
42.40
41.76
40.02
39.84
39.68
39.51
39.35
39.18
39.01
37.57
37.04
35.02
32.47
28.61
27.00
26.62
26.36
23.87
21.42
20.34
17.02
13.16
12.12

Elemental Composition

File Edit View Process Help

Single Mass Analysis

Tolerance = 1.0 PPM / DBE: min = -1.5, max = 50.0

Element prediction: Off

Number of isotope peaks used for i-FIT = 3

Monoisotopic Mass, Even Electron Ions

650 formula(e) evaluated with 1 results within limits (up to 50 best isotopic matches for each mass)

Elements Used:

Mass	Calc. Mass	mDa	PPM	DBE	Formula	i-FIT	i-FIT Norm	Fit Conf %	C	H	N	O
490.3534	490.3532	0.2	0.4	6.5	C29 H48 N O5	896.8	n/a	n/a	29	48	1	5

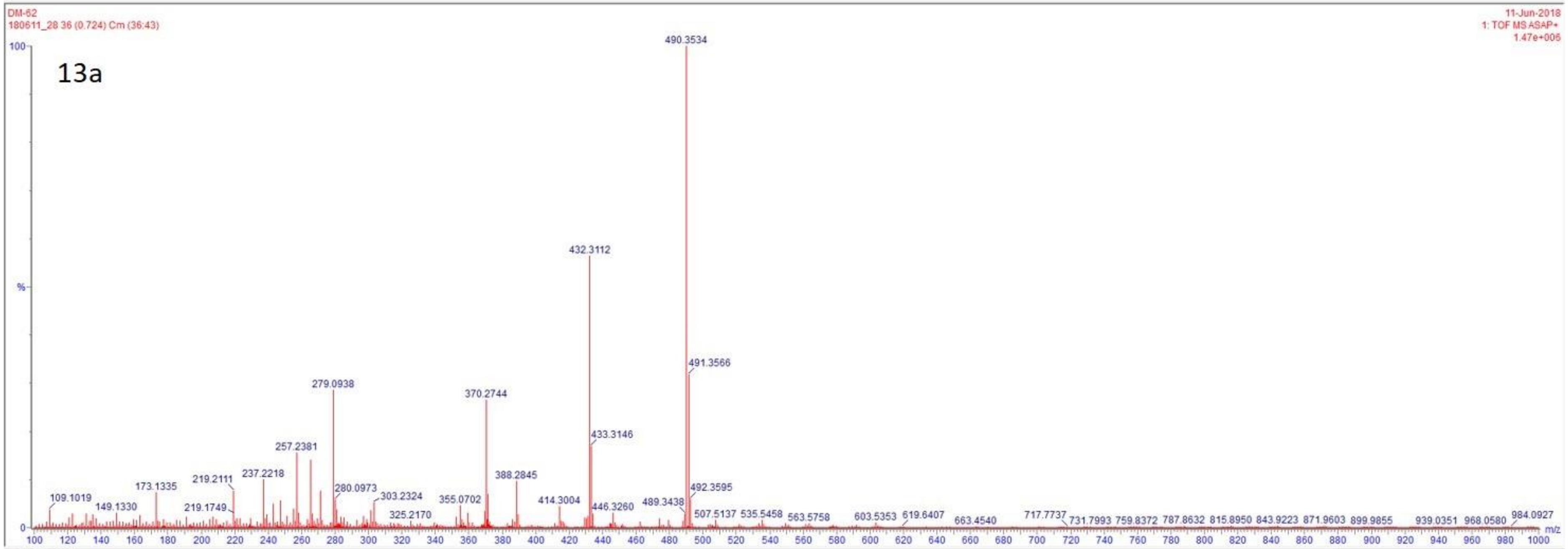

For Help, press F1

13b

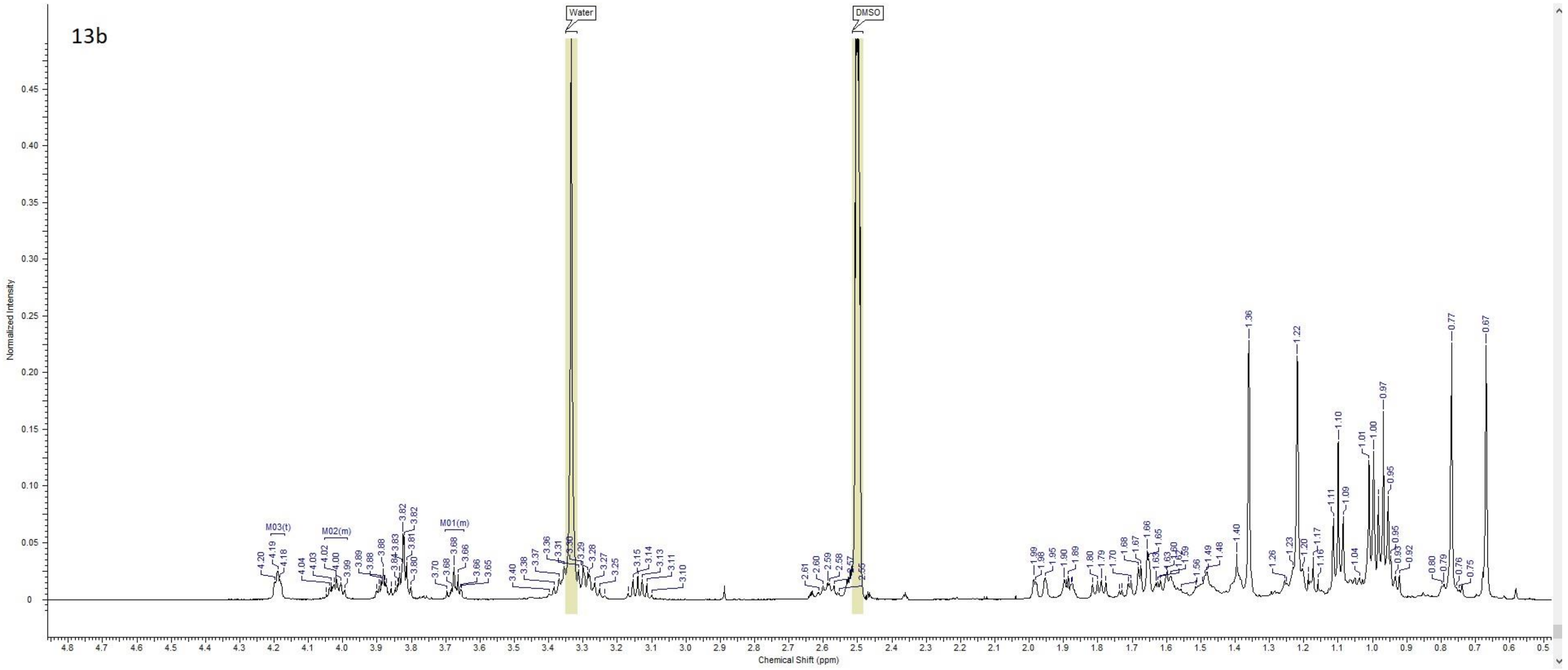

13b

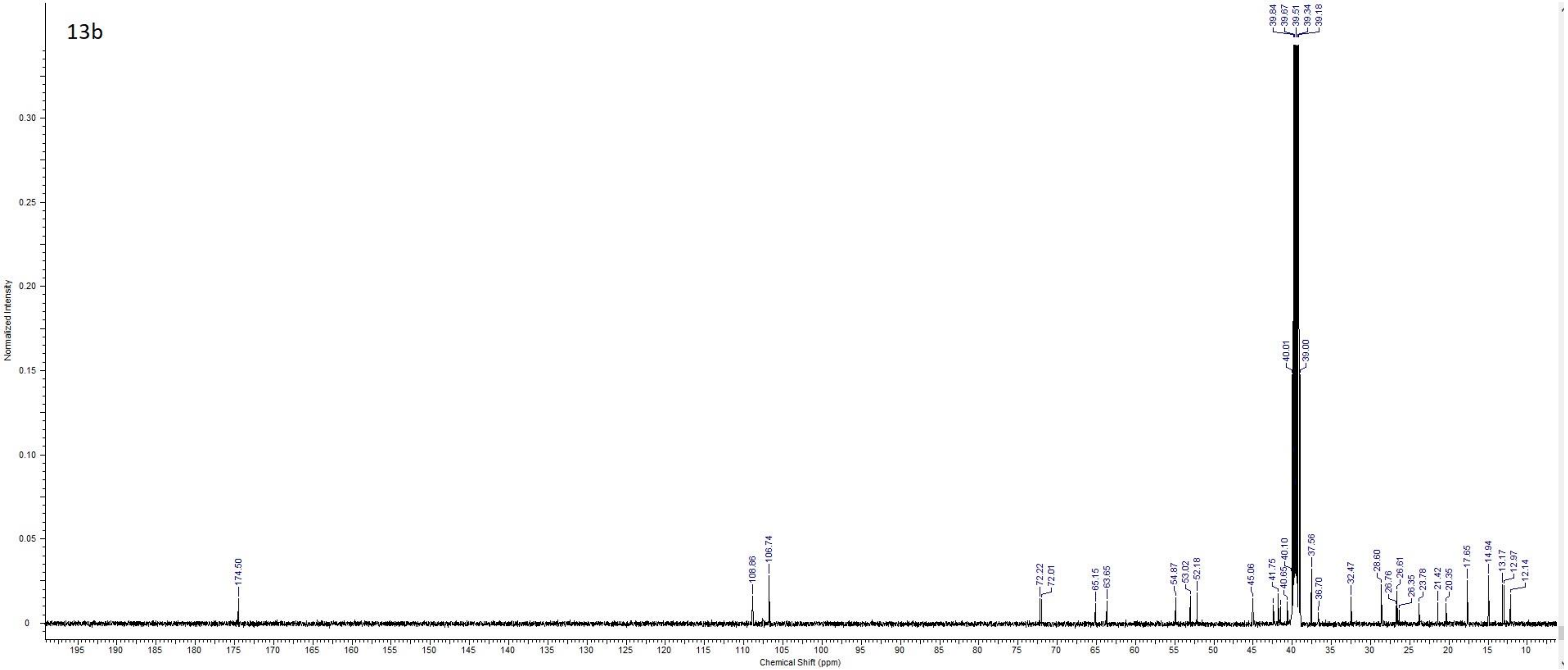

Normalized Intensity
Chemical Shift (ppm)
174.50
108.86
106.74
72.22
72.01
65.15
63.65
54.87
53.02
52.18
45.06
41.75
40.65
40.10
40.01
39.84
39.67
39.51
39.34
39.18
39.00
37.56
36.70
32.47
28.60
26.76
26.61
26.35
23.78
21.42
20.35
17.65
14.94
13.17
12.97
12.14

Elemental Composition

File Edit View Process Help

Single Mass Analysis

Tolerance = 1.0 PPM / DBE: min = -1.5, max = 50.0

Element prediction: Off

Number of isotope peaks used for i-FIT = 3

Monoisotopic Mass, Even Electron Ions

690 formula(e) evaluated with 1 results within limits (up to 50 best isotopic matches for each mass)

Elements Used:

Mass	Calc. Mass	mDa	PPM	DBE	Formula	i-FIT	i-FIT Norm	Fit Conf %	C	H	N	O
518.3849	518.3845	0.4	0.8	6.5	C31 H52 N O5	957.9	n/a	n/a	31	52	1	5

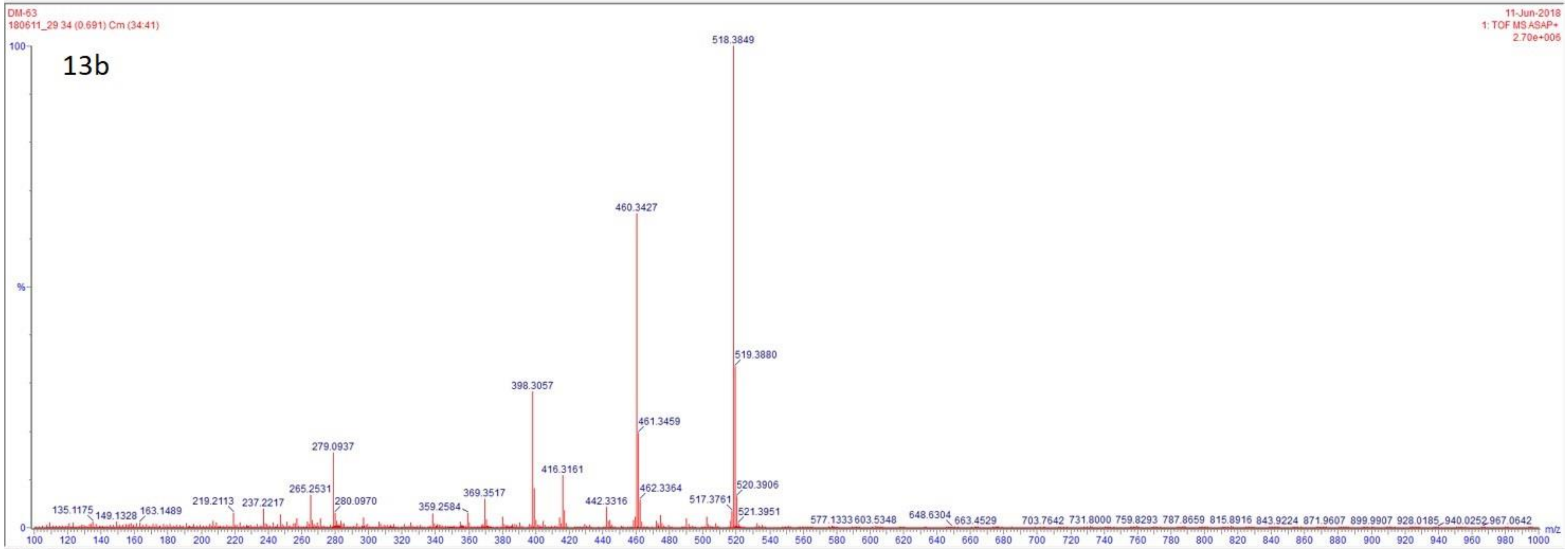

For Help, press F1

13c

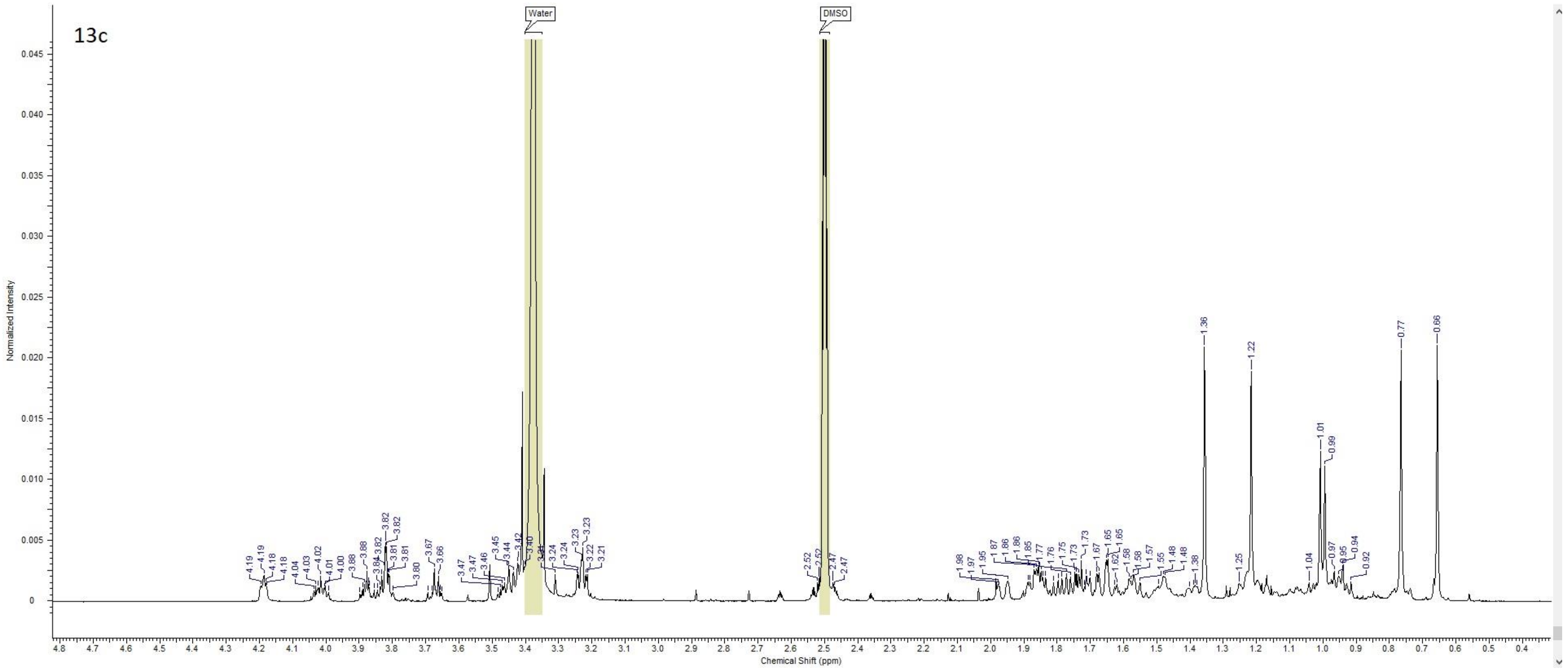
Water
DMSO
Normalized Intensity
Chemical Shift (ppm)
1.36
1.22
1.01
0.99
0.77
0.66

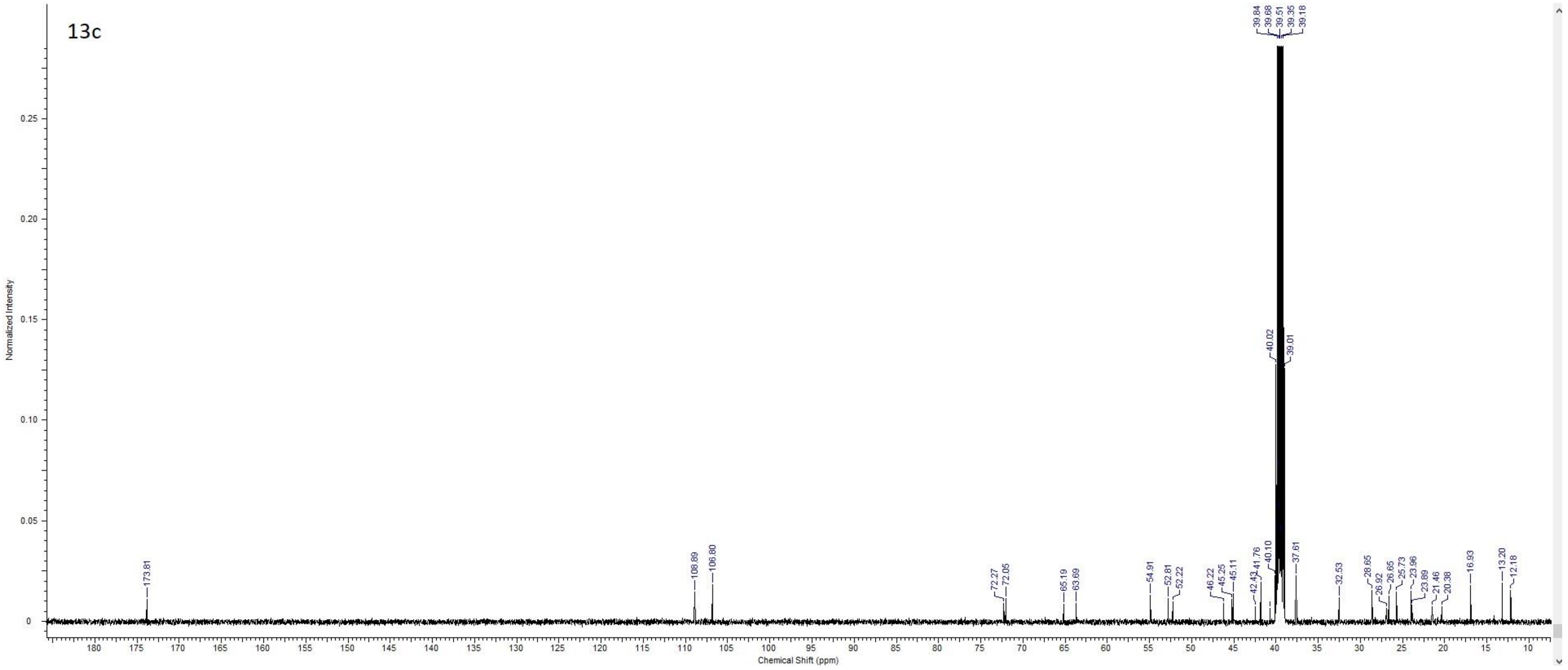

13c
Normalized Intensity
Chemical Shift (ppm)
173.81
108.89
106.80
72.27
72.05
65.19
63.69
54.91
52.81
52.22
46.22
45.25
45.11
42.43
41.76
40.10
40.02
39.84
39.68
39.51
39.35
39.18
39.01
37.61
32.53
28.65
26.92
26.65
25.73
23.96
23.89
21.46
20.38
16.93
13.20
12.18

Elemental Composition

File Edit View Process Help

Single Mass Analysis

Tolerance = 1.0 PPM / DBE: min = -1.5, max = 50.0

Element prediction: Off

Number of isotope peaks used for i-FIT = 3

Monoisotopic Mass, Even Electron Ions

687 formula(e) evaluated with 1 results within limits (up to 50 best isotopic matches for each mass)

Elements Used:

Mass	Calc. Mass	mDa	PPM	DBE	Formula	i-FIT	i-FIT Norm	Fit Conf %	C	H	N	O
516.3690	516.3689	0.1	0.2	7.5	C31 H50 N O5	927.2	n/a	n/a	31	50	1	5

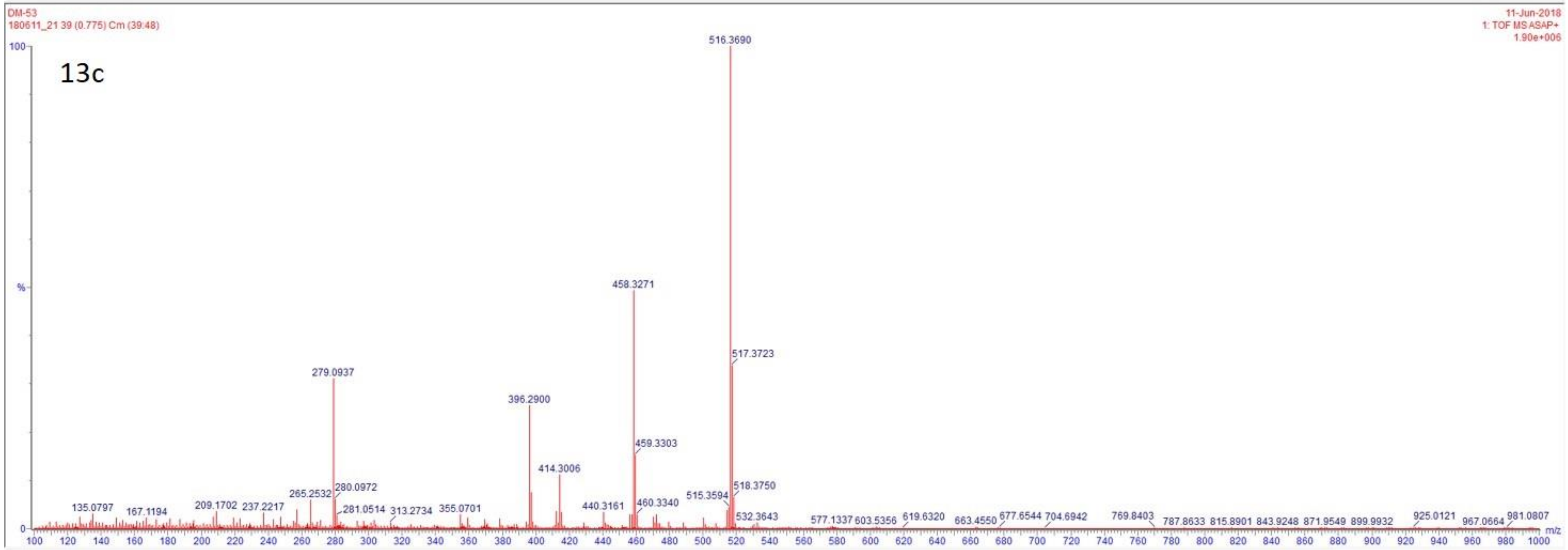

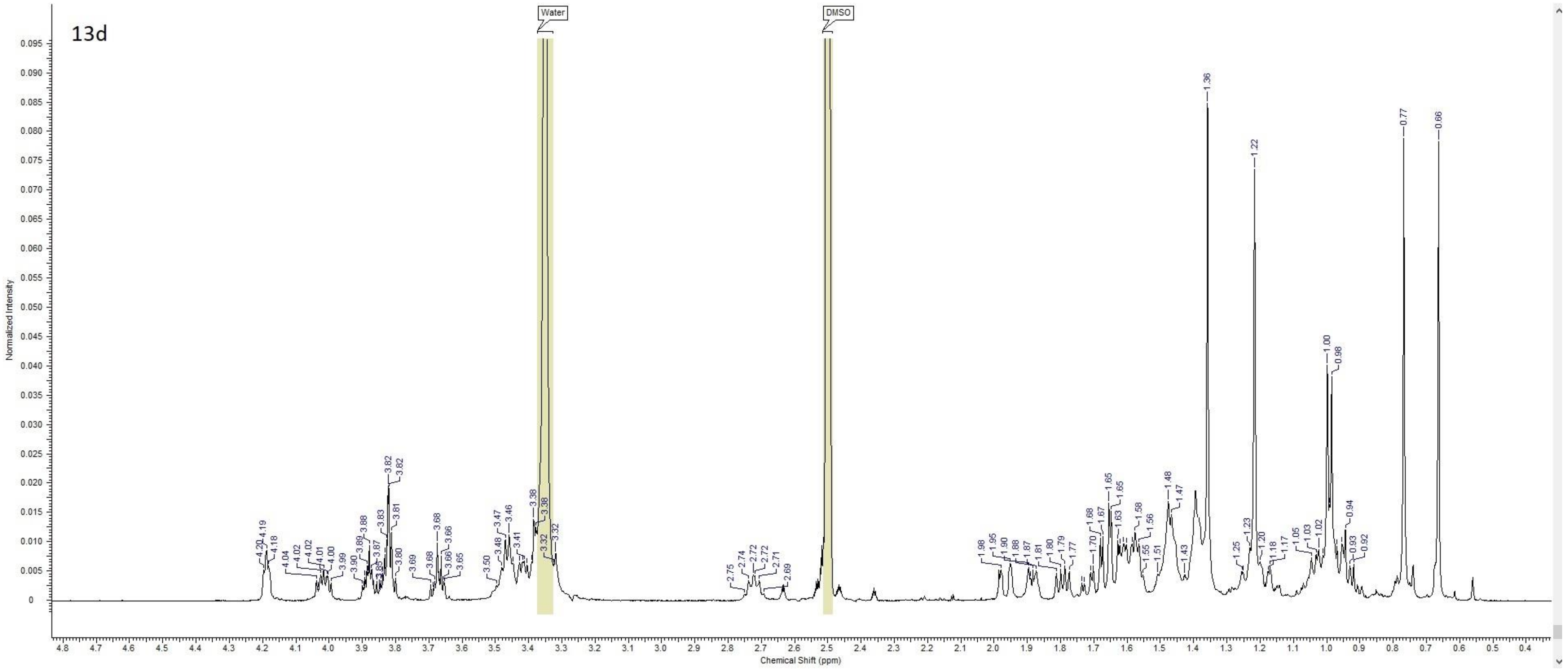
13d
Water
DMSO
Normalized Intensity
Chemical Shift (ppm)

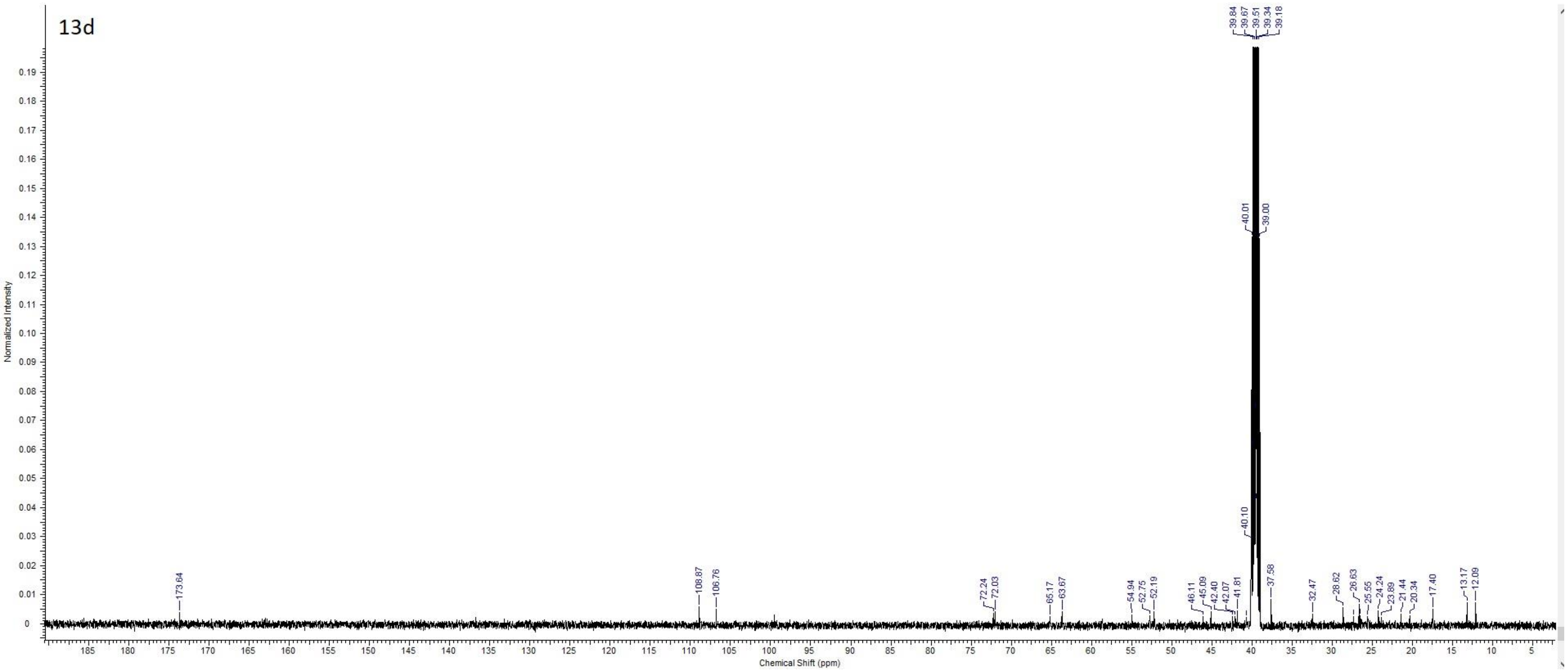

13d
Normalized Intensity
Chemical Shift (ppm)
173.64
108.87
106.76
72.24
72.03
65.17
63.67
54.94
52.75
52.19
46.11
45.09
42.40
42.07
41.81
40.10
40.01
39.84
39.67
39.51
39.34
39.18
39.00
37.58
32.47
28.62
26.63
25.55
24.24
23.89
21.44
20.34
17.40
13.17
12.09

Elemental Composition

File Edit View Process Help

Single Mass Analysis

Tolerance = 1.0 PPM / DBE: min = -1.5, max = 50.0

Element prediction: Off

Number of isotope peaks used for i-FIT = 3

Monoisotopic Mass, Even Electron Ions

705 formula(e) evaluated with 1 results within limits (up to 50 best isotopic matches for each mass)

Elements Used:

Mass	Calc. Mass	mDa	PPM	DBE	Formula	i-FIT	i-FIT Norm	Fit Conf %	C	H	N	O
530.3846	530.3845	0.1	0.2	7.5	C32 H52 N O5	892.5	n/a	n/a	32	52	1	5

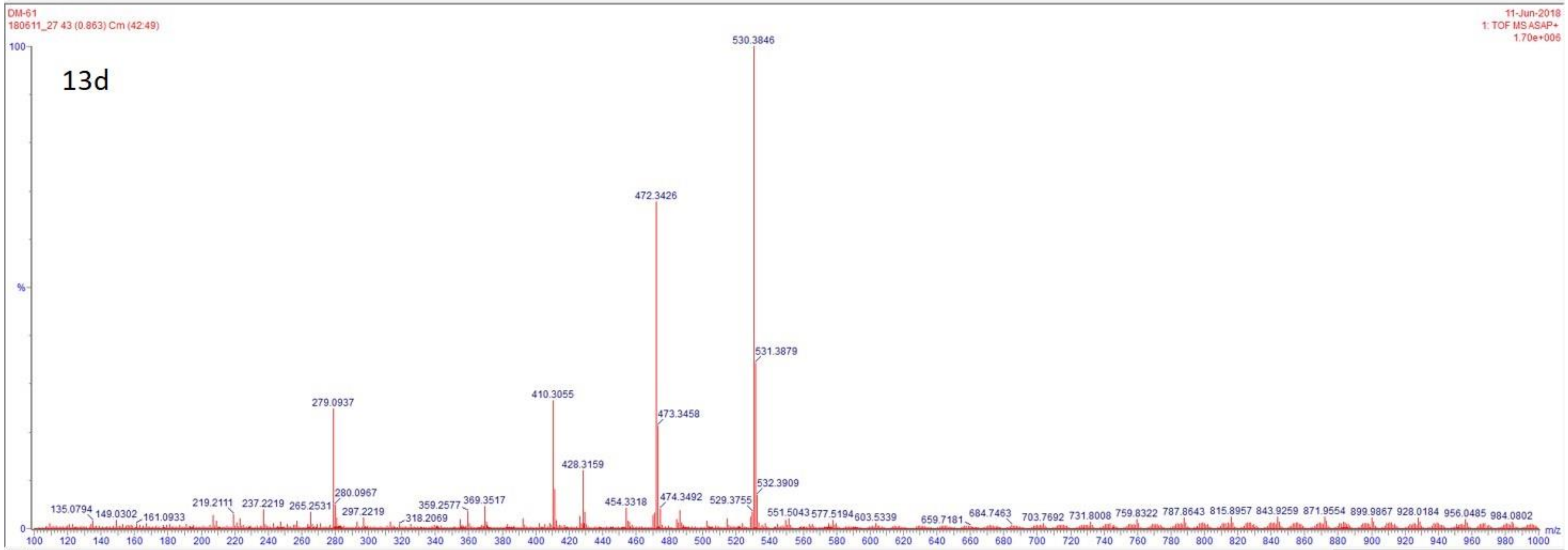

For Help, press F1

13e

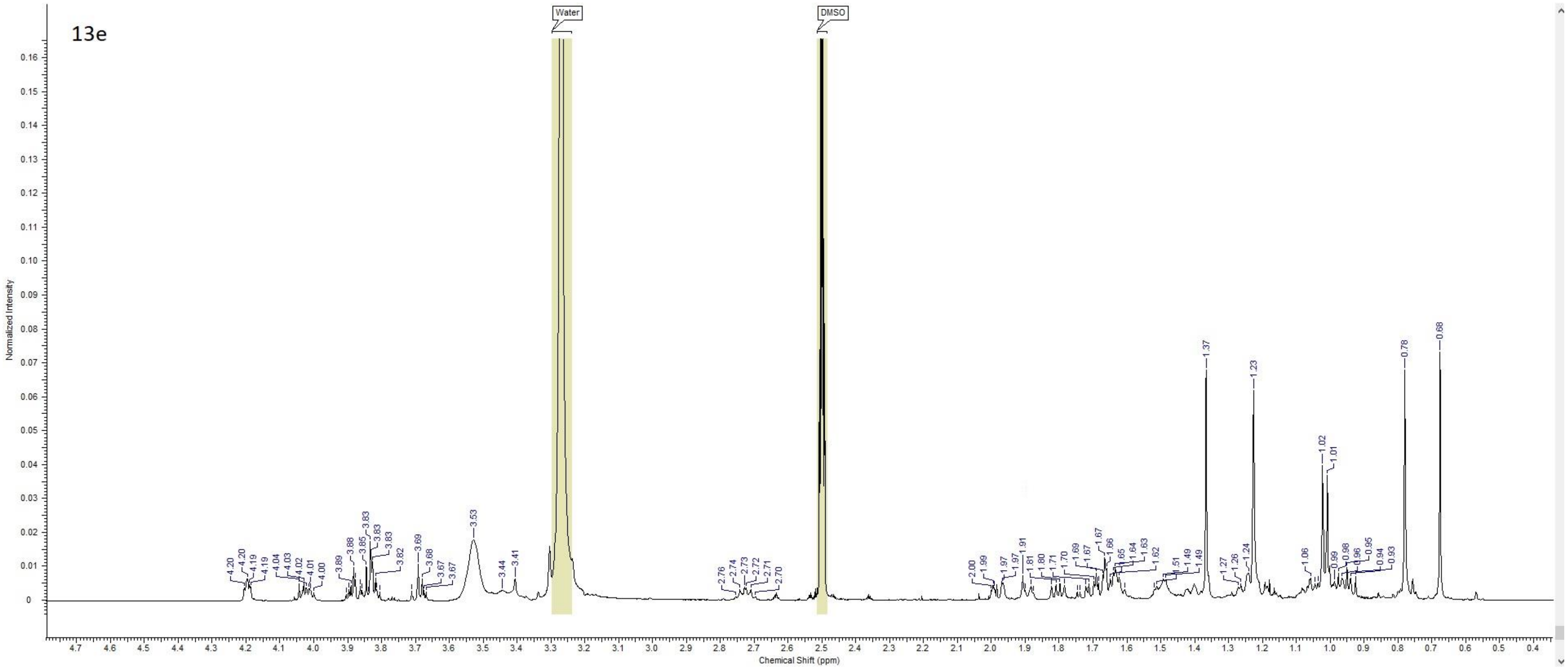

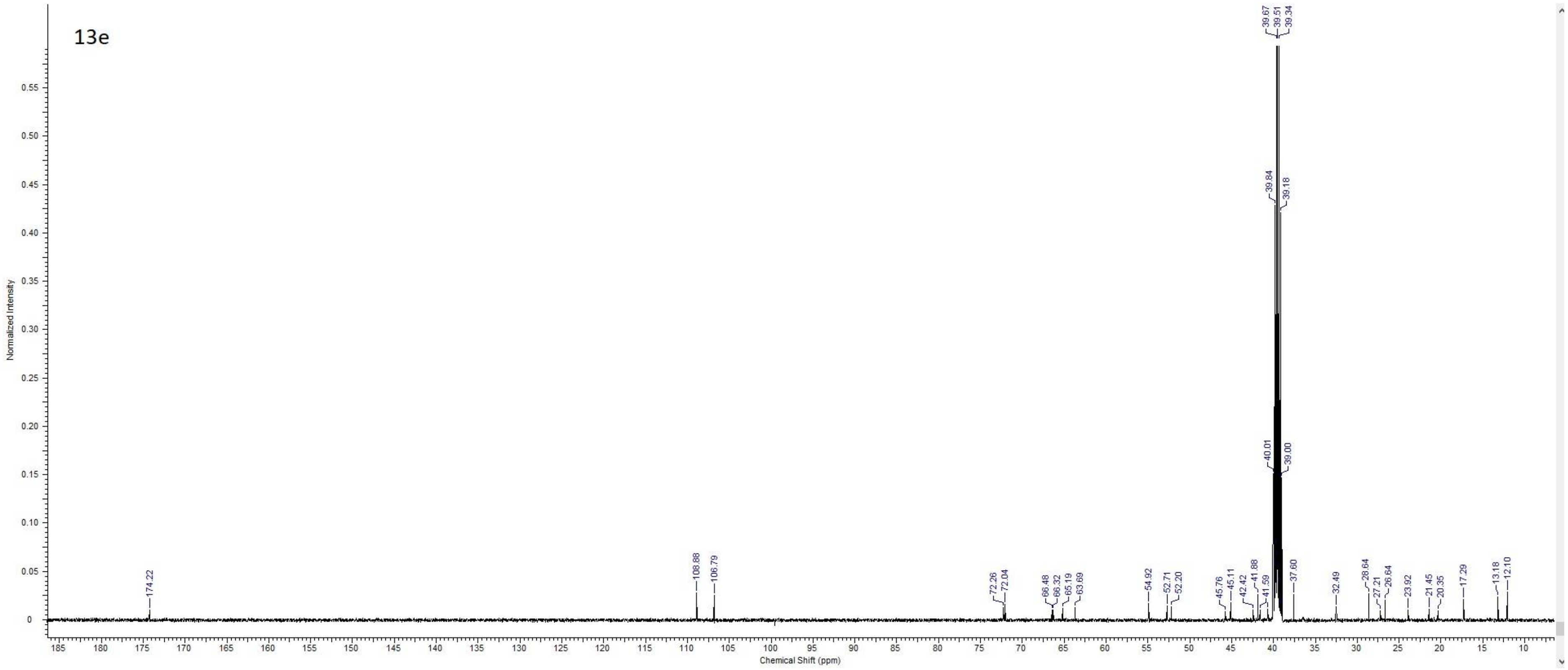
13e
Normalized Intensity
Chemical Shift (ppm)
174.22
108.88
106.79
72.26
72.04
66.48
66.32
65.19
63.69
54.92
52.71
52.20
45.76
45.11
42.42
41.88
41.59
40.01
39.84
39.67
39.51
39.34
39.18
39.00
37.60
32.49
28.64
27.21
26.64
23.92
21.45
20.35
17.29
13.18
12.10

Elemental Composition

File Edit View Process Help

Single Mass Analysis

Tolerance = 1.0 PPM / DBE: min = -1.5, max = 50.0

Element prediction: Off

Number of isotope peaks used for i-FIT = 3

Monoisotopic Mass, Even Electron Ions

712 formula(e) evaluated with 1 results within limits (up to 50 best isotopic matches for each mass)

Elements Used:

Mass	Calc. Mass	mDa	PPM	DBE	Formula	i-FIT	i-FIT Norm	Fit Conf %	C	H	N	O
532.3639	532.3638	0.1	0.2	7.5	C31 H50 N O6	908.5	n/a	n/a	31	50	1	6

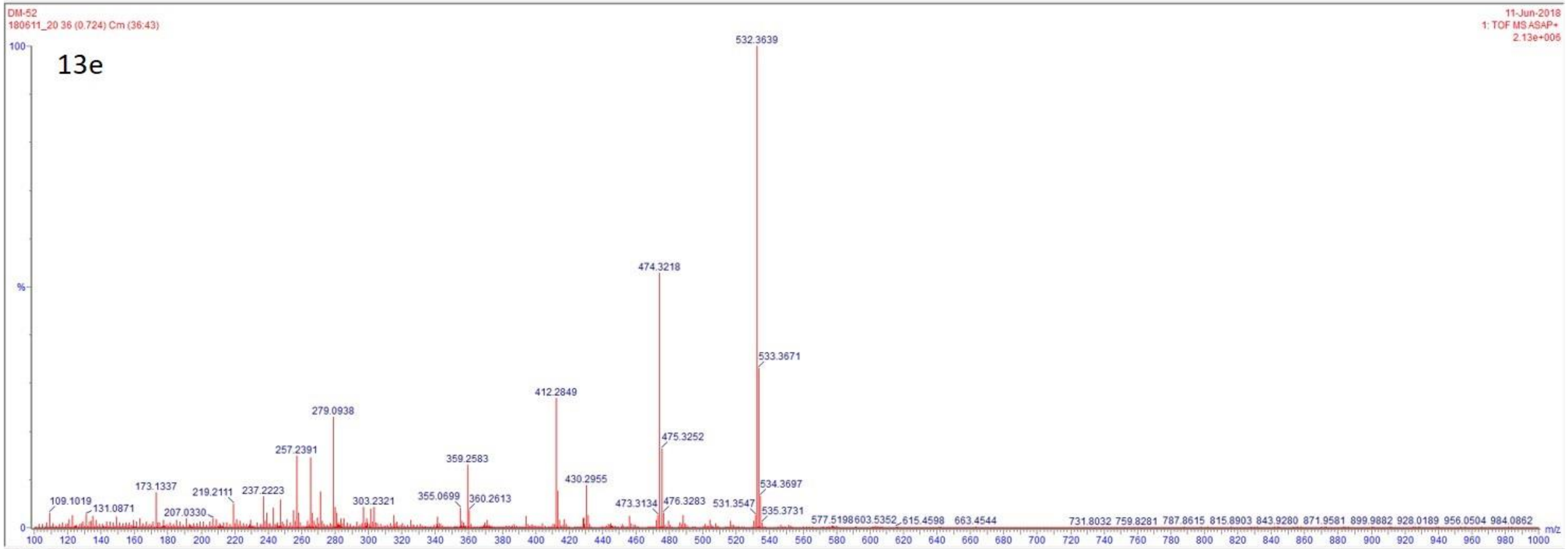

For Help, press F1

14a

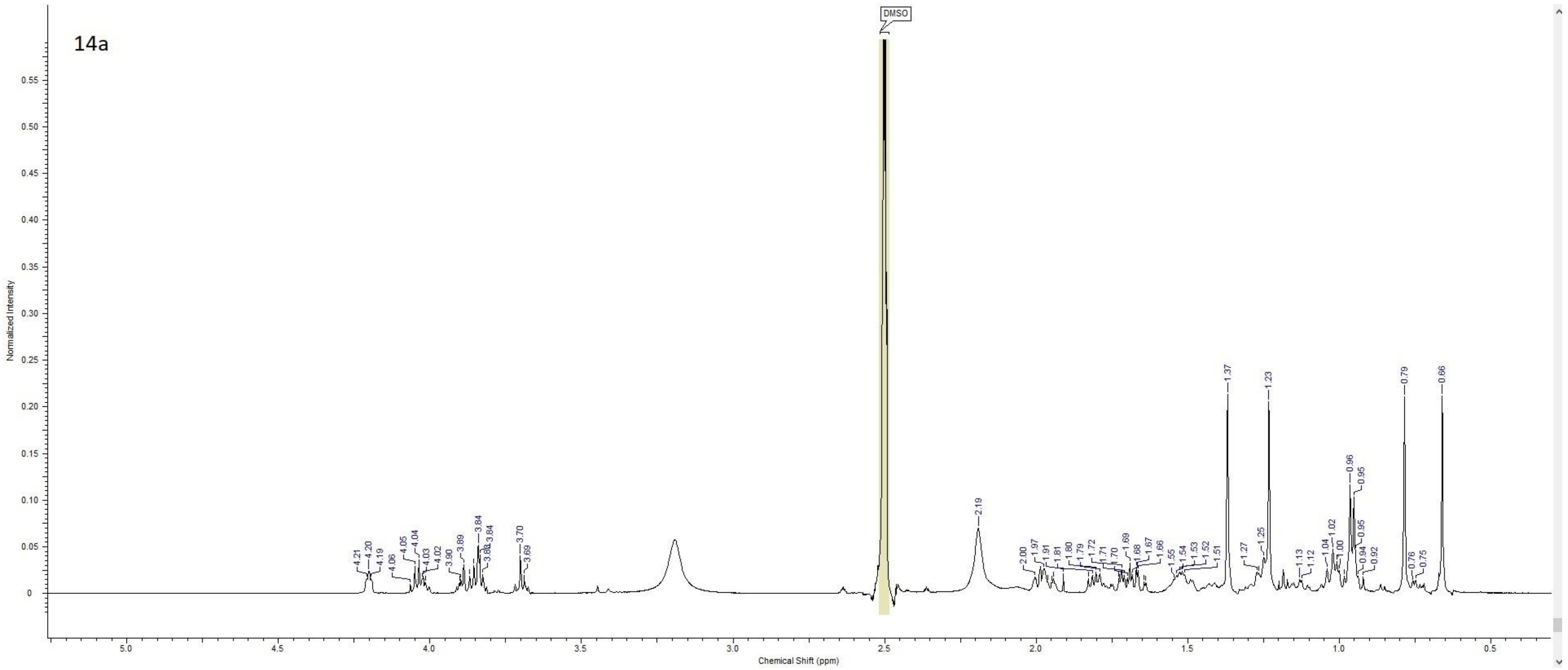

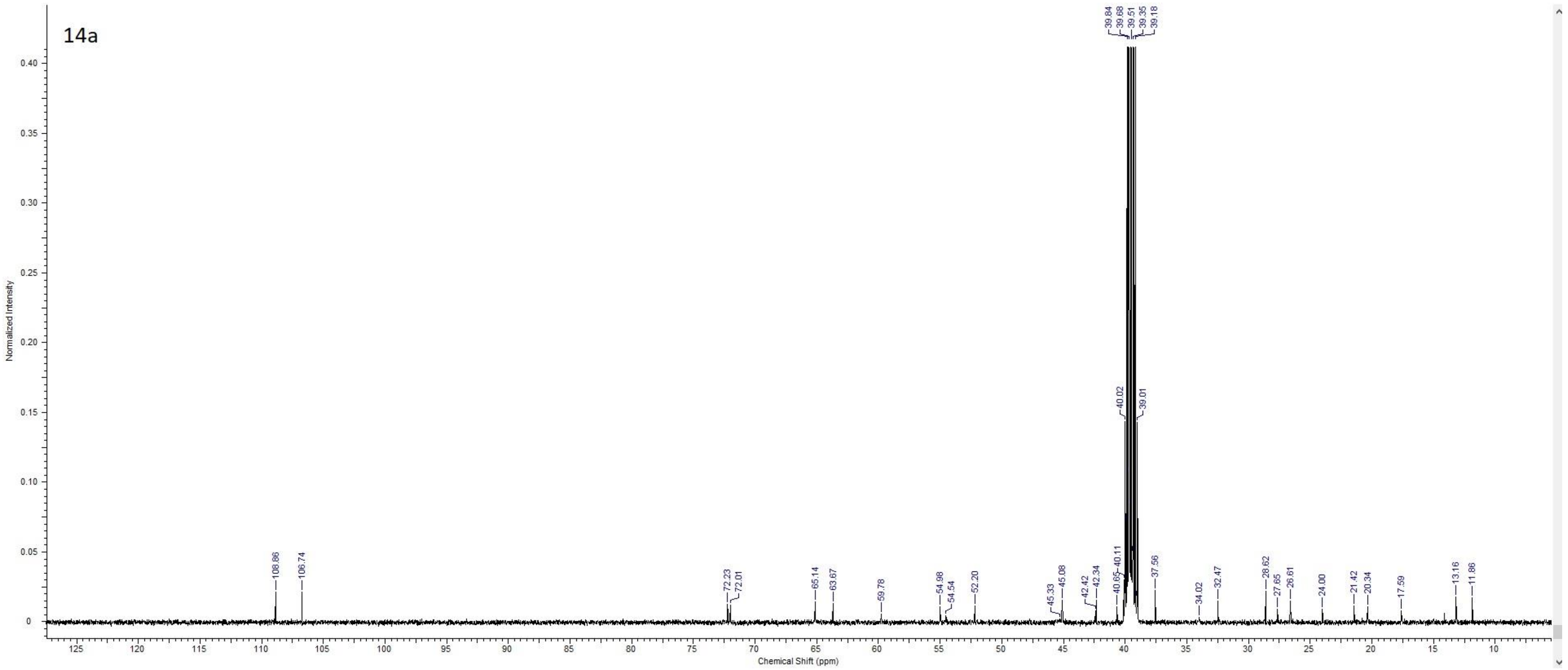
14a
Normalized Intensity
Chemical Shift (ppm)
108.86
106.74
72.23
72.01
65.14
63.67
59.78
54.98
54.54
52.20
45.33
45.08
42.42
42.34
40.65
40.11
40.02
39.84
39.68
39.51
39.35
39.18
39.01
37.56
34.02
32.47
28.62
27.65
26.61
24.00
21.42
20.34
17.59
13.16
11.86

Elemental Composition

File Edit View Process Help

Single Mass Analysis

Tolerance = 1.0 PPM / DBE: min = -1.5, max = 50.0

Element prediction: Off

Number of isotope peaks used for i-FIT = 3

Monoisotopic Mass, Even Electron Ions

632 formula(e) evaluated with 1 results within limits (up to 50 best isotopic matches for each mass)

Elements Used:

Mass	Calc. Mass	mDa	PPM	DBE	Formula	i-FIT	i-FIT Norm	Fit Conf %	C	H	N	O
476.3742	476.3740	0.2	0.4	5.5	C29 H50 N O4	834.1	n/a	n/a	29	50	1	4

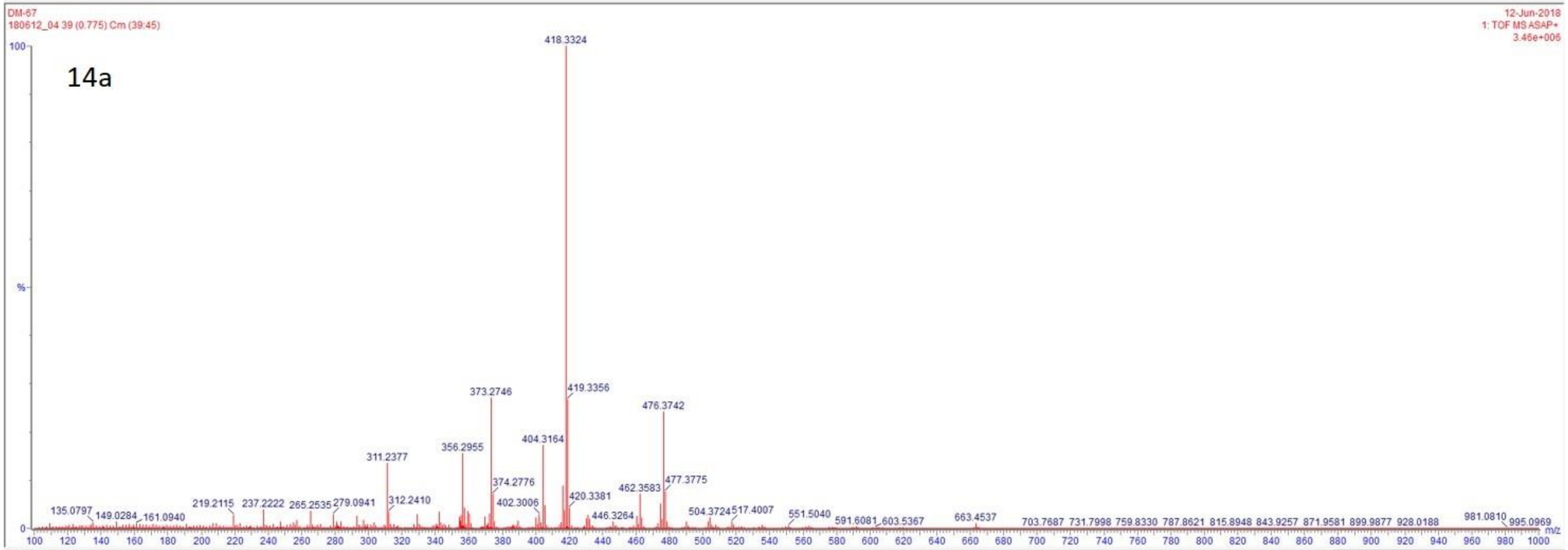

For Help, press F1

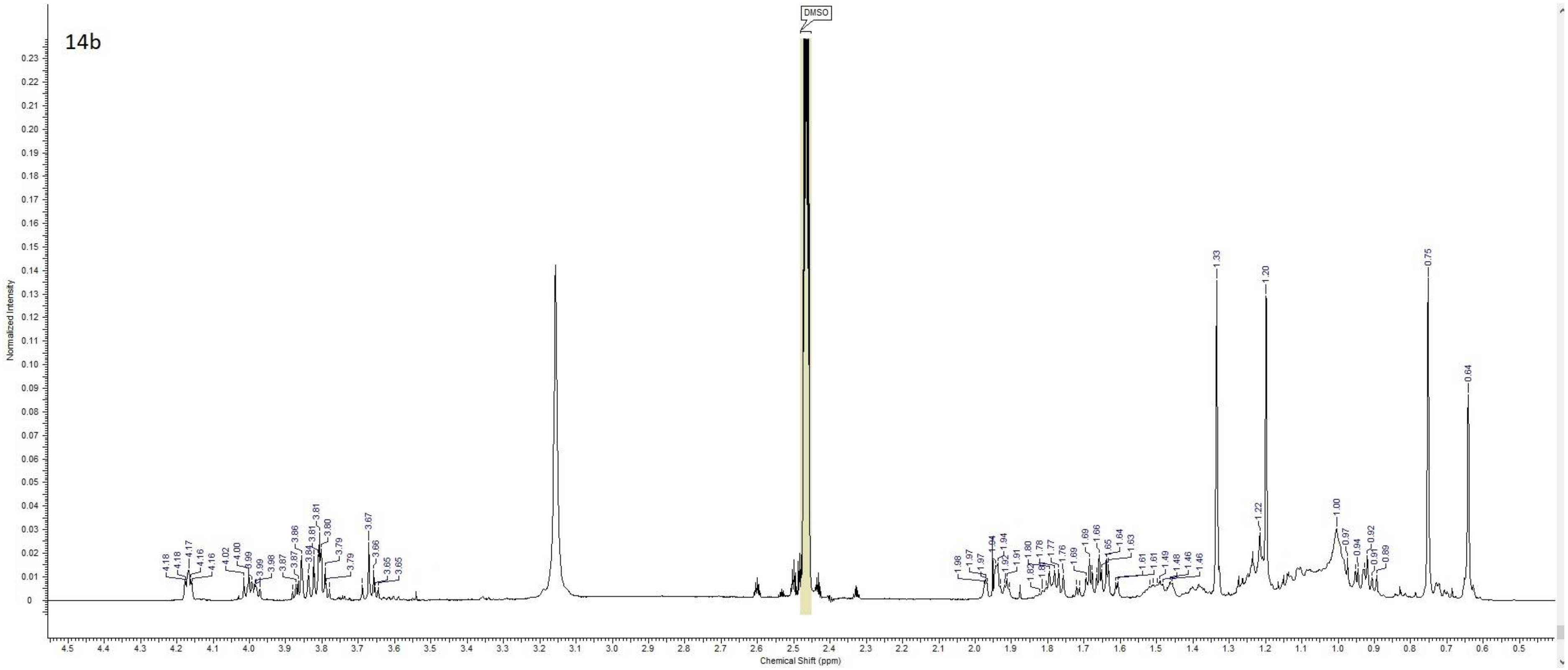

14b
DMSO
Normalized Intensity
Chemical Shift (ppm)

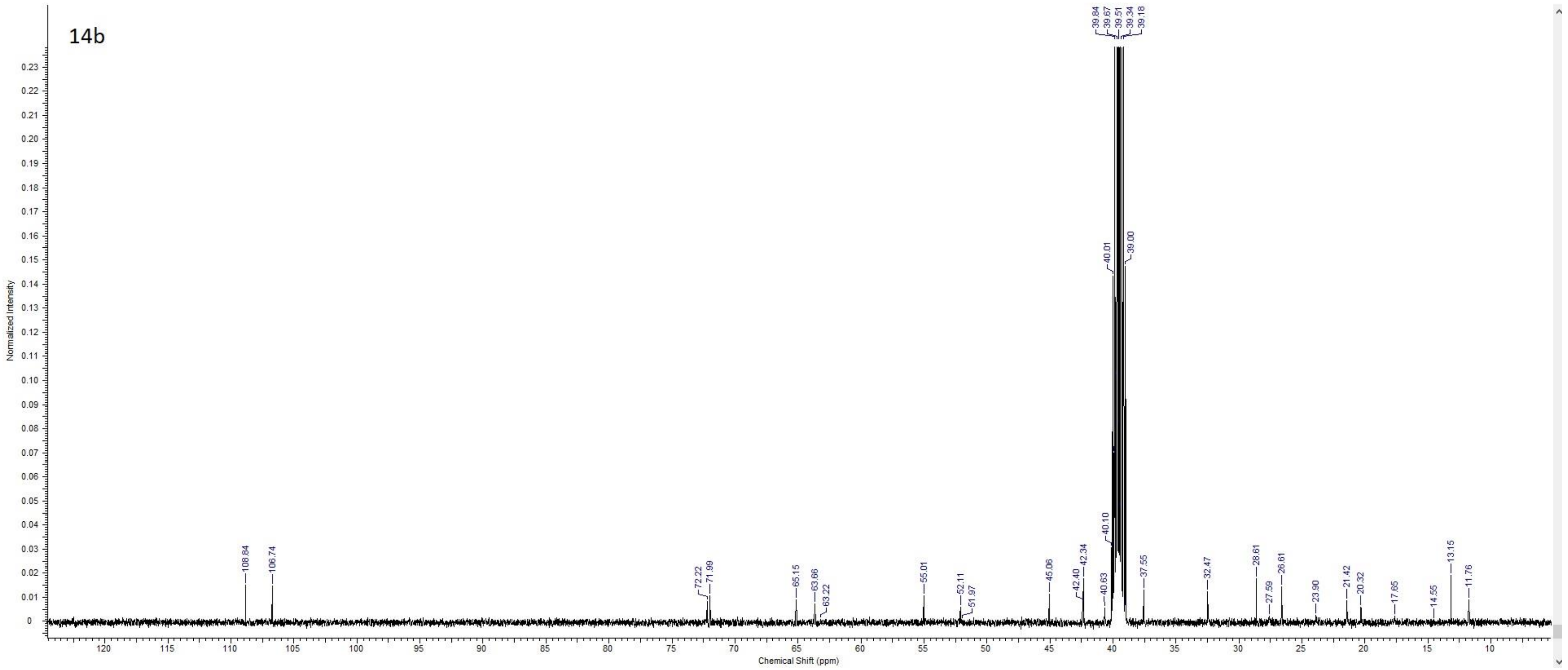
14b
Normalized Intensity
Chemical Shift (ppm)
108.84
106.74
72.22
71.99
65.15
63.66
63.22
55.01
52.11
51.97
45.06
42.40
42.34
40.63
40.10
40.01
39.84
39.67
39.51
39.34
39.18
39.00
37.55
32.47
28.61
27.59
26.61
23.90
21.42
20.32
17.65
14.55
13.15
11.76

Elemental Composition

File Edit View Process Help

Single Mass Analysis

Tolerance = 1.0 PPM / DBE: min = -1.5, max = 50.0

Element prediction: Off

Number of isotope peaks used for i-FIT = 3

Monoisotopic Mass, Even Electron Ions

672 formula(e) evaluated with 1 results within limits (up to 50 best isotopic matches for each mass)

Elements Used:

Mass	Calc. Mass	mDa	PPM	DBE	Formula	i-FIT	i-FIT Norm	Fit Conf %	C	H	N	O
504.4050	504.4053	-0.3	-0.6	5.5	C31 H54 N O4	742.8	n/a	n/a	31	54	1	4

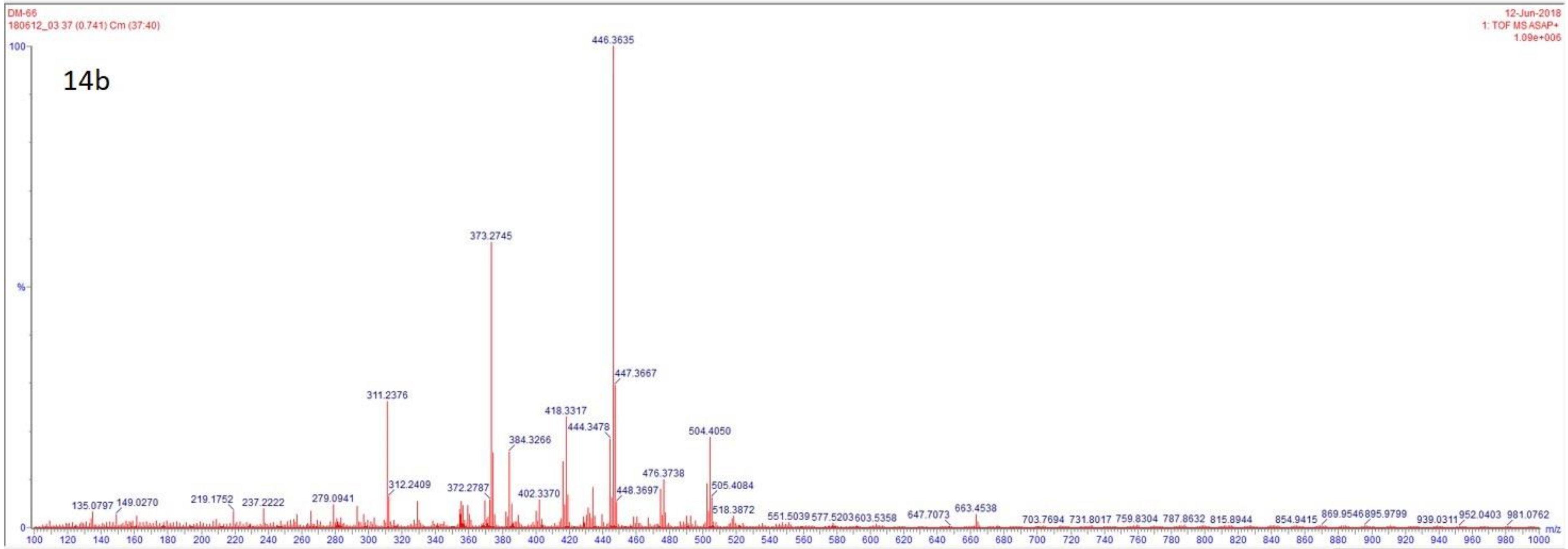

For Help, press F1

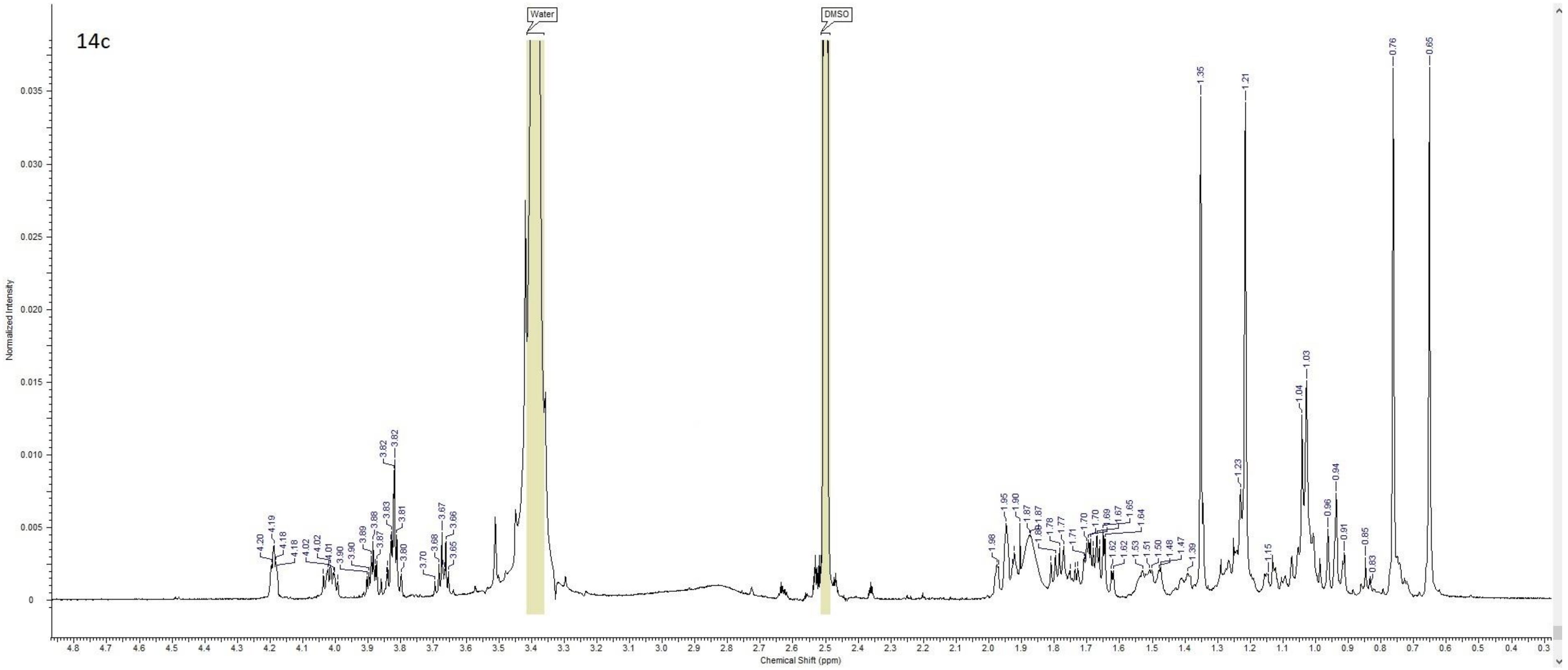
14c
Water
DMSO
Normalized Intensity
Chemical Shift (ppm)

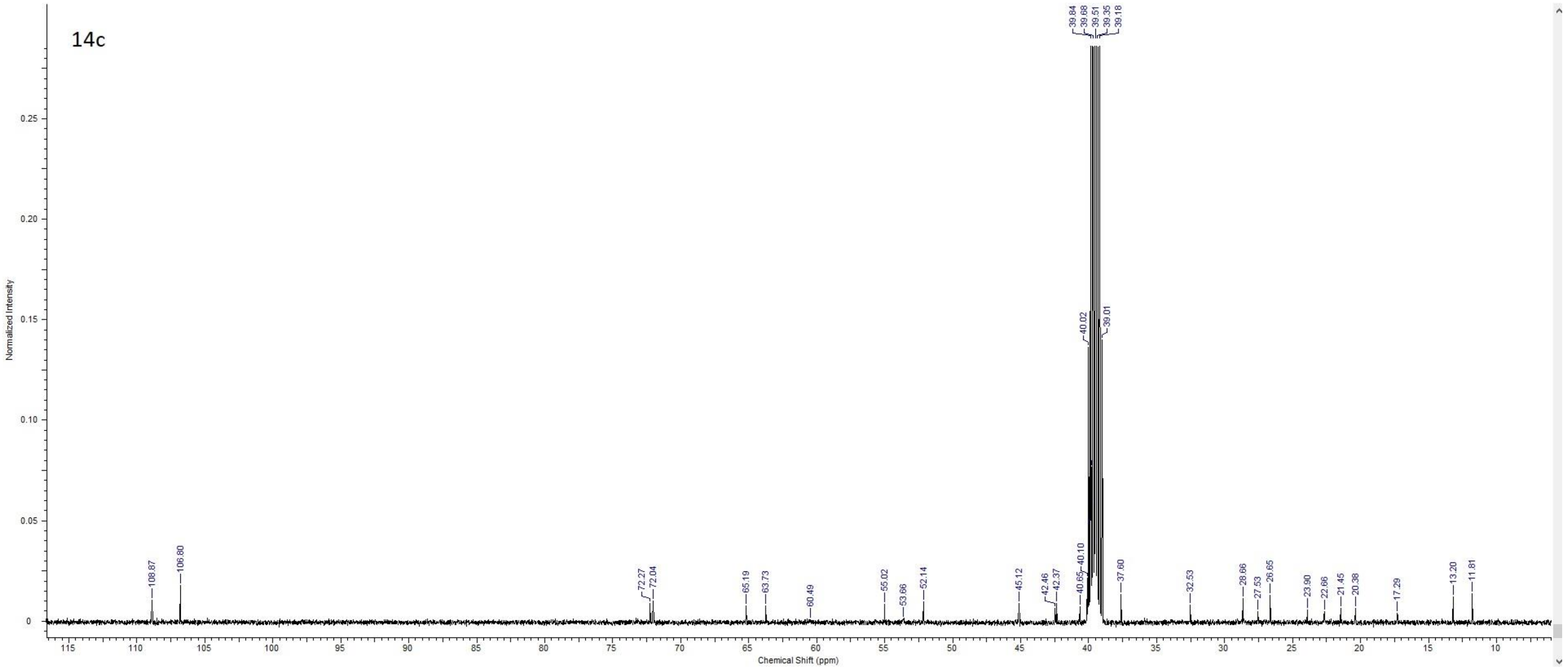
14c
Normalized Intensity
Chemical Shift (ppm)
108.87
106.80
72.27
72.04
65.19
63.73
60.49
55.02
53.66
52.14
45.12
42.46
42.37
40.65
40.10
40.02
39.84
39.68
39.51
39.35
39.18
39.01
37.60
32.53
28.66
27.53
26.65
23.90
22.66
21.45
20.38
17.29
13.20
11.81

Elemental Composition

File Edit View Process Help

Single Mass Analysis
Tolerance = 1.0 PPM / DBE: min = -1.5, max = 50.0
Element prediction: Off
Number of isotope peaks used for i-FIT = 3
Monoisotopic Mass, Even Electron Ions
665 formula(e) evaluated with 1 results within limits (up to 50 best isotopic matches for each mass)
Elements Used:

Mass	Calc. Mass	mDa	PPM	DBE	Formula	i-FIT	i-FIT Norm	Fit Conf %	C	H	N	O
502.3895	502.3896	-0.1	-0.2	6.5	C31 H52 N O4	700.6	n/a	n/a	31	52	1	4

DM-58
180611_23 34 (0.691) Cm (34:35)

11-Jun-2018
1: TOF MS ASAP+
6.94e+005

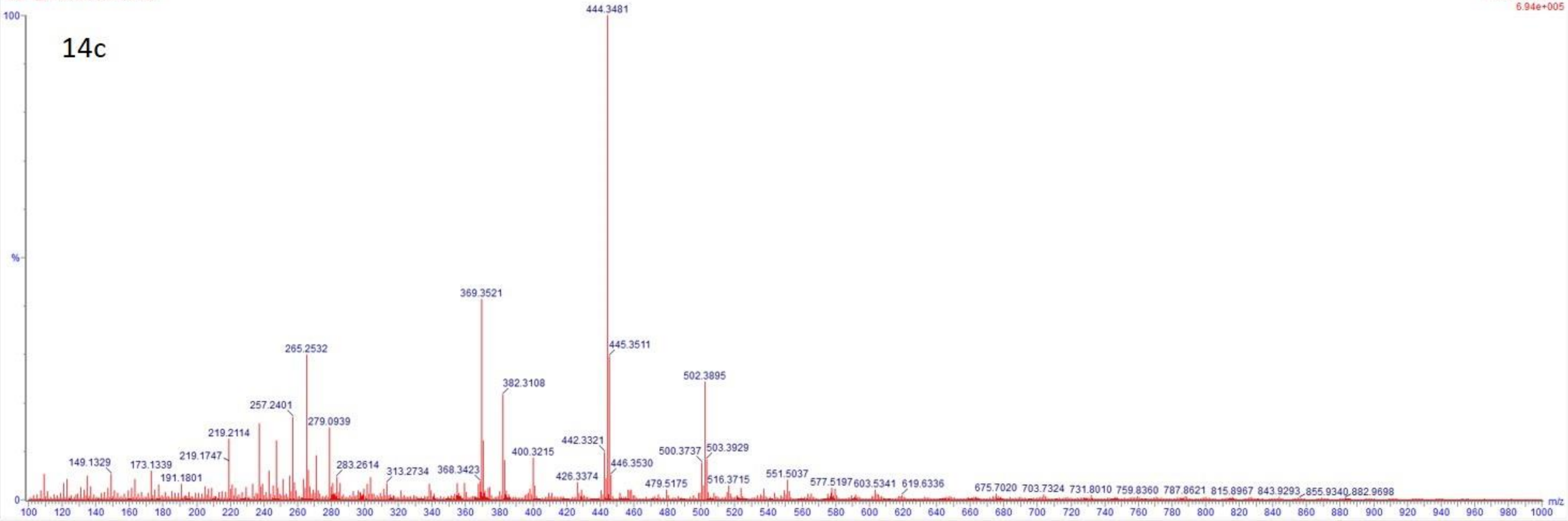

For Help, press F1

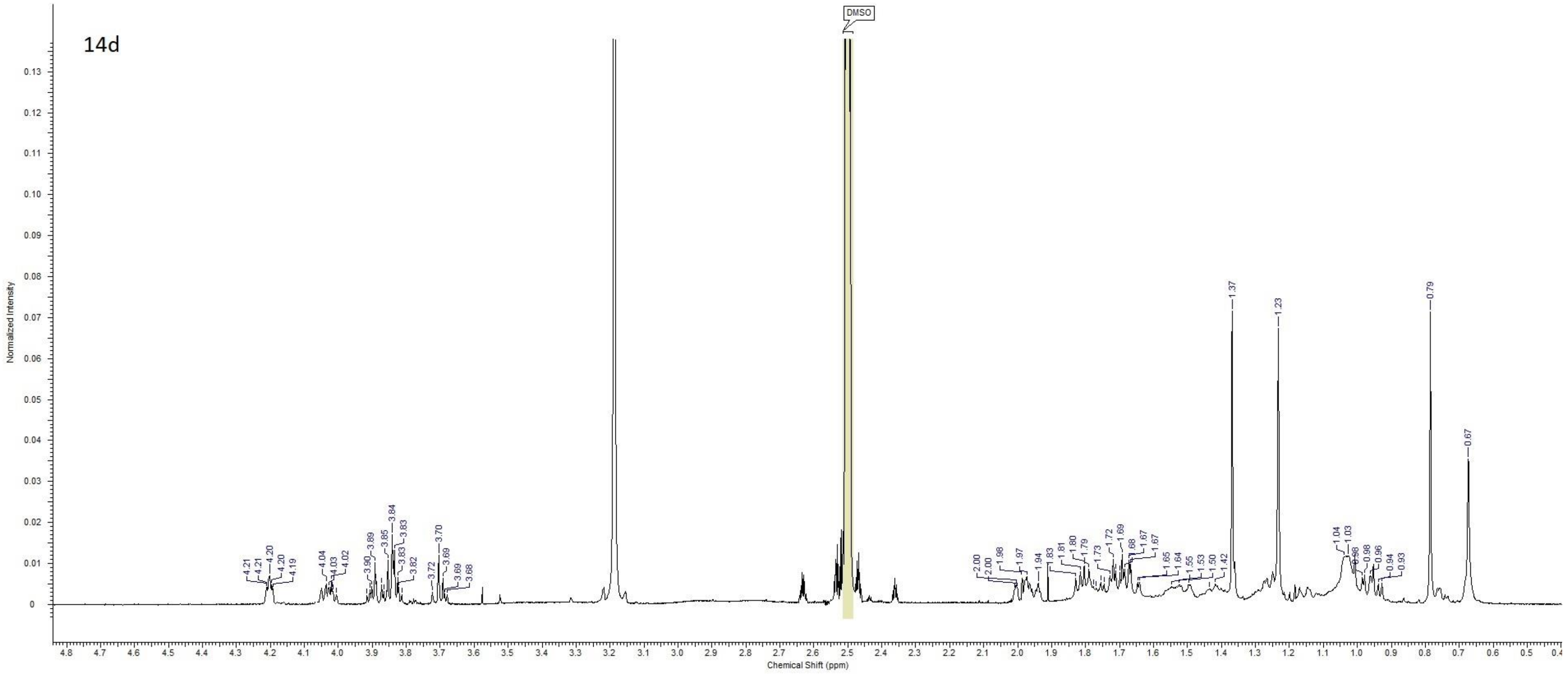

14d
DMSO
Normalized Intensity
Chemical Shift (ppm)
4.21
4.21
4.20
4.20
4.19
4.04
4.03
4.02
3.90
3.89
3.85
3.84
3.83
3.83
3.82
3.72
3.70
3.69
3.69
3.68
2.00
2.00
1.98
1.97
1.94
1.83
1.81
1.80
1.79
1.73
1.72
1.69
1.68
1.67
1.67
1.65
1.64
1.55
1.53
1.50
1.42
1.37
1.23
1.04
1.03
0.98
0.98
0.96
0.94
0.93
0.79
0.67

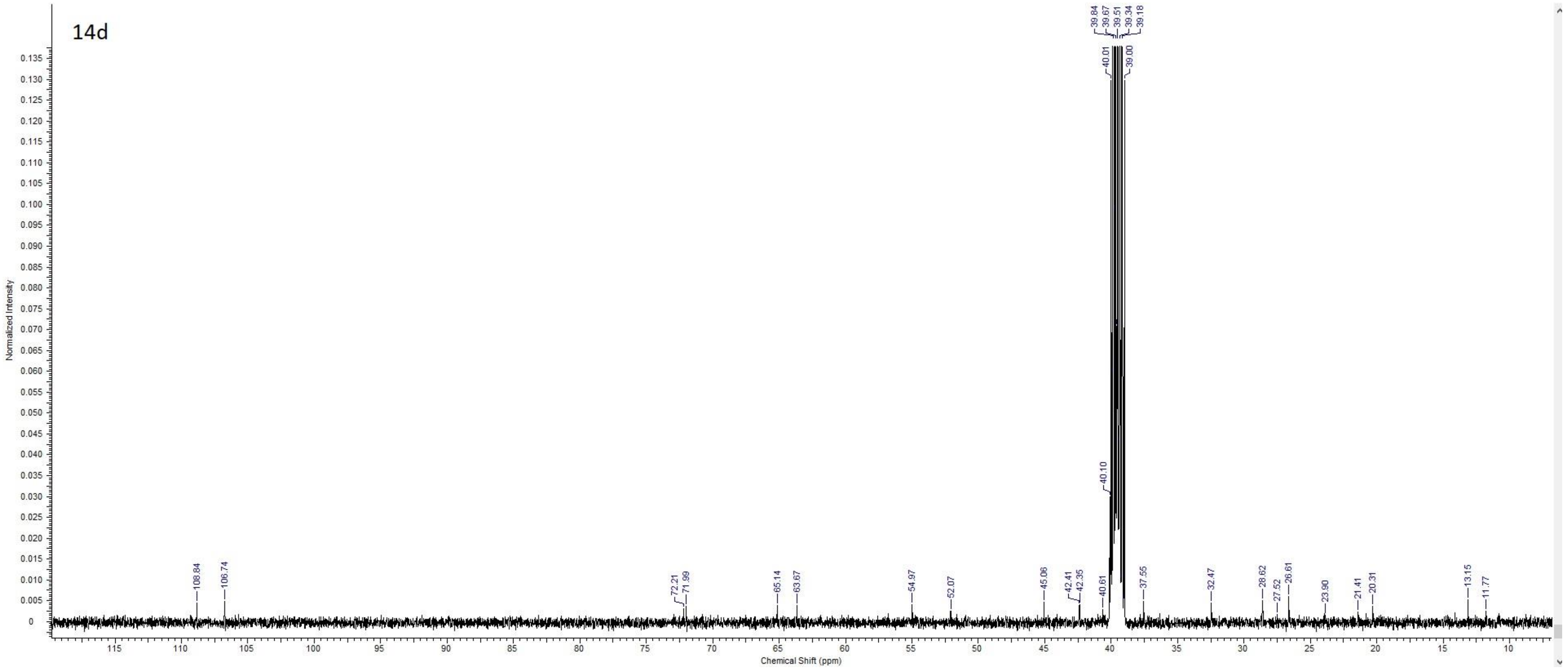

14d
Normalized Intensity
Chemical Shift (ppm)
108.84
106.74
72.21
71.99
65.14
63.67
54.97
52.07
45.06
42.41
42.35
40.61
40.10
40.01
39.84
39.67
39.51
39.34
39.18
39.00
37.55
32.47
28.62
27.52
26.61
23.90
21.41
20.31
13.15
11.77

Elemental Composition

File Edit View Process Help

Single Mass Analysis

Tolerance = 1.0 PPM / DBE: min = -1.5, max = 50.0

Element prediction: Off

Number of isotope peaks used for i-FIT = 3

Monoisotopic Mass, Even Electron Ions

687 formula(e) evaluated with 1 results within limits (up to 50 best isotopic matches for each mass)

Elements Used:

Mass	Calc. Mass	mDa	PPM	DBE	Formula	i-FIT	i-FIT Norm	Fit Conf %	C	H	N	O
516.4055	516.4053	0.2	0.4	6.5	C32 H54 N O4	738.7	n/a	n/a	32	54	1	4

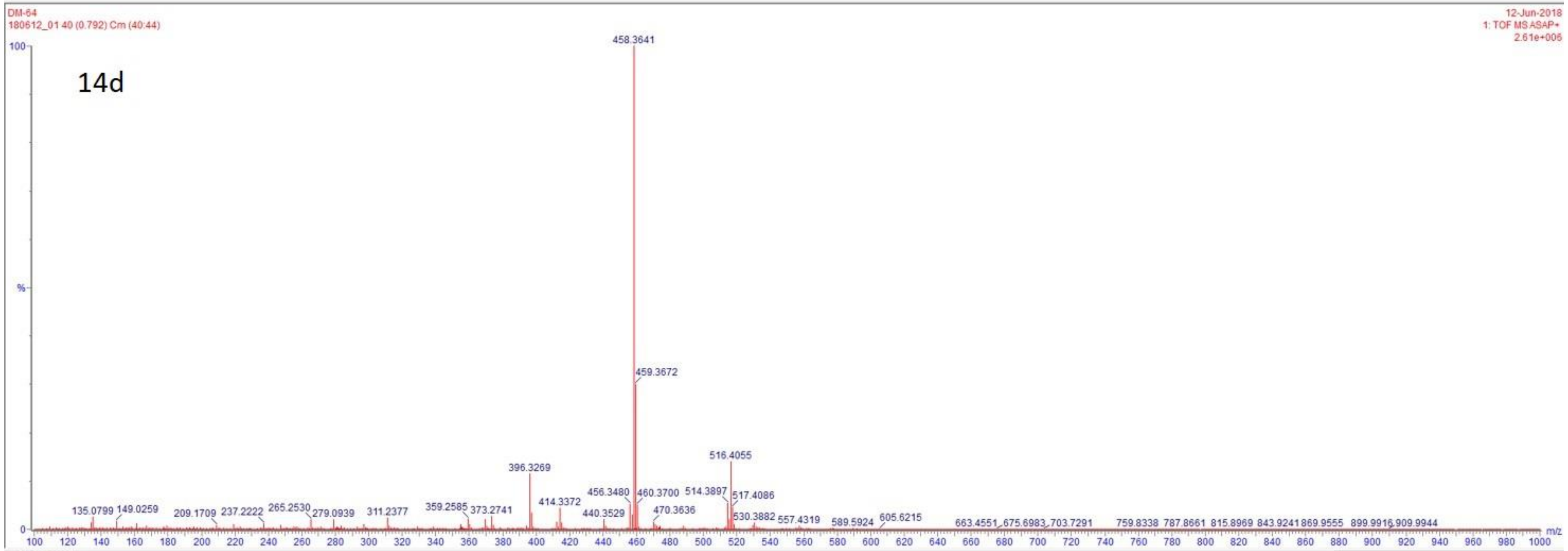

For Help, press F1

14e

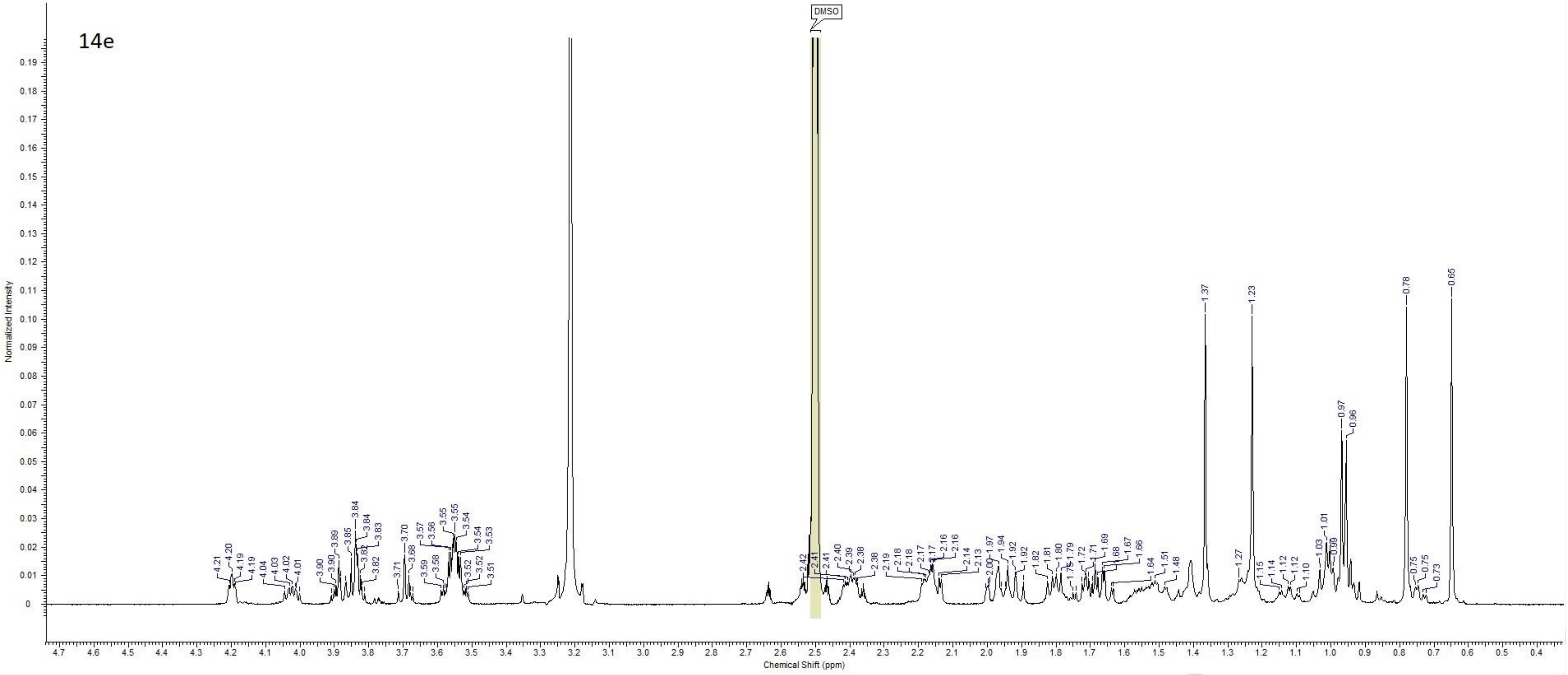

14e

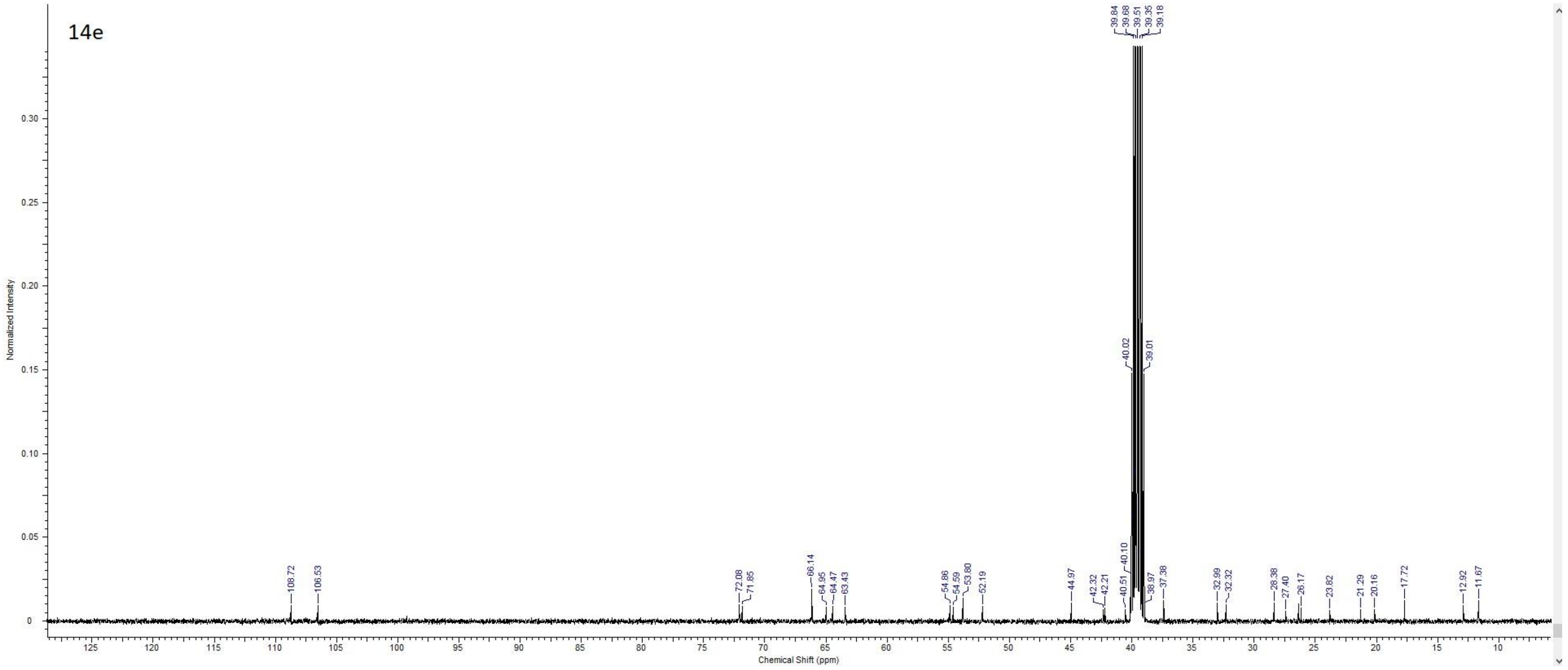
39.84
39.68
39.51
39.35
39.18
40.02
39.01
108.72
106.53
72.08
71.85
66.14
64.95
64.47
63.43
54.86
54.59
53.80
52.19
44.97
42.32
42.21
40.51
40.10
38.97
37.38
32.99
32.32
28.38
27.40
26.17
23.82
21.29
20.16
17.72
12.92
11.67
Normalized Intensity
Chemical Shift (ppm)

Elemental Composition

File Edit View Process Help

Single Mass Analysis

Tolerance = 1.0 PPM / DBE: min = -1.5, max = 50.0

Element prediction: Off

Number of isotope peaks used for i-FIT = 3

Monoisotopic Mass, Even Electron Ions

690 formula(e) evaluated with 1 results within limits (up to 50 best isotopic matches for each mass)

Elements Used:

Mass	Calc. Mass	mDa	PPM	DBE	Formula	i-FIT	i-FIT Norm	Fit Conf %	C	H	N	O
518.3849	518.3845	0.4	0.8	6.5	C31 H52 N O5	954.6	n/a	n/a	31	52	1	5

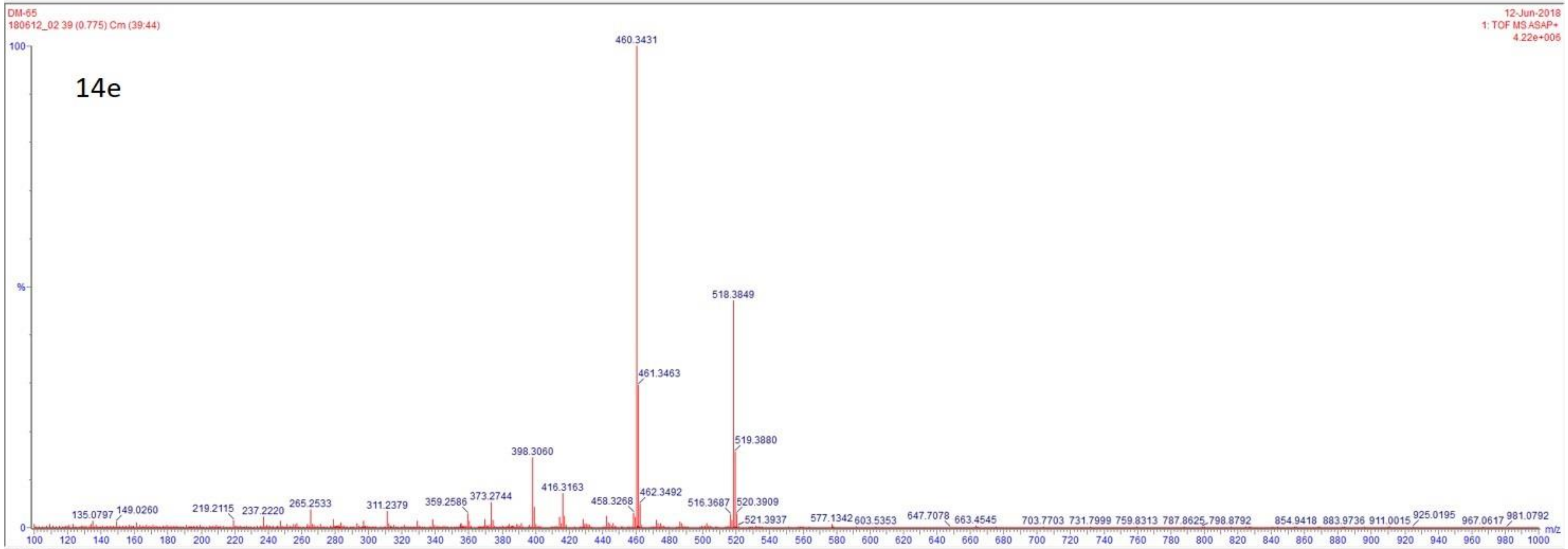

For Help, press F1

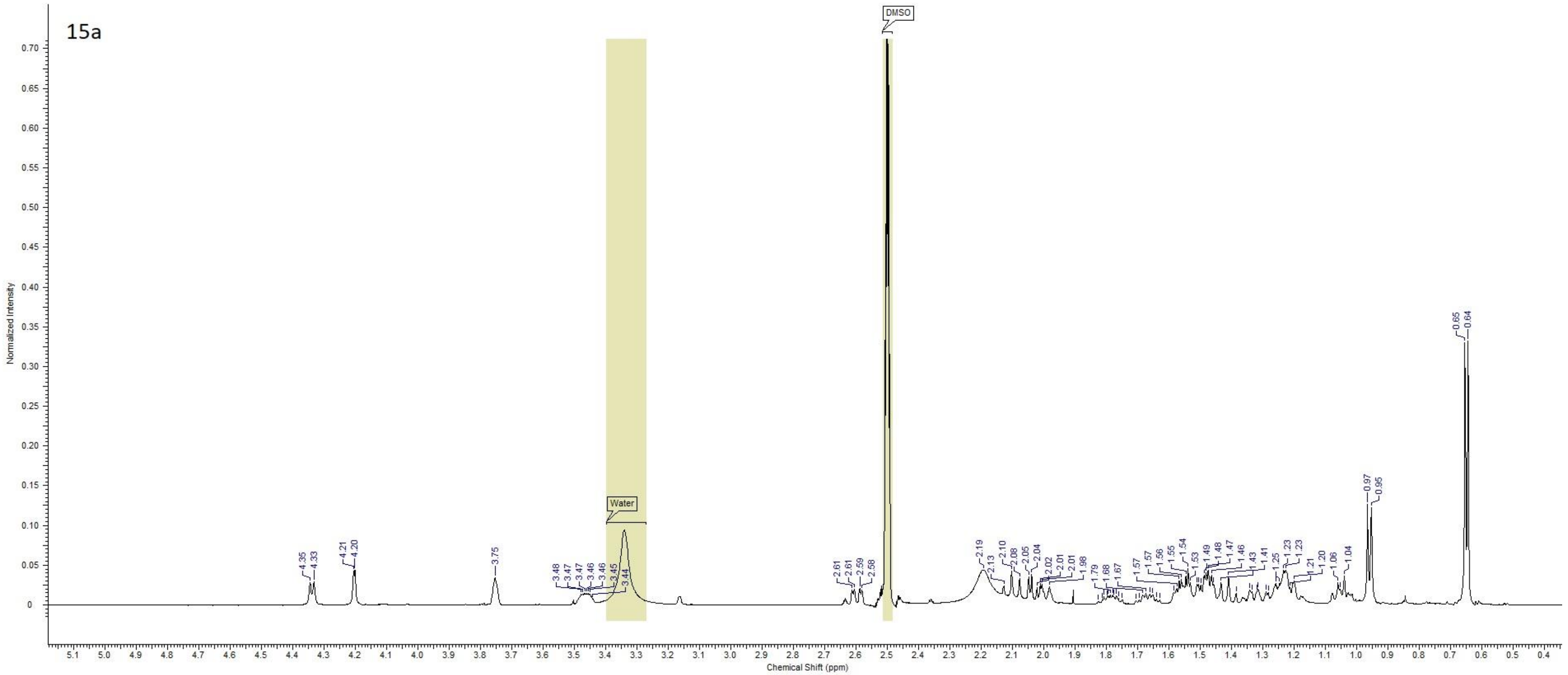
15a
DMSO
Water
Normalized Intensity
Chemical Shift (ppm)

15a

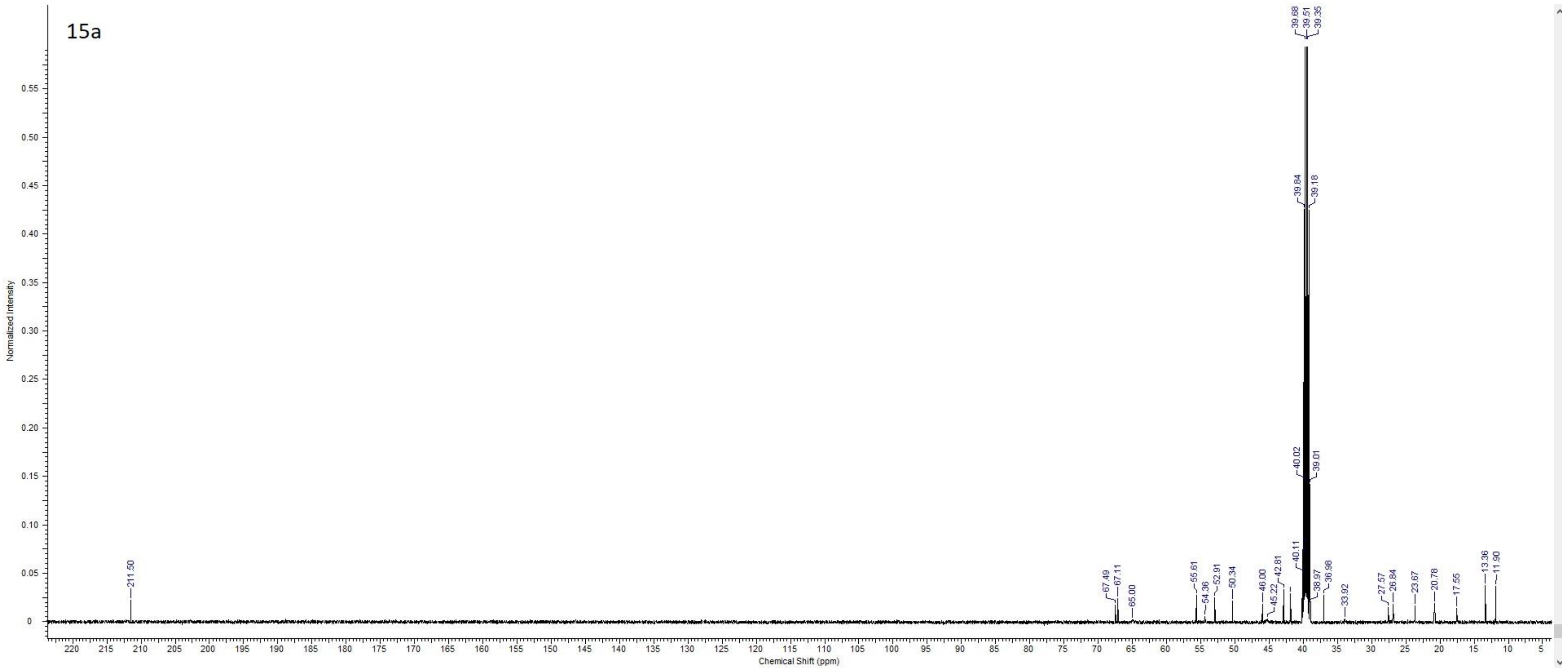
211.50
67.49
67.11
65.00
55.61
54.36
52.91
50.34
46.00
45.22
42.81
40.11
40.02
39.84
39.68
39.51
39.35
39.18
39.01
38.97
36.98
33.92
27.57
26.84
23.67
20.78
17.55
13.36
11.90
Normalized Intensity
Chemical Shift (ppm)

Elemental Composition

File Edit View Process Help

Single Mass Analysis
Tolerance = 1.0 PPM / DBE: min = -1.5, max = 50.0
Element prediction: Off
Number of isotope peaks used for i-FIT = 3
Monoisotopic Mass, Even Electron Ions
508 formula(e) evaluated with 1 results within limits (up to 50 best isotopic matches for each mass)
Elements Used:

Mass	Calc. Mass	mDa	PPM	DBE	Formula	i-FIT	i-FIT Norm	Fit Conf %	C	H	N	O
392.3164	392.3165	-0.1	-0.3	4.5	C24 H42 N O3	843.9	n/a	n/a	24	42	1	3

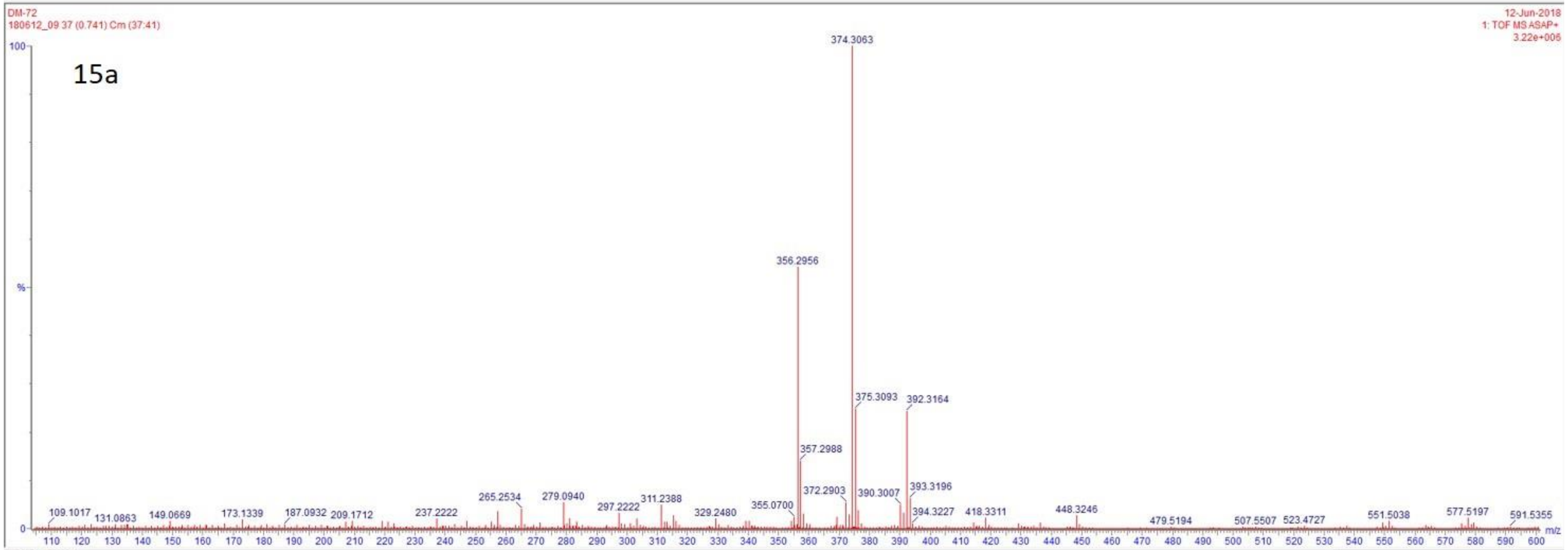

For Help, press F1

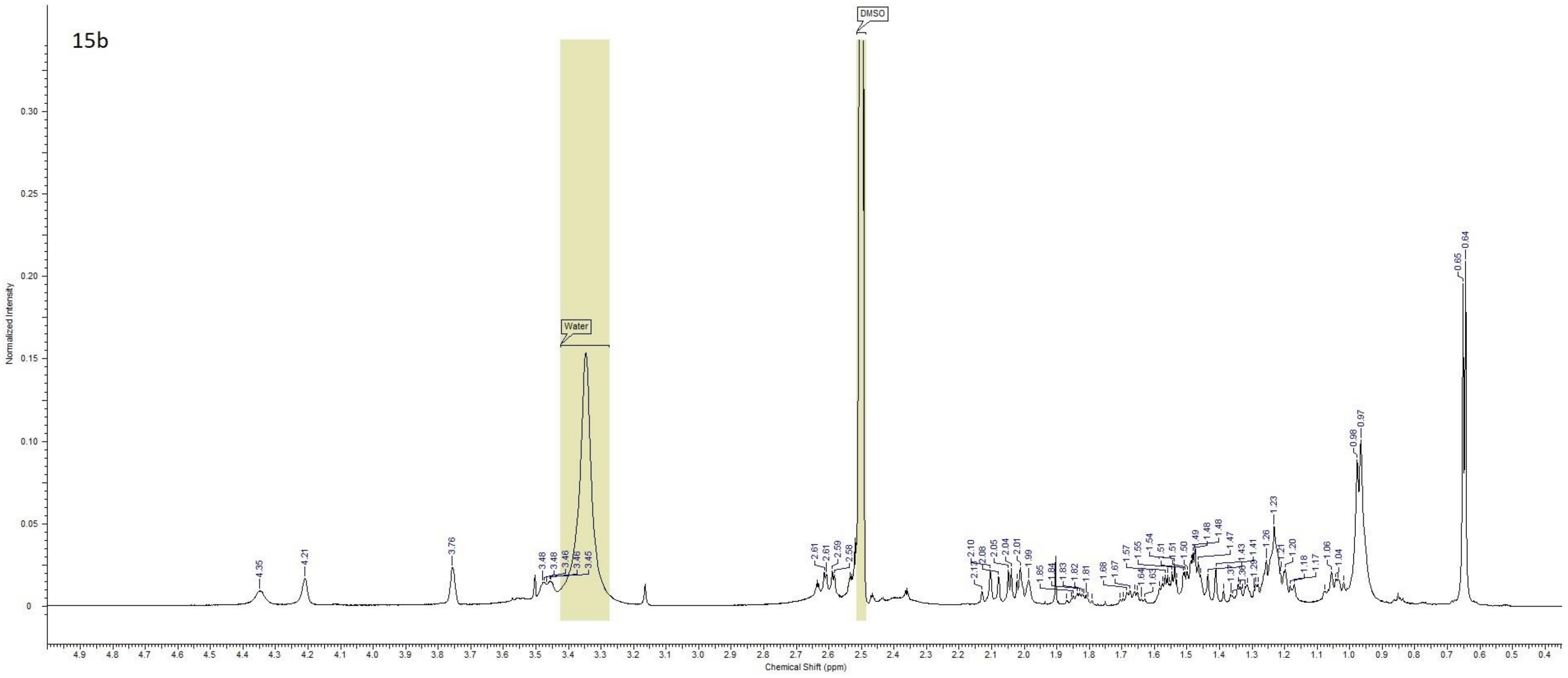
15b
Water
DMSO
Normalized Intensity
Chemical Shift (ppm)

15b

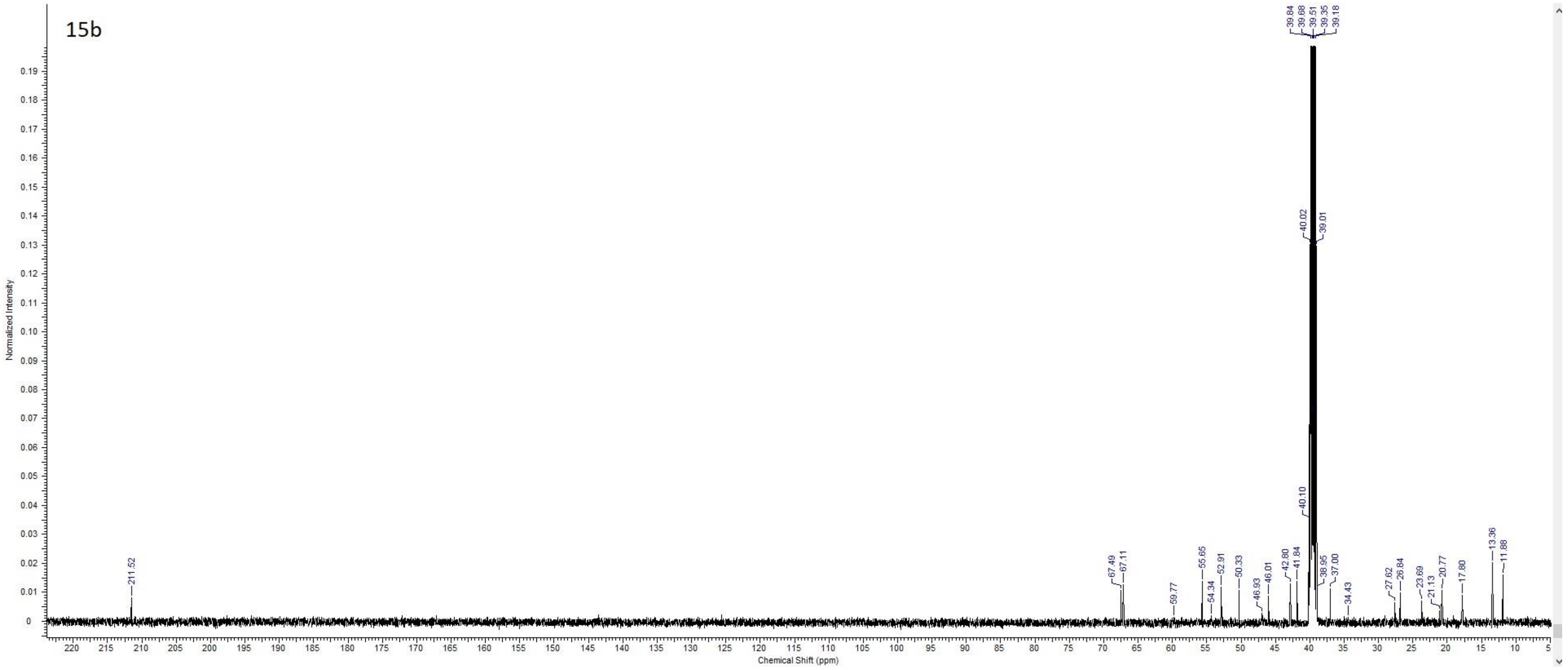

Normalized Intensity
Chemical Shift (ppm)
211.52
67.49
67.11
59.77
55.65
54.34
52.91
50.33
46.93
46.01
42.80
41.84
40.10
40.02
39.84
39.68
39.51
39.35
39.18
39.01
38.95
37.00
34.43
27.62
26.84
23.69
21.13
20.77
17.80
13.36
11.88

Elemental Composition

File Edit View Process Help

Single Mass Analysis
Tolerance = 1.0 PPM / DBE: min = -1.5, max = 50.0
Element prediction: Off
Number of isotope peaks used for i-FIT = 3
Monoisotopic Mass, Even Electron Ions
548 formula(e) evaluated with 1 results within limits (up to 50 best isotopic matches for each mass)
Elements Used:

Mass	Calc. Mass	mDa	PPM	DBE	Formula	i-FIT	i-FIT Norm	Fit Conf %	C	H	N	O
420.3476	420.3478	-0.2	-0.5	4.5	C26 H46 N O3	813.1	n/a	n/a	26	46	1	3

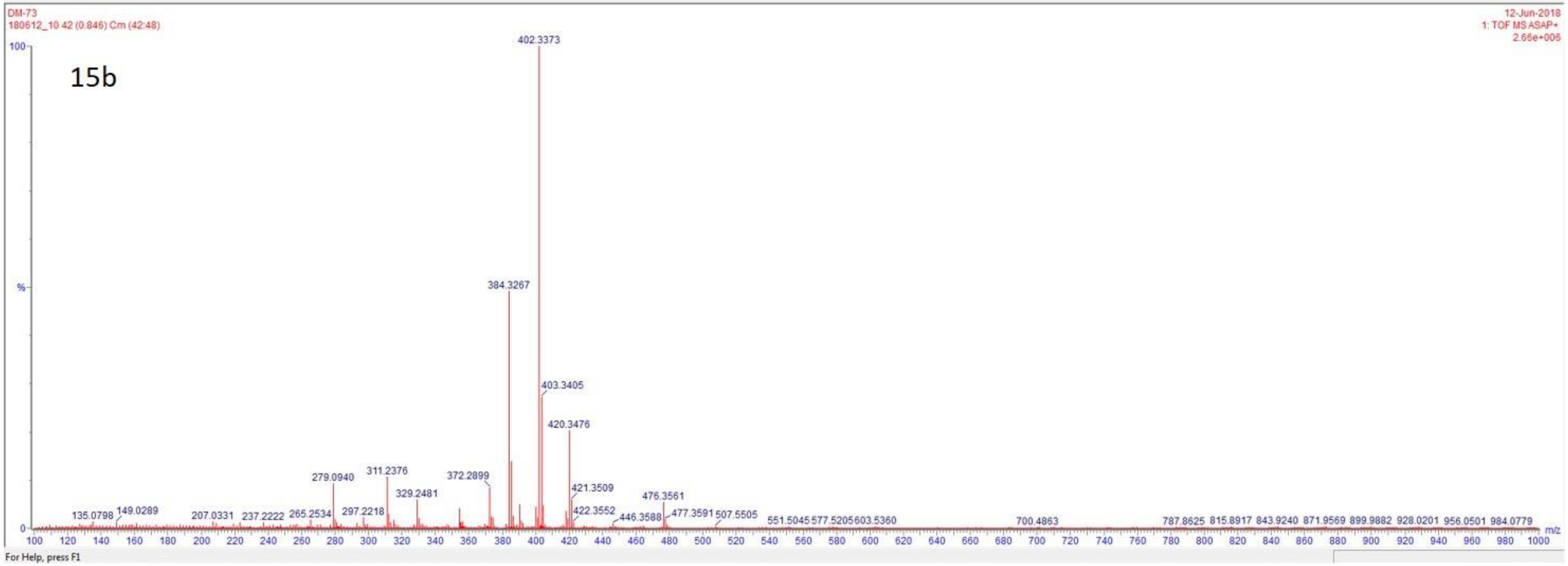

For Help, press F1

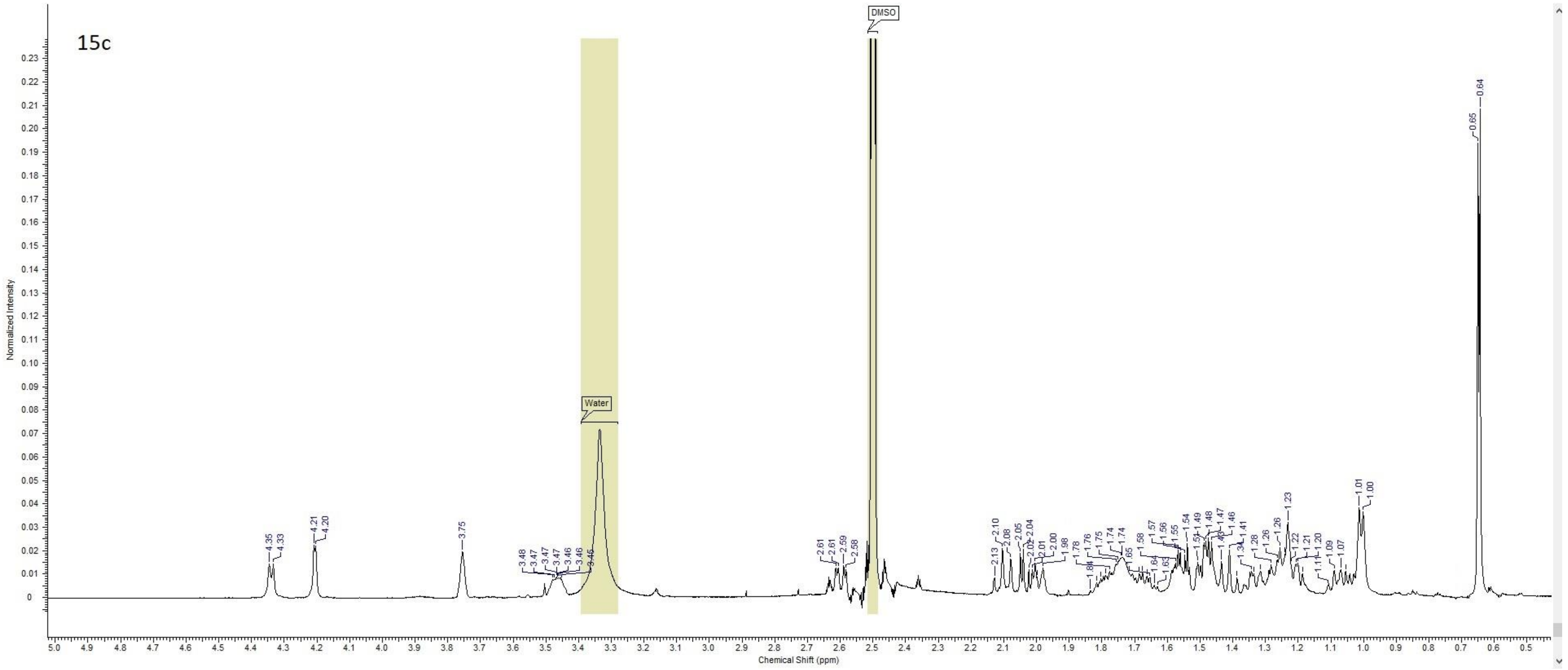
15c
Water
DMSO
Normalized Intensity
Chemical Shift (ppm)

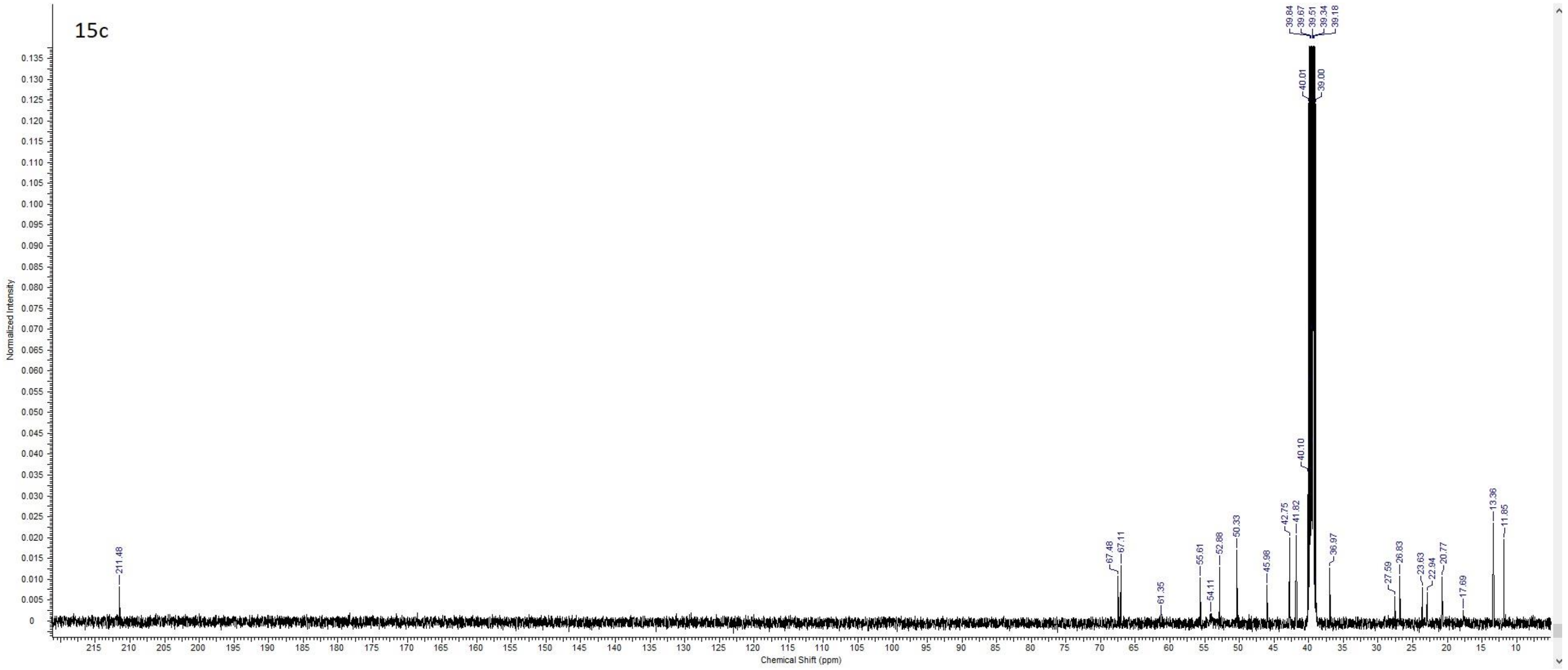
15c
Normalized Intensity
Chemical Shift (ppm)
211.48
67.48
67.11
61.35
55.61
54.11
52.88
50.33
45.98
42.75
41.82
40.10
40.01
39.84
39.67
39.51
39.34
39.18
39.00
36.97
27.59
26.83
23.63
22.94
20.77
17.69
13.36
11.85

Elemental Composition

File Edit View Process Help

Single Mass Analysis
Tolerance = 1.0 PPM / DBE: min = -1.5, max = 50.0
Element prediction: Off
Number of isotope peaks used for i-FIT = 3
Monoisotopic Mass, Even Electron Ions
544 formula(e) evaluated with 1 results within limits (up to 50 best isotopic matches for each mass)
Elements Used:

Mass	Calc. Mass	mDa	PPM	DBE	Formula	i-FIT	i-FIT Norm	Fit Conf %	C	H	N	O
418.3322	418.3321	0.1	0.2	5.5	C26 H44 N O3	1145.5	n/a	n/a	26	44	1	3

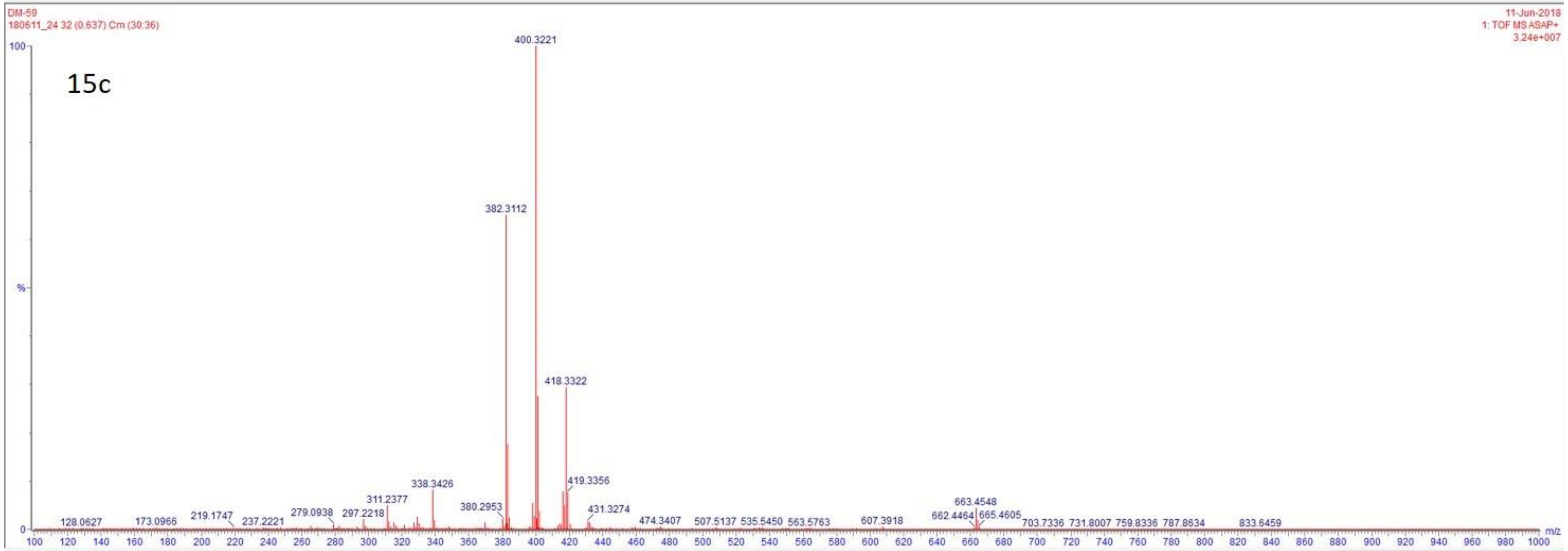

For Help, press F1

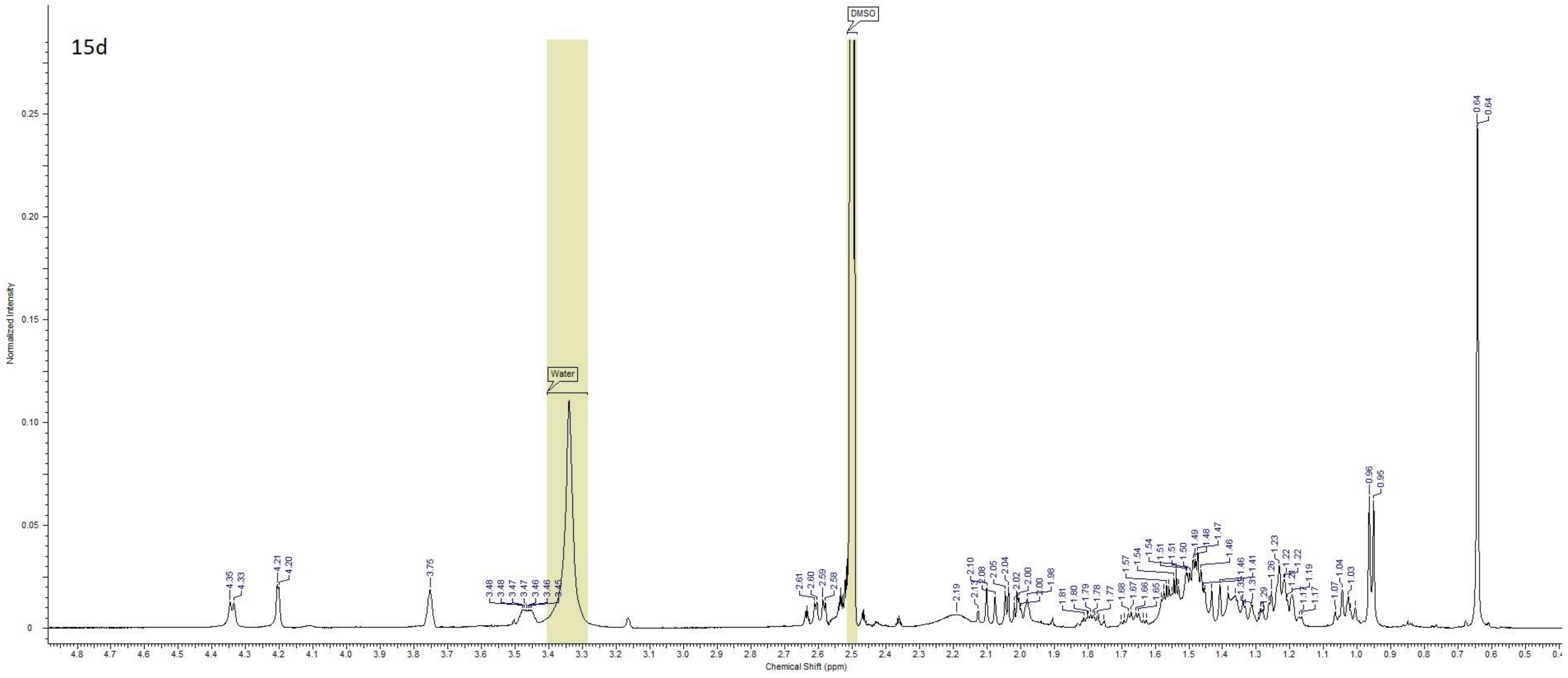

15d
Water
DMSO
Normalized Intensity
Chemical Shift (ppm)
4.35
4.33
4.21
4.20
3.75
3.48
3.48
3.47
3.46
3.46
3.45
2.61
2.60
2.59
2.58
2.19
0.96
0.95
0.64
0.64

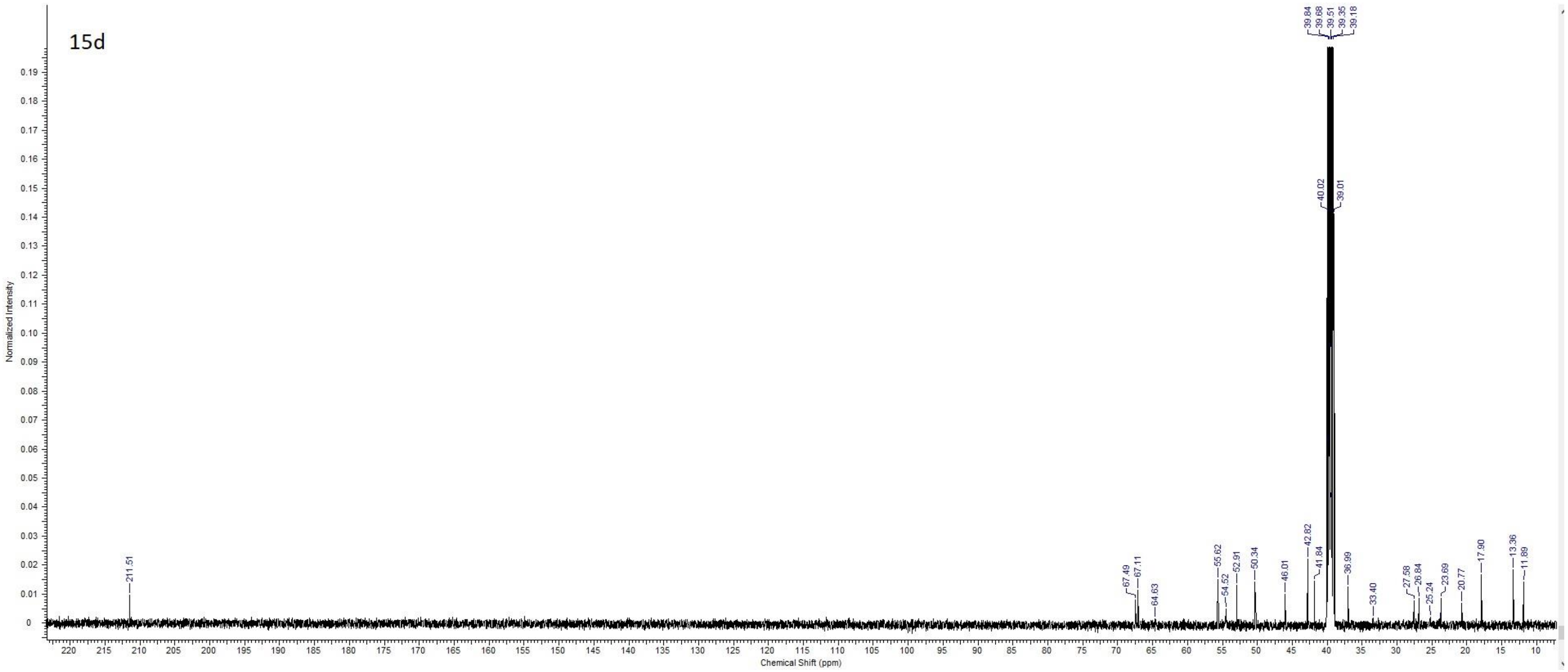
15d
Normalized Intensity
Chemical Shift (ppm)
211.51
67.49
67.11
64.63
55.62
54.52
52.91
50.34
46.01
42.82
41.84
40.02
39.84
39.68
39.51
39.35
39.18
39.01
36.99
33.40
27.58
26.84
25.24
23.69
20.77
17.90
13.36
11.89

Elemental Composition

File Edit View Process Help

Single Mass Analysis

Tolerance = 1.0 PPM / DBE: min = -1.5, max = 50.0

Element prediction: Off

Number of isotope peaks used for i-FIT = 3

Monoisotopic Mass, Even Electron Ions

566 formula(e) evaluated with 1 results within limits (up to 50 best isotopic matches for each mass)

Elements Used:

Mass	Calc. Mass	mDa	PPM	DBE	Formula	i-FIT	i-FIT Norm	Fit Conf %	C	H	N	O
432.3474	432.3478	-0.4	-0.9	5.5	C27 H46 N O3	820.3	n/a	n/a	27	46	1	3

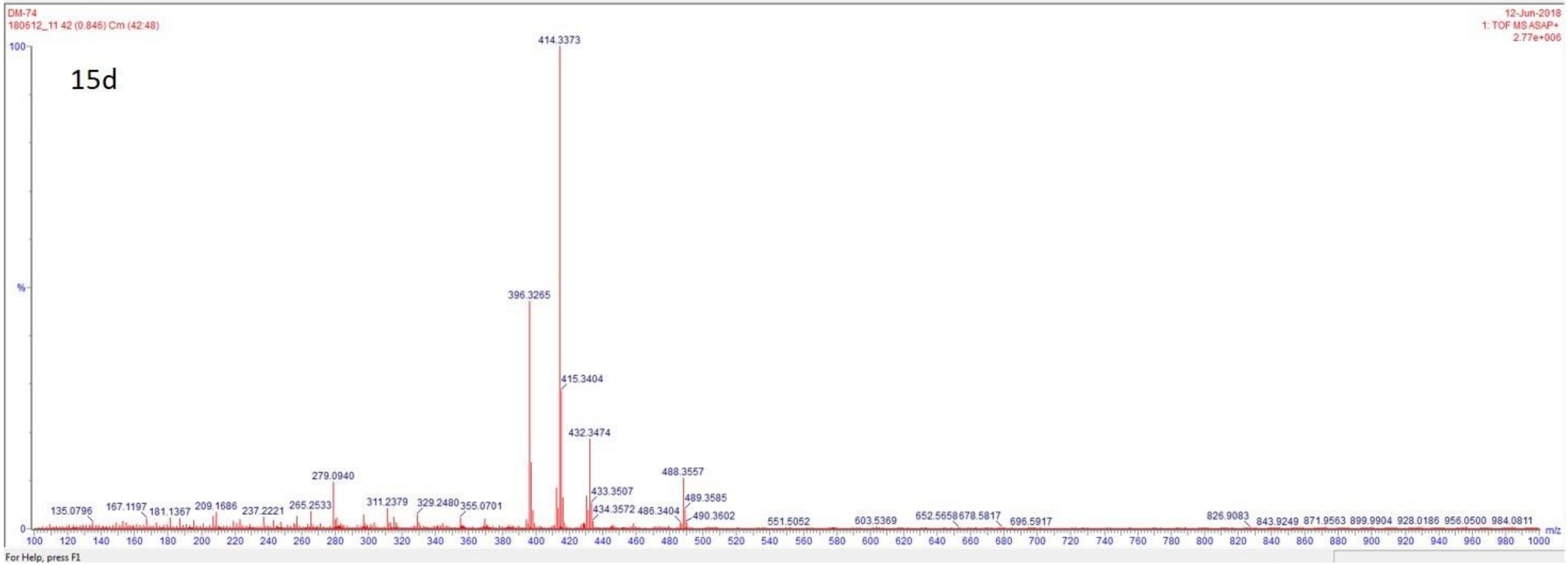

For Help, press F1

15e

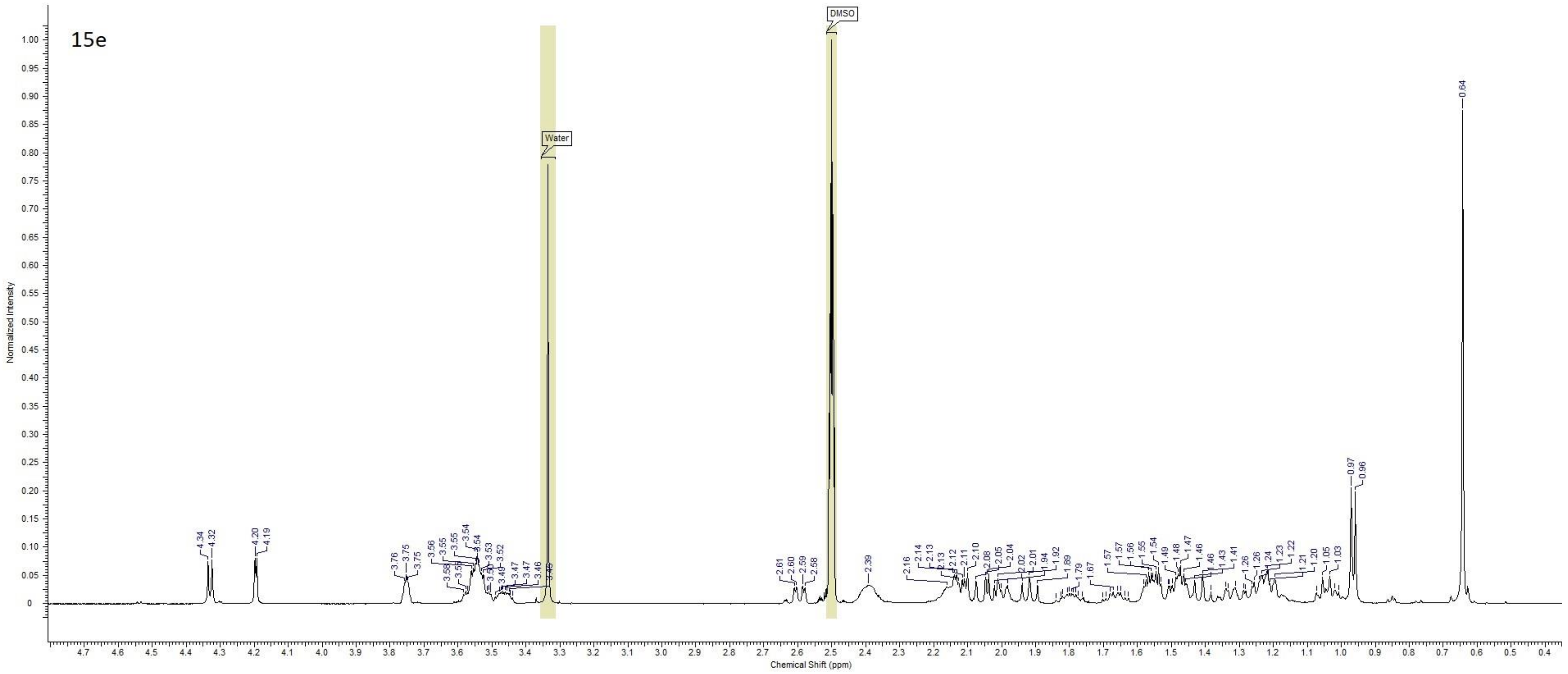

15e

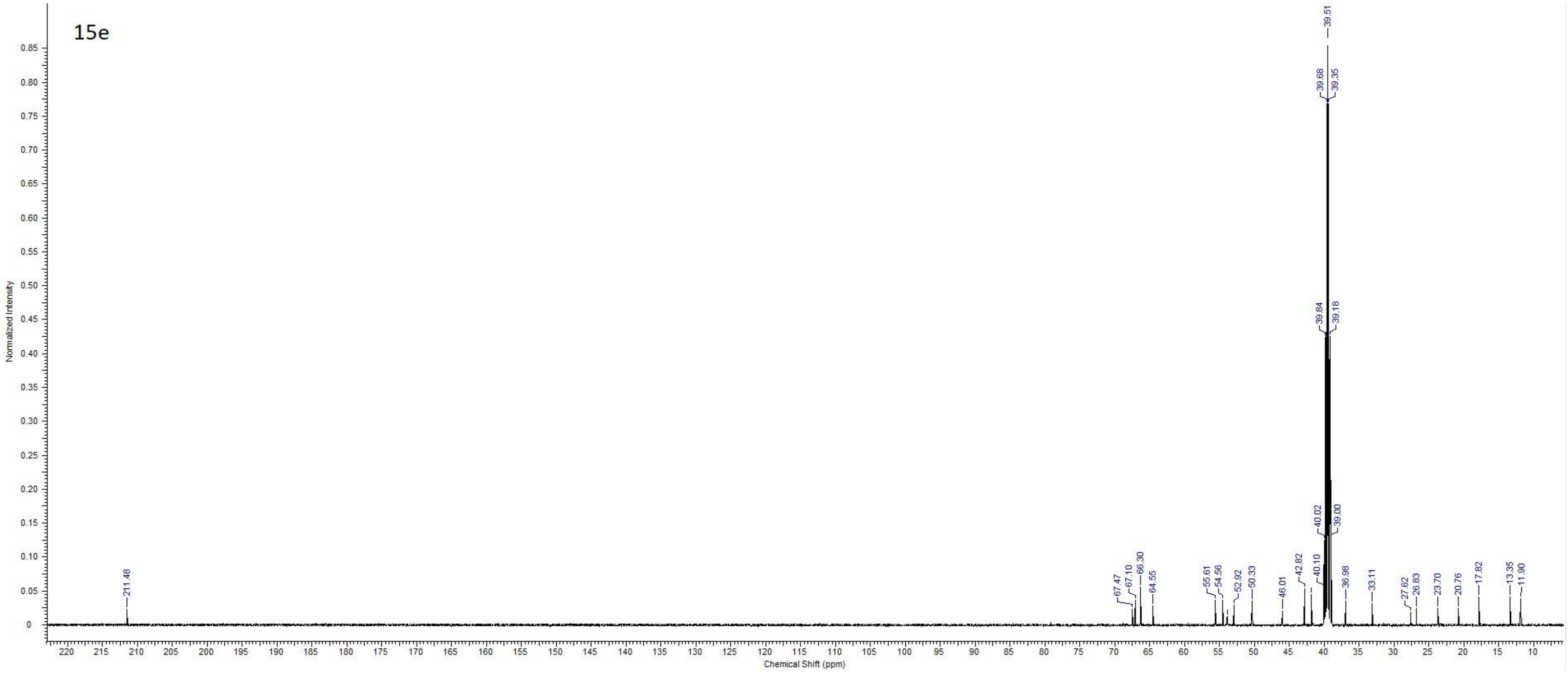
211.48
67.47
67.10
66.30
64.55
55.61
54.56
52.92
50.33
46.01
42.82
40.10
40.02
39.84
39.68
39.51
39.35
39.18
39.00
36.98
33.11
27.62
26.83
23.70
20.76
17.82
13.35
11.90
Normalized Intensity
Chemical Shift (ppm)

Elemental Composition

File Edit View Process Help

Single Mass Analysis

Tolerance = 1.0 PPM / DBE: min = -1.5, max = 50.0

Element prediction: Off

Number of isotope peaks used for i-FIT = 3

Monoisotopic Mass, Even Electron Ions

569 formula(e) evaluated with 1 results within limits (up to 50 best isotopic matches for each mass)

Elements Used:

Mass	Calc. Mass	mDa	PPM	DBE	Formula	i-FIT	i-FIT Norm	Fit Conf %	C	H	N	O
434.3267	434.3270	-0.3	-0.7	5.5	C26 H44 N O4	834.5	n/a	n/a	26	44	1	4

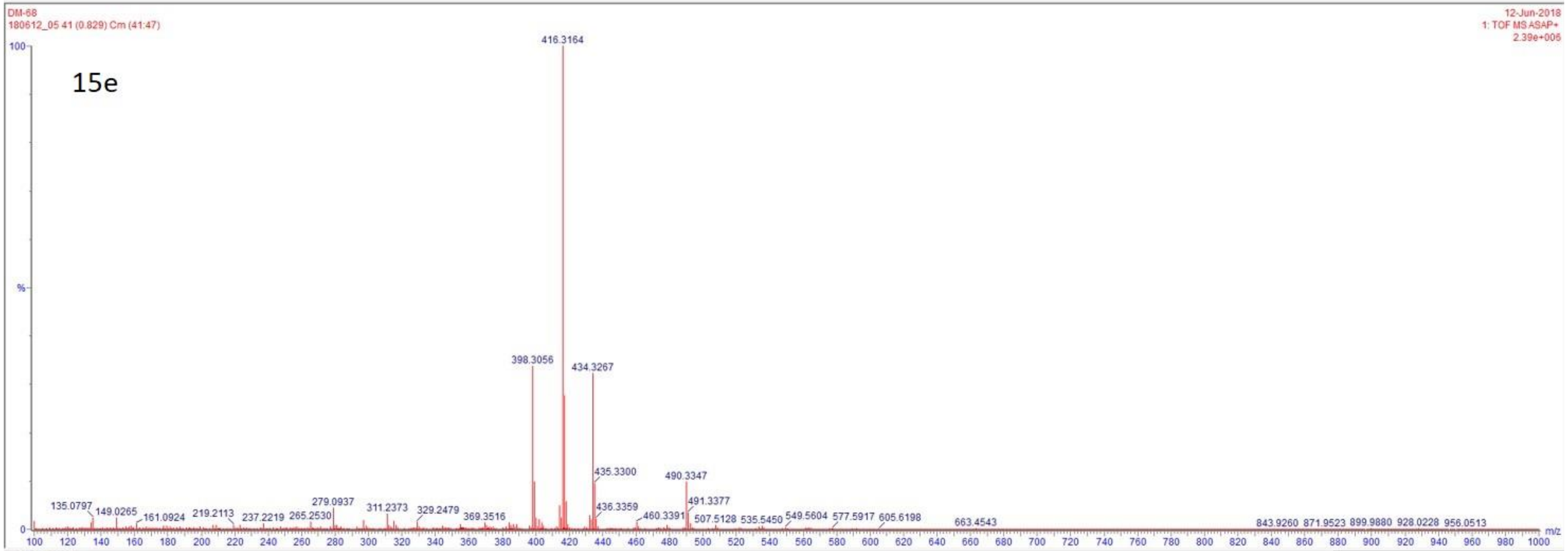

For Help, press F1

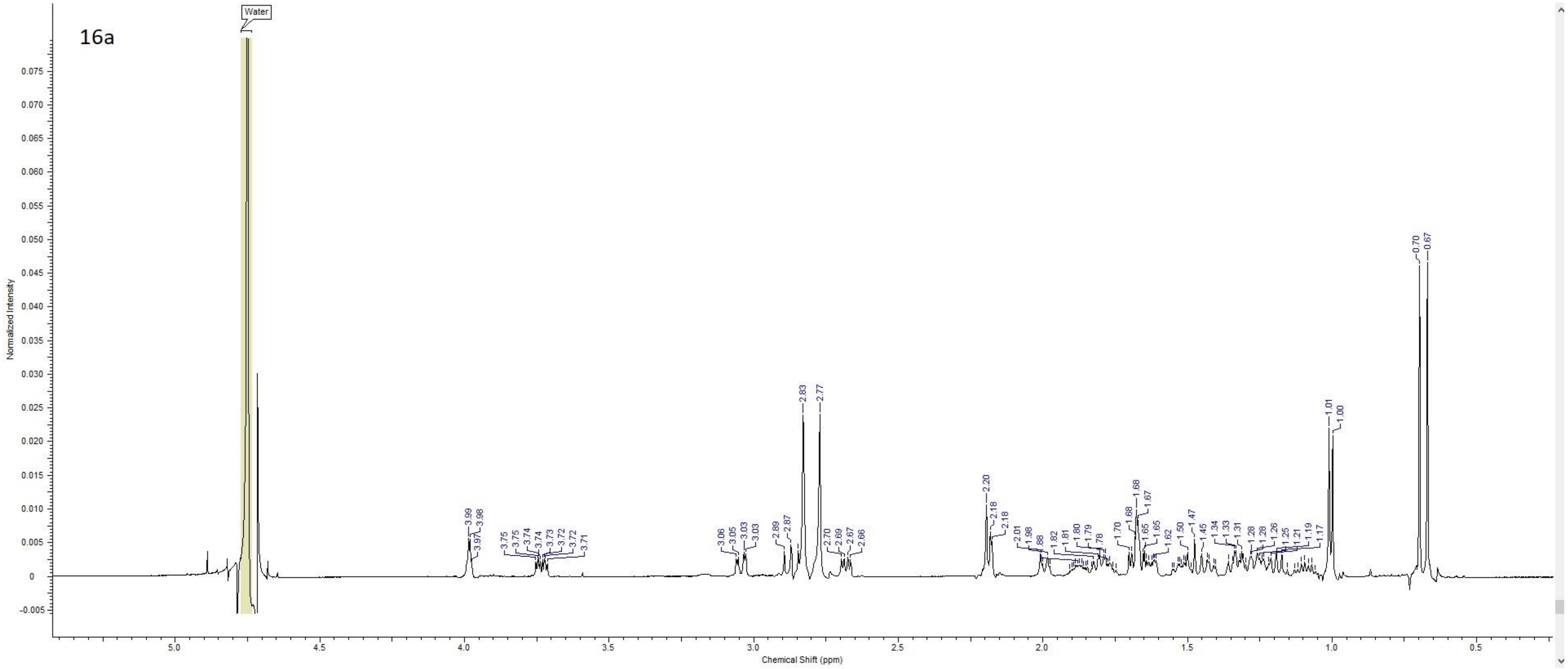
16a
Water
Normalized Intensity
Chemical Shift (ppm)

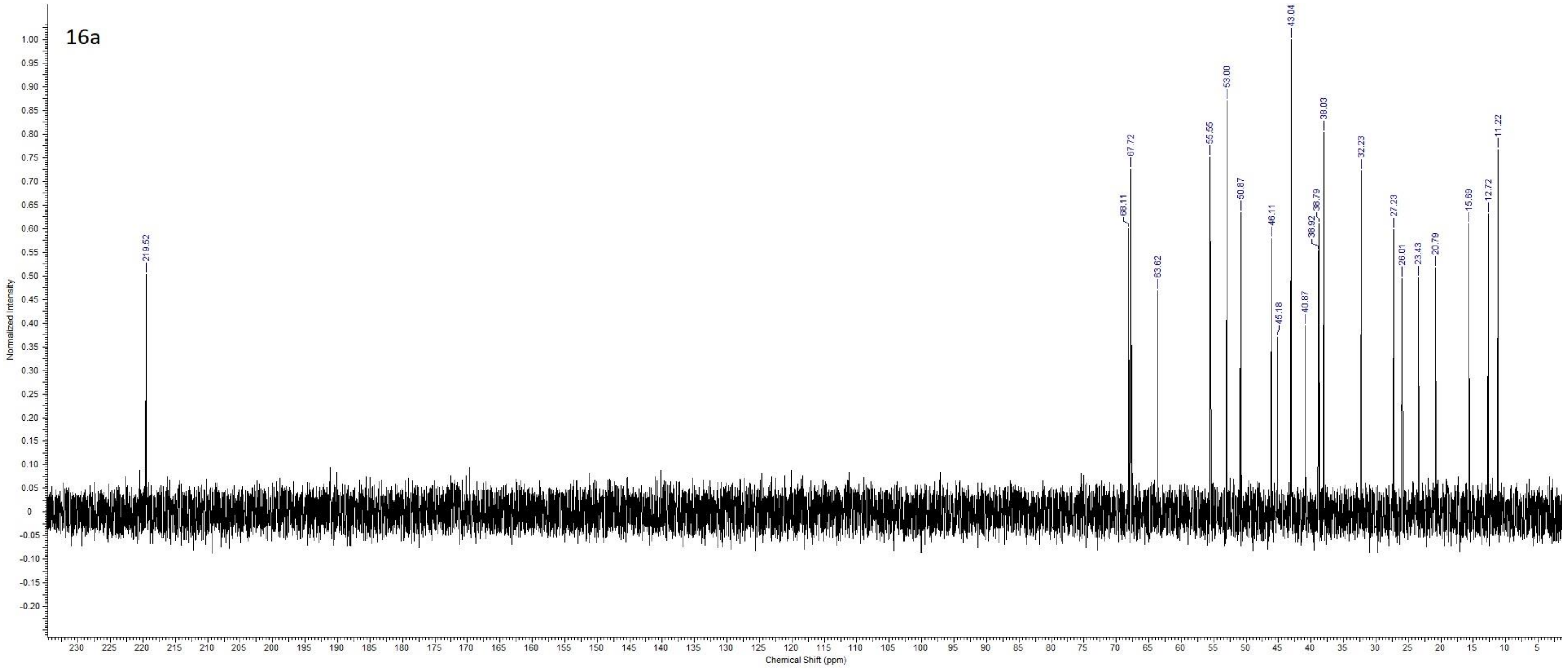
16a
219.52
68.11
67.72
63.62
55.55
53.00
50.87
46.11
45.18
43.04
40.87
38.92
38.79
38.03
32.23
27.23
26.01
23.43
20.79
15.69
12.72
11.22
Normalized Intensity
Chemical Shift (ppm)

Elemental Composition

File Edit View Process Help

Single Mass Analysis

Tolerance = 1.0 PPM / DBE: min = -1.5, max = 50.0

Element prediction: Off

Number of isotope peaks used for i-FIT = 3

Monoisotopic Mass, Even Electron Ions

508 formula(e) evaluated with 1 results within limits (up to 50 best isotopic matches for each mass)

Elements Used:

Mass	Calc. Mass	mDa	PPM	DBE	Formula	i-FIT	i-FIT Norm	Fit Conf %	C	H	N	O
392.3164	392.3165	-0.1	-0.3	4.5	C24 H42 N O3	976.1	n/a	n/a	24	42	1	3

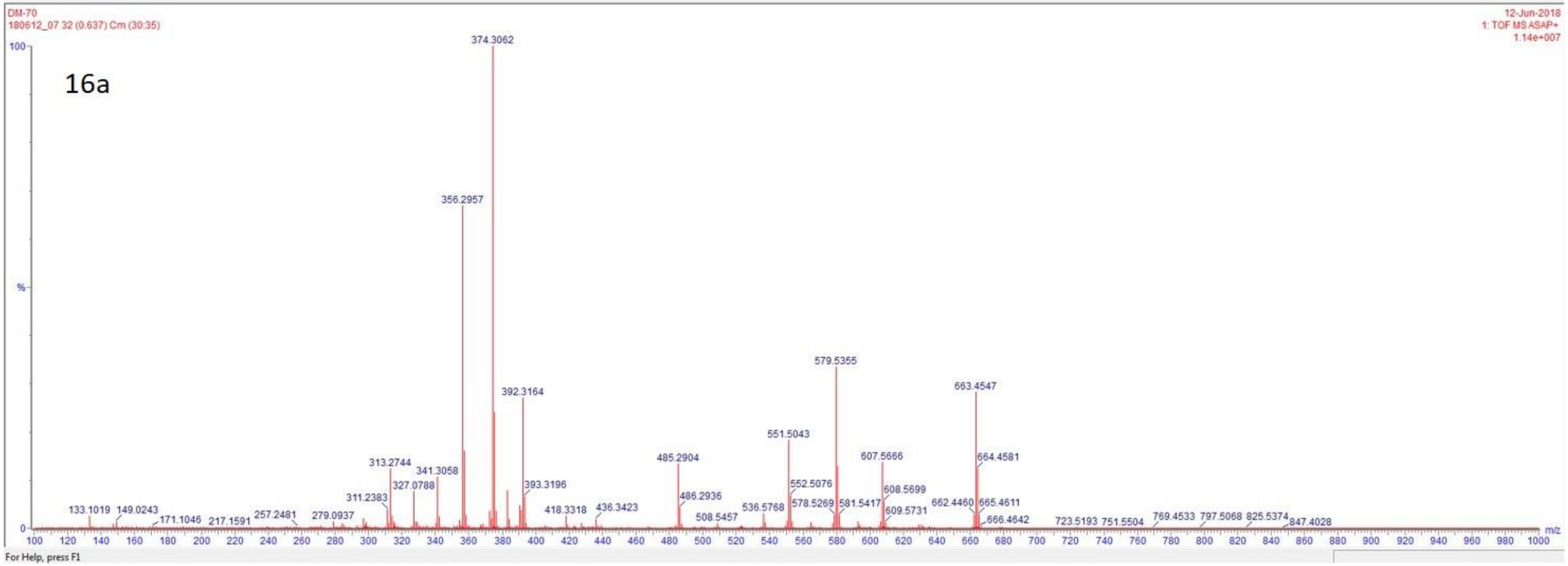

For Help, press F1

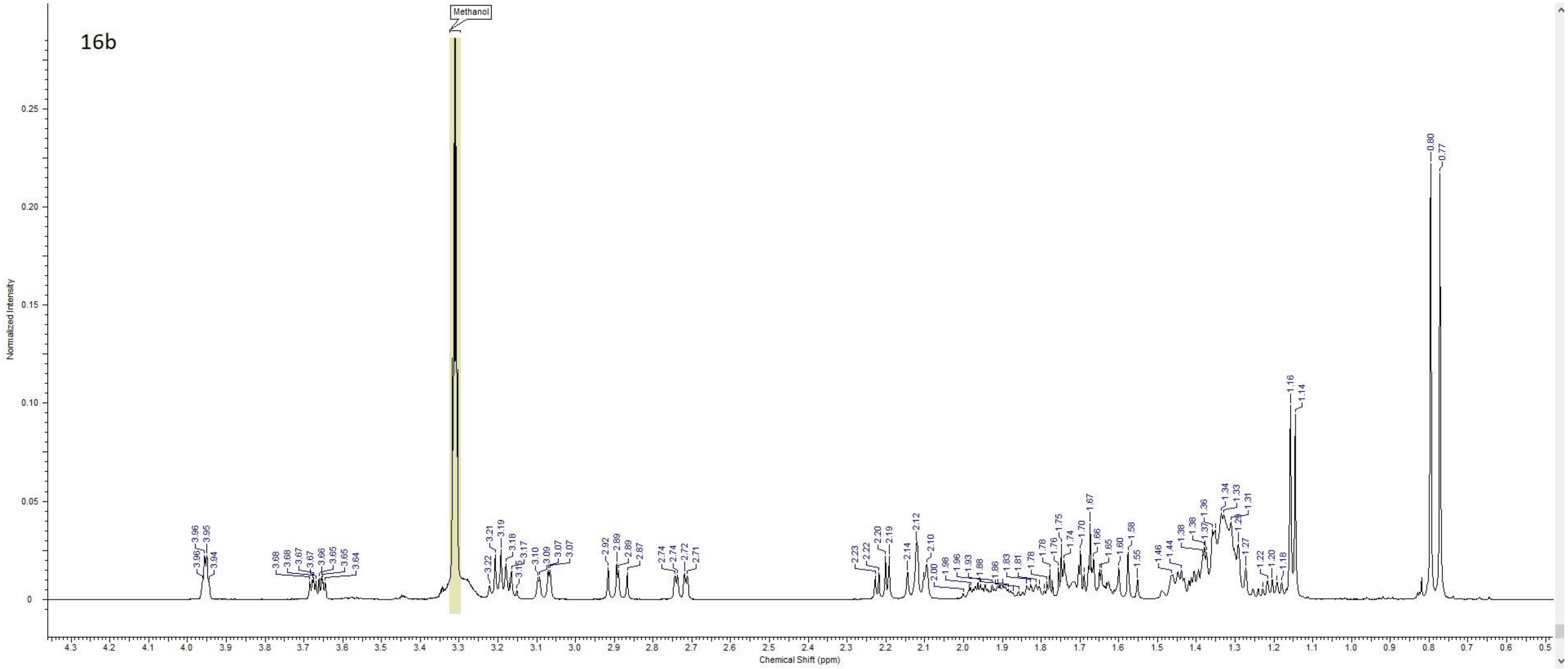
16b
Methanol
Normalized Intensity
Chemical Shift (ppm)

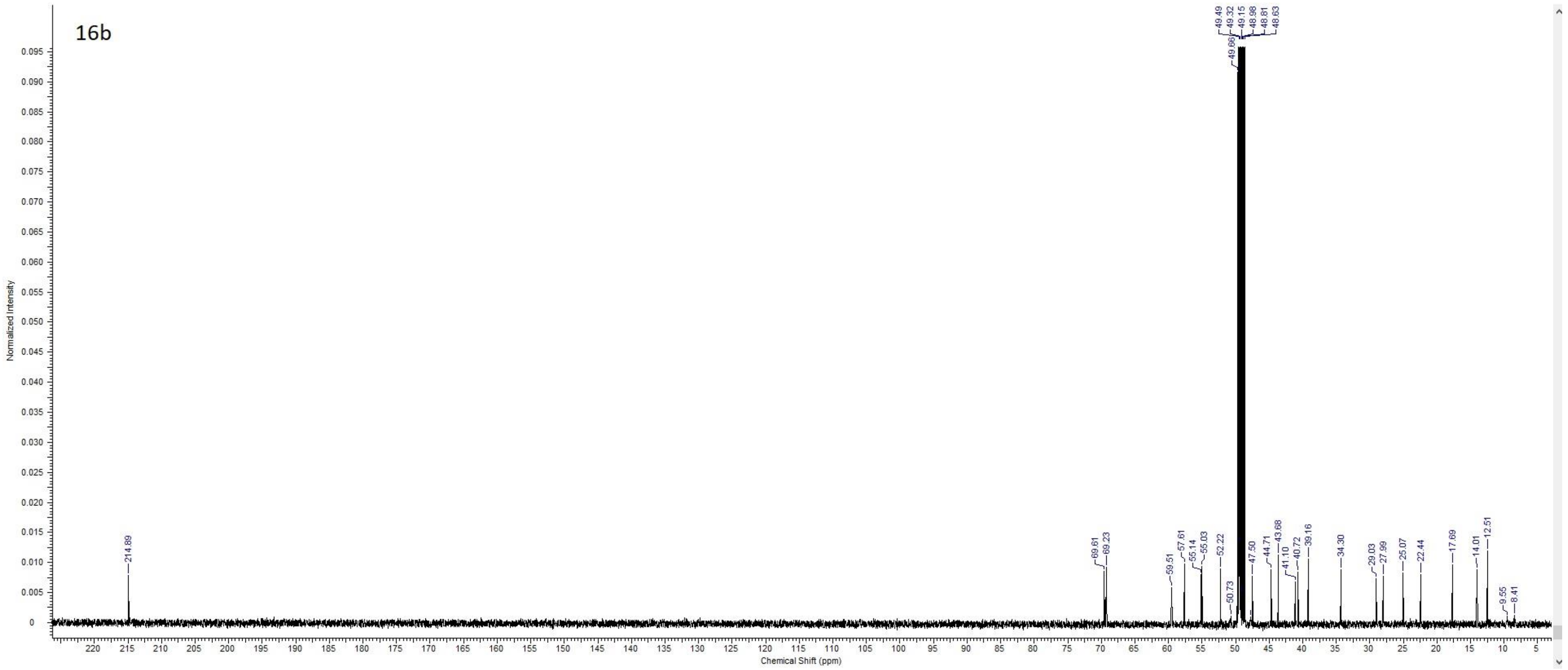
16b
214.89
69.61
69.23
59.51
57.61
55.14
55.03
52.22
50.73
49.66
49.49
49.32
49.15
48.98
48.81
48.63
47.50
44.71
43.68
41.10
40.72
39.16
34.30
29.03
27.99
25.07
22.44
17.69
14.01
12.51
9.55
8.41
Normalized Intensity
Chemical Shift (ppm)

Elemental Composition

File Edit View Process Help

Single Mass Analysis

Tolerance = 1.0 PPM / DBE: min = -1.5, max = 50.0

Element prediction: Off

Number of isotope peaks used for i-FIT = 3

Monoisotopic Mass, Even Electron Ions

548 formula(e) evaluated with 1 results within limits (up to 50 best isotopic matches for each mass)

Elements Used:

Mass	Calc. Mass	mDa	PPM	DBE	Formula	i-FIT	i-FIT Norm	Fit Conf %	C	H	N	O
420.3480	420.3478	0.2	0.5	4.5	C26 H46 N O3	1230.4	n/a	n/a	26	46	1	3

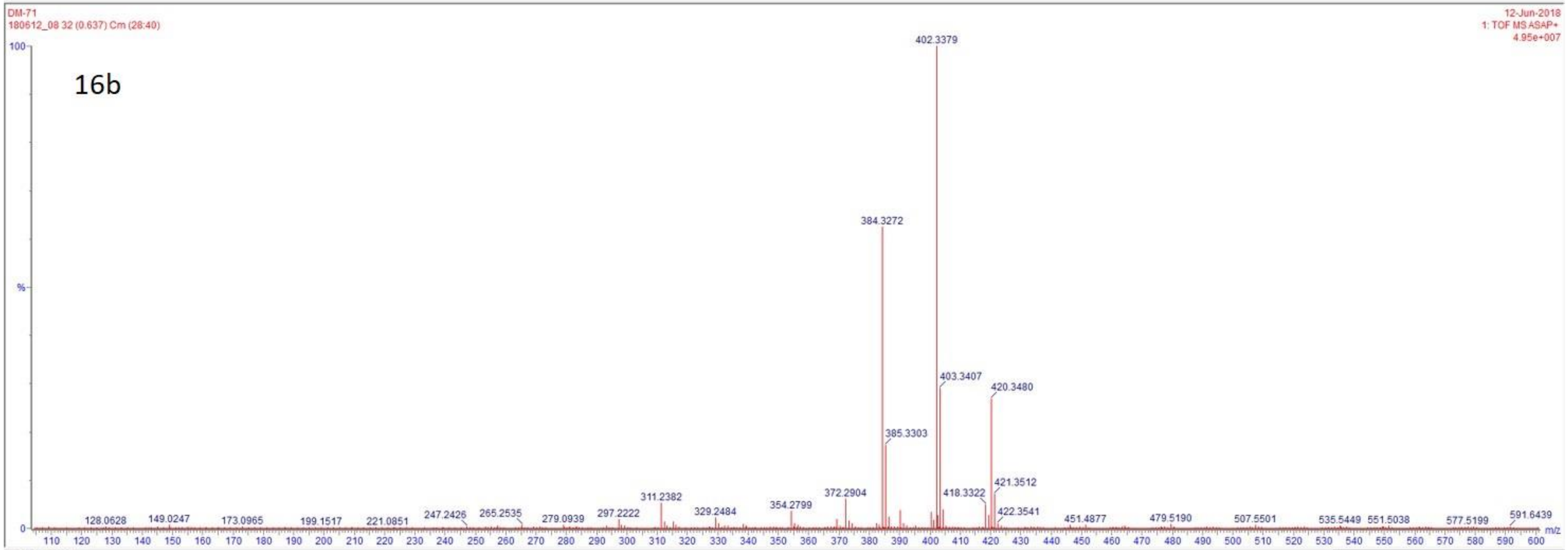

For Help, press F1

16c

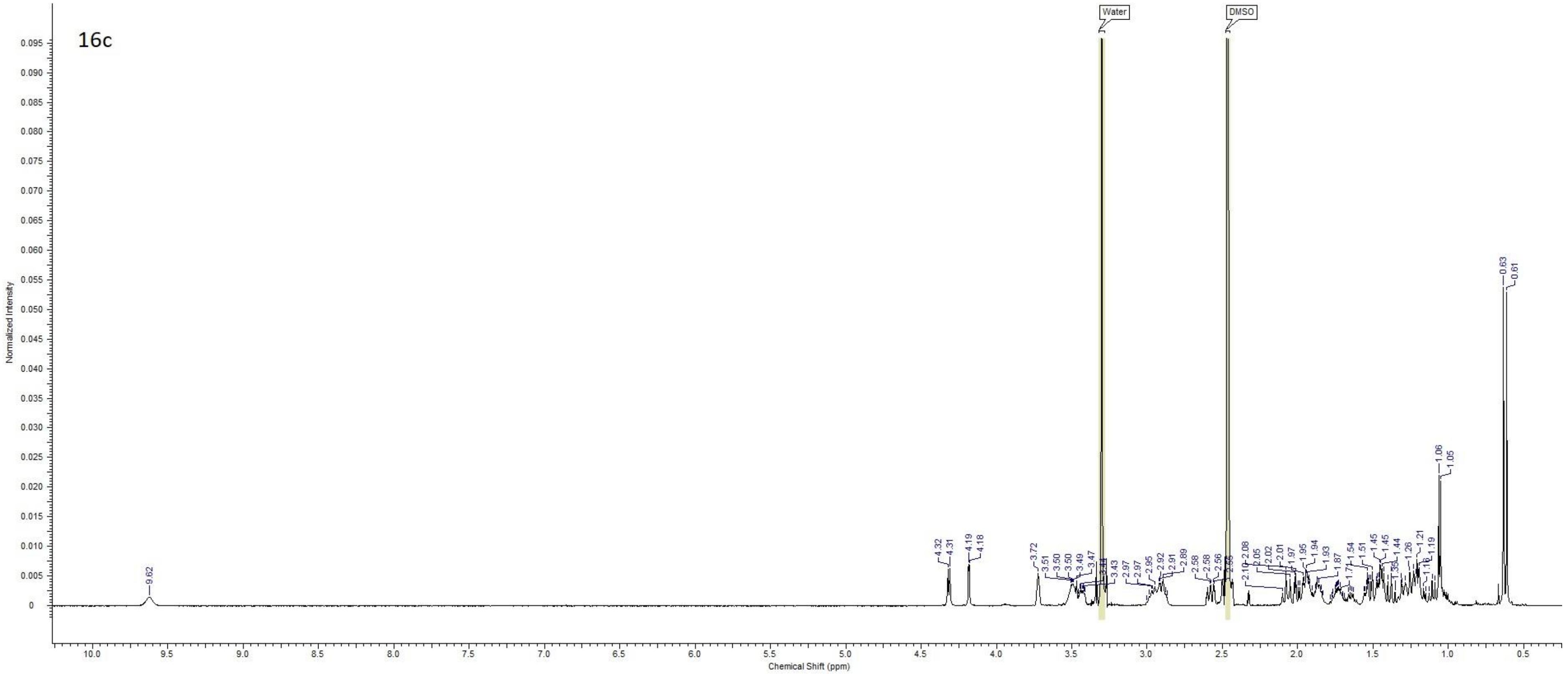
Water
DMSO
Normalized Intensity
Chemical Shift (ppm)
9.62
4.32
4.31
4.19
4.18
3.72
1.06
1.05
0.63
0.61

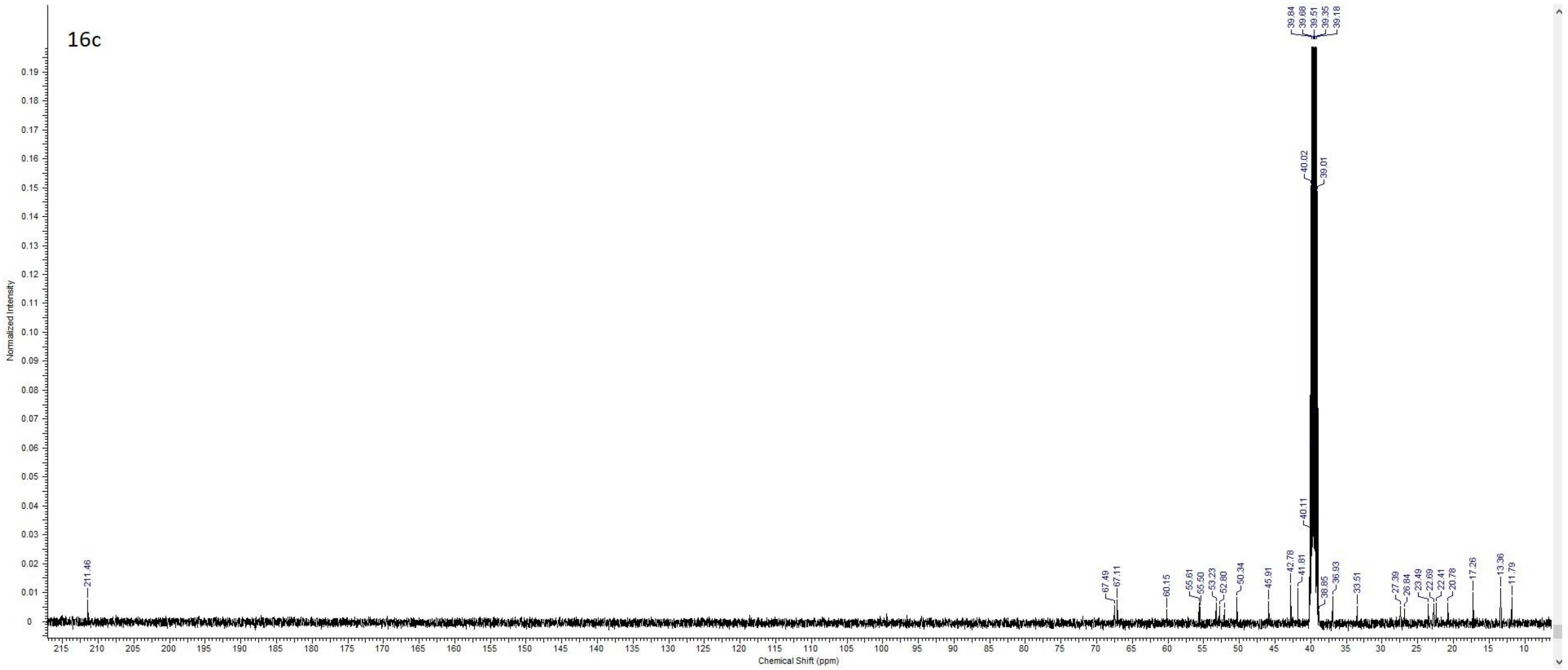
16c
Normalized Intensity
Chemical Shift (ppm)
211.46
67.49
67.11
60.15
55.61
55.50
53.23
52.80
50.34
45.91
42.78
41.81
40.11
40.02
39.84
39.68
39.51
39.35
39.18
39.01
38.85
36.93
33.51
27.39
26.84
23.49
22.69
22.41
20.78
17.26
13.36
11.79

Elemental Composition

File Edit View Process Help

Single Mass Analysis

Tolerance = 1.0 PPM / DBE: min = -1.5, max = 50.0

Element prediction: Off

Number of isotope peaks used for i-FIT = 3

Monoisotopic Mass, Even Electron Ions

544 formula(e) evaluated with 1 results within limits (up to 50 best isotopic matches for each mass)

Elements Used:

Mass	Calc. Mass	mDa	PPM	DBE	Formula	i-FIT	i-FIT Norm	Fit Conf %	C	H	N	O
418.3321	418.3321	0.0	0.0	5.5	C26 H44 N O3	1120.9	n/a	n/a	26	44	1	3

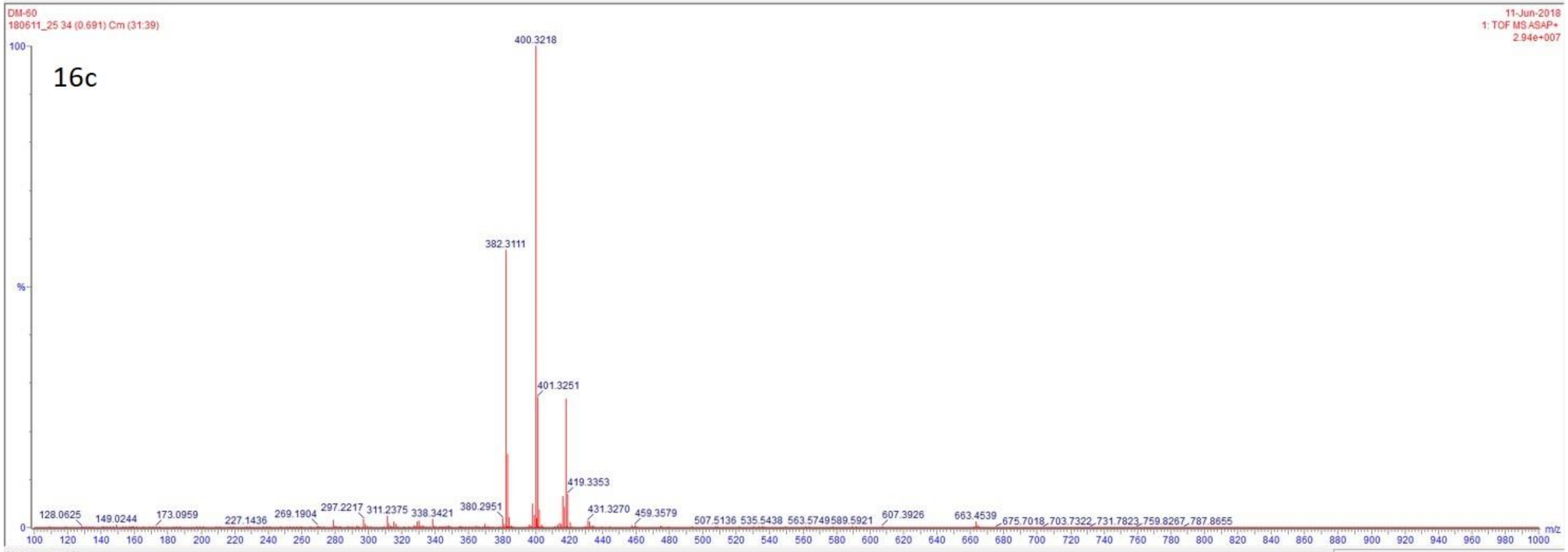

For Help, press F1

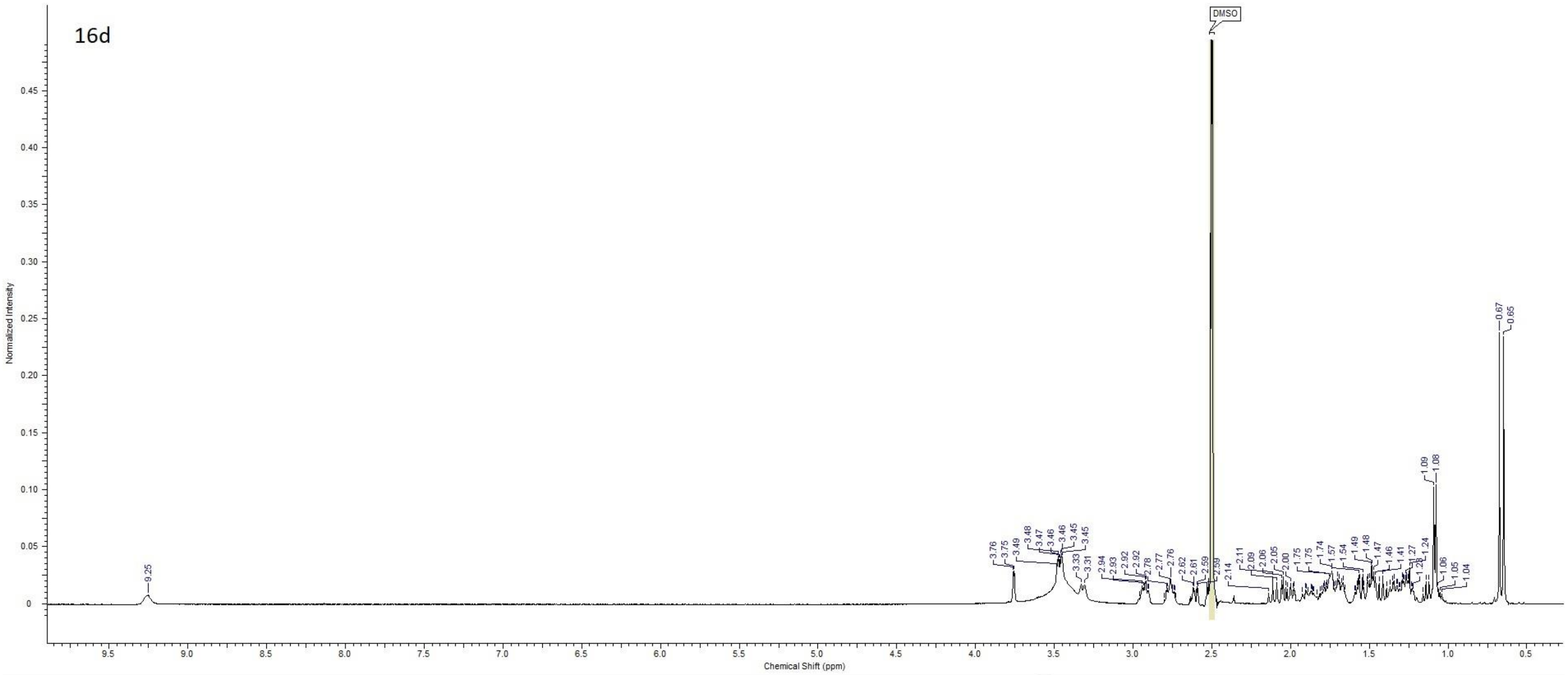
16d
DMSO
Normalized Intensity
Chemical Shift (ppm)
9.25
3.76
3.75
3.49
3.48
3.47
3.46
3.46
3.45
3.45
3.33
3.31
2.94
2.93
2.92
2.92
2.78
2.77
2.76
2.62
2.61
2.59
2.59
2.14
2.11
2.09
2.06
2.05
2.00
1.75
1.75
1.74
1.57
1.54
1.49
1.48
1.47
1.46
1.41
1.27
1.24
1.09
1.08
1.06
1.05
1.04
0.67
0.65

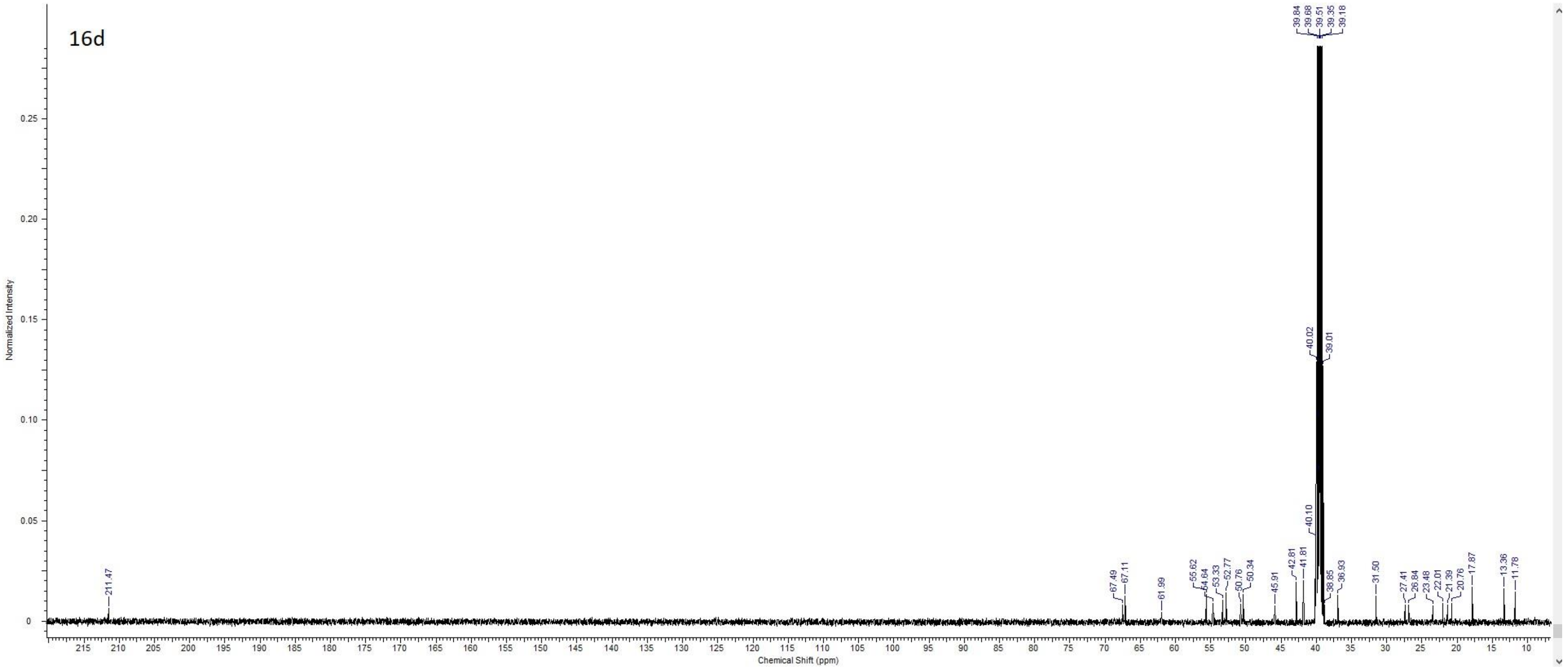
16d
211.47
67.49
67.11
61.99
55.62
54.64
53.33
52.77
50.76
50.34
45.91
42.81
41.81
40.10
40.02
39.84
39.68
39.51
39.35
39.18
39.01
38.85
36.93
31.50
27.41
26.84
23.48
22.01
21.39
20.76
17.87
13.36
11.78
Normalized Intensity
Chemical Shift (ppm)

Elemental Composition

File Edit View Process Help

Single Mass Analysis

Tolerance = 1.0 PPM / DBE: min = -1.5, max = 50.0

Element prediction: Off

Number of isotope peaks used for i-FIT = 3

Monoisotopic Mass, Even Electron Ions

566 formula(e) evaluated with 1 results within limits (up to 50 best isotopic matches for each mass)

Elements Used:

Mass	Calc. Mass	mDa	PPM	DBE	Formula	i-FIT	i-FIT Norm	Fit Conf %	C	H	N	O
432.3475	432.3478	-0.3	-0.7	5.5	C27 H46 N O3	1156.5	n/a	n/a	27	46	1	3

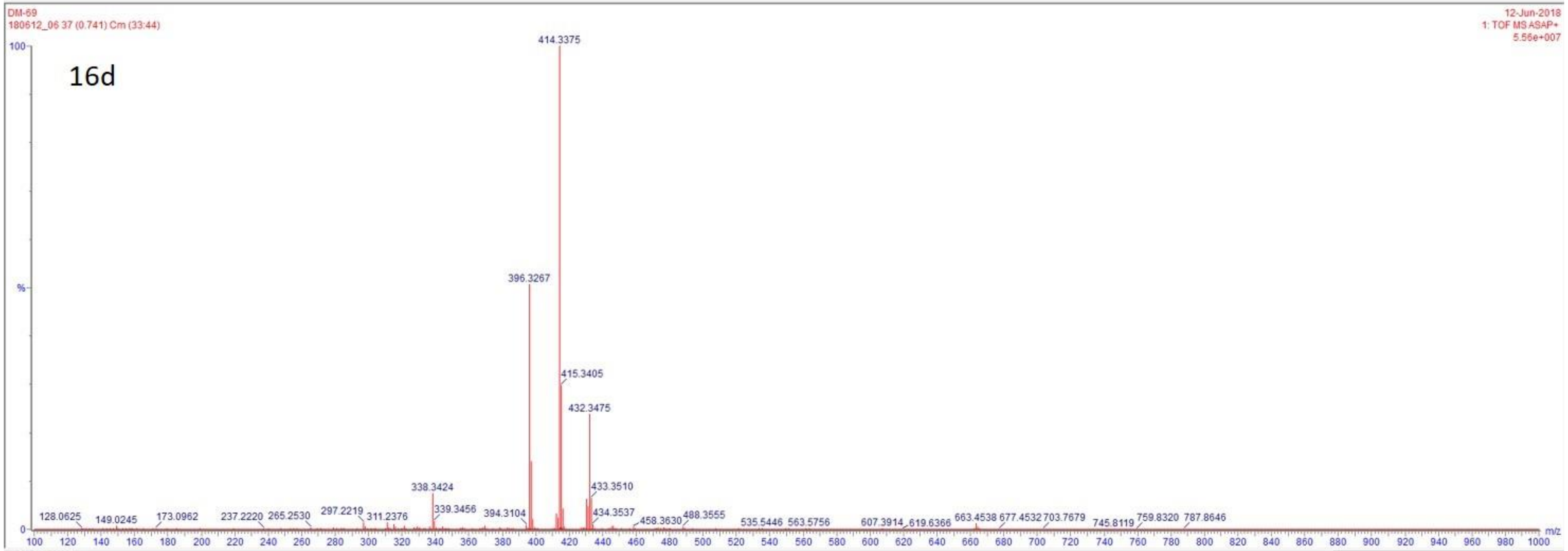

For Help, press F1

16e

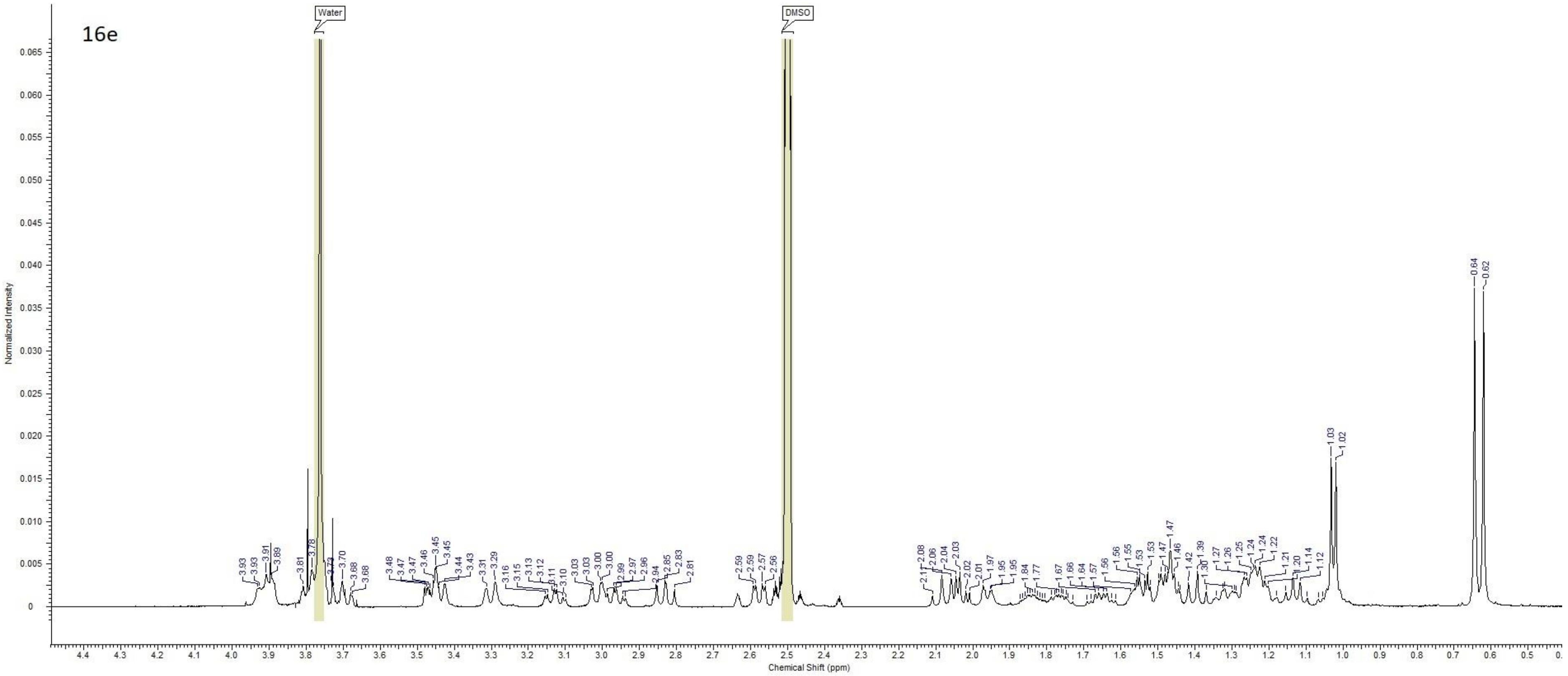

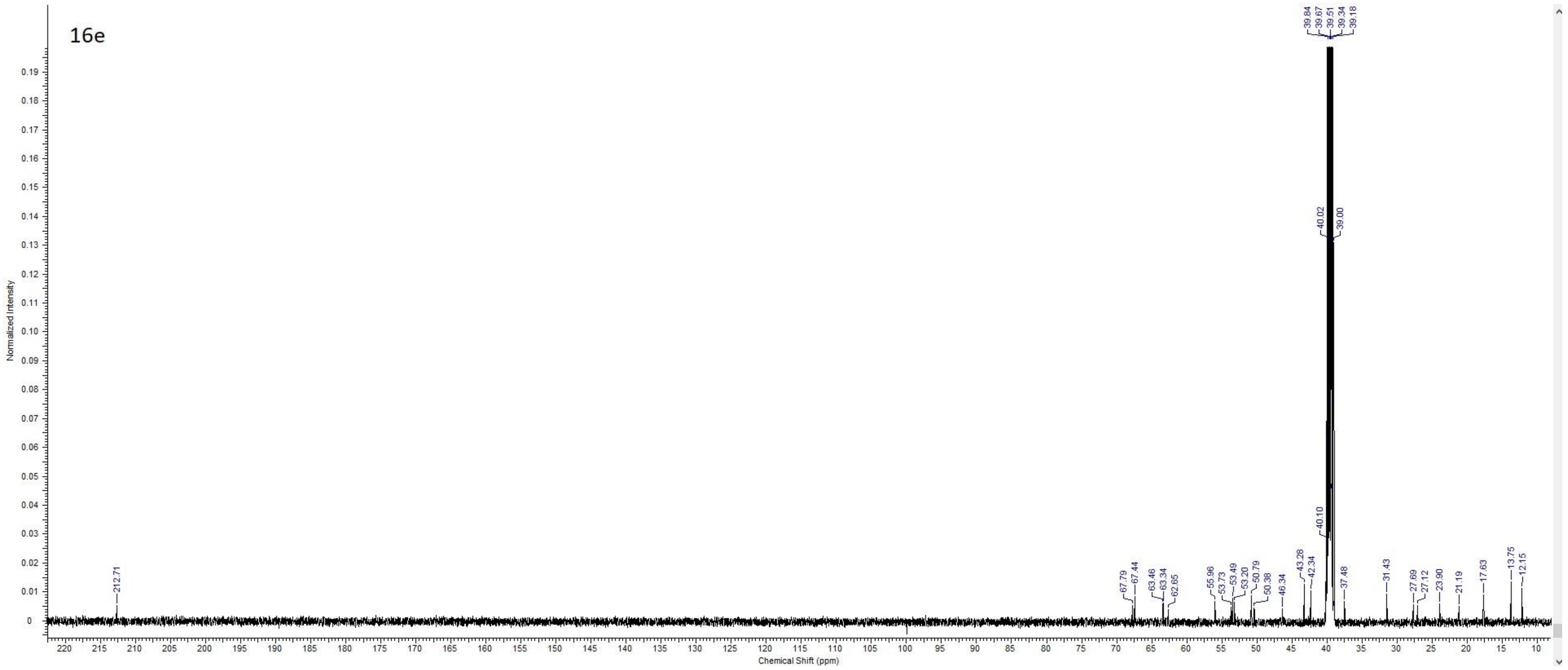
16e
Normalized Intensity
Chemical Shift (ppm)
212.71
67.79
67.44
63.46
63.34
62.65
55.96
53.73
53.49
53.20
50.79
50.38
46.34
43.28
42.34
40.10
40.02
39.84
39.67
39.51
39.34
39.18
39.00
37.48
31.43
27.69
27.12
23.90
21.19
17.63
13.75
12.15

Elemental Composition

File Edit View Process Help

Single Mass Analysis

Tolerance = 1.0 PPM / DBE: min = -1.5, max = 50.0

Element prediction: Off

Number of isotope peaks used for i-FIT = 3

Monoisotopic Mass, Even Electron Ions

569 formula(e) evaluated with 1 results within limits (up to 50 best isotopic matches for each mass)

Elements Used:

Mass	Calc. Mass	mDa	PPM	DBE	Formula	i-FIT	i-FIT Norm	Fit Conf %	C	H	N	O
434.3272	434.3270	0.2	0.5	5.5	C26 H44 N O4	938.1	n/a	n/a	26	44	1	4

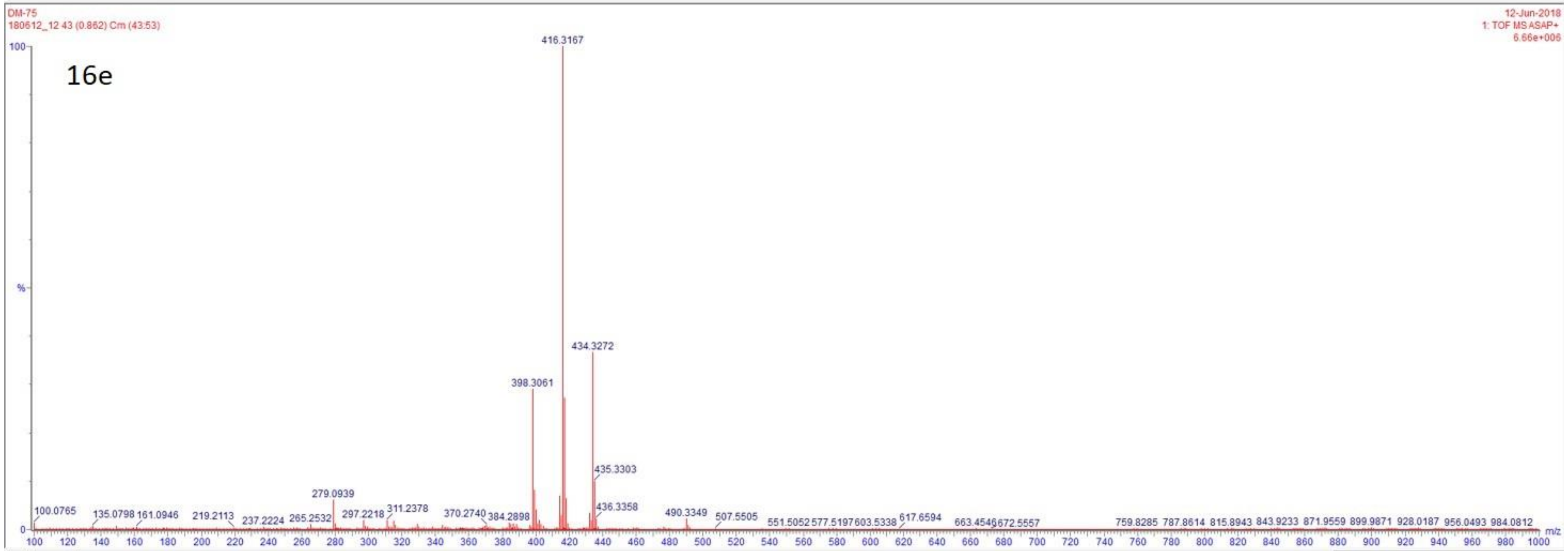

For Help, press F1

18

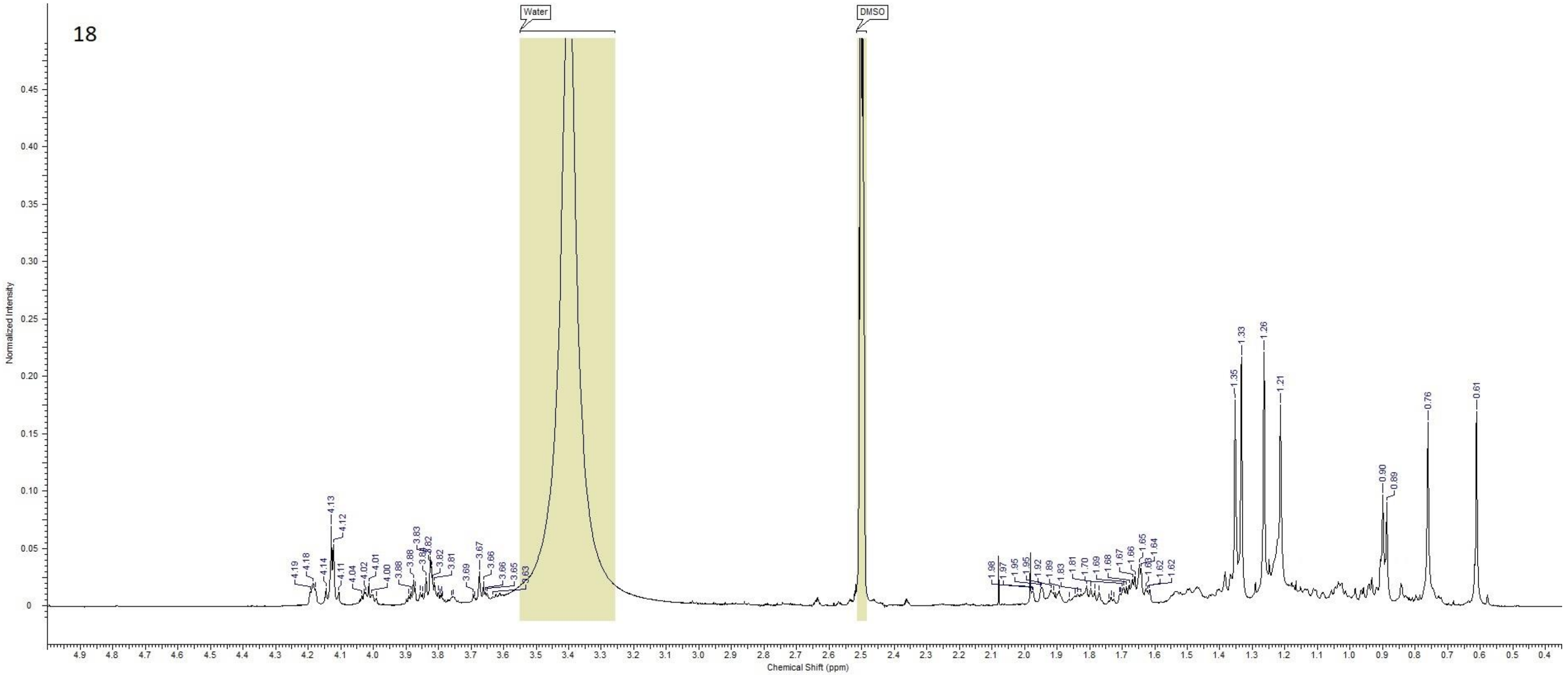

Water
DMSO
Normalized Intensity
Chemical Shift (ppm)
4.19
4.18
4.14
4.13
4.12
4.11
4.04
4.02
4.01
4.00
3.88
3.88
3.83
3.82
3.82
3.81
3.69
3.67
3.66
3.66
3.65
3.63
1.98
1.97
1.95
1.95
1.92
1.89
1.83
1.81
1.70
1.69
1.68
1.67
1.66
1.65
1.64
1.62
1.62
1.35
1.33
1.26
1.21
0.90
0.89
0.76
0.61

Elemental Composition

File Edit View Process Help

Single Mass Analysis

Tolerance = 10.0 PPM / DBE: min = -1.5, max = 50.0

Element prediction: Off

Number of isotope peaks used for i-FIT = 3

Monoisotopic Mass, Odd and Even Electron Ions

73 formula(e) evaluated with 1 results within limits (up to 50 best isotopic matches for each mass)

Elements Used:

Mass	Calc. Mass	mDa	PPM	DBE	Formula	i-FIT	i-FIT Norm	Fit Conf %	C	H	O
561.3426	561.3427	-0.1	-0.2	8.5	C32 H49 O8	481.7	n/a	n/a	32	49	8

18

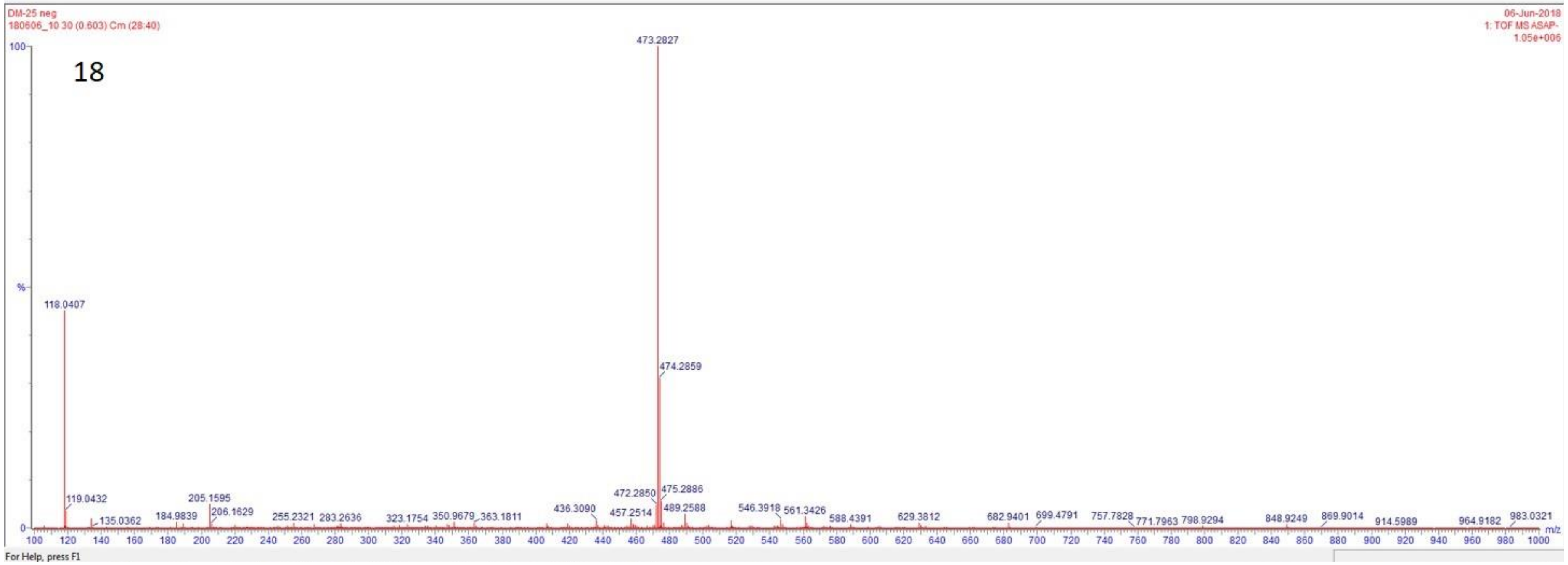

For Help, press F1

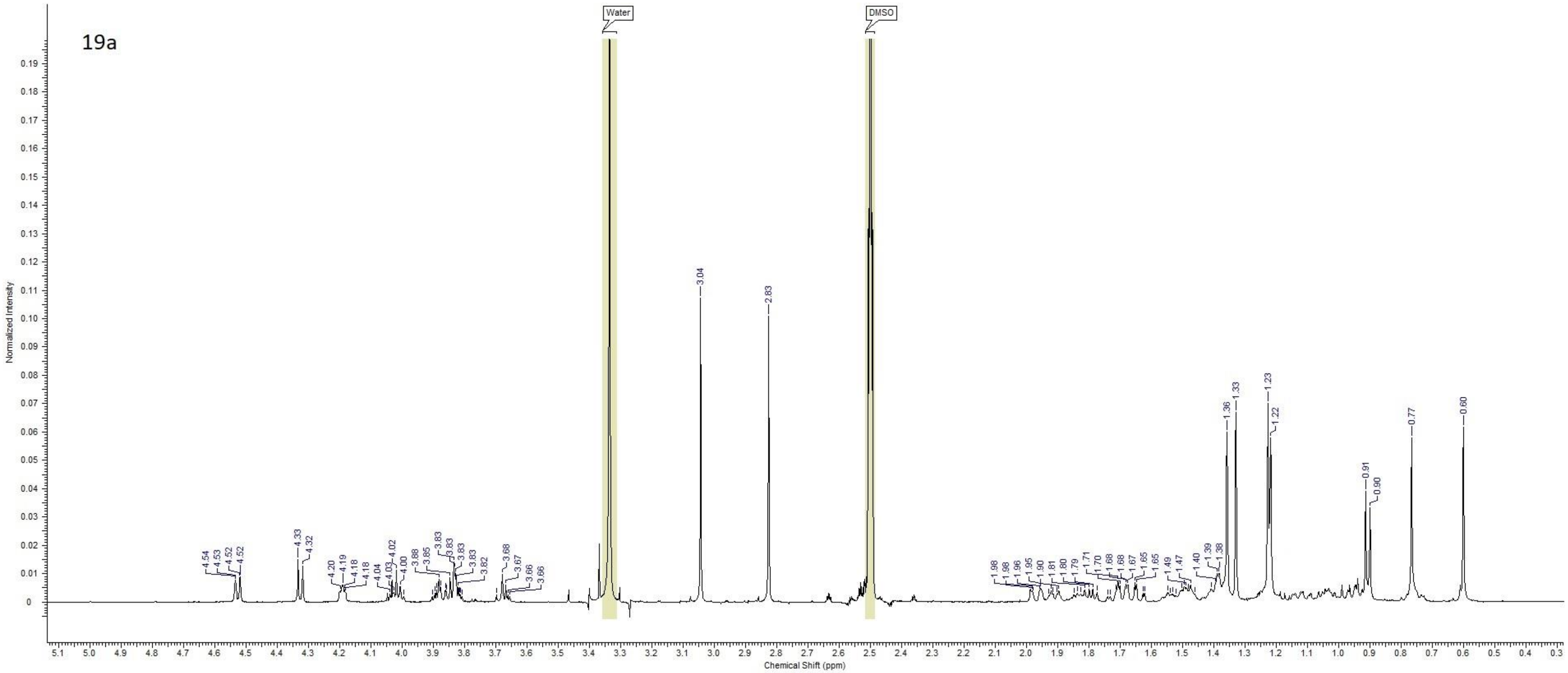
19a
Water
DMSO
Normalized Intensity
Chemical Shift (ppm)
4.54 4.53 4.52 4.52
4.33 4.32
4.20 4.19 4.18 4.18 4.04 4.03 4.02 4.00
3.88 3.85 3.83 3.83 3.83 3.83 3.82
3.68 3.67 3.66 3.66
3.04
2.83
1.98 1.98 1.96 1.95 1.90 1.81 1.80 1.79 1.71 1.70 1.68 1.68 1.67 1.65 1.65 1.49 1.47 1.40 1.39 1.38
1.36 1.33
1.23 1.22
0.91 0.90
0.77
0.60

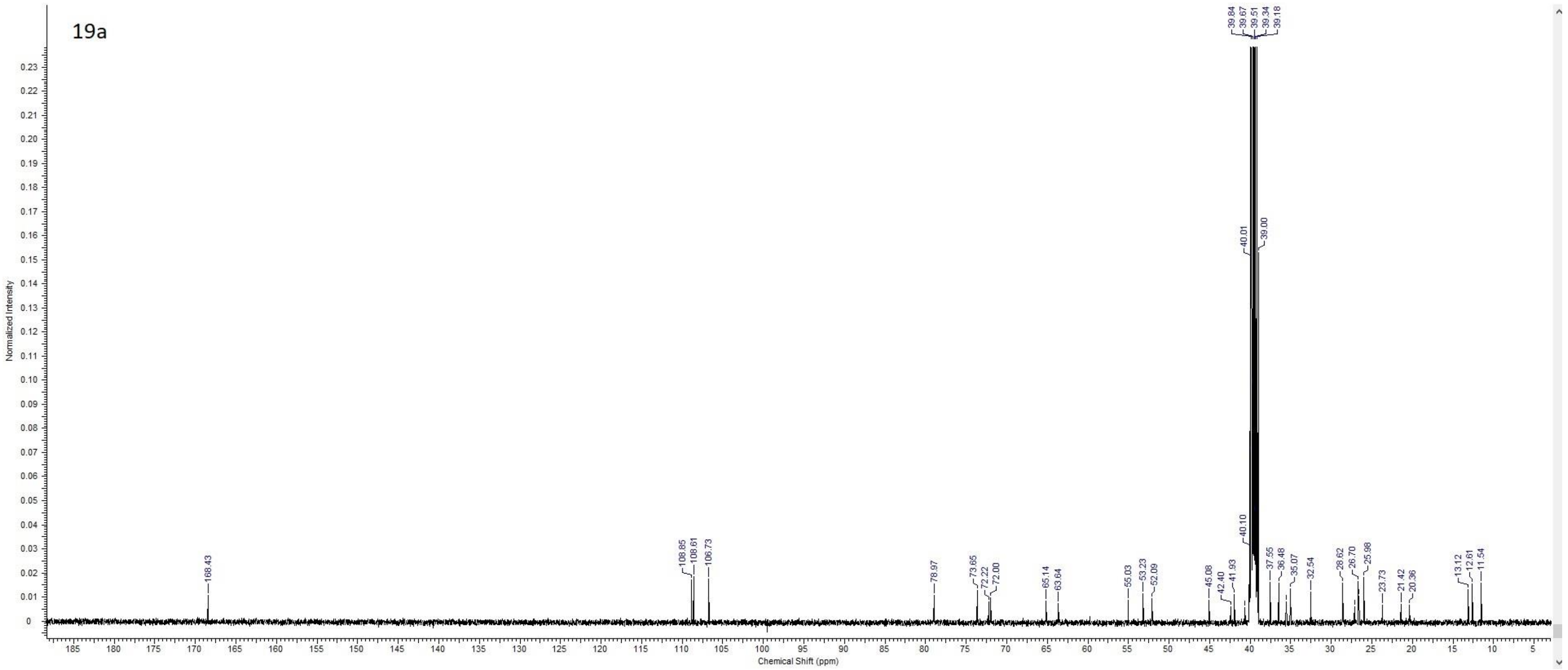
19a
Normalized Intensity
Chemical Shift (ppm)
168.43
108.85
108.61
106.73
78.97
73.65
72.22
72.00
65.14
63.64
55.03
53.23
52.09
45.08
42.40
41.93
40.10
40.01
39.84
39.67
39.51
39.34
39.18
39.00
37.55
36.48
35.07
32.54
28.62
26.70
25.98
23.73
21.42
20.36
13.12
12.61
11.54

Elemental Composition

File Edit View Process Help

Single Mass Analysis
Tolerance = 1.1 PPM / DBE: min = -1.5, max = 50.0
Element prediction: Off
Number of isotope peaks used for i-FIT = 3
Monoisotopic Mass, Even Electron Ions
959 formula(e) evaluated with 1 results within limits (up to 50 best isotopic matches for each mass)
Elements Used:

Mass	Calc. Mass	mDa	PPM	DBE	Formula	i-FIT	i-FIT Norm	Fit Conf %	C	H	N	O
590.4061	590.4057	0.4	0.7	7.5	C34 H56 N O7	35.4	n/a	n/a	34	56	1	7

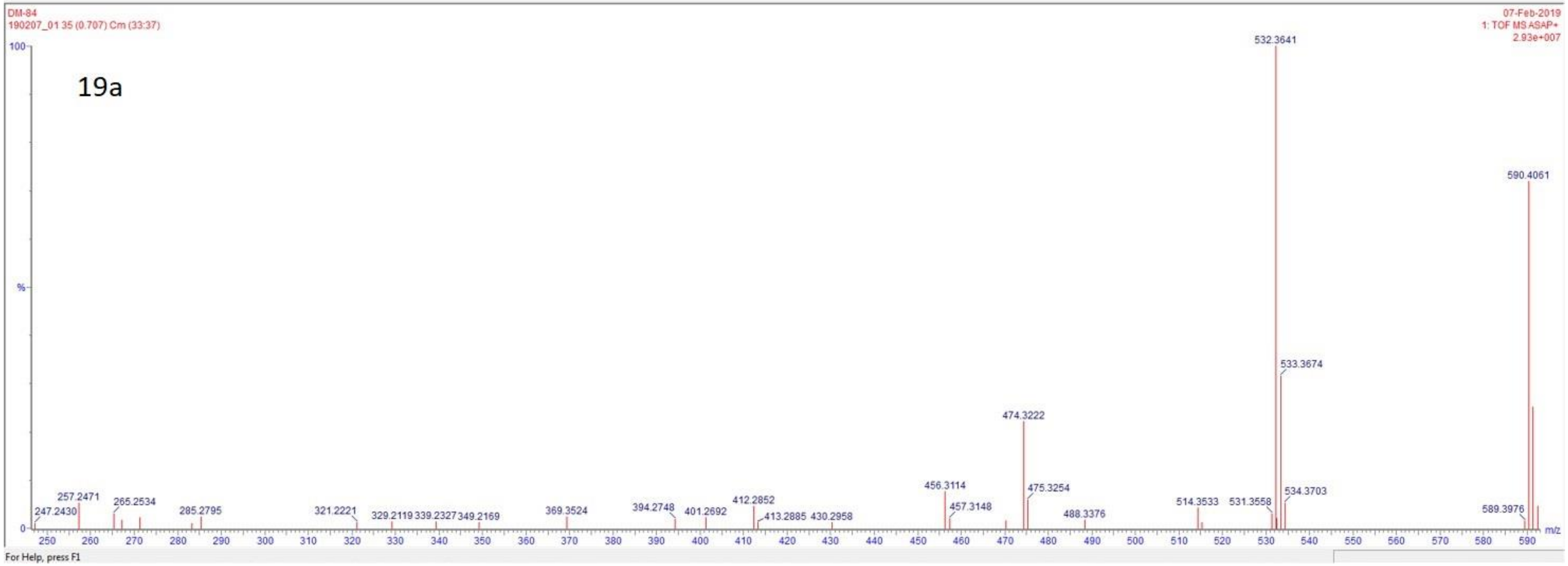

For Help, press F1

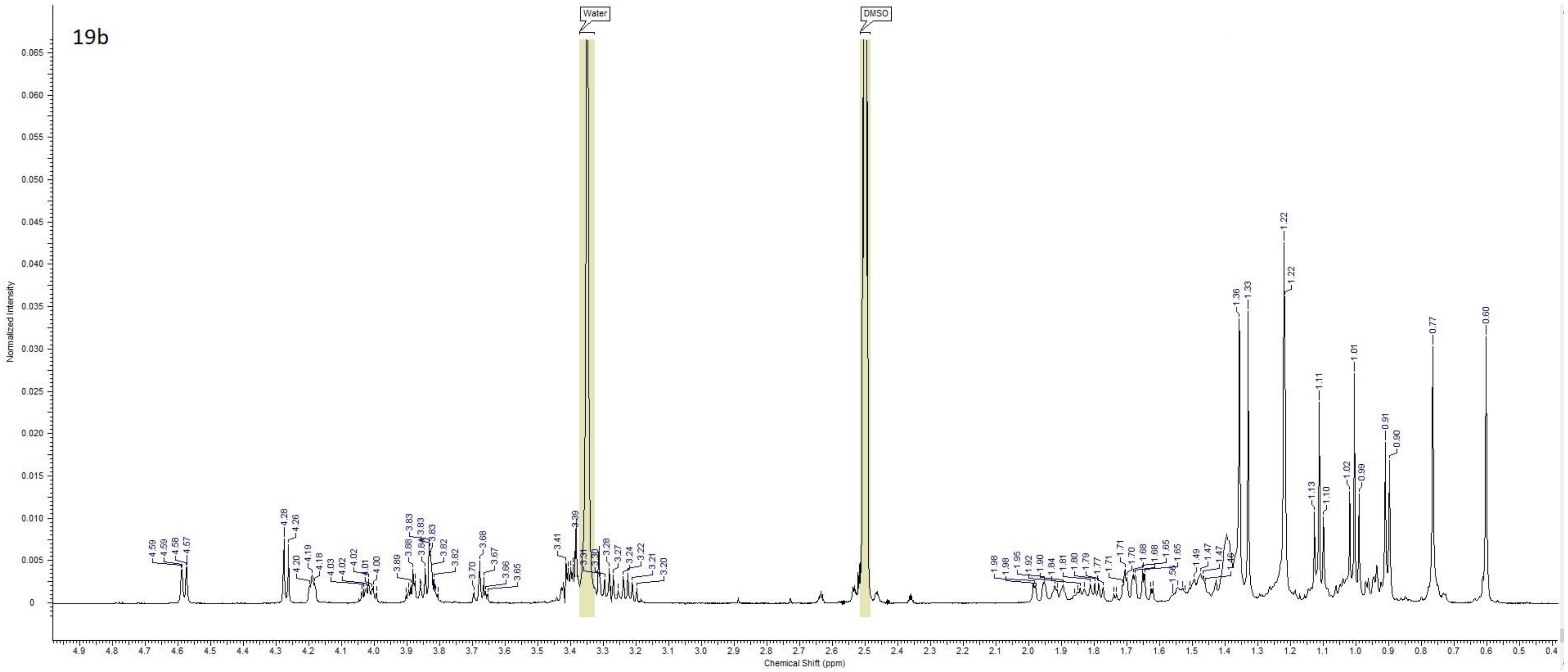
19b
Water
DMSO
Normalized Intensity
Chemical Shift (ppm)

19b

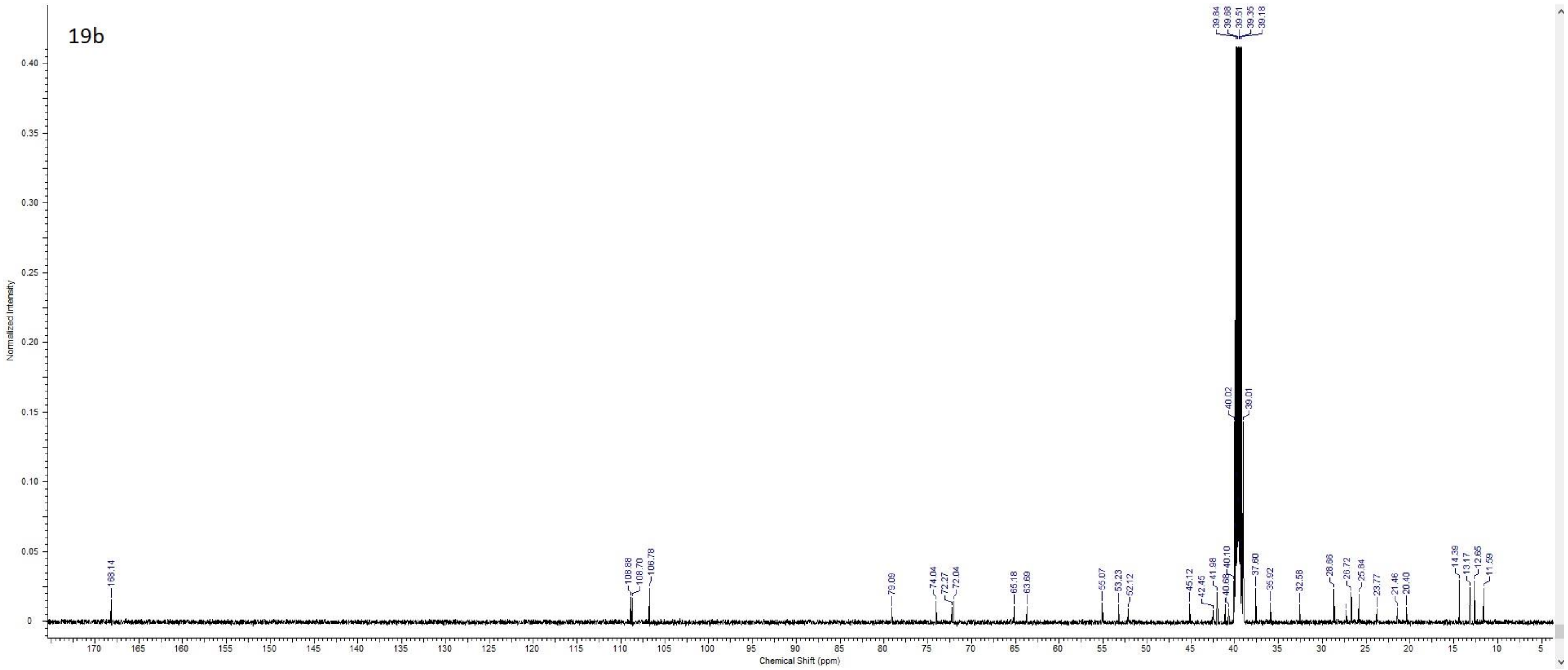

168.14
108.88
108.70
106.78
79.09
74.04
72.27
72.04
65.18
63.69
55.07
53.23
52.12
45.12
42.45
41.98
40.68
40.10
40.02
39.84
39.68
39.51
39.35
39.18
39.01
37.60
35.92
32.58
28.66
26.72
25.84
23.77
21.46
20.40
14.39
13.17
12.65
11.59
Normalized Intensity
Chemical Shift (ppm)

Elemental Composition

File Edit View Process Help

Single Mass Analysis

Tolerance = 1.0 PPM / DBE: min = -1.5, max = 50.0

Element prediction: Off

Number of isotope peaks used for i-FIT = 3

Monoisotopic Mass, Even Electron Ions

1049 formula(e) evaluated with 1 results within limits (up to 50 best isotopic matches for each mass)

Elements Used:

Mass	Calc. Mass	mDa	PPM	DBE	Formula	i-FIT	i-FIT Norm	Fit Conf %	C	H	N	O
618.4375	618.4370	0.5	0.8	7.5	C36 H60 N O7	35.1	n/a	n/a	36	60	1	7

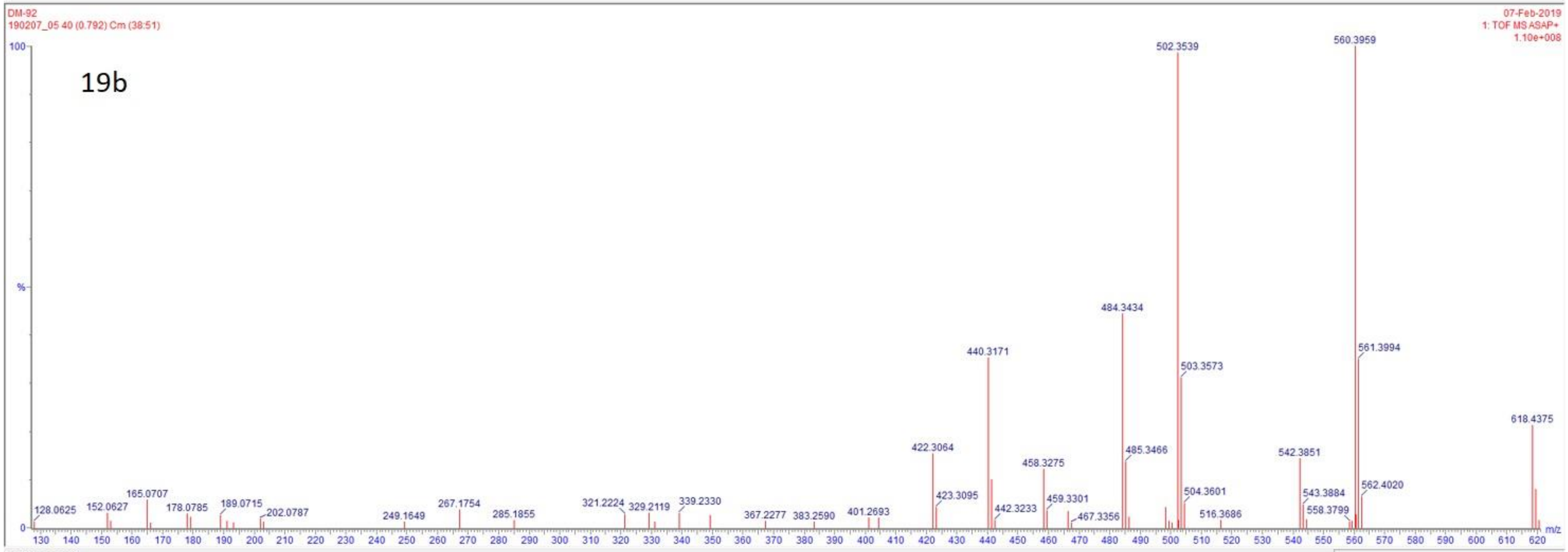

For Help, press F1

19c

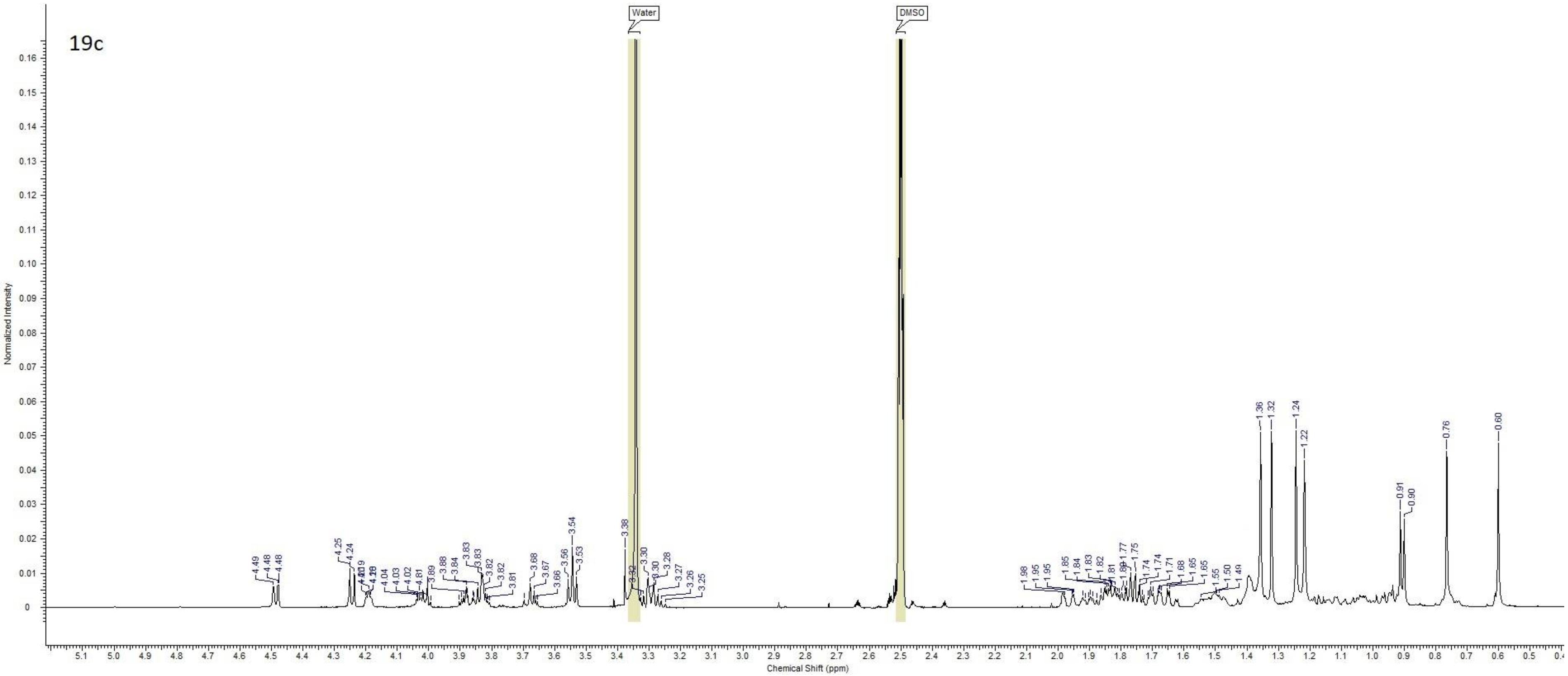

19c

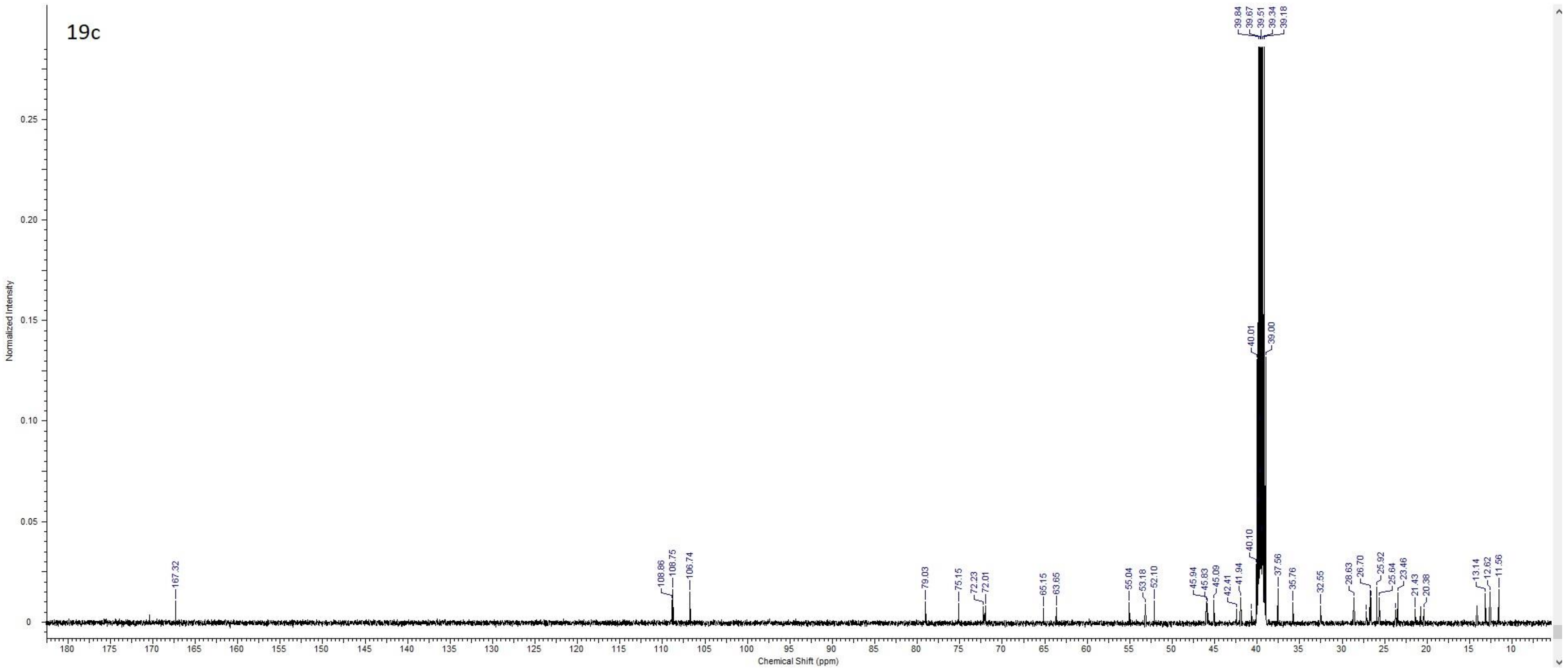

Normalized Intensity
Chemical Shift (ppm)
167.32
108.86
108.75
106.74
79.03
75.15
72.23
72.01
65.15
63.65
55.04
53.18
52.10
45.94
45.83
45.09
42.41
41.94
40.10
40.01
39.84
39.67
39.51
39.34
39.18
39.00
37.56
35.76
32.55
28.63
26.70
25.92
25.64
23.46
21.43
20.38
13.14
12.62
11.56

Elemental Composition

File Edit View Process Help

Single Mass Analysis

Tolerance = 1.0 PPM / DBE: min = -1.5, max = 50.0

Element prediction: Off

Number of isotope peaks used for i-FIT = 3

Monoisotopic Mass, Even Electron Ions

1039 formula(e) evaluated with 1 results within limits (up to 50 best isotopic matches for each mass)

Elements Used:

Mass	Calc. Mass	mDa	PPM	DBE	Formula	i-FIT	i-FIT Norm	Fit Conf %	C	H	N	O
616.4218	616.4213	0.5	0.8	8.5	C36 H58 N O7	35.3	n/a	n/a	36	58	1	7

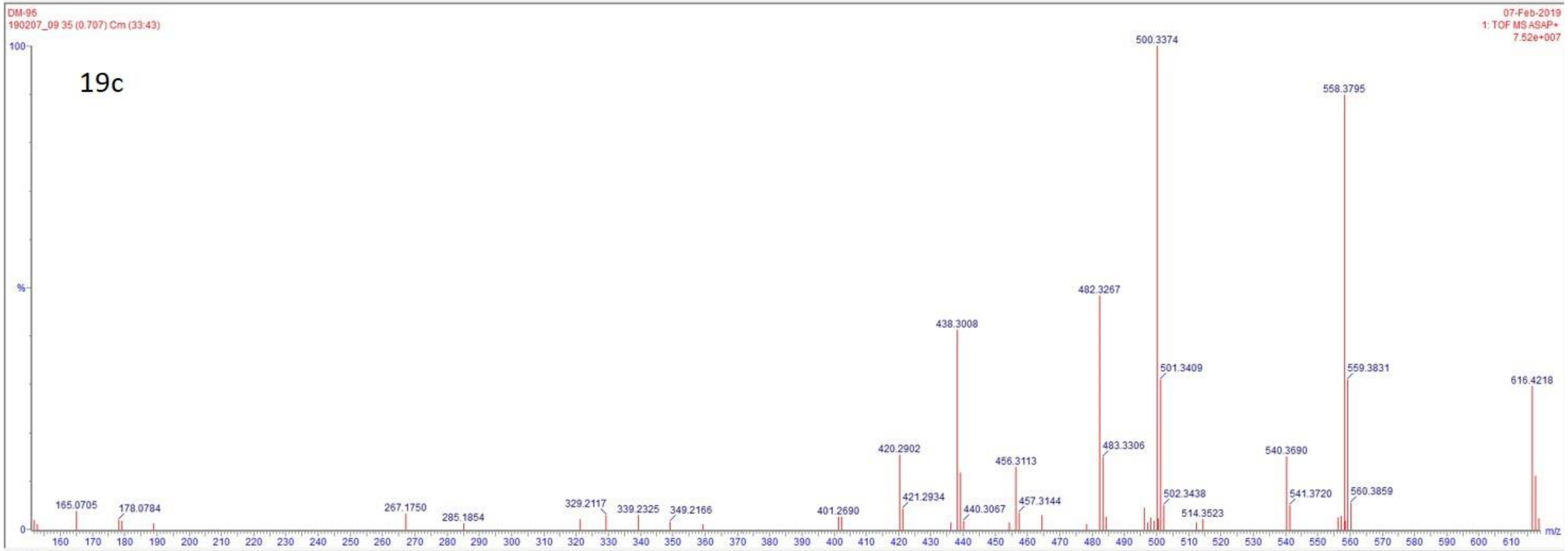

For Help, press F1

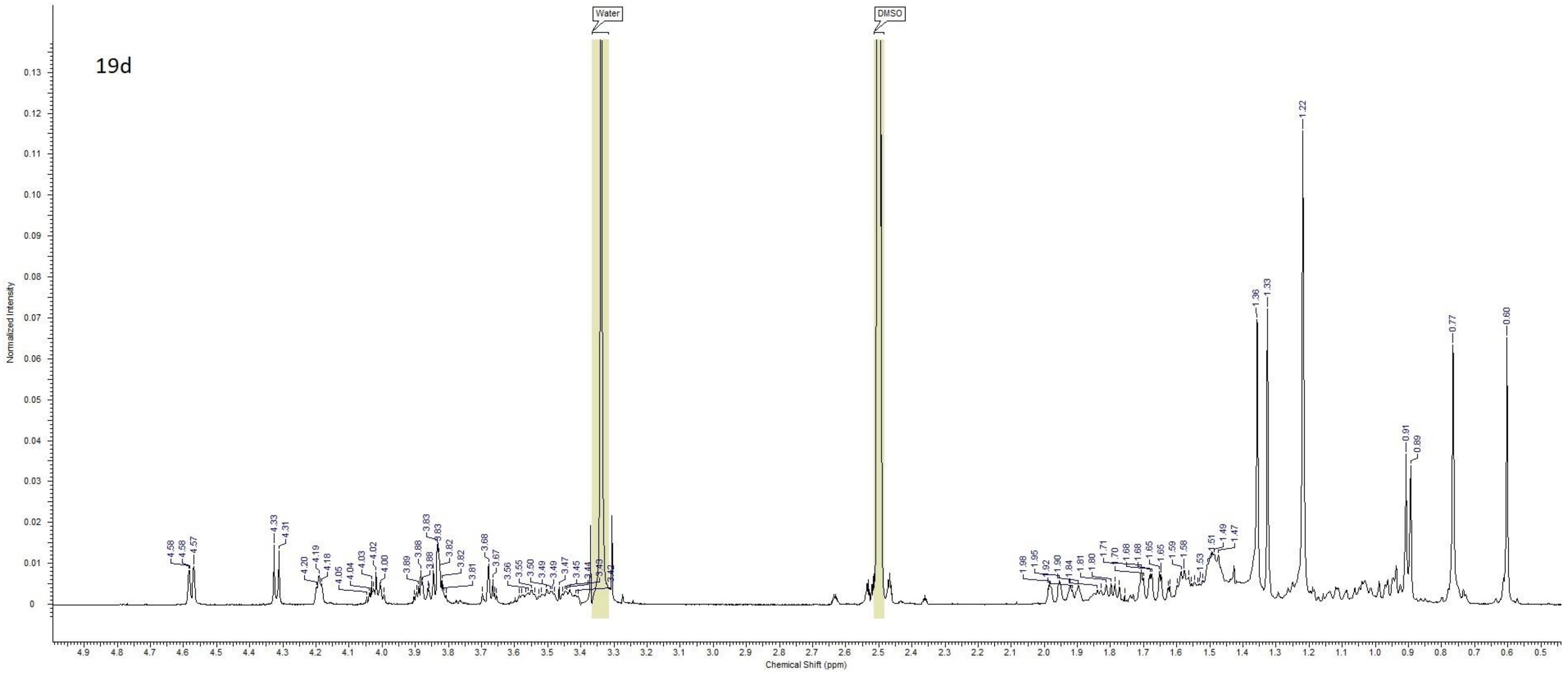
19d
Water
DMSO
Normalized Intensity
Chemical Shift (ppm)

19d

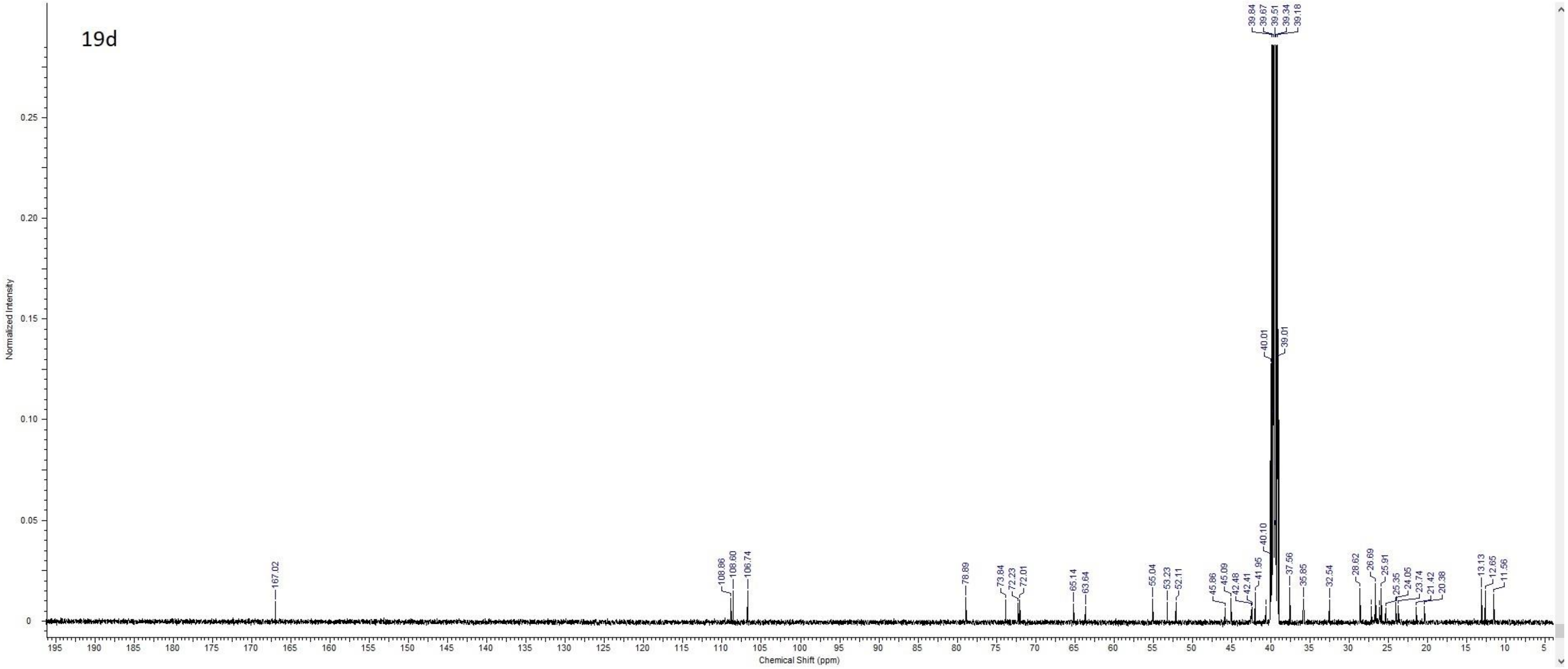

Normalized Intensity
Chemical Shift (ppm)
0
0.05
0.10
0.15
0.20
0.25
195 190 185 180 175 170 165 160 155 150 145 140 135 130 125 120 115 110 105 100 95 90 85 80 75 70 65 60 55 50 45 40 35 30 25 20 15 10 5
167.02
108.86
108.60
106.74
78.89
73.84
72.23
72.01
65.14
63.64
55.04
53.23
52.11
45.86
45.09
42.48
42.41
41.95
40.10
40.01
39.84
39.67
39.51
39.34
39.18
39.01
37.56
35.85
32.54
28.62
26.69
25.91
25.35
24.05
23.74
21.42
20.38
13.13
12.65
11.56

Elemental Composition

File Edit View Process Help

Single Mass Analysis
Tolerance = 1.0 PPM / DBE: min = -1.5, max = 50.0
Element prediction: Off
Number of isotope peaks used for i-FIT = 3
Monoisotopic Mass, Even Electron Ions
1089 formula(e) evaluated with 1 results within limits (up to 50 best isotopic matches for each mass)
Elements Used:

Mass	Calc. Mass	mDa	PPM	DBE	Formula	i-FIT	i-FIT Norm	Fit Conf %	C	H	N	O
630.4370	630.4370	0.0	0.0	8.5	C37 H60 N O7	62.2	n/a	n/a	37	60	1	7

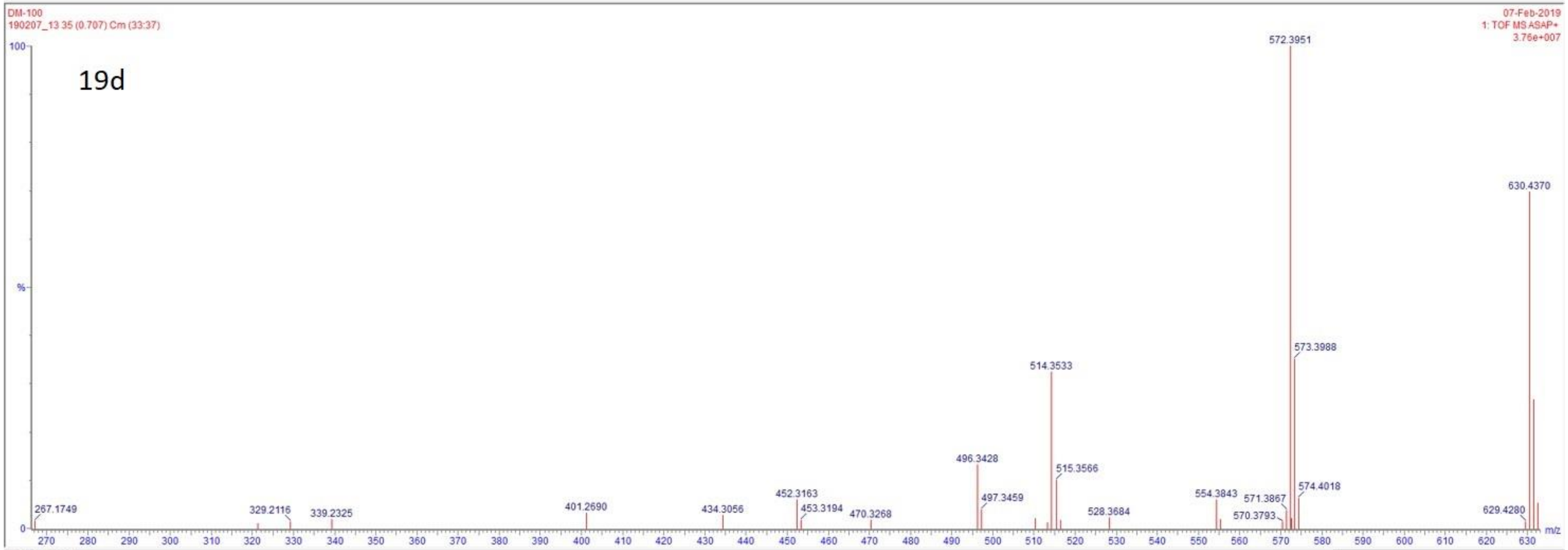

For Help, press F1

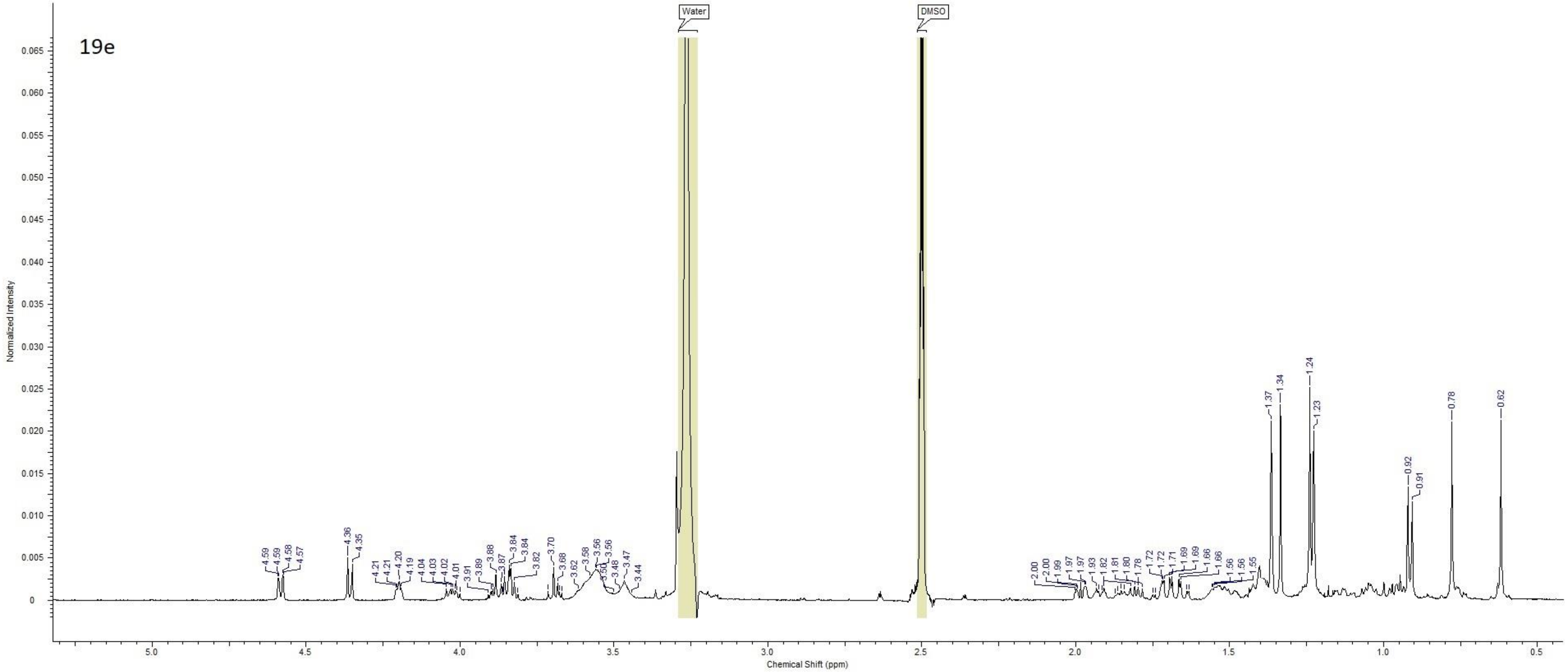

19e
Water
DMSO
Normalized Intensity
Chemical Shift (ppm)
4.59
4.59
4.58
4.57
4.36
4.35
4.21
4.21
4.20
4.19
4.04
4.03
4.02
4.01
3.91
3.89
3.88
3.87
3.84
3.84
3.82
3.70
3.68
3.62
3.58
3.56
3.56
3.50
3.48
3.47
3.44
2.00
2.00
1.99
1.97
1.97
1.93
1.82
1.81
1.80
1.78
1.72
1.72
1.71
1.69
1.69
1.66
1.66
1.56
1.56
1.55
1.37
1.34
1.24
1.23
0.92
0.91
0.78
0.62

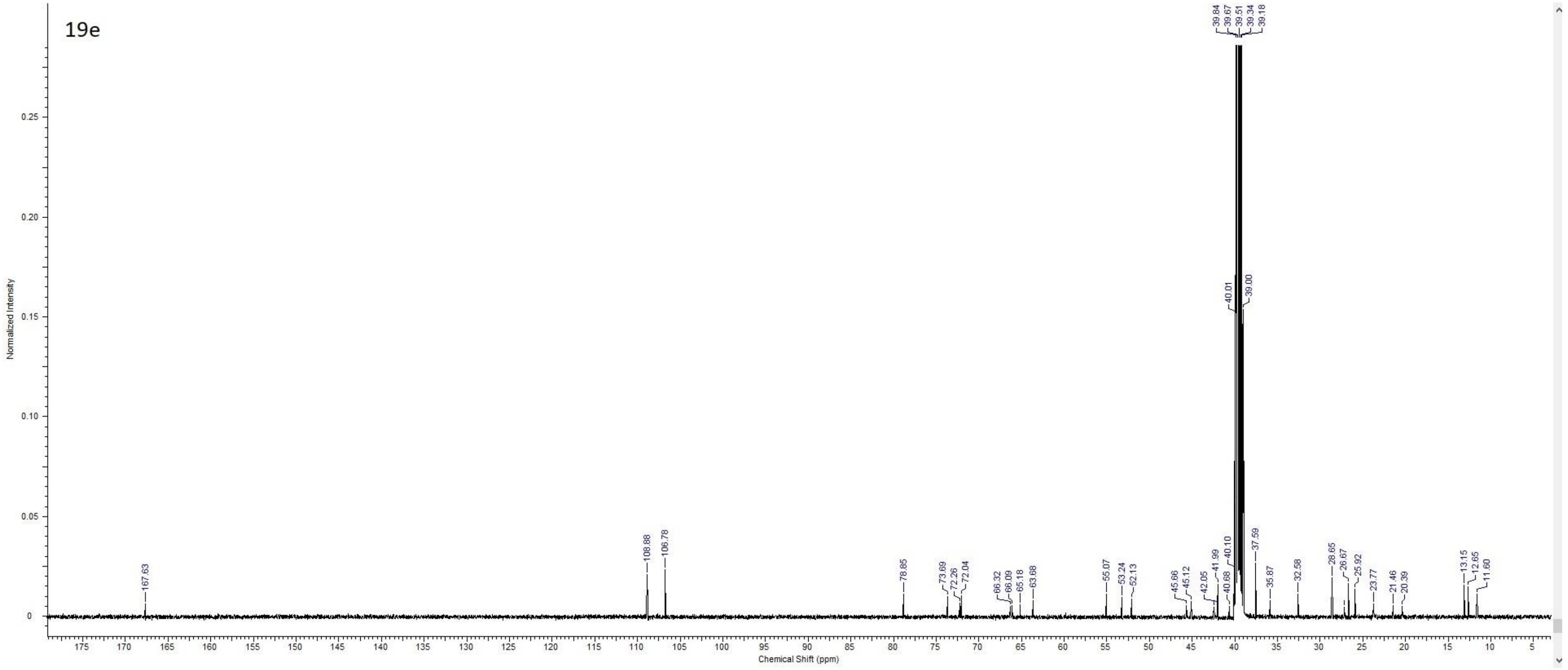

19e
Normalized Intensity
Chemical Shift (ppm)
167.63
108.88
106.78
78.85
73.69
72.26
72.04
66.32
66.09
65.18
63.68
55.07
53.24
52.13
45.66
45.12
42.05
41.99
40.68
40.10
40.01
39.84
39.67
39.51
39.34
39.18
39.00
37.59
35.87
32.58
28.65
26.67
25.92
23.77
21.46
20.39
13.15
12.65
11.60

Elemental Composition

File Edit View Process Help

Single Mass Analysis

Tolerance = 1.0 PPM / DBE: min = -1.5, max = 50.0

Element prediction: Off

Number of isotope peaks used for i-FIT = 3

Monoisotopic Mass, Even Electron Ions

855 formula(e) evaluated with 1 results within limits (up to 50 best isotopic matches for each mass)

Elements Used:

Mass	Calc. Mass	mDa	PPM	DBE	Formula	i-FIT	i-FIT Norm	Fit Conf %	C	H	N	O
632.4168	632.4162	0.6	0.9	8.5	C36 H58 N O8	1257.0	n/a	n/a	36	58	1	8

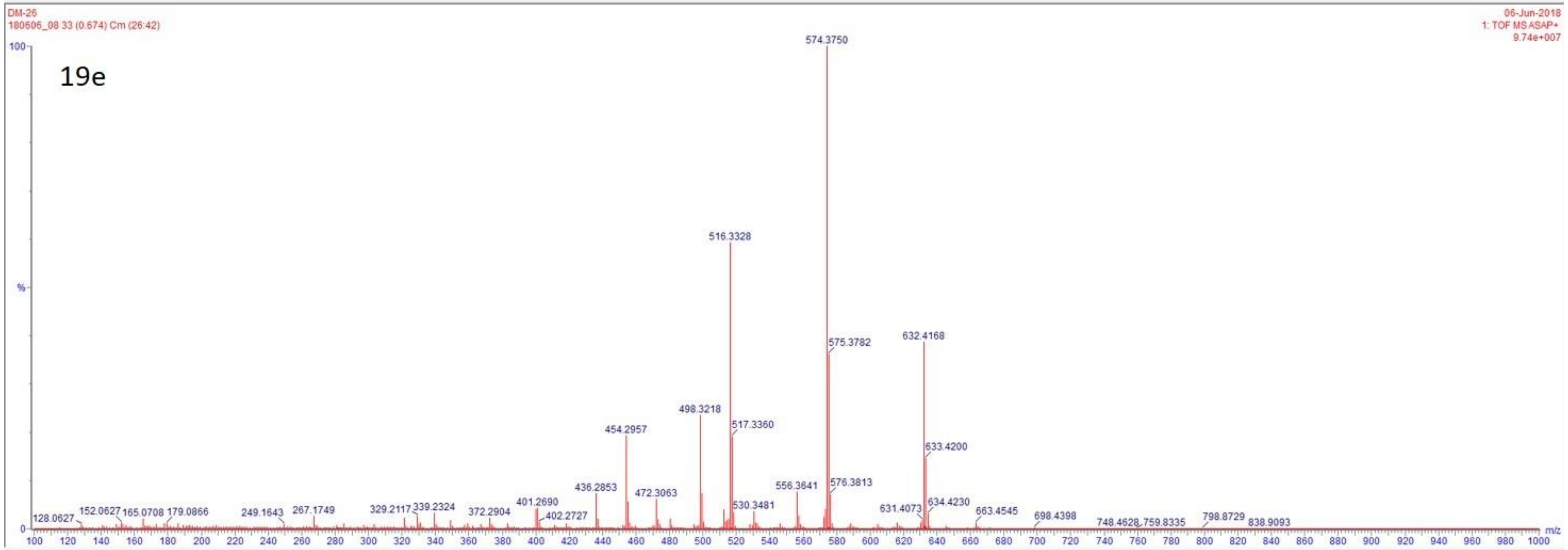

For Help, press F1

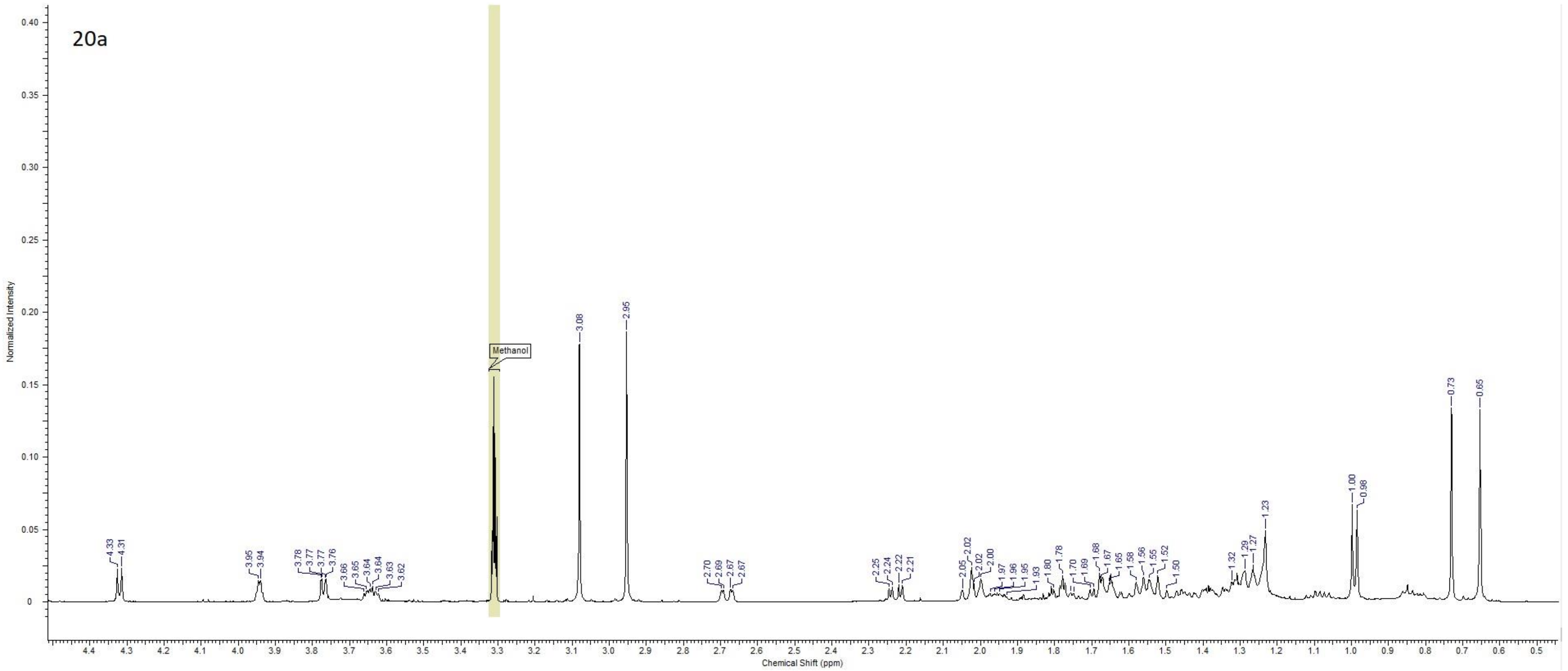
20a
Methanol
Normalized Intensity
Chemical Shift (ppm)
4.33
4.31
3.95
3.94
3.78
3.77
3.77
3.76
3.66
3.65
3.64
3.64
3.63
3.62
3.08
2.95
2.70
2.69
2.67
2.67
2.25
2.24
2.22
2.21
2.05
2.02
2.02
2.00
1.97
1.96
1.95
1.93
1.80
1.78
1.70
1.69
1.68
1.67
1.65
1.58
1.56
1.55
1.52
1.50
1.32
1.29
1.27
1.23
1.00
0.98
0.73
0.65

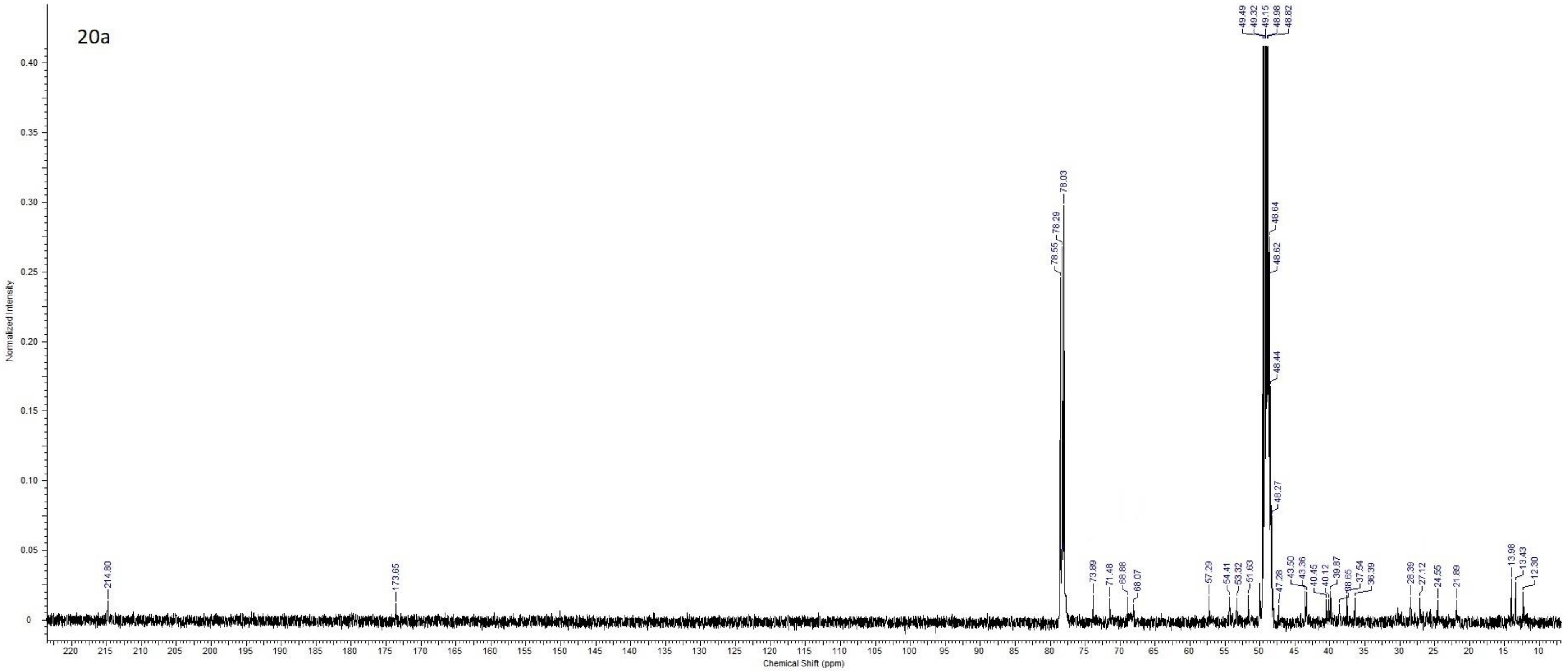
20a
Normalized Intensity
Chemical Shift (ppm)
214.80
173.65
78.55
78.29
78.03
73.89
71.48
68.88
68.07
57.29
54.41
53.32
51.63
49.49
49.32
49.15
48.98
48.82
48.64
48.62
48.44
48.27
47.28
43.50
43.36
40.45
40.12
39.87
38.65
37.54
36.39
28.39
27.12
24.55
21.89
13.98
13.43
12.30

20a

Qualitative Compound Report

**Data File**	201119_DM-87_02.d	**Sample Name**	DM-87
**Sample Type**	Sample	**Position**	P1-B2
**Instrument Name**	G6230B TOF	**User Name**	
**Acq Method**	HRMS_Jakub_ref.m	**Acquired Time**	19-Nov-20 12:25:55 PM
**IRM Calibration Status**	Success	**DA Method**	test.m
**Comment**			
**Sample Group**		**Info.**	
**Stream Name**		**Acquisition SW Version**	6200 series TOF/6500 series Q-TOF B.09.00 (B9044.1 SP1)

Compound Table

Compound Label	RT	Mass	Abund	Formula	Tgt Mass	Diff (ppm)
Cpd 1: C26 H43 N O6	4.402	465.3101	726270	C26 H43 N O6	465.309	2.27

Compound Label	m/z	RT	Algorithm	Mass
Cpd 1: C26 H43 N O6	466.3172	4.402	Find By Formula	465.3101

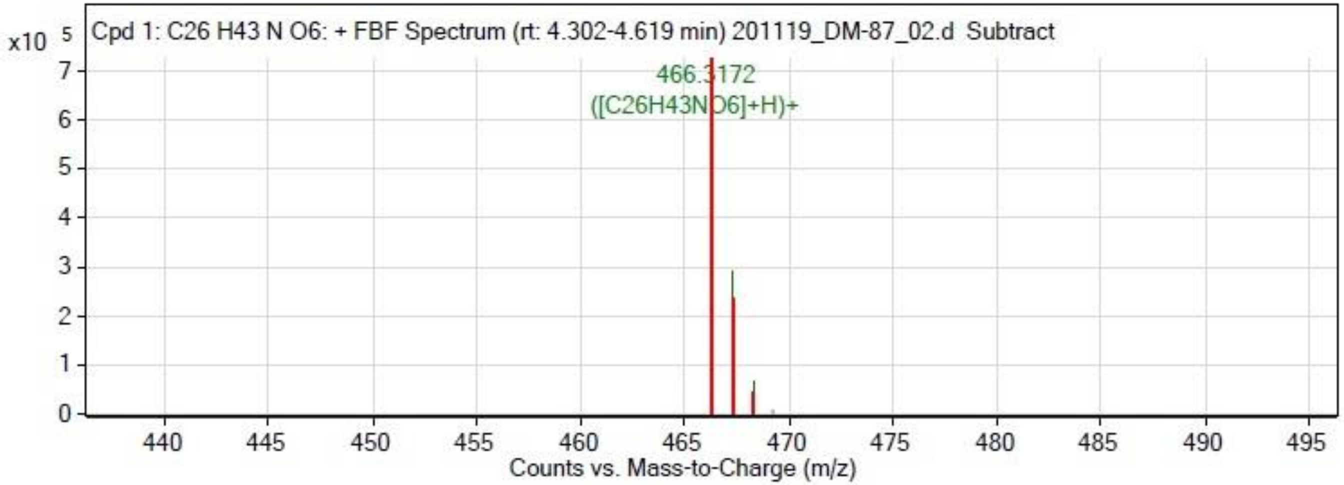

MS Spectrum Peak List

m/z	z	Abund	Formula	Ion
466.3172	1	726270	C26H43NO6	(M+H)+
467.3209	1	292254.84	C26H43NO6	(M+H)+
468.3247	1	67122.92	C26H43NO6	(M+H)+

--- End Of Report ---

20b

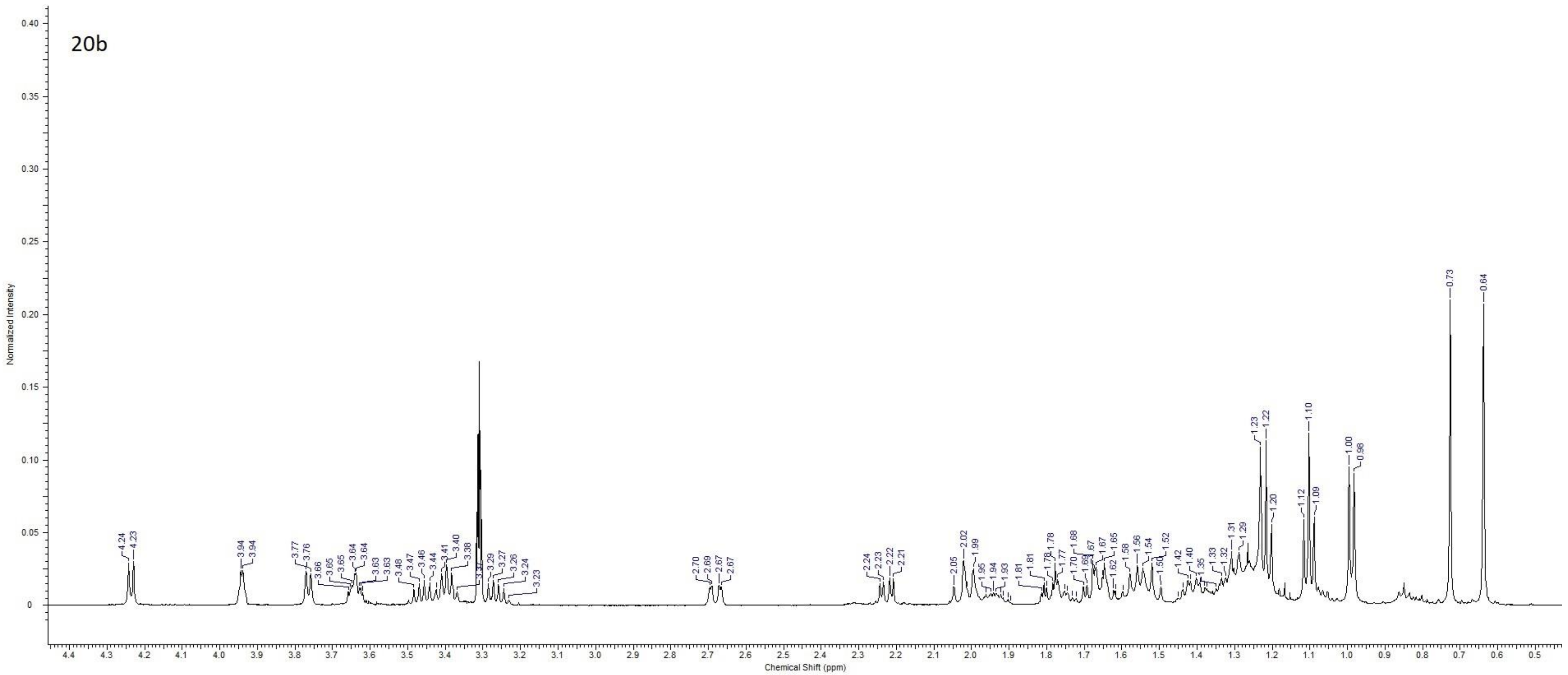
Normalized Intensity
Chemical Shift (ppm)
4.24
4.23
3.94
3.94
3.77
3.76
2.70
2.69
2.67
2.67
2.24
2.23
2.22
2.21
2.05
2.02
1.99
1.23
1.22
1.20
1.12
1.10
1.09
1.00
0.98
0.73
0.64

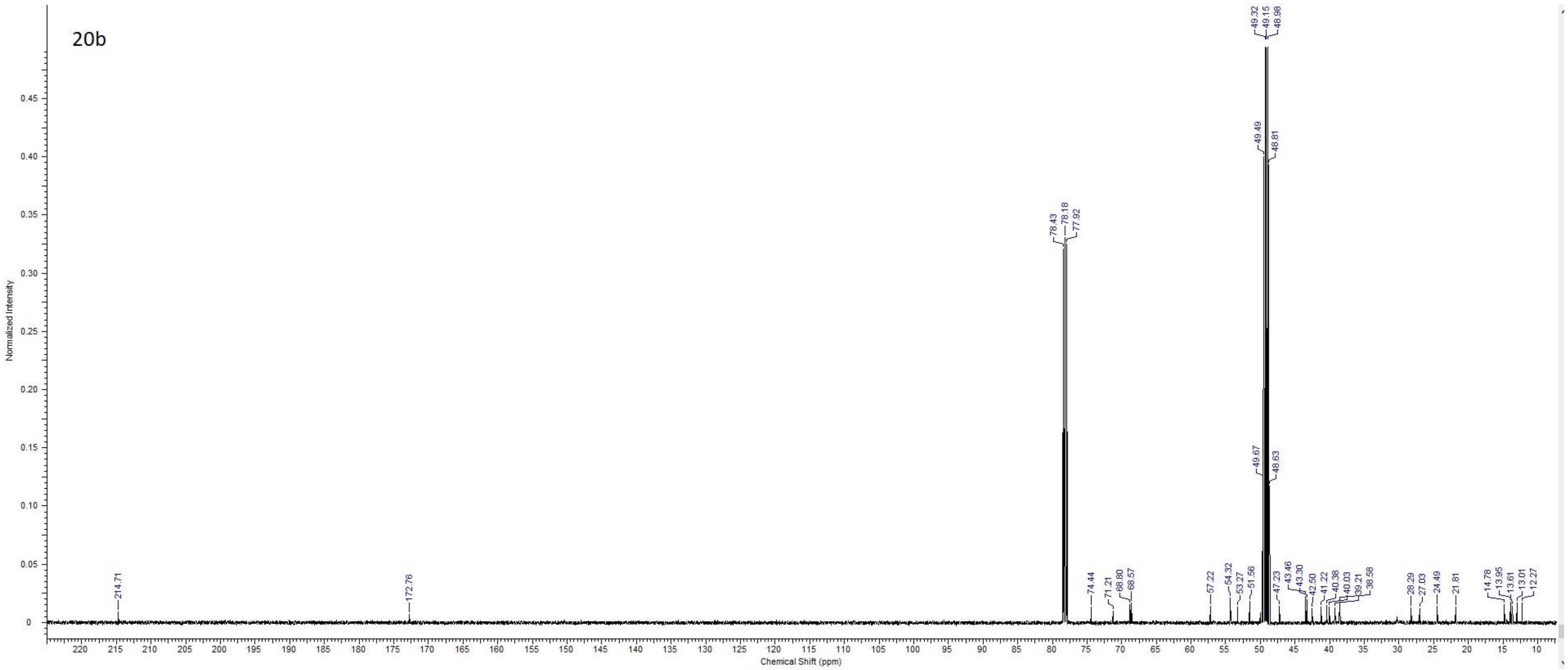
20b
Normalized Intensity
Chemical Shift (ppm)
214.71
172.76
78.43
78.18
77.92
74.44
71.21
68.80
68.57
57.22
54.32
53.27
51.56
49.67
49.49
49.32
49.15
48.98
48.81
48.63
47.23
43.46
43.30
42.50
41.22
40.38
40.03
39.21
38.58
28.29
27.03
24.49
21.81
14.78
13.95
13.61
13.01
12.27

Elemental Composition

File Edit View Process Help

Single Mass Analysis
Tolerance = 1.0 PPM / DBE: min = -1.5, max = 50.0
Element prediction: Off
Number of isotope peaks used for i-FIT = 3
Monoisotopic Mass, Even Electron Ions
682 formula(e) evaluated with 1 results within limits (up to 50 best isotopic matches for each mass)
Elements Used:

Mass	Calc. Mass	mDa	PPM	DBE	Formula	i-FIT	i-FIT Norm	Fit Conf %	C	H	N	O
494.3481	494.3482	-0.1	-0.2	5.5	C28 H48 N O6	29.9	n/a	n/a	28	48	1	6

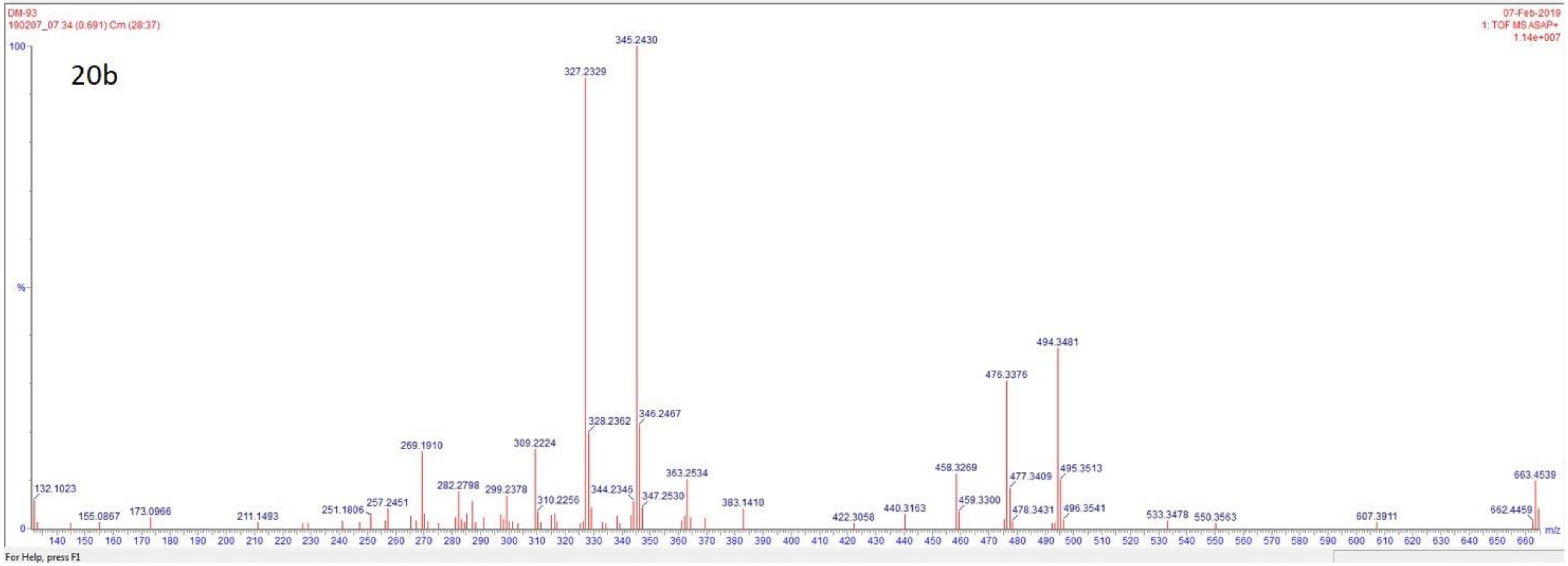

For Help, press F1

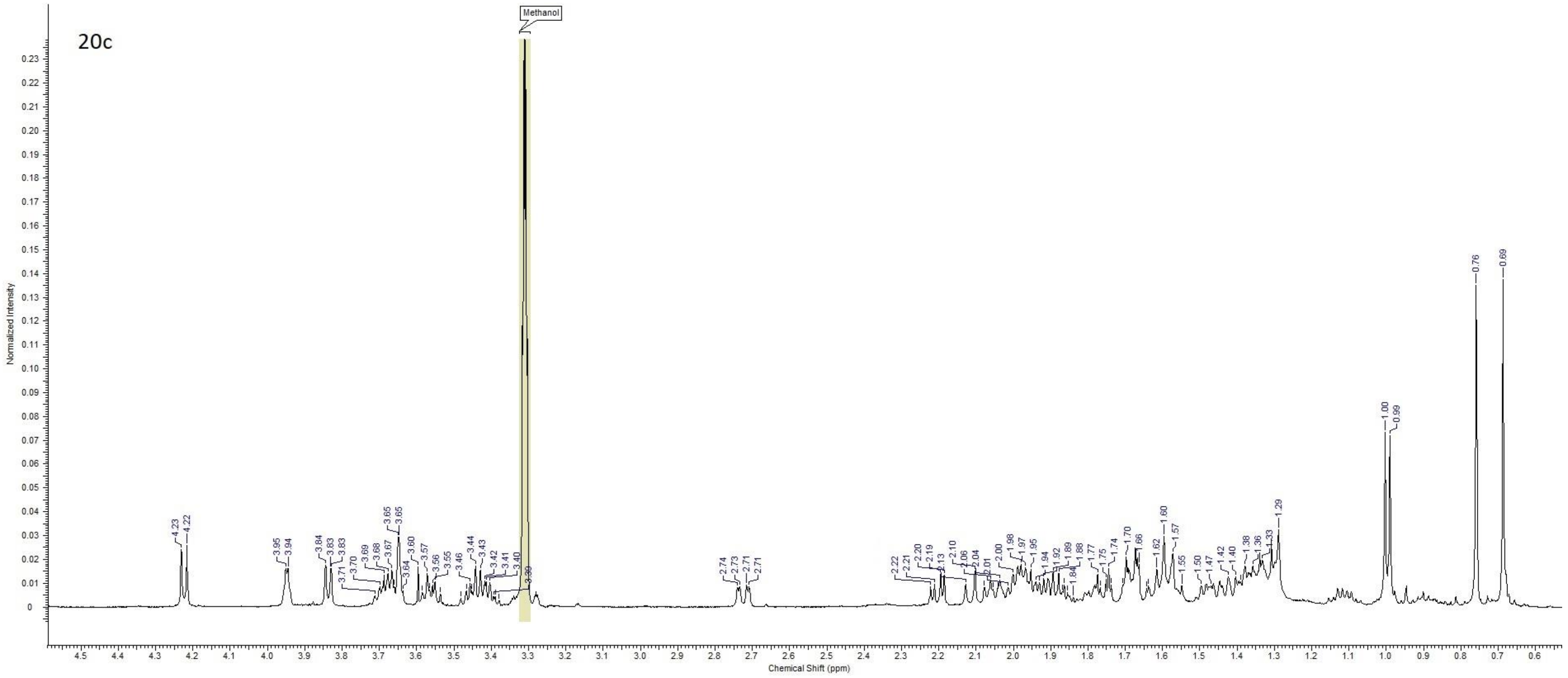
20c
Methanol
Normalized Intensity
Chemical Shift (ppm)

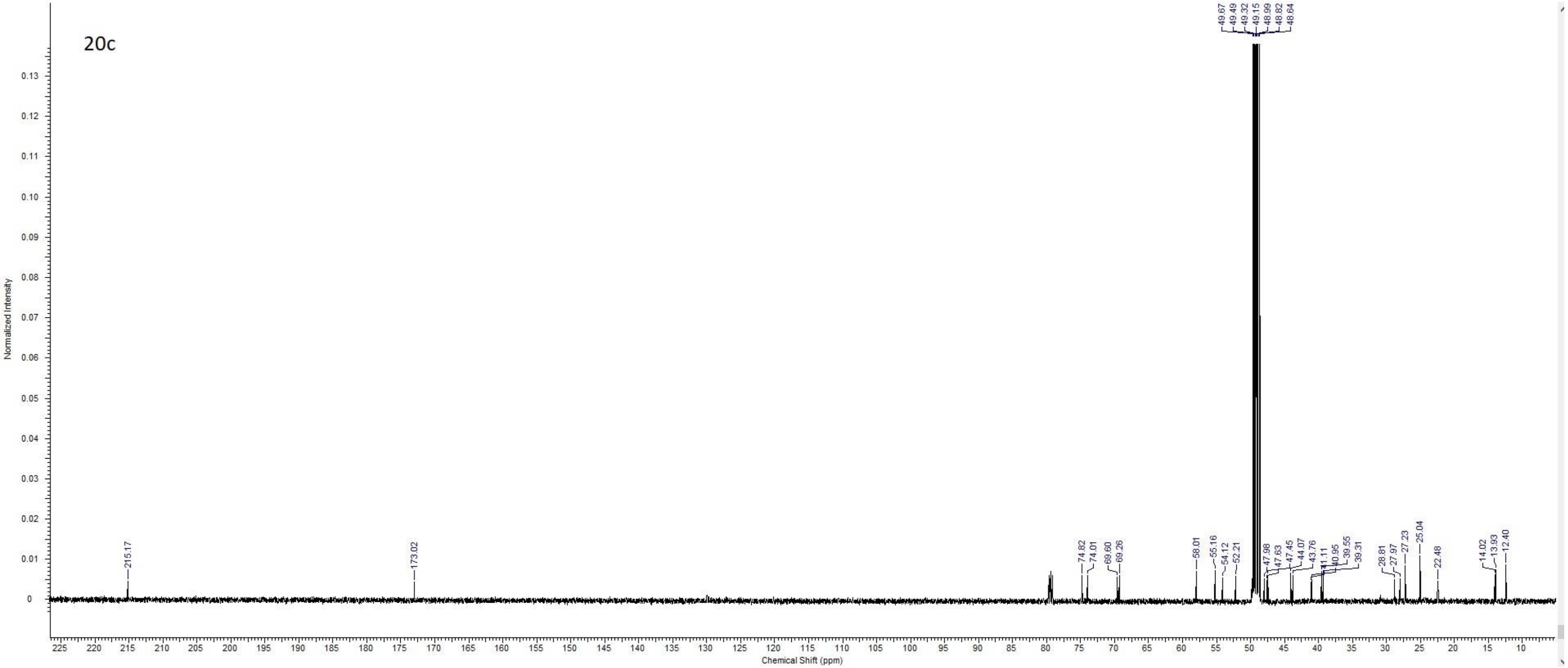
20c
Normalized Intensity
Chemical Shift (ppm)
49.67
49.49
49.32
49.15
48.99
48.82
48.64
215.17
173.02
74.82
74.01
69.60
69.26
58.01
55.16
54.12
52.21
47.98
47.63
47.45
44.07
43.76
41.11
40.95
39.55
39.31
28.81
27.97
27.23
25.04
22.48
14.02
13.93
12.40

Elemental Composition

File Edit View Process Help

Single Mass Analysis

Tolerance = 1.0 PPM / DBE: min = -1.5, max = 50.0

Element prediction: Off

Number of isotope peaks used for i-FIT = 3

Monoisotopic Mass, Even Electron Ions

677 formula(e) evaluated with 1 results within limits (up to 50 best isotopic matches for each mass)

Elements Used:

Mass	Calc. Mass	mDa	PPM	DBE	Formula	i-FIT	i-FIT Norm	Fit Conf %	C	H	N	O
492.3325	492.3325	0.0	0.0	6.5	C28 H46 N O6	41.8	n/a	n/a	28	46	1	6

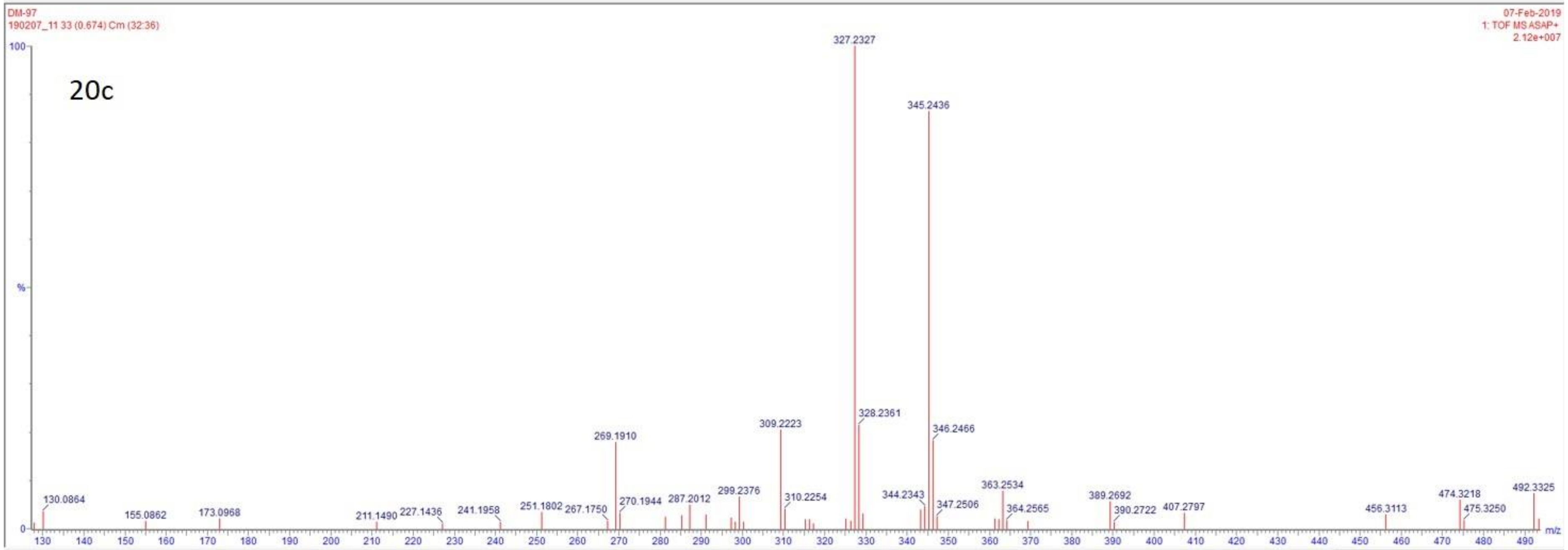

For Help, press F1

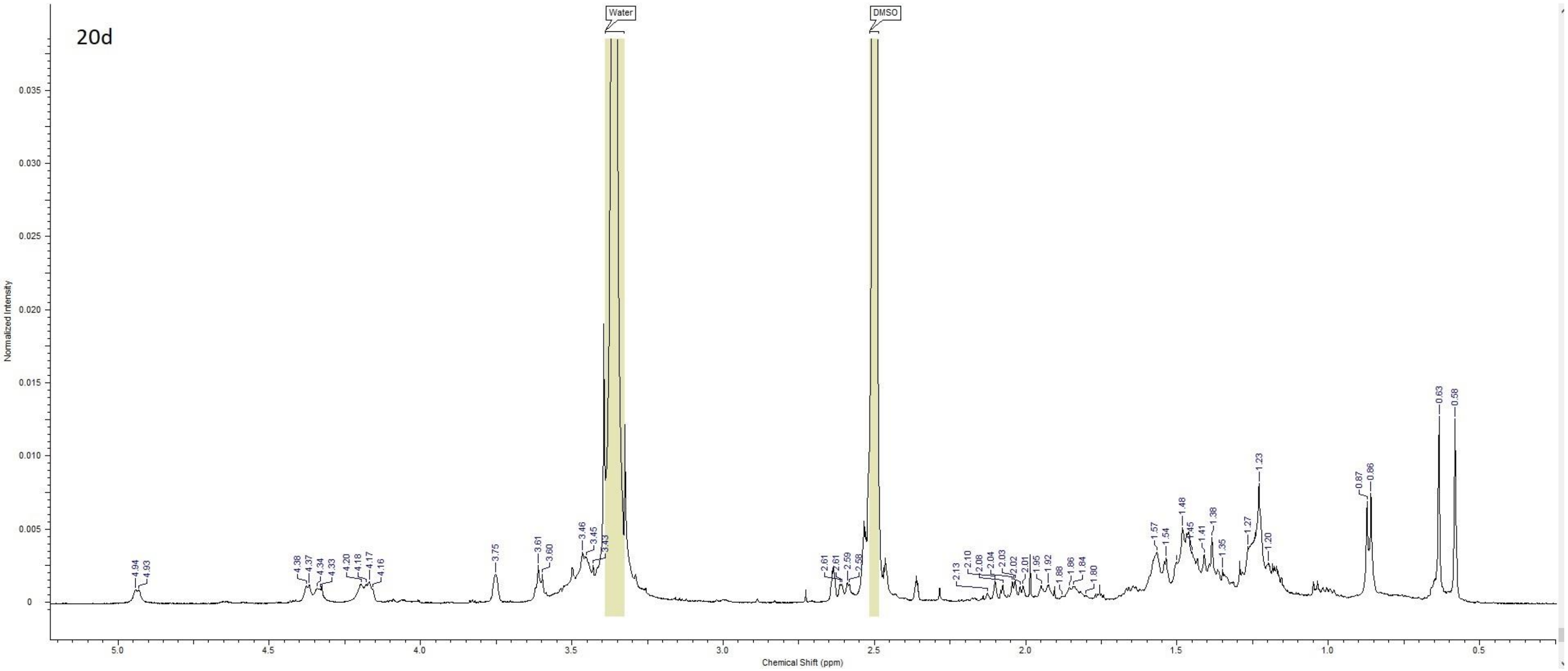
20d
Water
DMSO
Normalized Intensity
Chemical Shift (ppm)
4.94
4.93
4.38
4.37
4.34
4.33
4.20
4.18
4.17
4.16
3.75
3.61
3.60
3.46
3.45
3.43
2.61
2.61
2.59
2.58
2.13
2.10
2.08
2.04
2.03
2.02
2.01
1.95
1.92
1.88
1.86
1.84
1.80
1.57
1.54
1.48
1.45
1.41
1.38
1.35
1.27
1.23
1.20
0.87
0.86
0.63
0.58

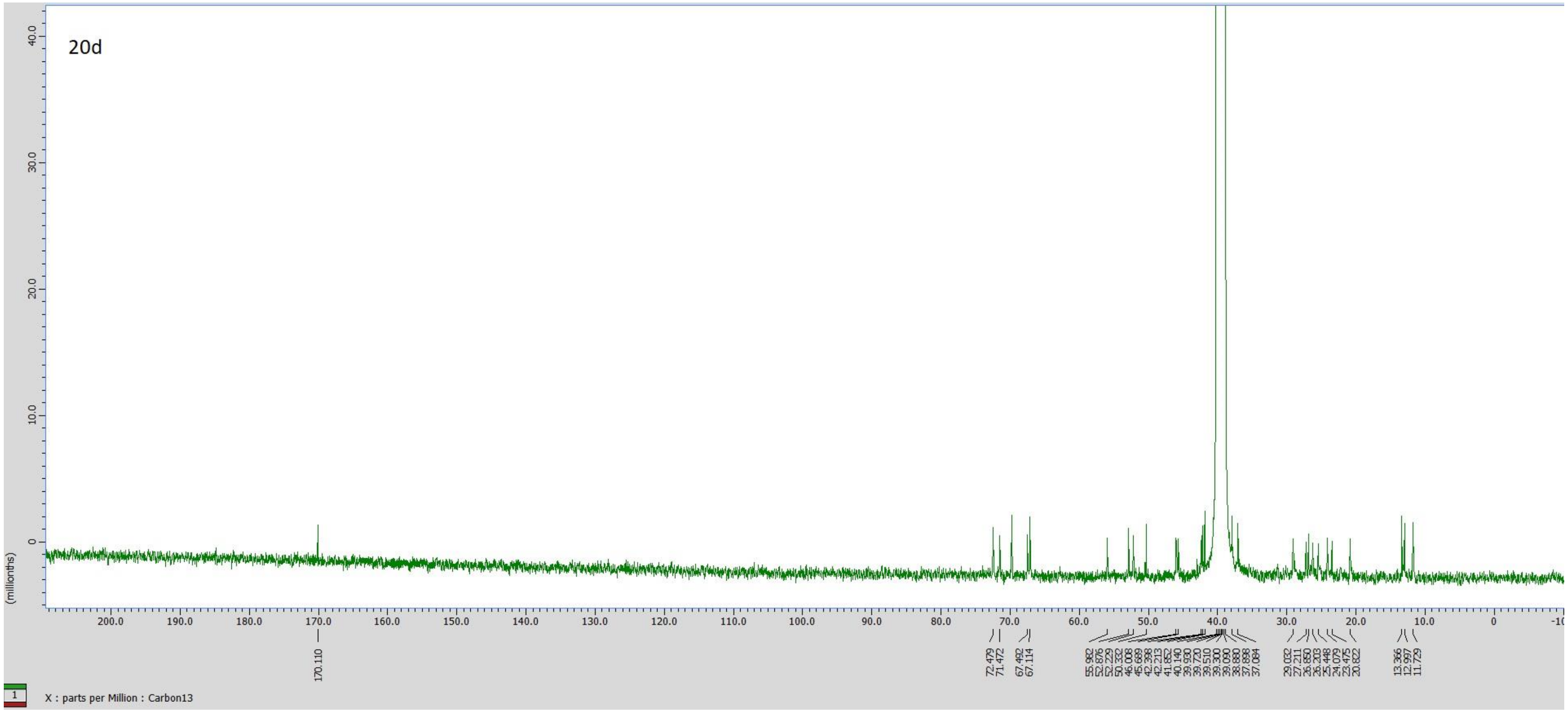

20d
(millionths)
40.0
30.0
20.0
10.0
0
200.0
190.0
180.0
170.0
160.0
150.0
140.0
130.0
120.0
110.0
100.0
90.0
80.0
70.0
60.0
50.0
40.0
30.0
20.0
10.0
0
-10
170.110
72.479
71.472
67.492
67.114
55.982
52.876
52.229
50.332
46.008
45.689
42.398
42.213
41.852
40.140
39.930
39.720
39.510
39.300
39.090
38.880
37.898
37.084
29.032
27.211
26.850
26.203
25.448
24.079
23.475
20.822
13.366
12.997
11.729
1
X : parts per Million : Carbon13

Elemental Composition

File Edit View Process Help

Single Mass Analysis

Tolerance = 1.0 PPM / DBE: min = -1.5, max = 50.0

Element prediction: Off

Number of isotope peaks used for i-FIT = 3

Monoisotopic Mass, Even Electron Ions

712 formula(e) evaluated with 1 results within limits (up to 50 best isotopic matches for each mass)

Elements Used:

Mass	Calc. Mass	mDa	PPM	DBE	Formula	i-FIT	i-FIT Norm	Fit Conf %	C	H	N	O
506.3479	506.3482	-0.3	-0.6	6.5	C29 H48 N O6	39.5	n/a	n/a	29	48	1	6

20d

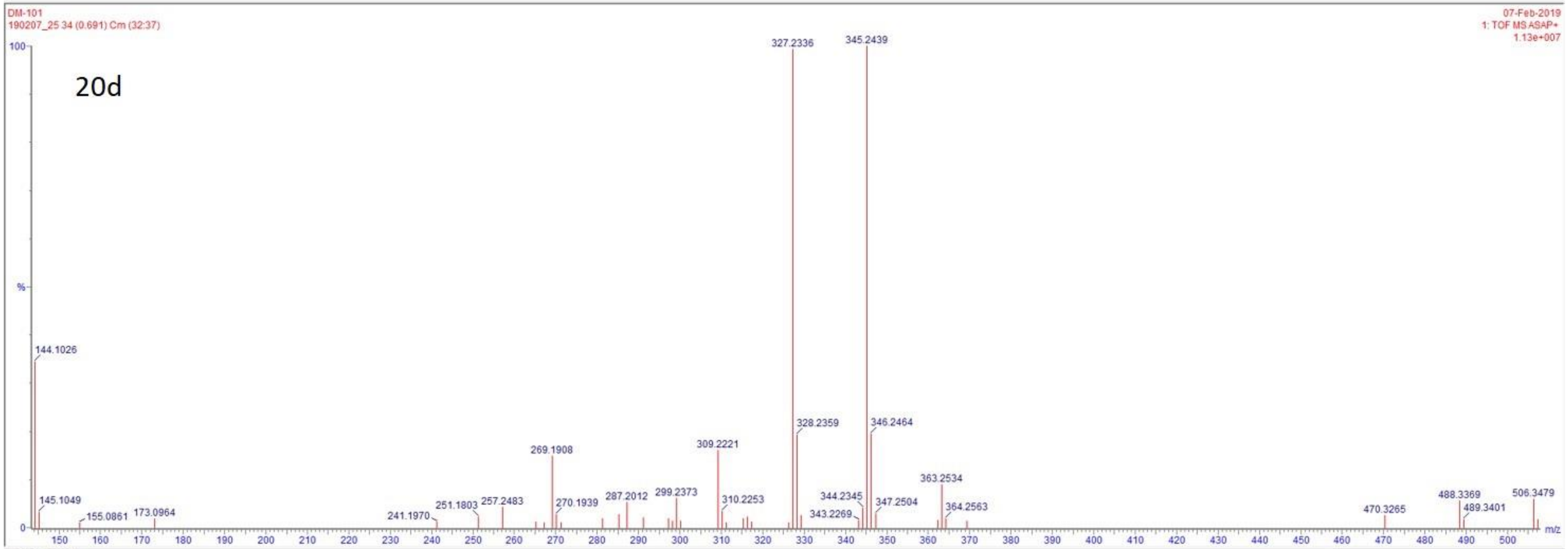

For Help, press F1

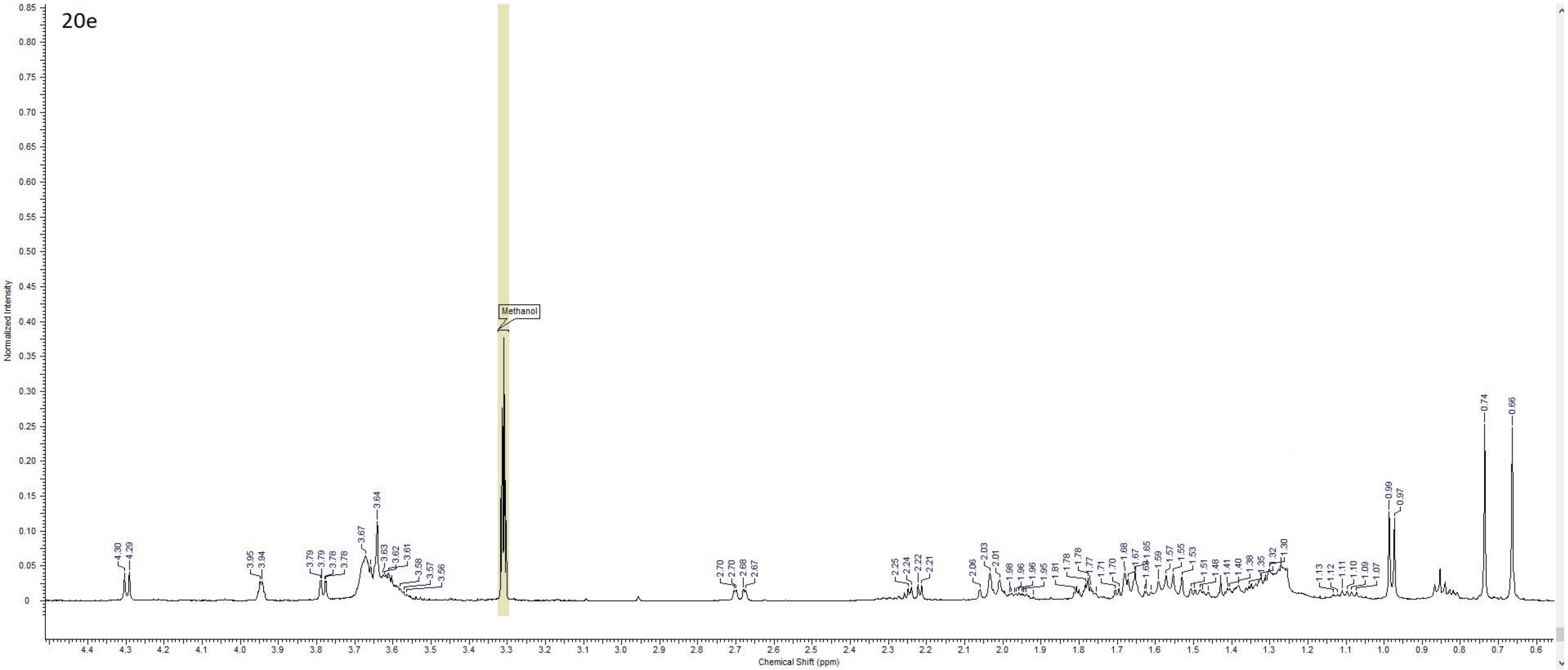
20e
Methanol
Normalized Intensity
Chemical Shift (ppm)
4.30
4.29
3.95
3.94
3.79
3.79
3.78
3.78
3.67
3.64
3.63
3.62
3.61
3.58
3.57
3.56
2.70
2.70
2.68
2.67
2.25
2.24
2.22
2.21
2.06
2.03
2.01
1.98
1.96
1.96
1.95
1.81
1.78
1.78
1.77
1.71
1.70
1.68
1.67
1.65
1.59
1.57
1.55
1.53
1.51
1.48
1.41
1.40
1.38
1.35
1.32
1.30
1.13
1.12
1.11
1.10
1.09
1.07
0.99
0.97
0.74
0.66

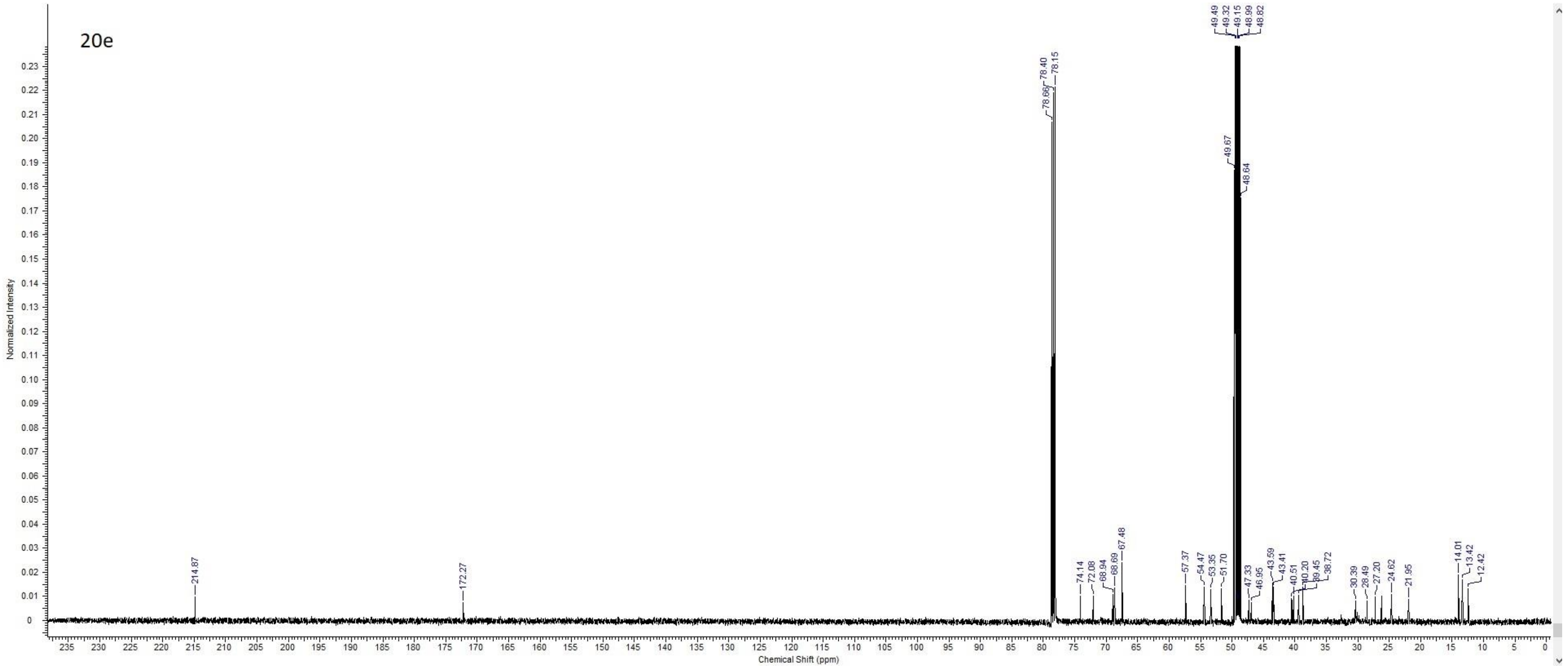
20e
Normalized Intensity
Chemical Shift (ppm)
214.87
172.27
78.66
78.40
78.15
74.14
72.08
68.94
68.69
67.48
57.37
54.47
53.35
51.70
49.67
49.49
49.32
49.15
48.99
48.82
48.64
47.33
46.95
43.59
43.41
40.51
40.20
39.45
38.72
30.39
28.49
27.20
24.62
21.95
14.01
13.42
12.42

Elemental Composition

File Edit View Process Help

Single Mass Analysis
Tolerance = 1.0 PPM / DBE: min = -1.5, max = 50.0
Element prediction: Off
Number of isotope peaks used for i-FIT = 3
Monoisotopic Mass, Even Electron Ions
716 formula(e) evaluated with 1 results within limits (up to 50 best isotopic matches for each mass)
Elements Used:

Mass	Calc. Mass	mDa	PPM	DBE	Formula	i-FIT	i-FIT Norm	Fit Conf %	C	H	N	O
508.3272	508.3274	-0.2	-0.4	6.5	C28 H46 N O7	95.1	n/a	n/a	28	46	1	7

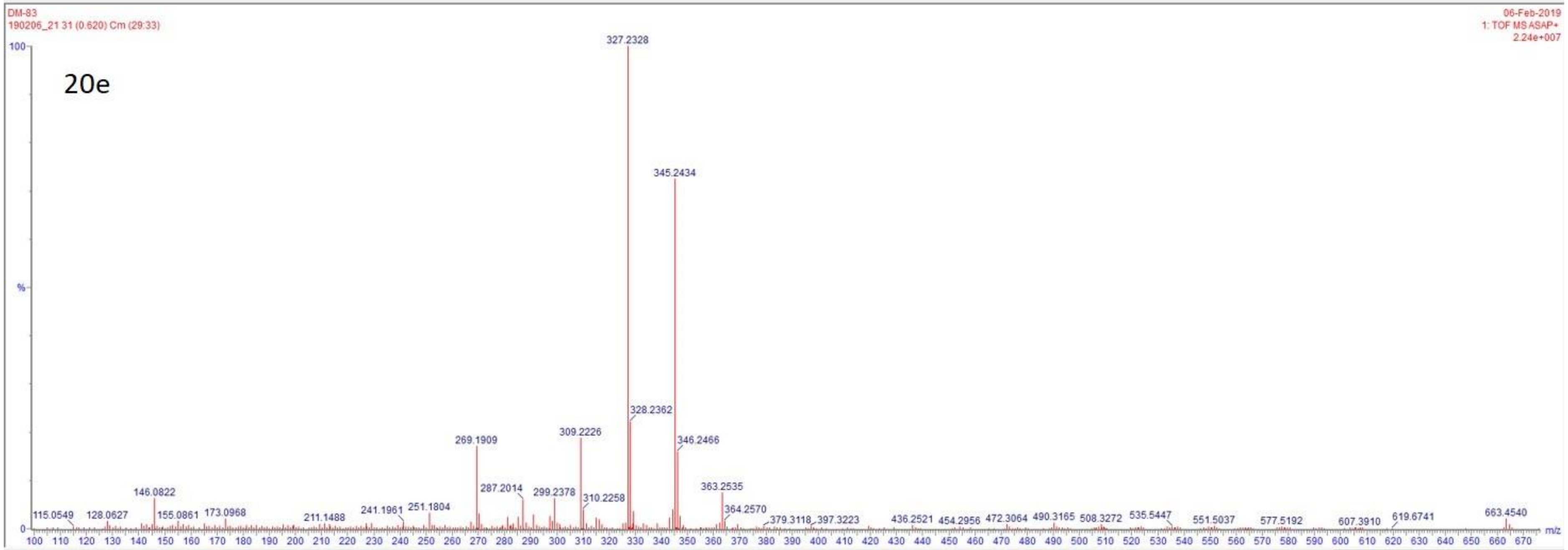

For Help, press F1

21a

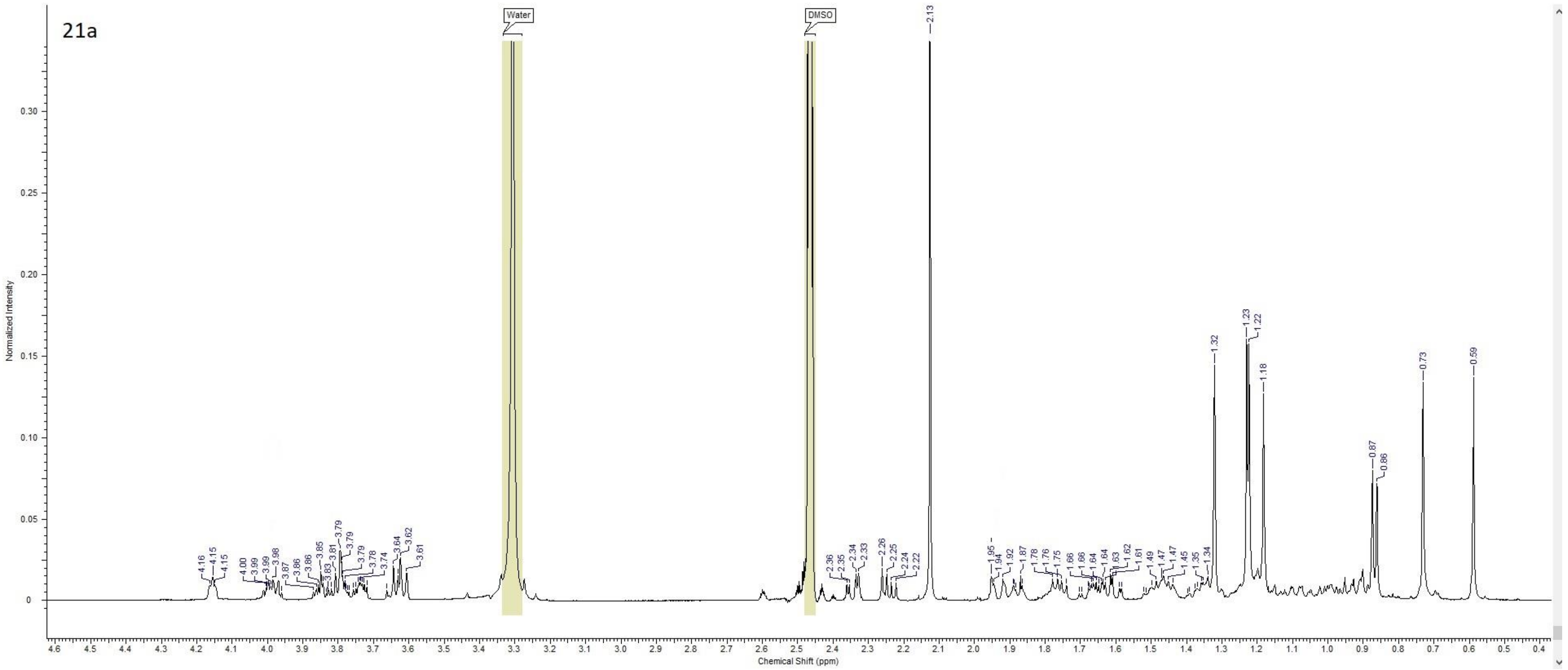
Water
DMSO
2.13
1.32
1.23
1.22
1.18
0.87
0.86
0.73
0.59
Normalized Intensity
Chemical Shift (ppm)

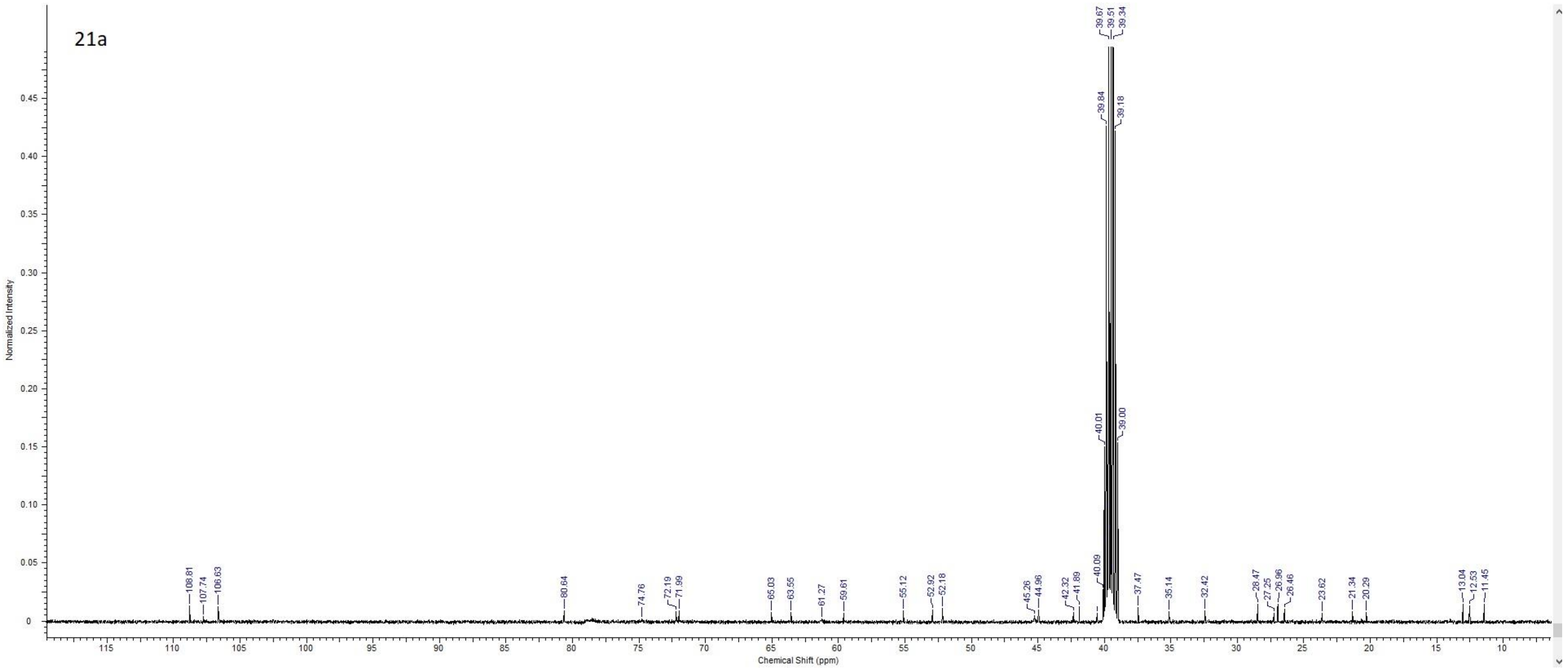
21a
Normalized Intensity
Chemical Shift (ppm)

Elemental Composition

File Edit View Process Help

Single Mass Analysis

Tolerance = 1.0 PPM / DBE: min = -1.5, max = 50.0
Element prediction: Off
Number of isotope peaks used for i-FIT = 3
Monoisotopic Mass, Even Electron Ions
923 formula(e) evaluated with 1 results within limits (up to 50 best isotopic matches for each mass)
Elements Used:

Mass	Calc. Mass	mDa	PPM	DBE	Formula	i-FIT	i-FIT Norm	Fit Conf %	C	H	N	O
576.4269	576.4264	0.5	0.9	6.5	C34 H58 N O6	29.2	n/a	n/a	34	58	1	6

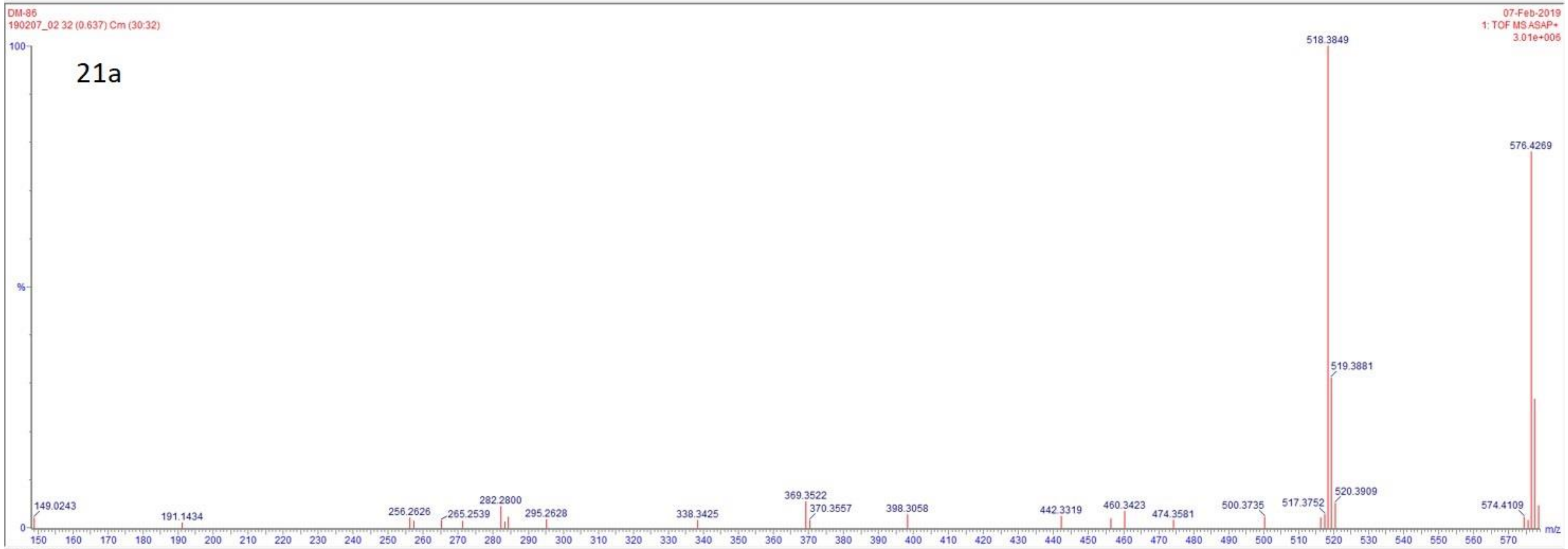

For Help, press F1

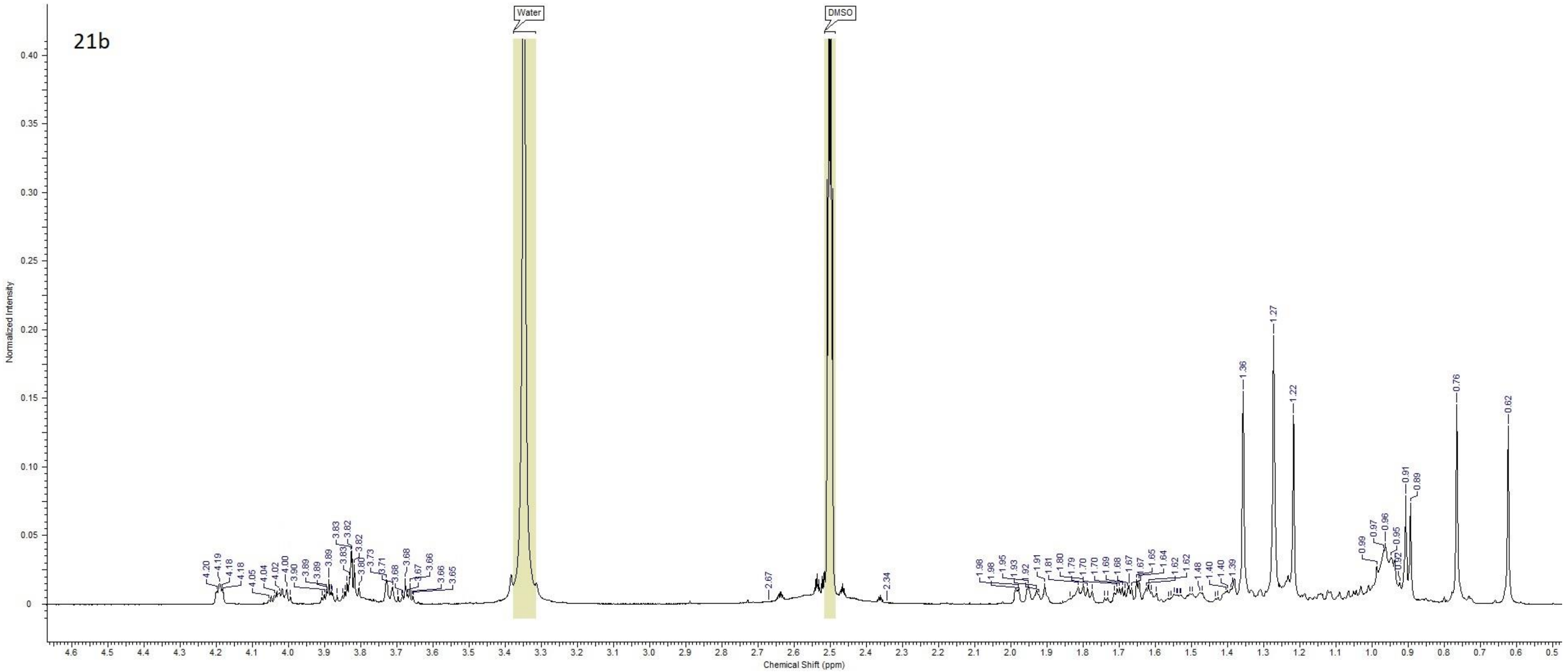
21b
Water
DMSO
Normalized Intensity
Chemical Shift (ppm)

21b

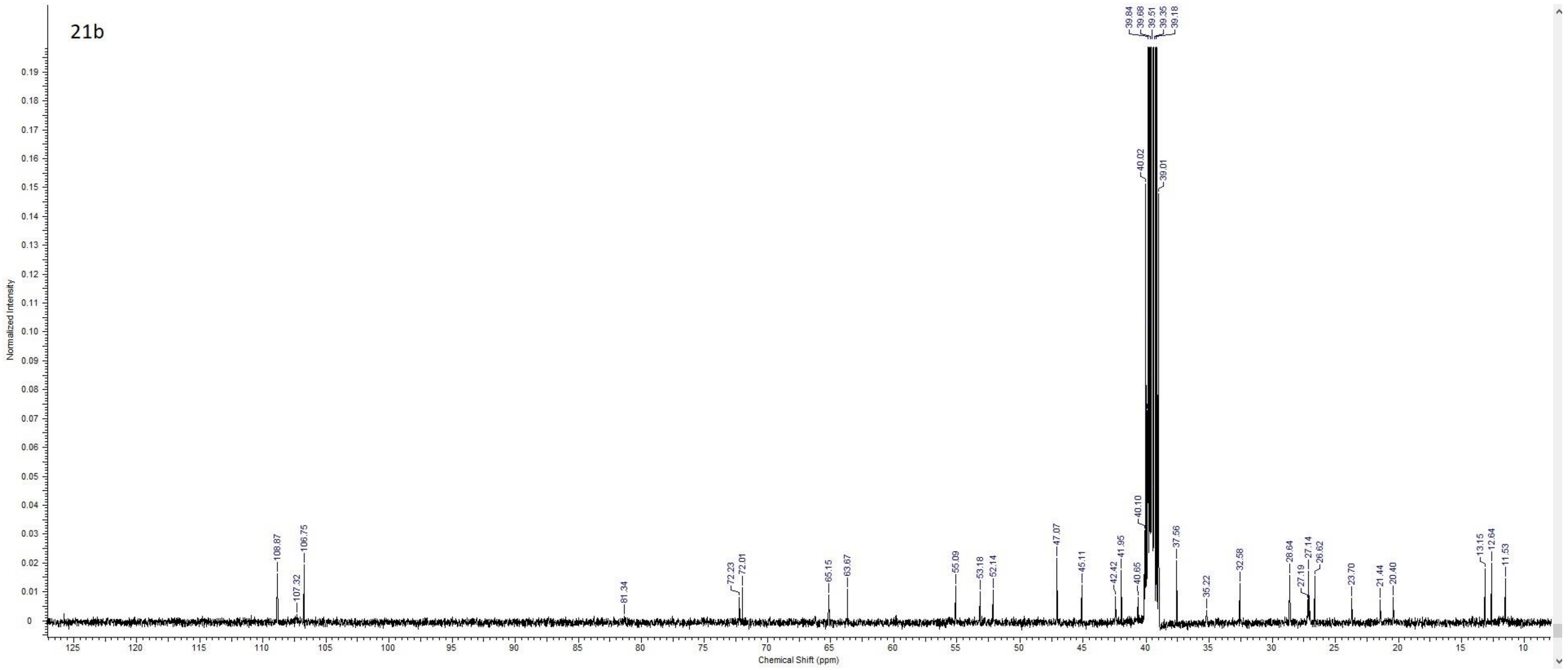

Normalized Intensity
Chemical Shift (ppm)
108.87
107.32
106.75
81.34
72.23
72.01
65.15
63.67
55.09
53.18
52.14
47.07
45.11
42.42
41.95
40.65
40.10
40.02
39.84
39.68
39.51
39.35
39.18
39.01
37.56
35.22
32.58
28.64
27.19
27.14
26.62
23.70
21.44
20.40
13.15
12.64
11.53

Elemental Composition

File Edit View Process Help

Single Mass Analysis
Tolerance = 1.0 PPM / DBE: min = -1.5, max = 50.0
Element prediction: Off
Number of isotope peaks used for i-FIT = 3
Monoisotopic Mass, Even Electron Ions
1009 formula(e) evaluated with 1 results within limits (up to 50 best isotopic matches for each mass)
Elements Used:

Mass	Calc. Mass	mDa	PPM	DBE	Formula	i-FIT	i-FIT Norm	Fit Conf %	C	H	N	O
604.4579	604.4577	0.2	0.3	6.5	C36 H62 N O6	93.8	n/a	n/a	36	62	1	6

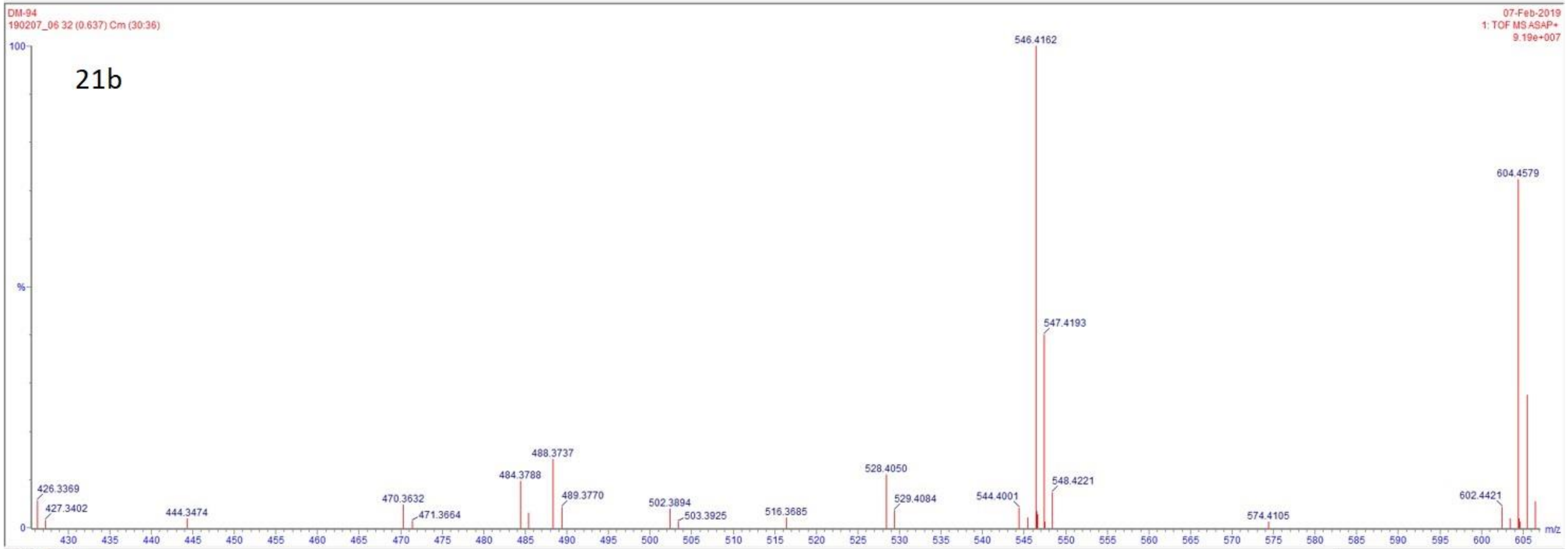

For Help, press F1

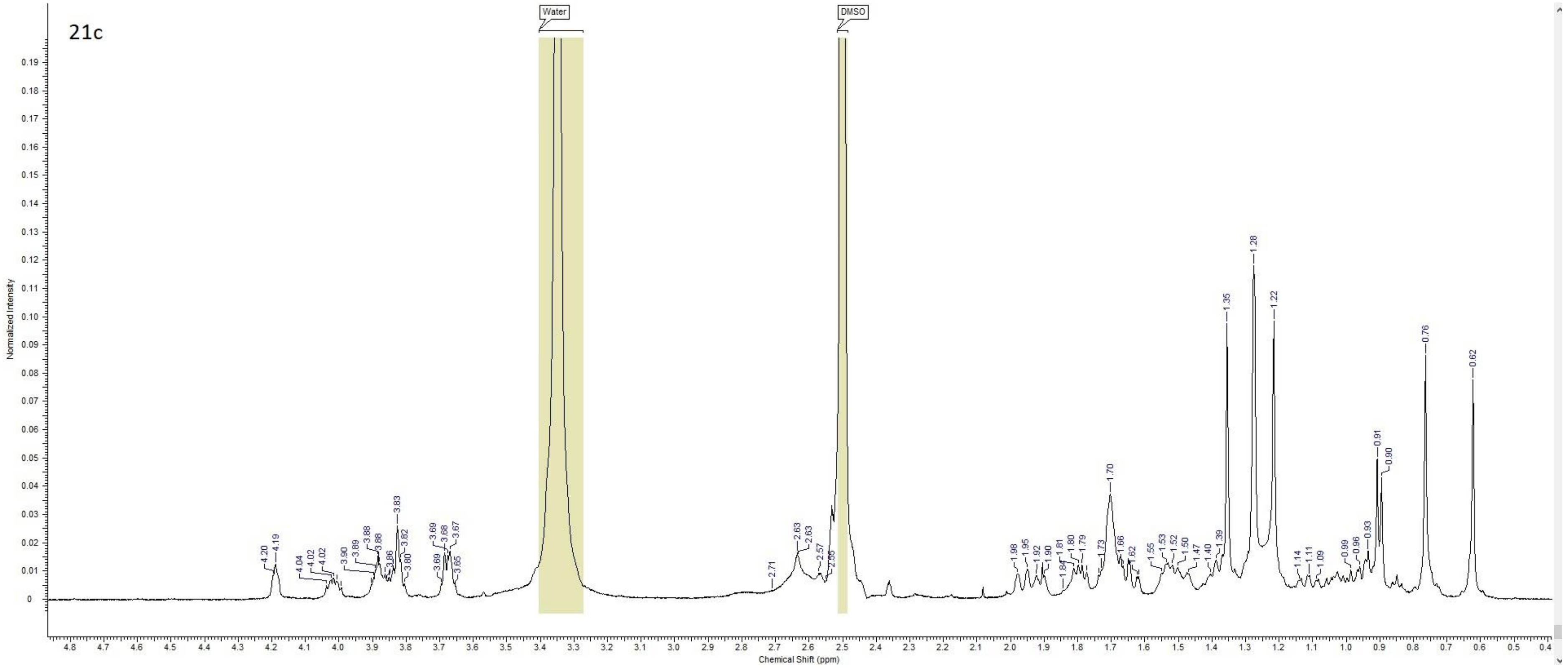
21c
Water
DMSO
Normalized Intensity
Chemical Shift (ppm)
4.20
4.19
4.04
4.02
4.02
3.90
3.89
3.88
3.88
3.86
3.83
3.82
3.80
3.69
3.69
3.68
3.67
3.65
2.71
2.63
2.63
2.57
2.55
1.98
1.95
1.92
1.90
1.84
1.81
1.80
1.79
1.73
1.70
1.66
1.62
1.55
1.53
1.52
1.50
1.47
1.40
1.39
1.35
1.28
1.22
1.14
1.11
1.09
0.99
0.96
0.93
0.91
0.90
0.76
0.62

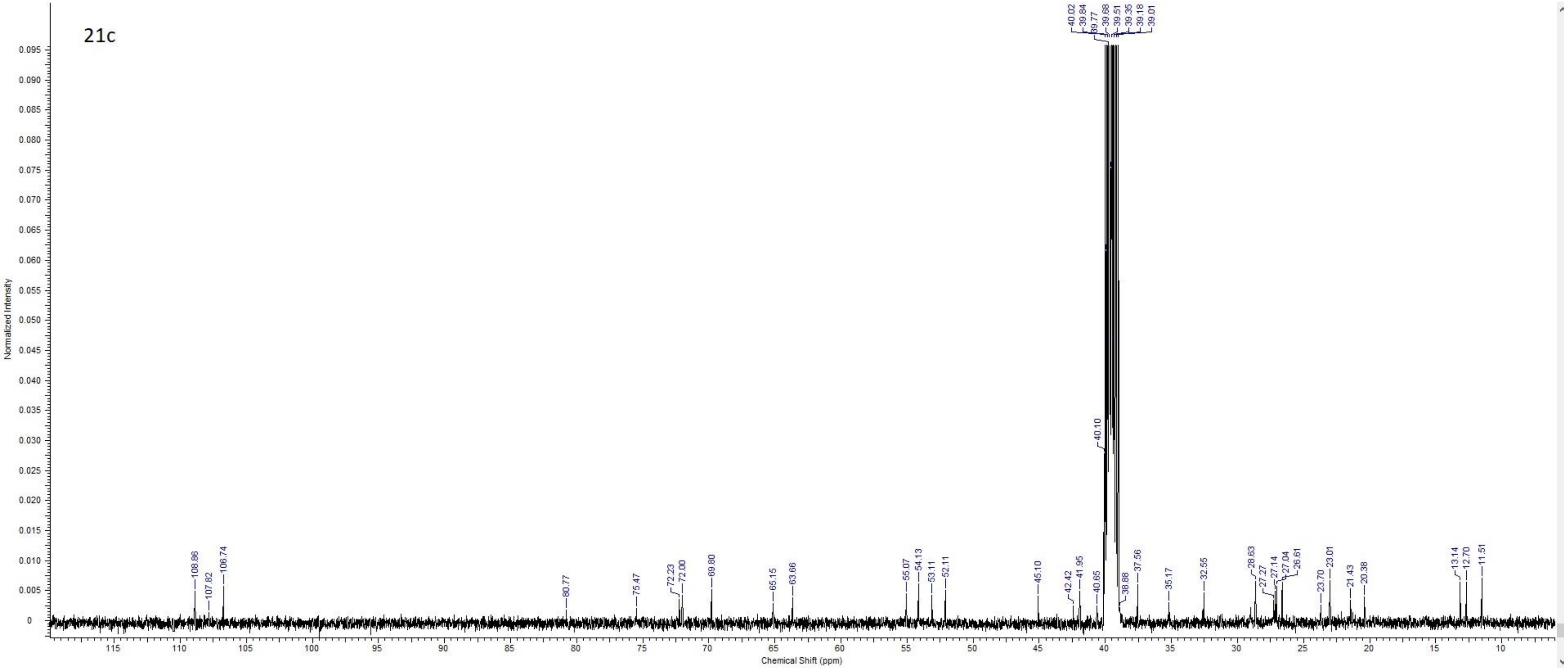
21c
Normalized Intensity
Chemical Shift (ppm)
108.86
107.82
106.74
80.77
75.47
72.23
72.00
69.80
65.15
63.66
55.07
54.13
53.11
52.11
45.10
42.42
41.95
40.65
40.10
40.02
39.84
39.77
39.68
39.51
39.35
39.18
39.01
38.88
37.56
35.17
32.55
28.63
27.27
27.14
27.04
26.61
23.70
23.01
21.43
20.38
13.14
12.70
11.51

Elemental Composition

File Edit View Process Help

Single Mass Analysis

Tolerance = 1.0 PPM / DBE: min = -1.5, max = 50.0

Element prediction: Off

Number of isotope peaks used for i-FIT = 3

Monoisotopic Mass, Even Electron Ions

1001 formula(e) evaluated with 1 results within limits (up to 50 best isotopic matches for each mass)

Elements Used:

Mass	Calc. Mass	mDa	PPM	DBE	Formula	i-FIT	i-FIT Norm	Fit Conf %	C	H	N	O
602.4426	602.4421	0.5	0.8	7.5	C36 H60 N O6	89.0	n/a	n/a	36	60	1	6

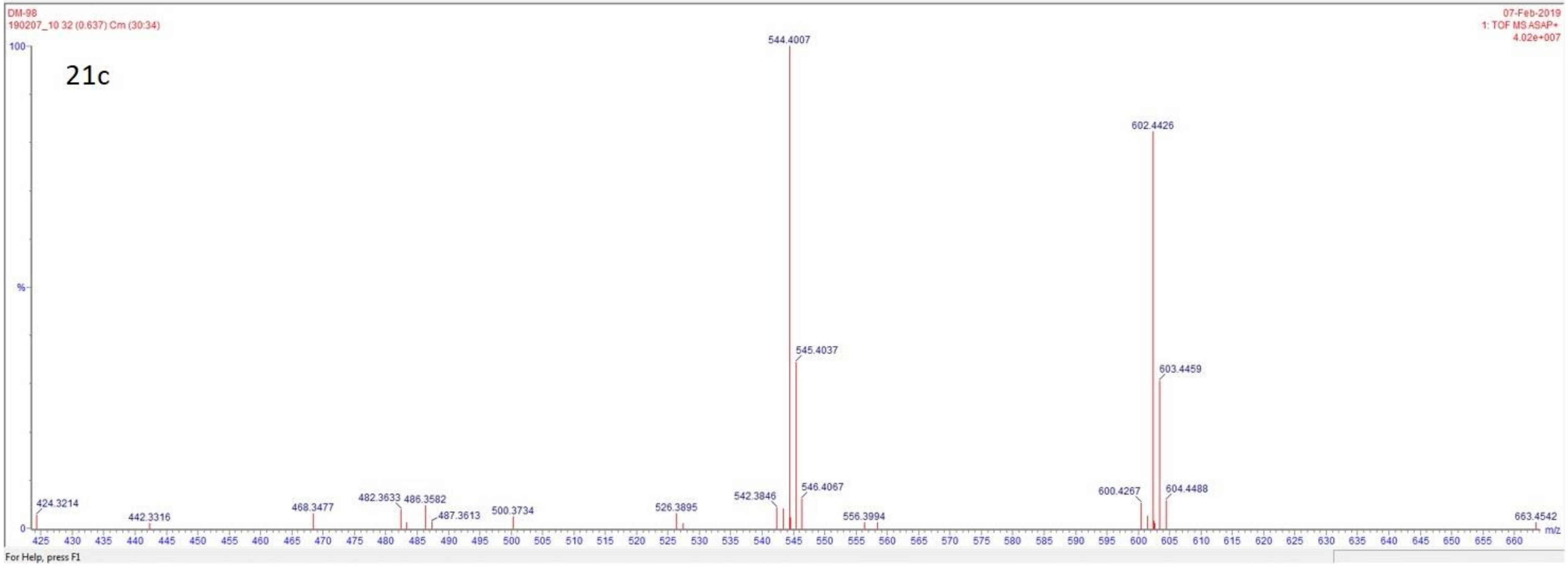

For Help, press F1

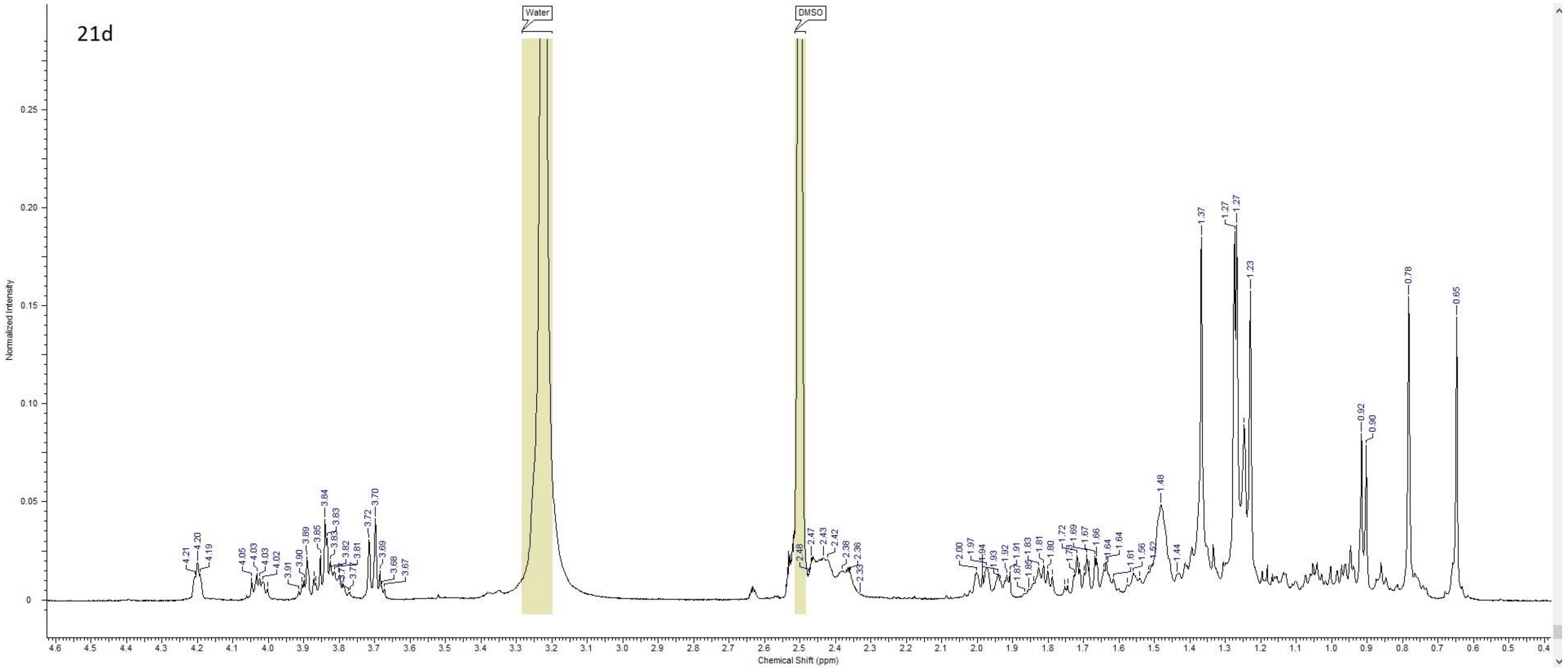

21d
Water
DMSO
Normalized Intensity
Chemical Shift (ppm)

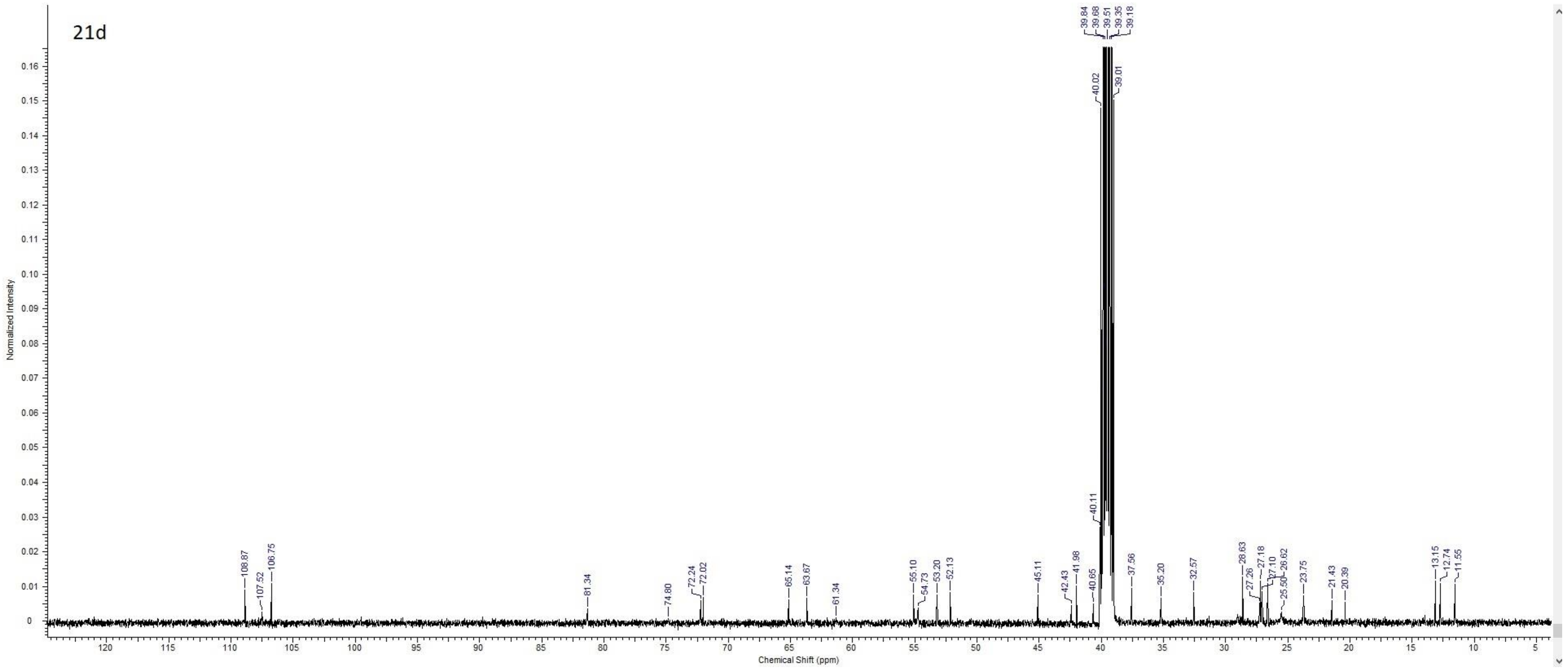
21d
Normalized Intensity
Chemical Shift (ppm)

Elemental Composition

File Edit View Process Help

Single Mass Analysis
Tolerance = 1.0 PPM / DBE: min = -1.5, max = 50.0
Element prediction: Off
Number of isotope peaks used for i-FIT = 3
Monoisotopic Mass, Even Electron Ions
1046 formula(e) evaluated with 1 results within limits (all results (up to 1000) for each mass)
Elements Used:

Mass	Calc. Mass	mDa	PPM	DBE	Formula	i-FIT	i-FIT Norm	Fit Conf %	C	H	N	O
616.4578	616.4577	0.1	0.2	7.5	C37 H62 N O6	31.3	n/a	n/a	37	62	1	6

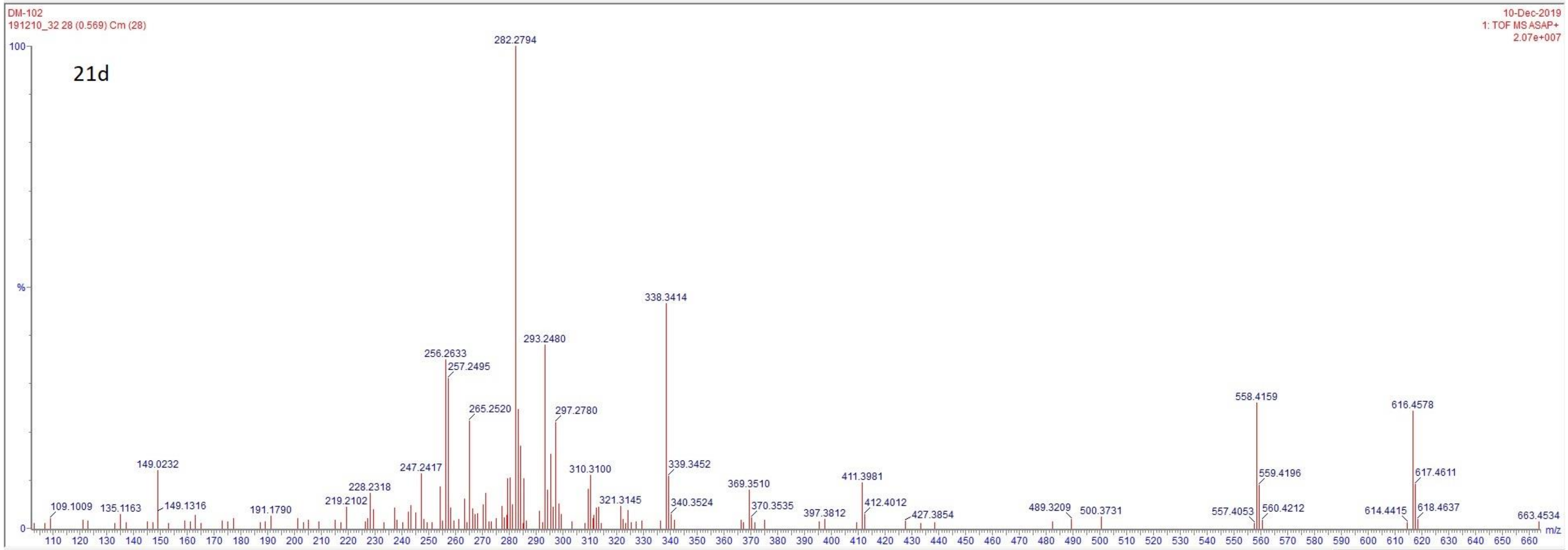

For Help, press F1

21e

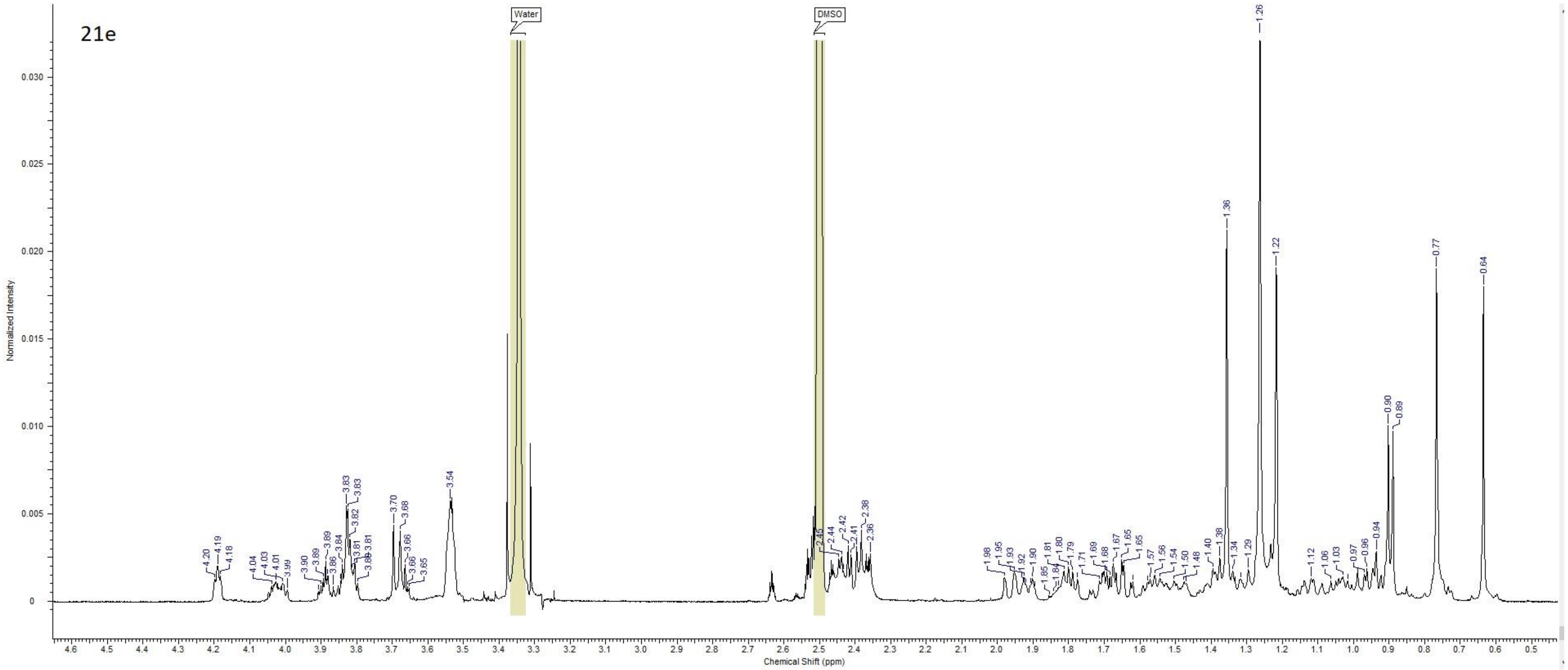

21e

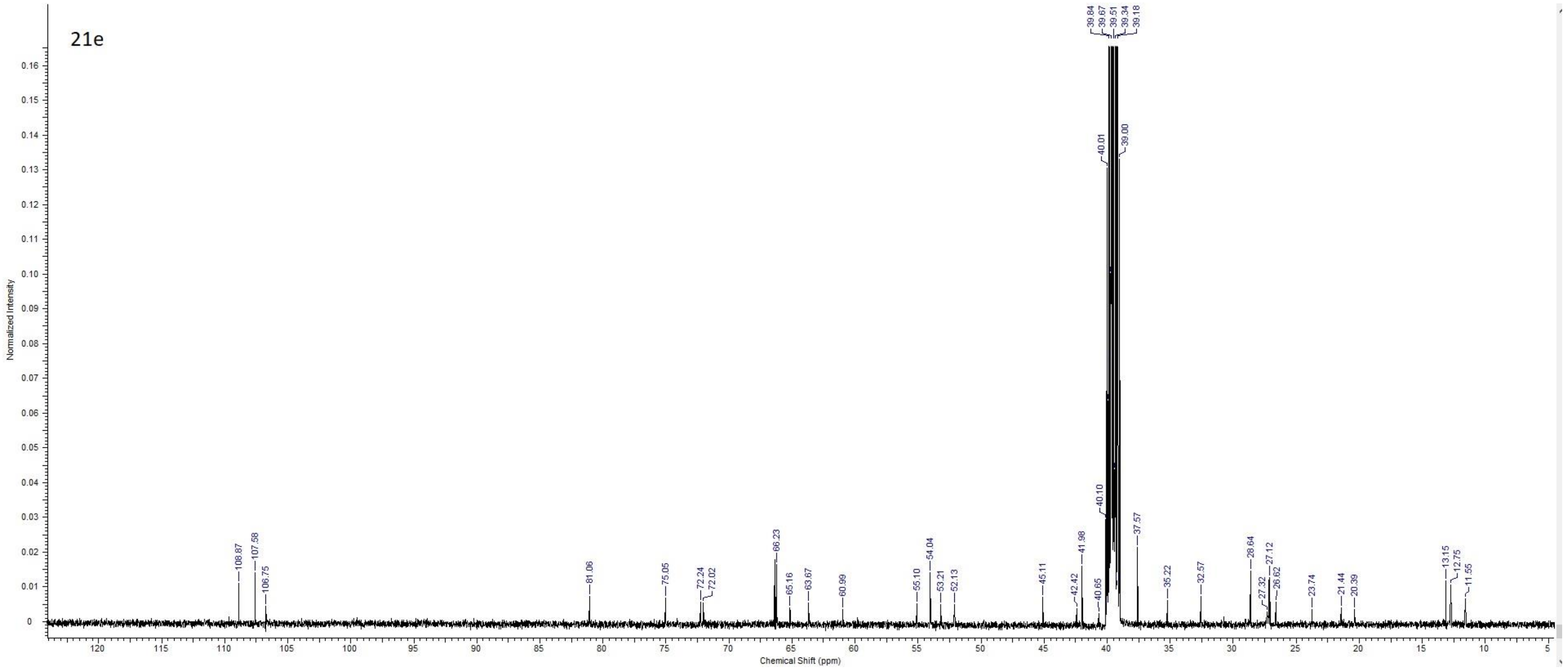

Normalized Intensity
Chemical Shift (ppm)
108.87
107.58
106.75
81.06
75.05
72.24
72.02
66.23
65.16
63.67
60.99
55.10
54.04
53.21
52.13
45.11
42.42
41.98
40.65
40.10
40.01
39.84
39.67
39.51
39.34
39.18
39.00
37.57
35.22
32.57
28.64
27.32
27.12
26.62
23.74
21.44
20.39
13.15
12.75
11.55

Elemental Composition

File Edit View Process Help

Single Mass Analysis

Tolerance = 1.1 PPM / DBE: min = -1.5, max = 50.0

Element prediction: Off

Number of isotope peaks used for i-FIT = 3

Monoisotopic Mass, Even Electron Ions

1049 formula(e) evaluated with 1 results within limits (up to 50 best isotopic matches for each mass)

Elements Used:

Mass	Calc. Mass	mDa	PPM	DBE	Formula	i-FIT	i-FIT Norm	Fit Conf %	C	H	N	O
618.4376	618.4370	0.6	1.0	7.5	C36 H60 N O7	38.3	n/a	n/a	36	60	1	7

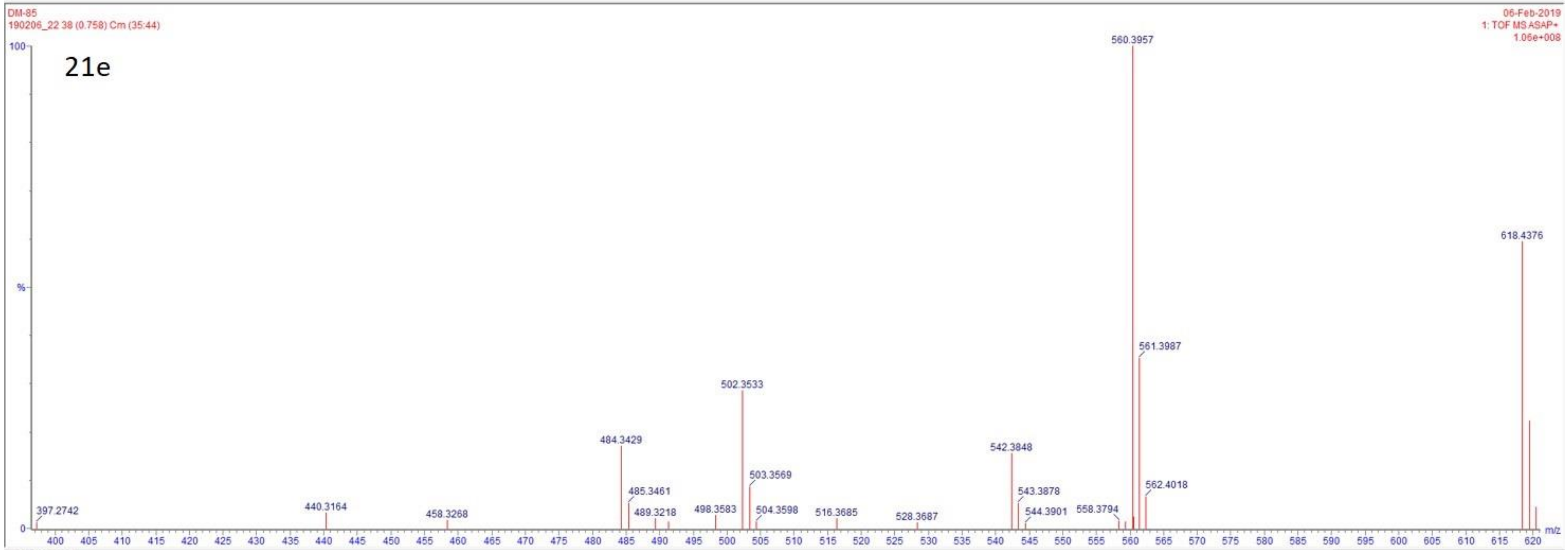

For Help, press F1

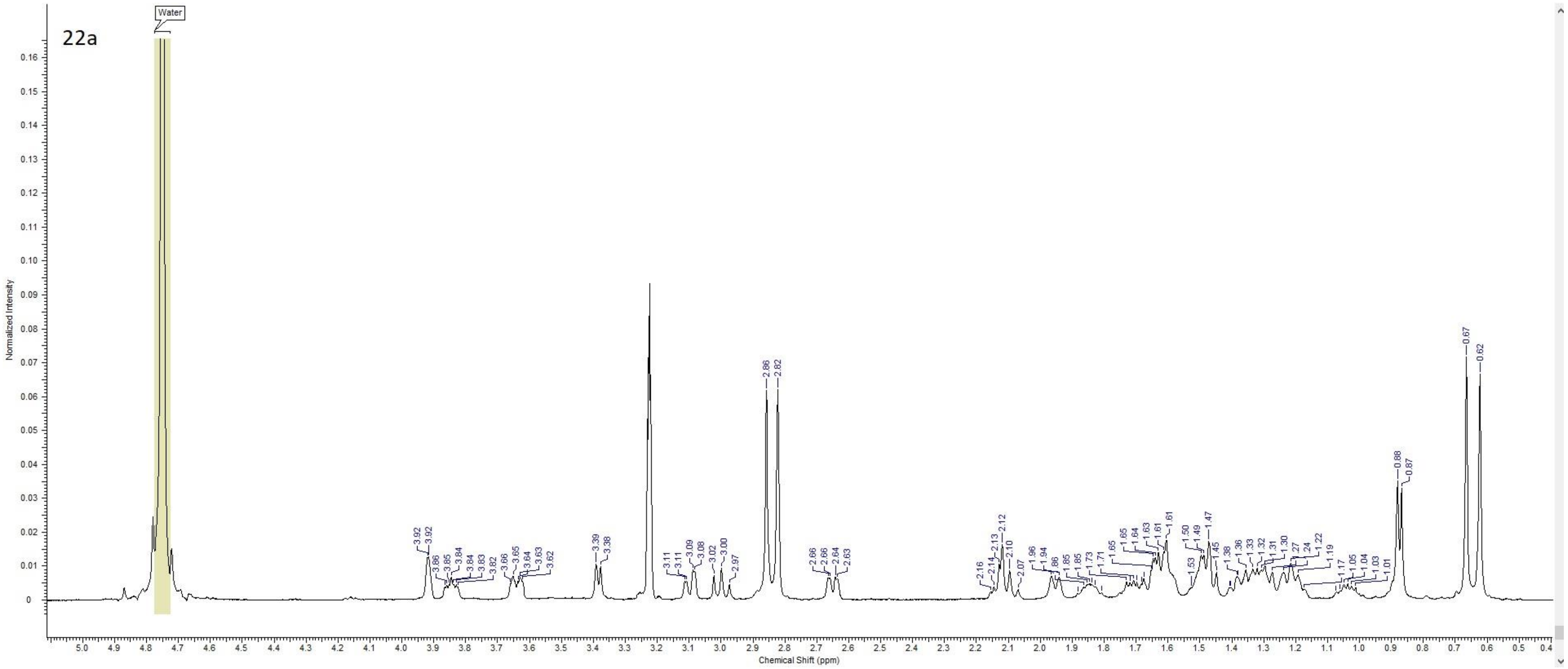
22a
Water
Normalized Intensity
Chemical Shift (ppm)

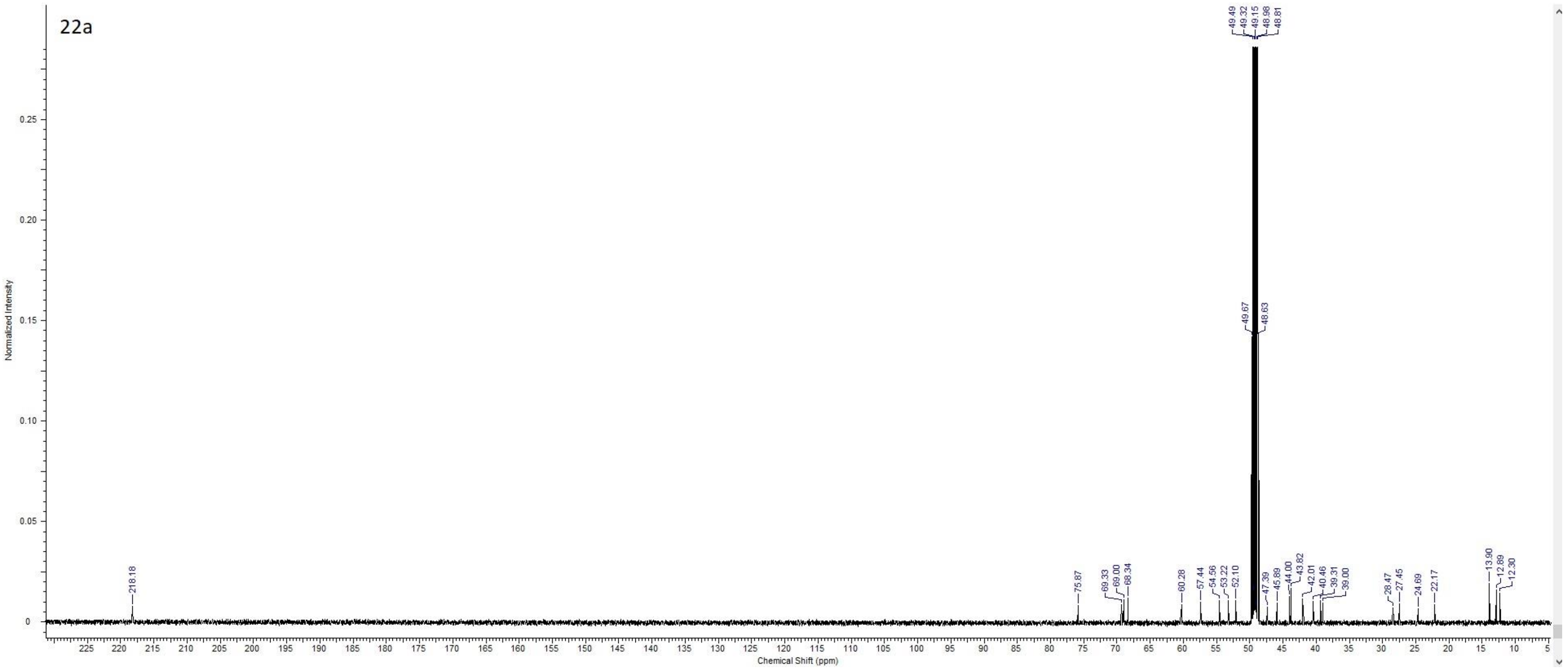

22a
Normalized Intensity
Chemical Shift (ppm)
218.18
75.87
69.33
69.00
68.34
60.28
57.44
54.56
53.22
52.10
49.49
49.32
49.15
48.98
48.81
49.67
48.63
47.39
45.89
44.00
43.82
42.01
40.46
39.31
39.00
28.47
27.45
24.69
22.17
13.90
12.89
12.30

Elemental Composition

File Edit View Process Help

Single Mass Analysis
Tolerance = 1.0 PPM / DBE: min = -1.5, max = 50.0
Element prediction: Off
Number of isotope peaks used for i-FIT = 3
Monoisotopic Mass, Even Electron Ions
579 formula(e) evaluated with 1 results within limits (up to 50 best isotopic matches for each mass)
Elements Used:

Mass	Calc. Mass	mDa	PPM	DBE	Formula	i-FIT	i-FIT Norm	Fit Conf %	C	H	N	O
452.3377	452.3376	0.1	0.2	4.5	C26 H46 N O5	33.2	n/a	n/a	26	46	1	5

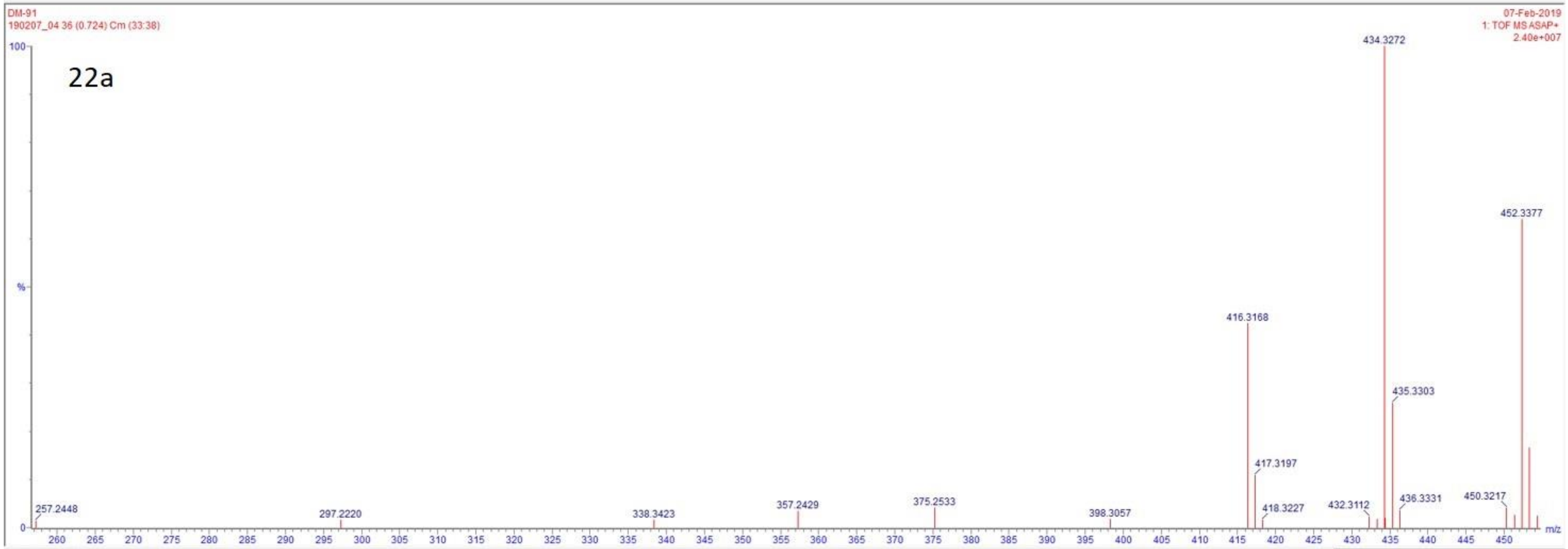

For Help, press F1

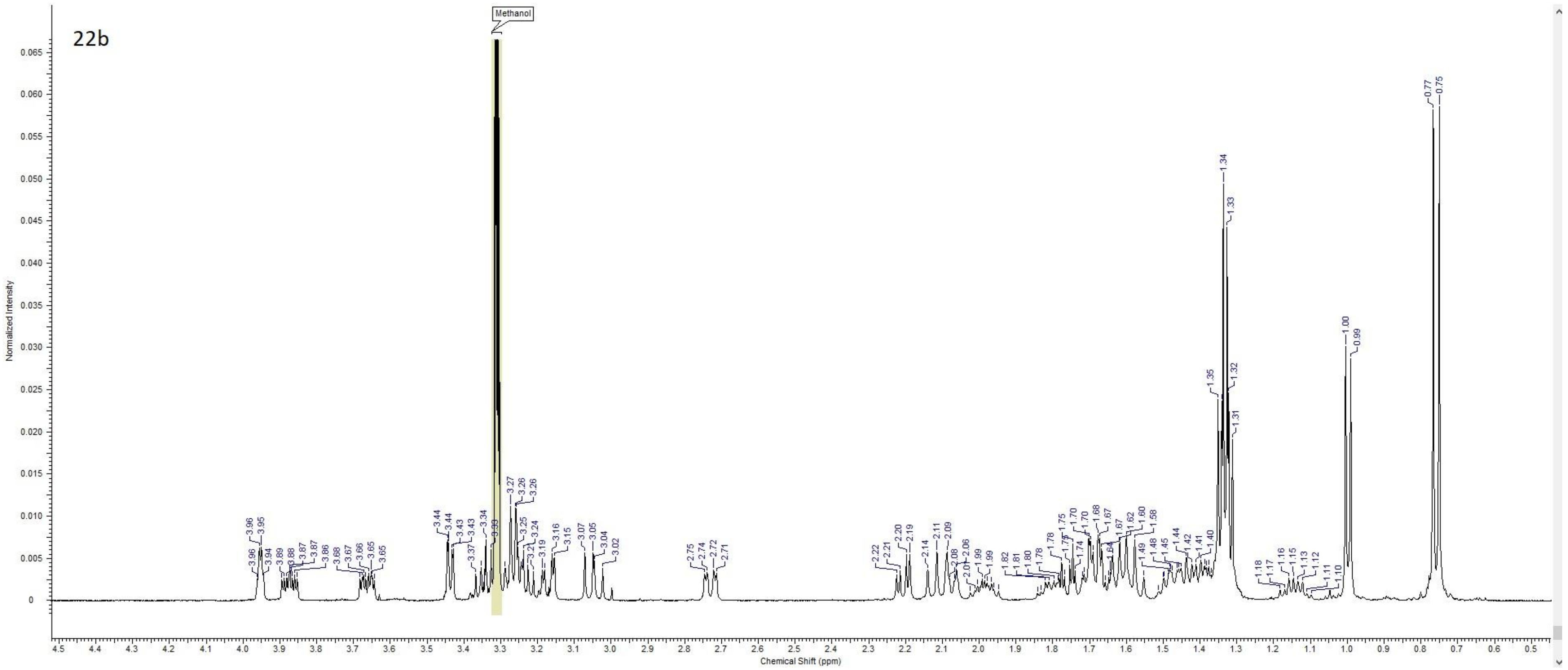
22b
Methanol
Normalized Intensity
Chemical Shift (ppm)

22b

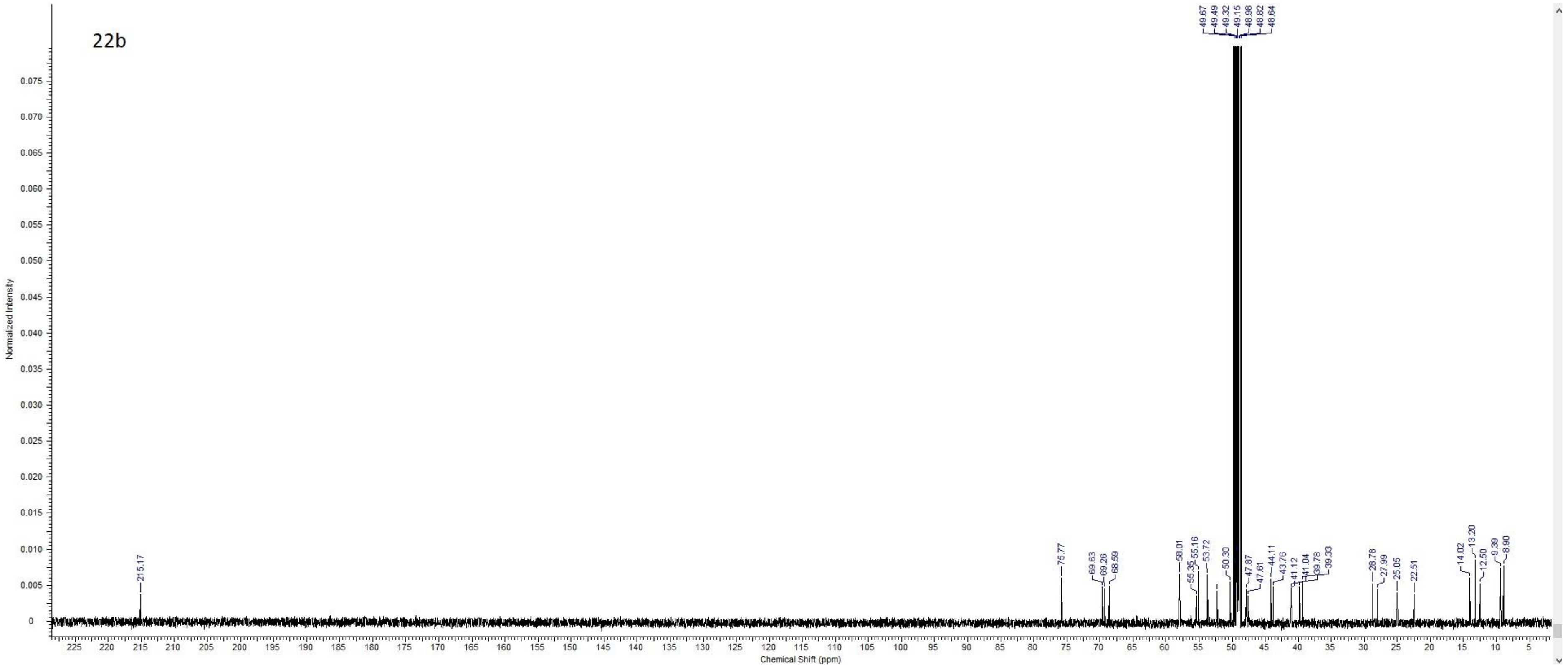
215.17
75.77
69.63
69.26
68.59
58.01
55.35
55.16
53.72
50.30
49.67
49.49
49.32
49.15
48.98
48.82
48.64
47.87
47.61
44.11
43.76
41.12
41.04
39.78
39.33
28.78
27.99
25.05
22.51
14.02
13.20
12.50
9.39
8.90
Normalized Intensity
Chemical Shift (ppm)

Elemental Composition

File Edit View Process Help

Single Mass Analysis

Tolerance = 1.0 PPM / DBE: min = -1.5, max = 50.0

Element prediction: Off

Number of isotope peaks used for i-FIT = 3

Monoisotopic Mass, Even Electron Ions

647 formula(e) evaluated with 1 results within limits (up to 50 best isotopic matches for each mass)

Elements Used:

Mass	Calc. Mass	mDa	PPM	DBE	Formula	i-FIT	i-FIT Norm	Fit Conf %	C	H	N	O
480.3689	480.3689	0.0	0.0	4.5	C28 H50 N O5	27.2	n/a	n/a	28	50	1	5

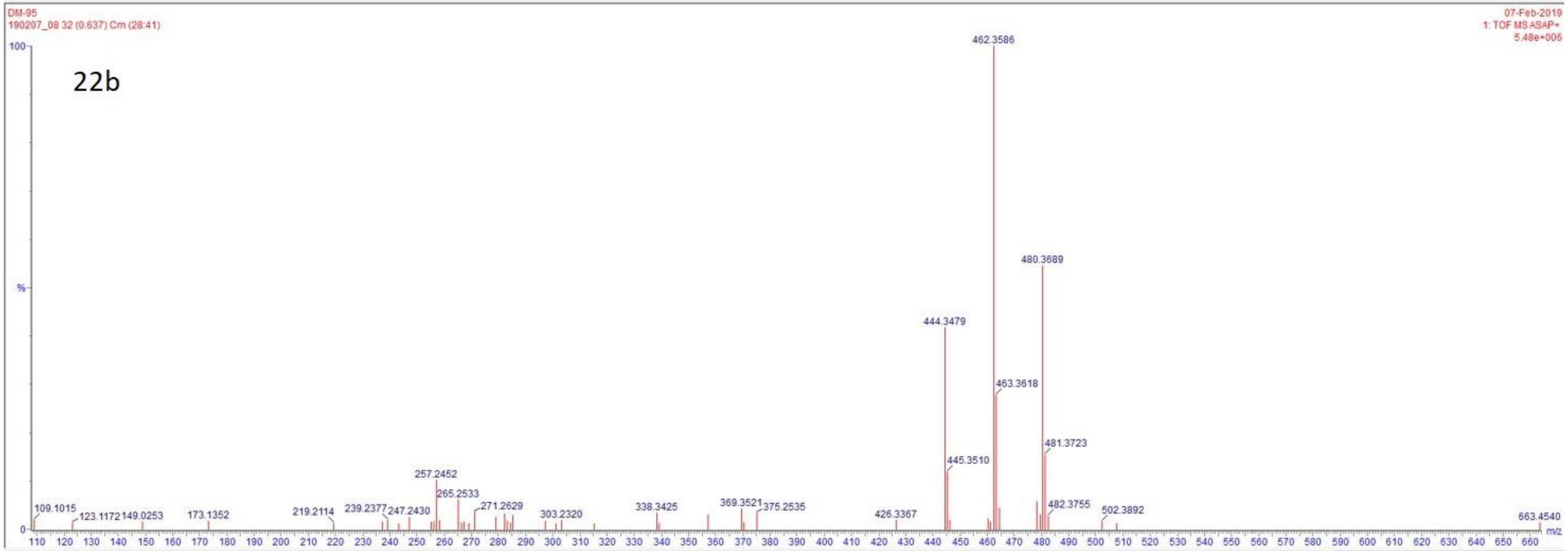

For Help, press F1

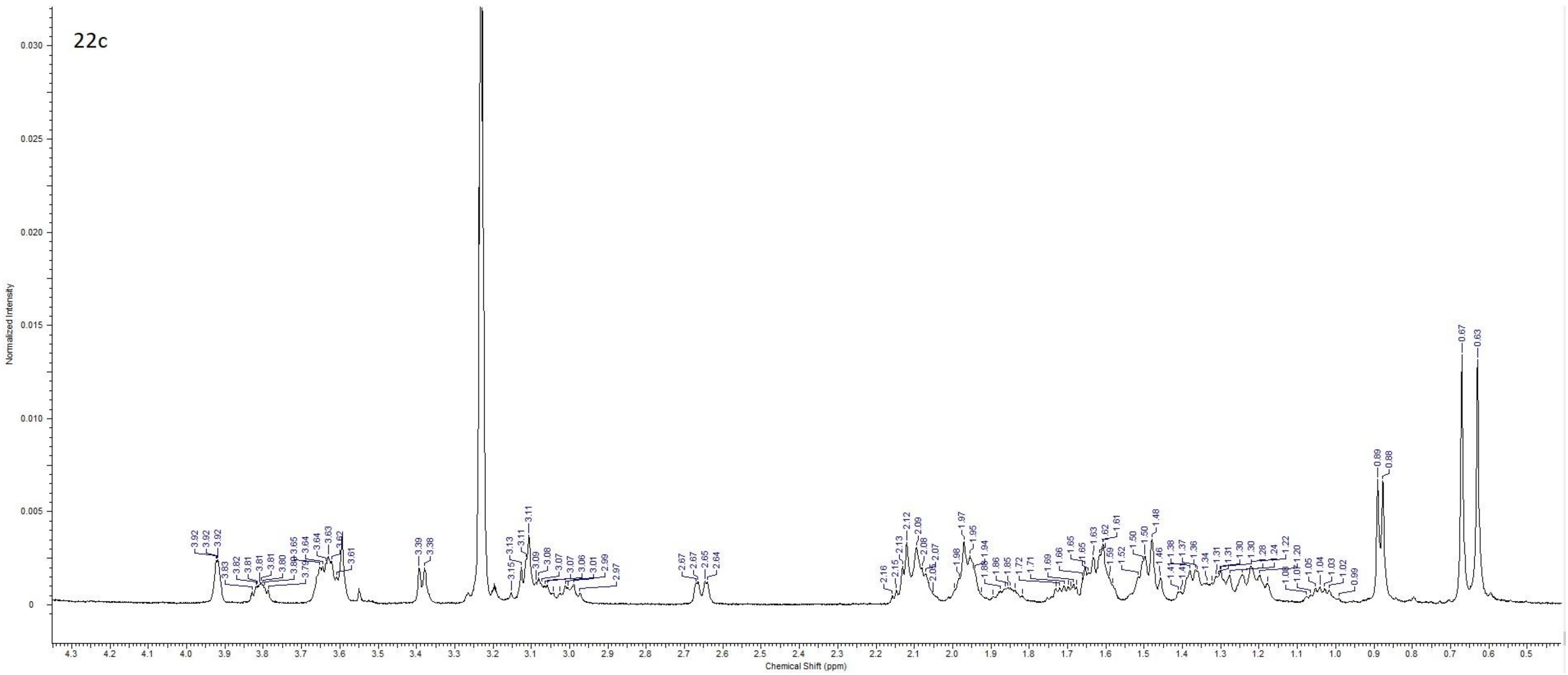
22c
Normalized Intensity
Chemical Shift (ppm)

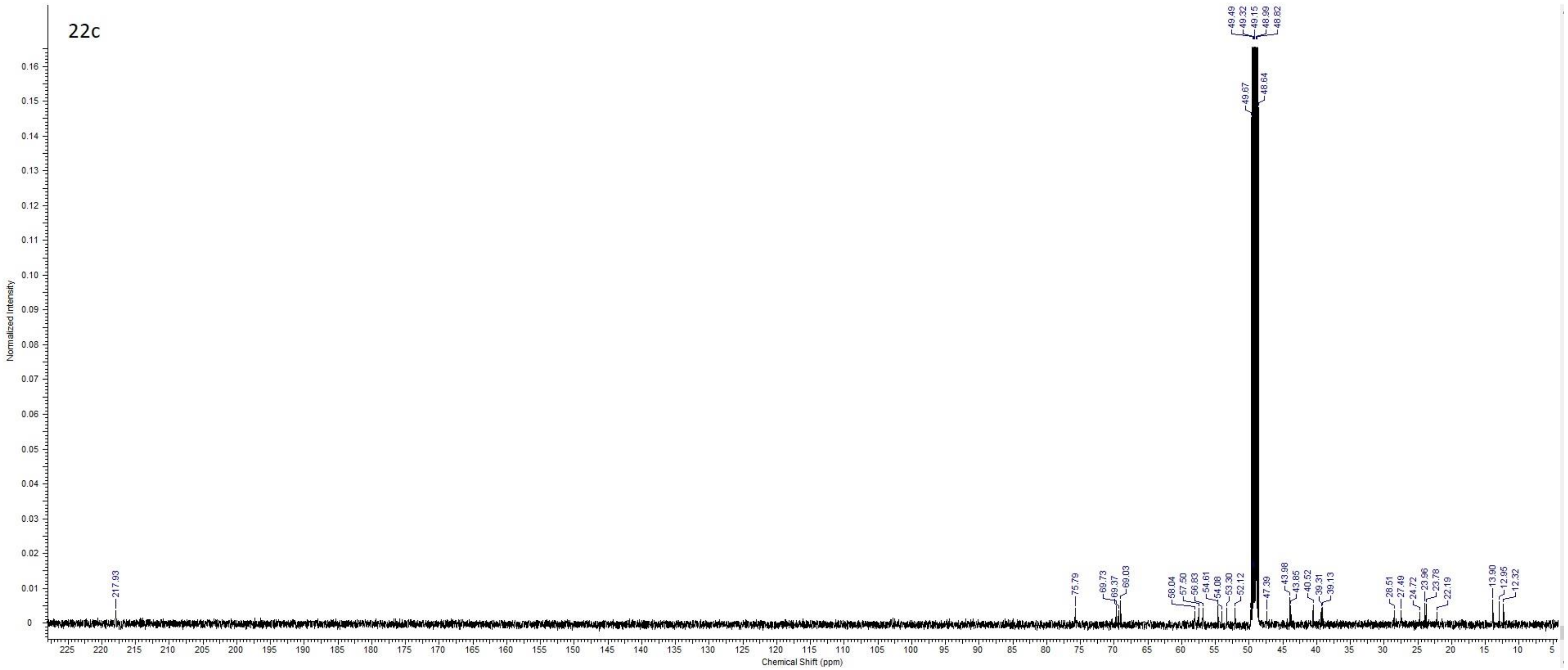

22c
Normalized Intensity
Chemical Shift (ppm)
217.93
75.79
69.73
69.37
69.03
58.04
57.50
56.83
54.61
54.08
53.30
52.12
49.67
49.49
49.32
49.15
48.99
48.82
48.64
47.39
43.98
43.85
40.52
39.31
39.13
28.51
27.49
24.72
23.96
23.78
22.19
13.90
12.95
12.32

Elemental Composition

File Edit View Process Help

Single Mass Analysis
Tolerance = 1.0 PPM / DBE: min = -1.5, max = 50.0
Element prediction: Off
Number of isotope peaks used for i-FIT = 3
Monoisotopic Mass, Even Electron Ions
644 formula(e) evaluated with 1 results within limits (up to 50 best isotopic matches for each mass)
Elements Used:

Mass	Calc. Mass	mDa	PPM	DBE	Formula	i-FIT	i-FIT Norm	Fit Conf %	C	H	N	O
478.3535	478.3532	0.3	0.6	5.5	C28 H48 N O5	34.9	n/a	n/a	28	48	1	5

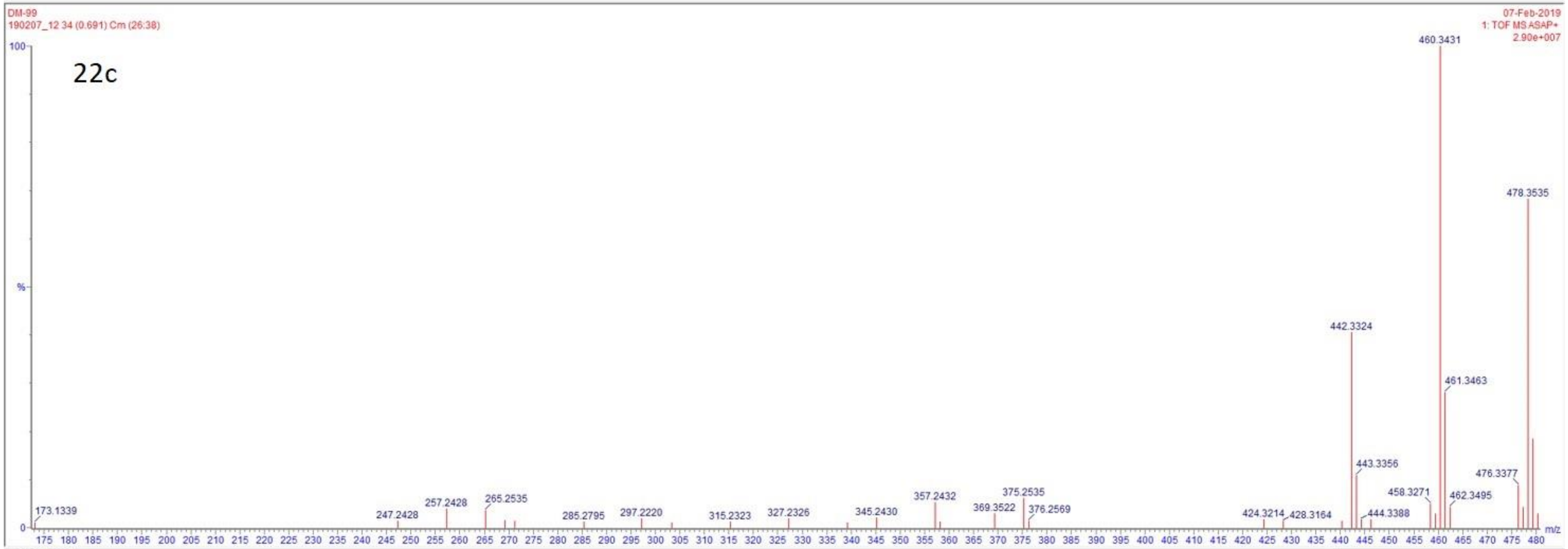

For Help, press F1

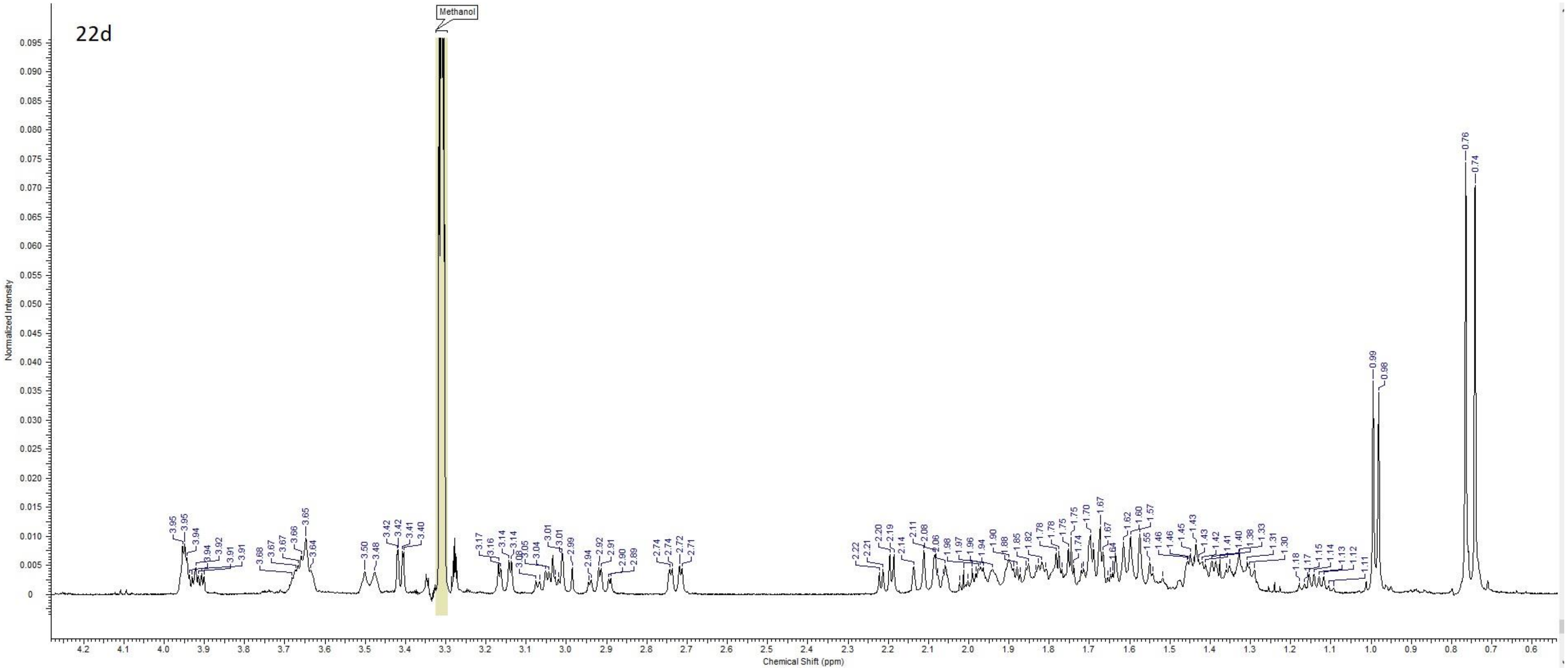

22d
Methanol
Normalized Intensity
Chemical Shift (ppm)

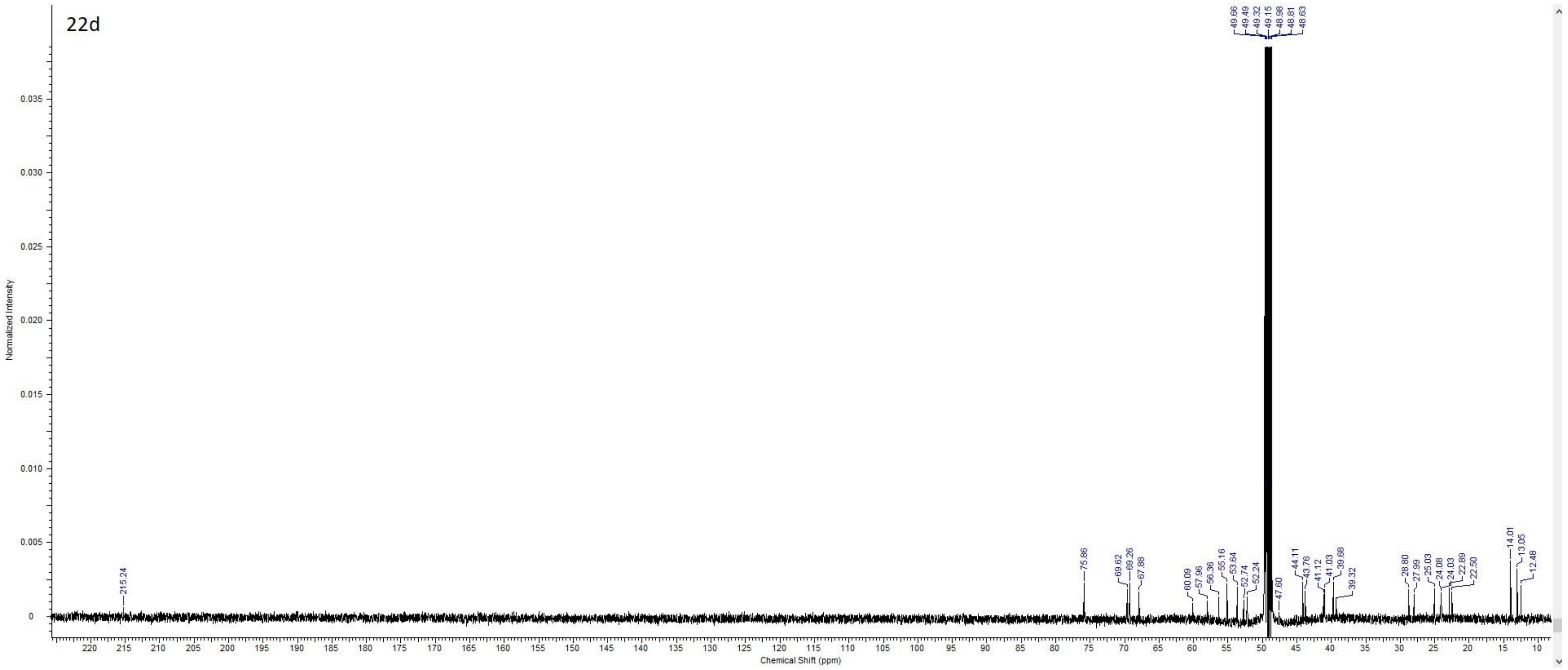

22d
Normalized Intensity
Chemical Shift (ppm)
215.24
75.86
69.62
69.26
67.88
60.09
57.96
56.36
55.16
53.64
52.74
52.24
49.66
49.49
49.32
49.15
48.98
48.81
48.63
47.60
44.11
43.76
41.12
41.03
39.68
39.32
28.80
27.99
25.03
24.08
24.03
22.89
22.50
14.01
13.05
12.48

Elemental Composition

File Edit View Process Help

Single Mass Analysis

Tolerance = 50.0 PPM / DBE: min = -1.5, max = 50.0

Element prediction: Off

Number of isotope peaks used for i-FIT = 3

Monoisotopic Mass, Even Electron Ions

174 formula(e) evaluated with 1 results within limits (all results (up to 1000) for each mass)

Elements Used:

Mass	Calc. Mass	mDa	PPM	DBE	Formula	i-FIT	i-FIT Norm	Fit Conf %	C	H	N	O
492.3686	492.3689	-0.3	-0.6	5.5	C29 H50 N O5	998.4	n/a	n/a	29	50	1	5

DM-103
200526_03 45 (0.896) Cm (45:48)

1: TOF MS ASAP+
5.59e+007

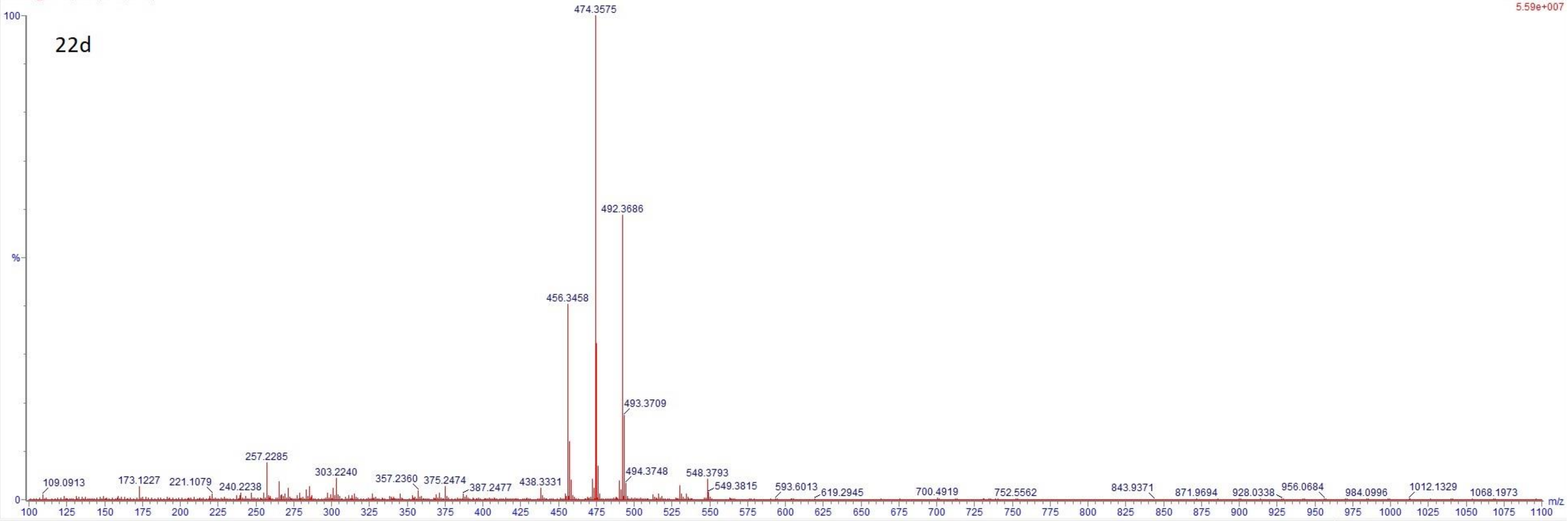

For Help, press F1

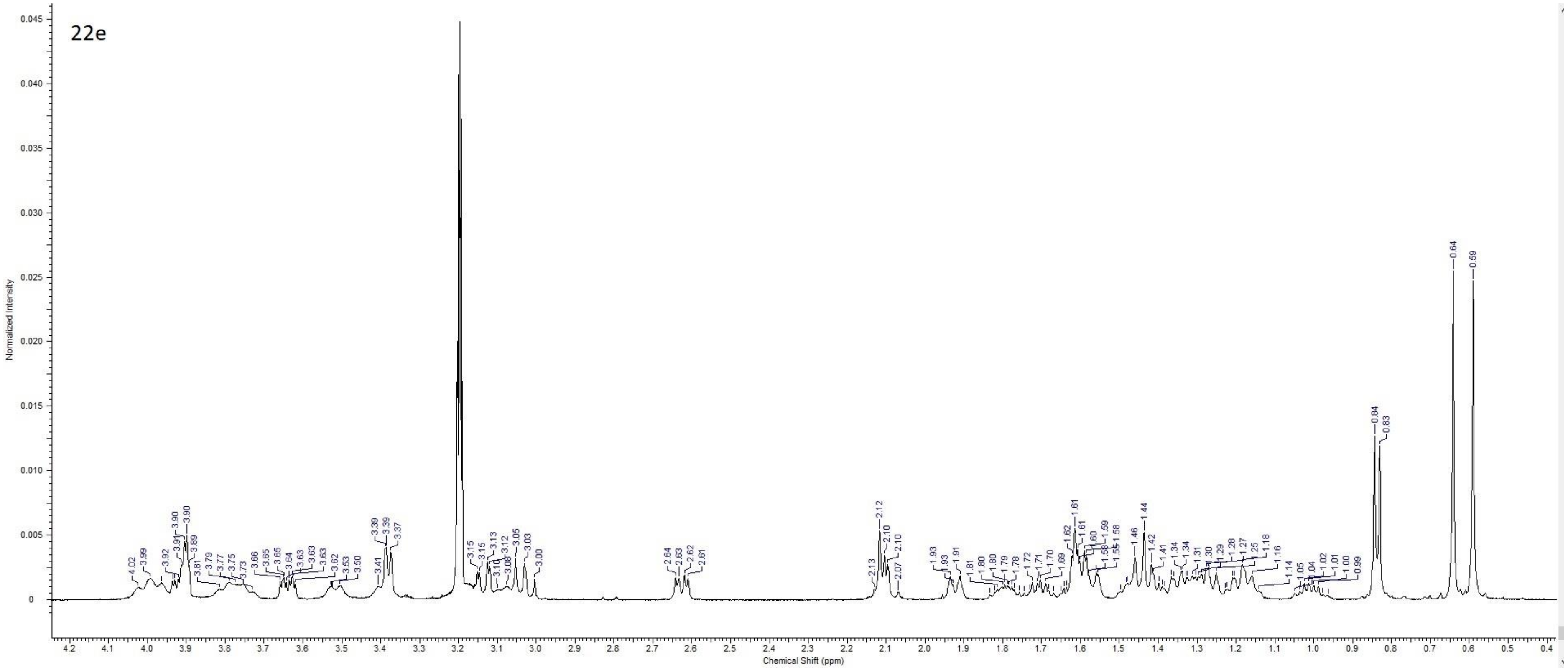
22e
Normalized Intensity
Chemical Shift (ppm)

22e

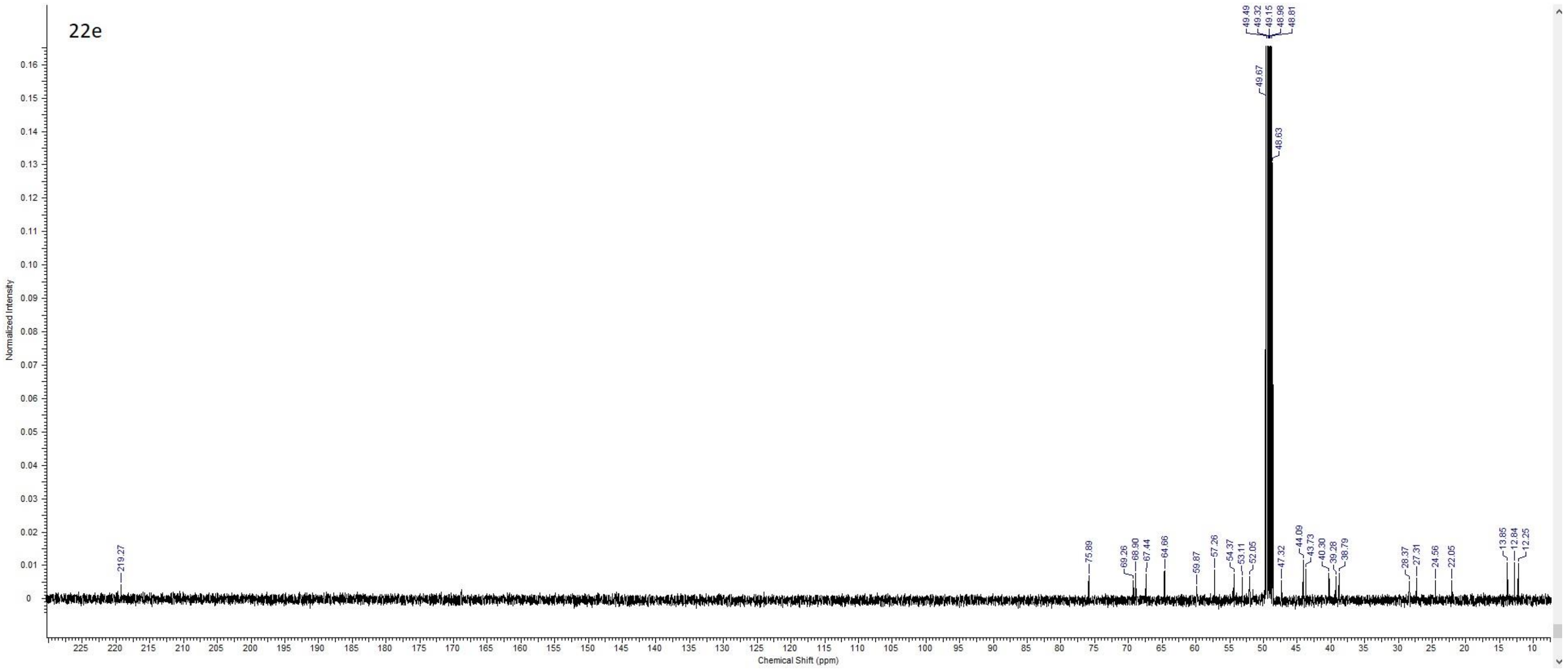

49.49
49.32
49.15
48.98
48.81
49.67
48.63
219.27
75.89
69.26
68.90
67.44
64.66
59.87
57.26
54.37
53.11
52.05
47.32
44.09
43.73
40.30
39.28
38.79
28.37
27.31
24.56
22.05
13.85
12.84
12.25
Normalized Intensity
Chemical Shift (ppm)

Elemental Composition

File Edit View Process Help

Single Mass Analysis
Tolerance = 1.1 PPM / DBE: min = -1.5, max = 50.0
Element prediction: Off
Number of isotope peaks used for i-FIT = 3
Monoisotopic Mass, Even Electron Ions
682 formula(e) evaluated with 1 results within limits (up to 50 best isotopic matches for each mass)
Elements Used:

Mass	Calc. Mass	mDa	PPM	DBE	Formula	i-FIT	i-FIT Norm	Fit Conf %	C	H	N	O
494.3482	494.3482	0.0	0.0	5.5	C28 H48 N O6	27.0	n/a	n/a	28	48	1	6

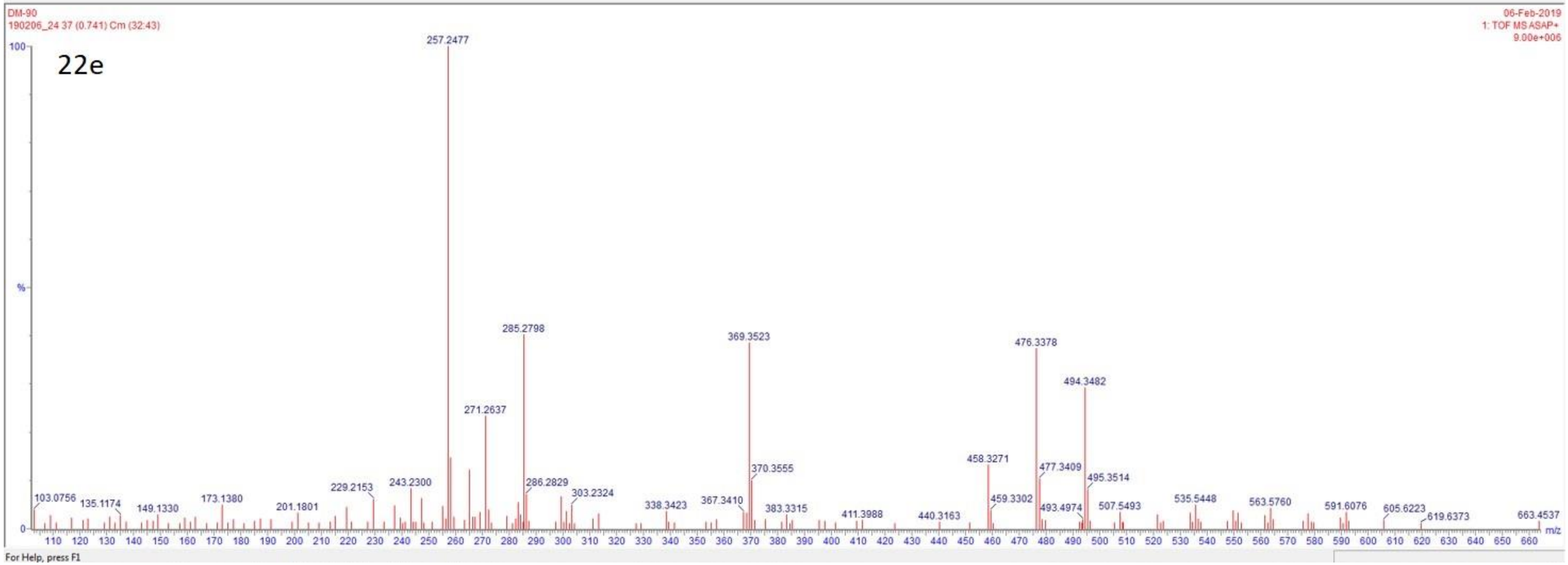

For Help, press F1

23

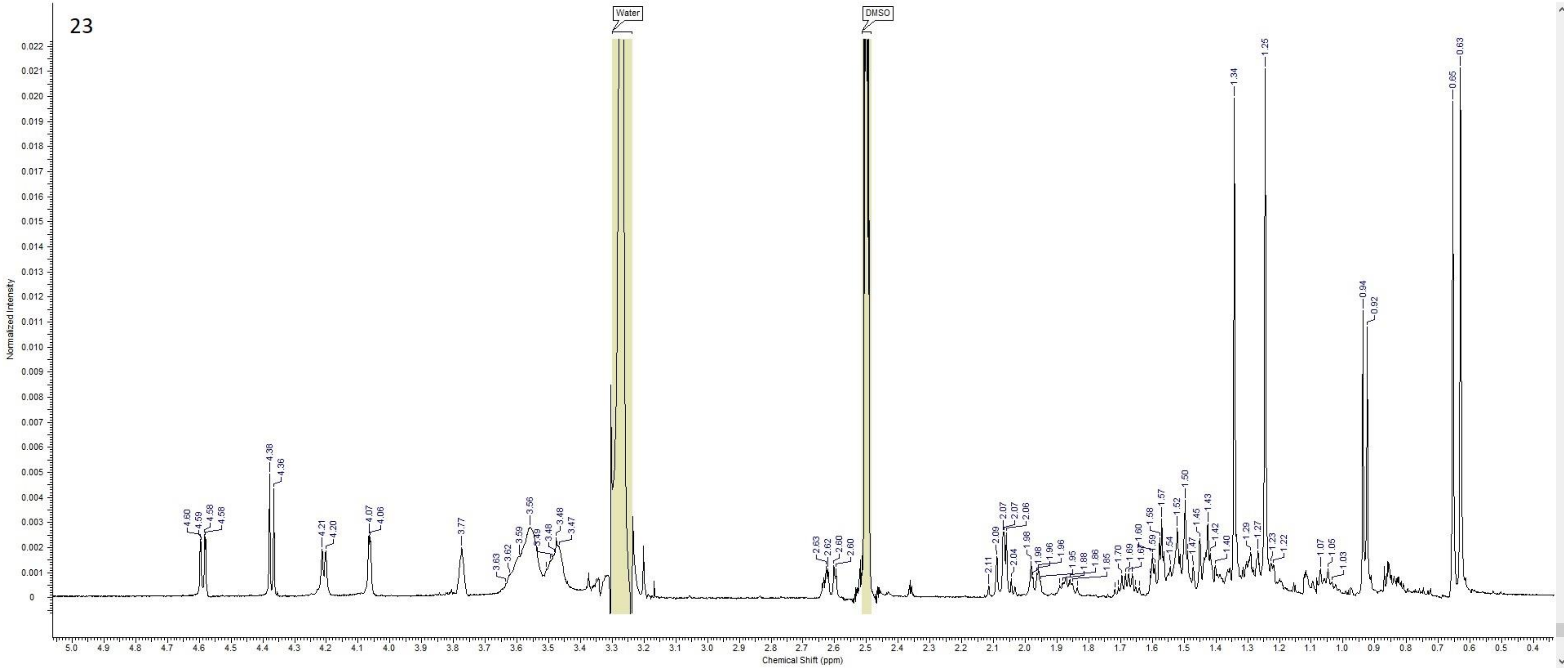

23

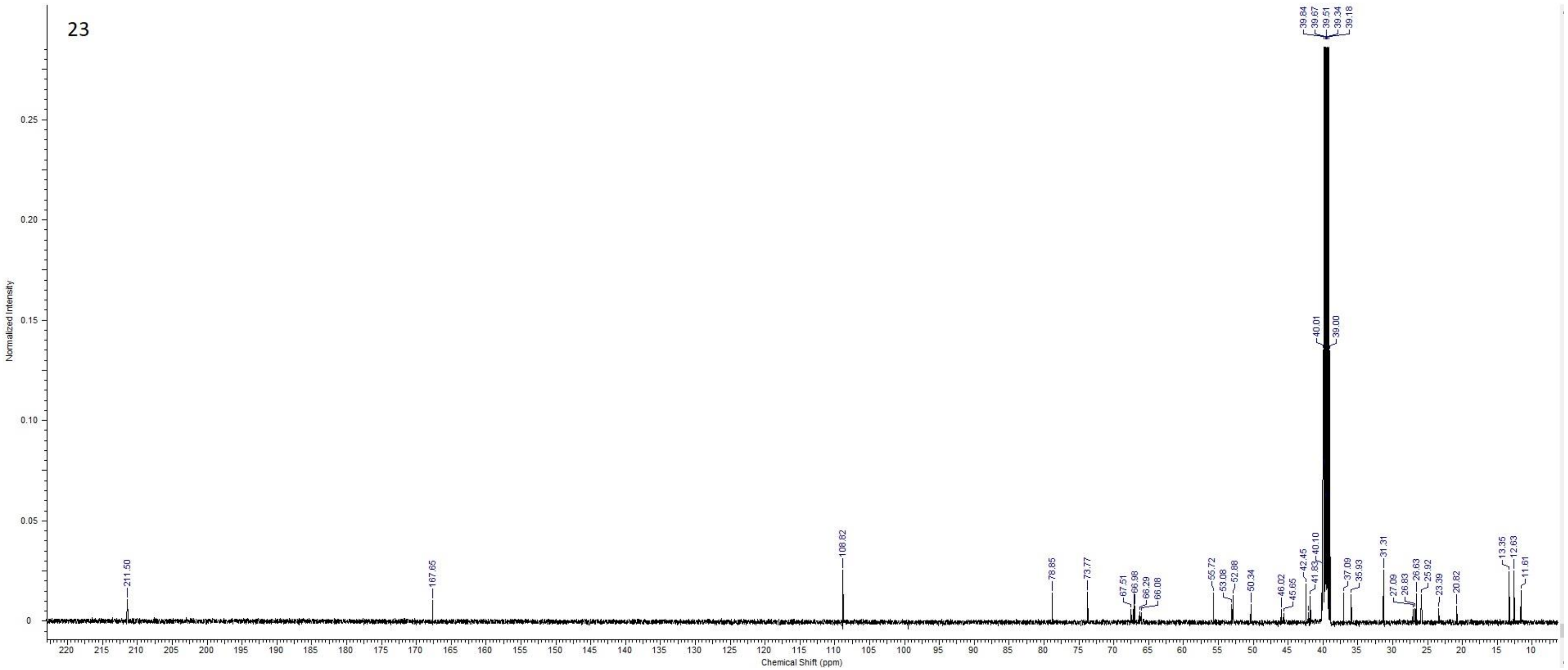

39.84
39.67
39.51
39.34
39.18
40.01
39.00
211.50
167.65
108.82
78.85
73.77
67.51
66.98
66.29
66.08
55.72
53.08
52.88
50.34
46.02
45.65
42.45
41.83
40.10
37.09
35.93
31.31
27.09
26.83
26.63
25.92
23.39
20.82
13.35
12.63
11.61
Normalized Intensity
Chemical Shift (ppm)

Elemental Composition

File Edit View Process Help

Single Mass Analysis
Tolerance = 1.0 PPM / DBE: min = -1.5, max = 50.0
Element prediction: Off
Number of isotope peaks used for i-FIT = 3
Monoisotopic Mass, Even Electron Ions
738 formula(e) evaluated with 1 results within limits (up to 50 best isotopic matches for each mass)
Elements Used:

Mass	Calc. Mass	mDa	PPM	DBE	Formula	i-FIT	i-FIT Norm	Fit Conf %	C	H	N	O
548.3586	548.3587	-0.1	-0.2	7.5	C31 H50 N O7	832.8	n/a	n/a	31	50	1	7

23

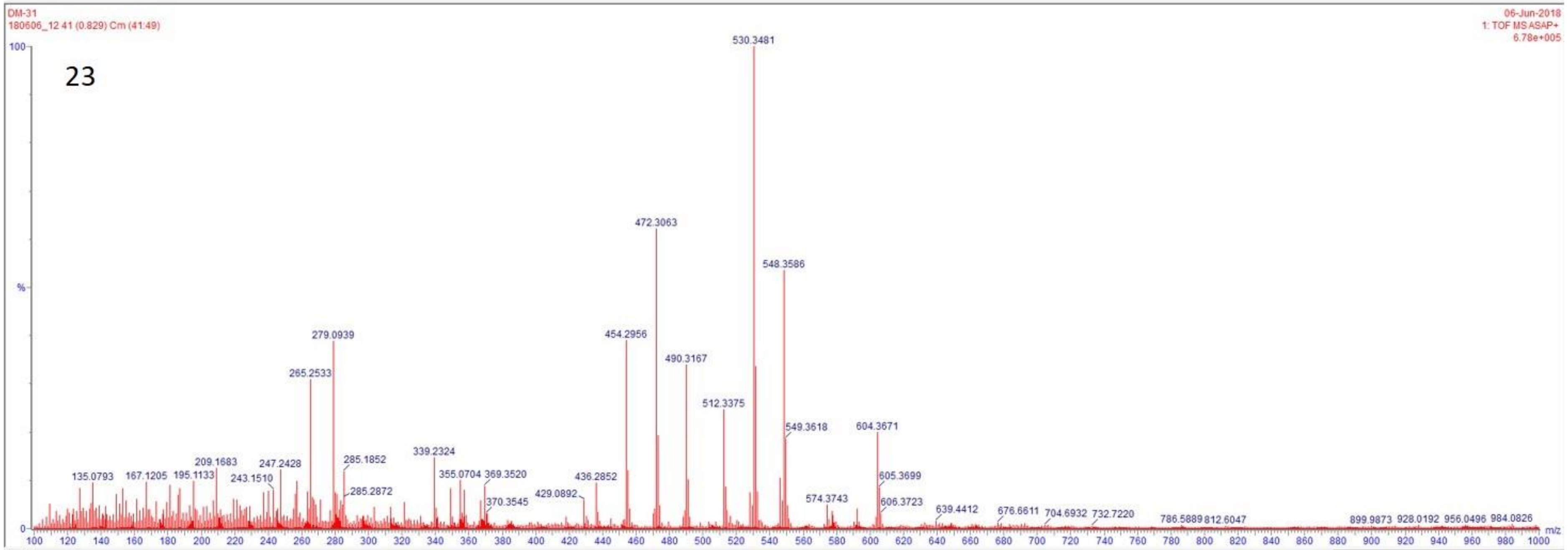

For Help, press F1

24

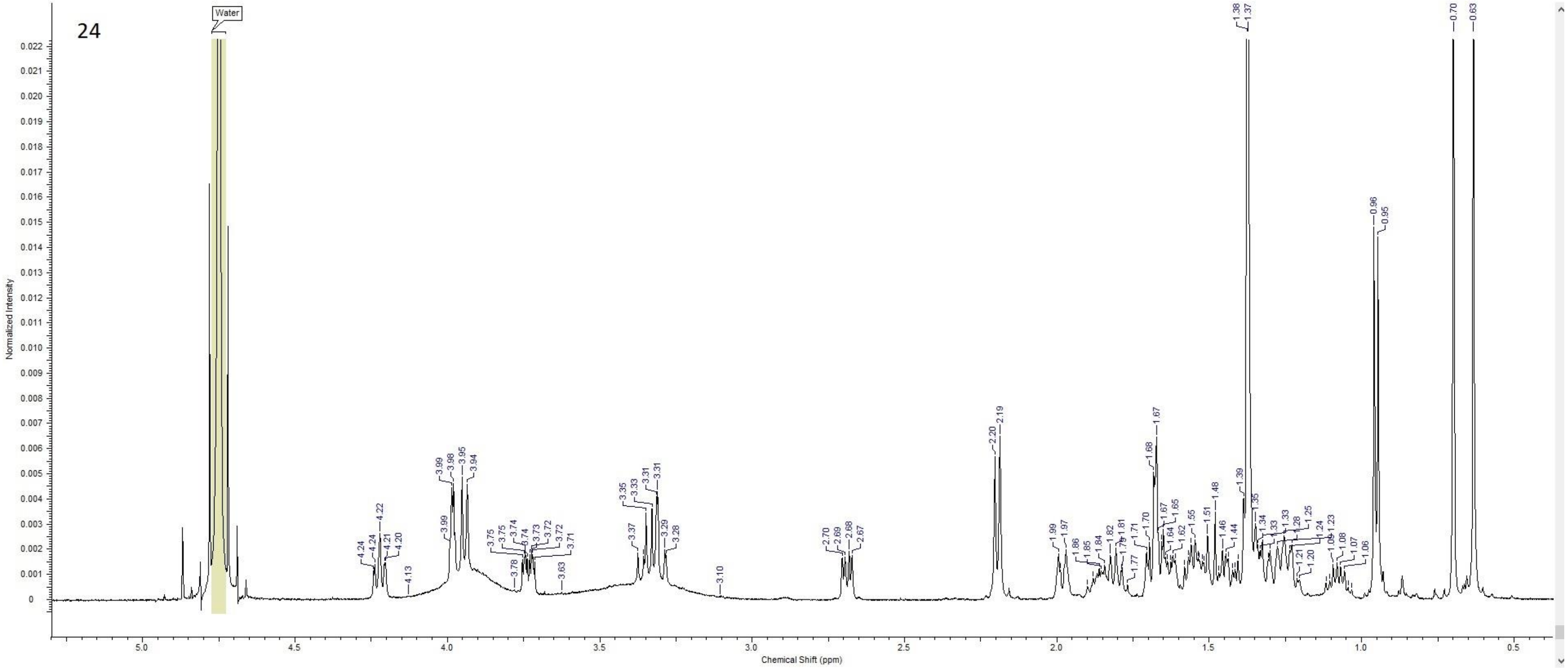

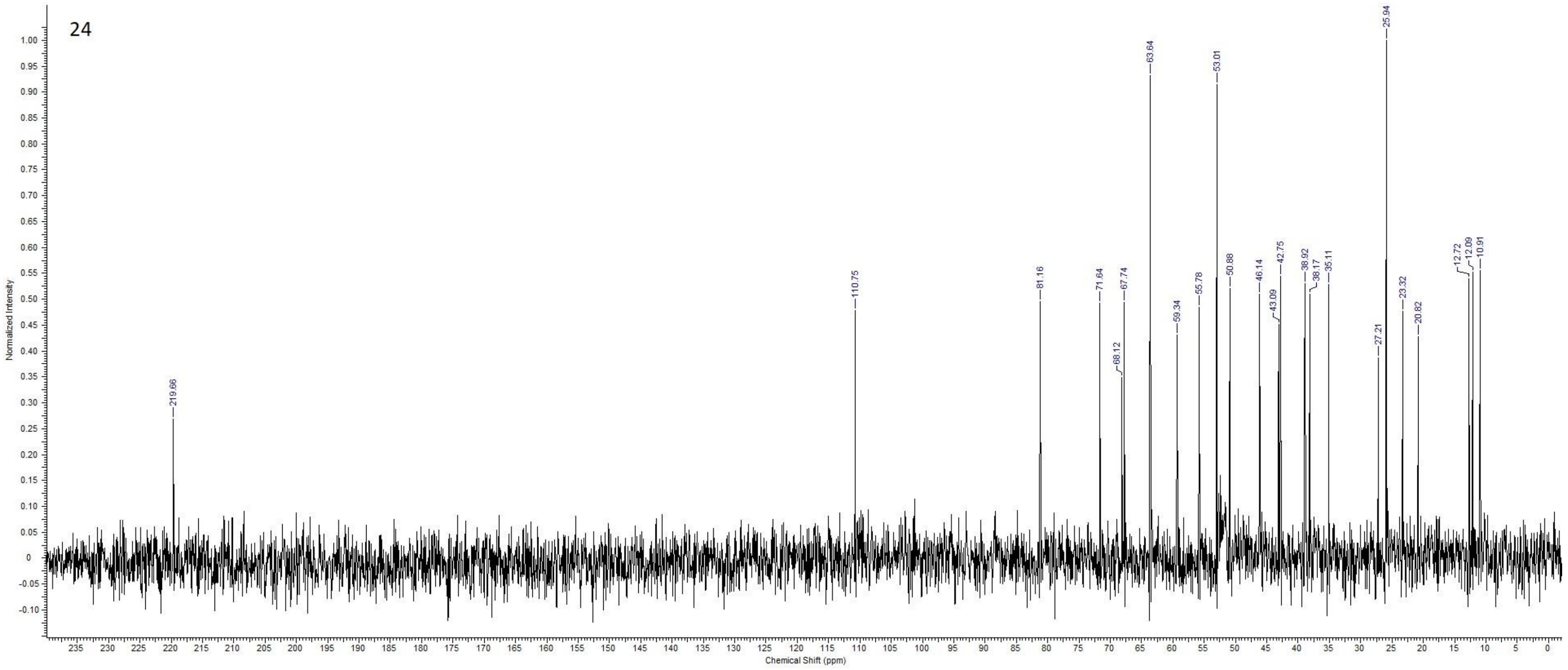
24
Normalized Intensity
Chemical Shift (ppm)
219.66
110.75
81.16
71.64
68.12
67.74
63.64
59.34
55.78
53.01
50.88
46.14
43.09
42.75
38.92
38.17
35.11
27.21
25.94
23.32
20.82
12.72
12.09
10.91

Elemental Composition

File Edit View Process Help

Single Mass Analysis
Tolerance = 1.1 PPM / DBE: min = -1.5, max = 50.0
Element prediction: Off
Number of isotope peaks used for i-FIT = 3
Monoisotopic Mass, Even Electron Ions
794 formula(e) evaluated with 1 results within limits (up to 50 best isotopic matches for each mass)
Elements Used:

Mass	Calc. Mass	mDa	PPM	DBE	Formula	i-FIT	i-FIT Norm	Fit Conf %	C	H	N	O
534.3796	534.3795	0.1	0.2	6.5	C31 H52 N O6	84.2	n/a	n/a	31	52	1	6

24

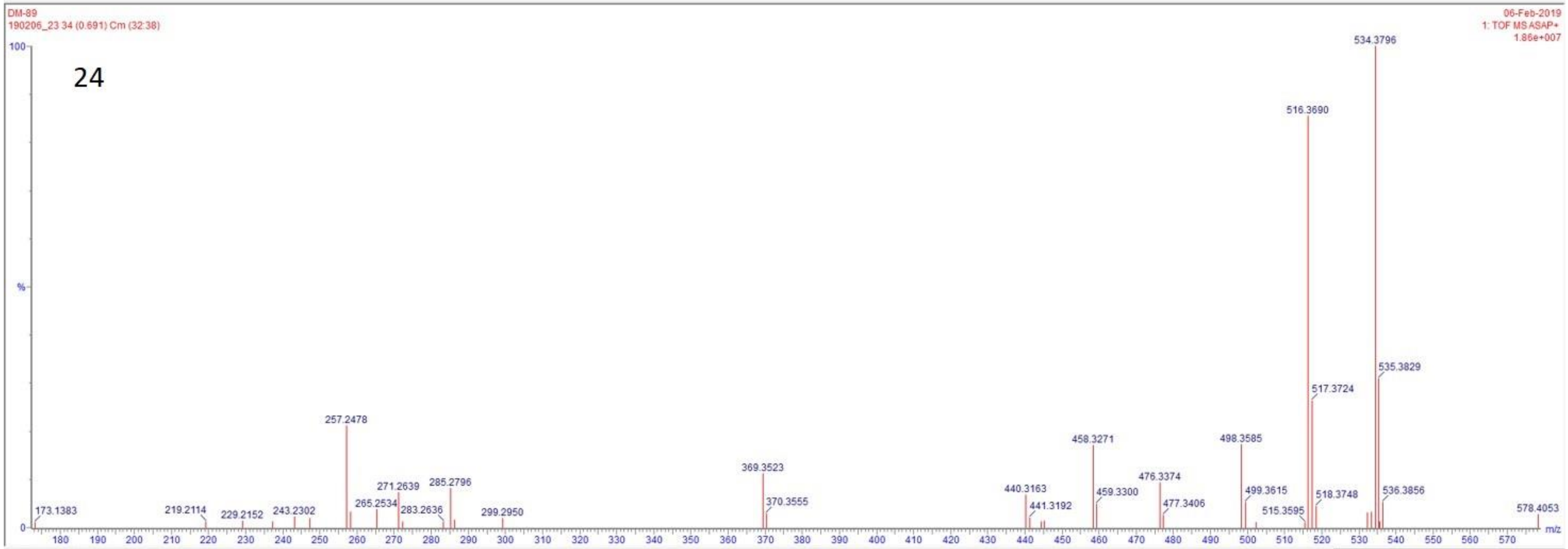

For Help, press F1
